# Supplementary material for: Does Connected Health Technology Improve Health-Related Outcomes in Rural Cardiac Populations? Systematic Review Narrative Synthesis
Source: Int J Environ Res Public Health. 2022 Feb 17;19(4):2302. doi: 10.3390/ijerph19042302 (PMC8871734; doi:10.3390/ijerph19042302)
Supplement: Supplementary file 1 [file ijerph-19-02302-s001.zip › Supplementary Material S1.pdf]

Supplementary Material - Search strategies

CINAHL Search

Accessibility Information and Tips

Print Search History

EBSCOhost Thursday, May 27, 2021 5:21:30 PM

#	Query	Limiters/Expanders	Last Run Via	Results
S50	S9 AND S18 AND S28 AND S39 AND S48	Limiters - Published Date: 19900101-20211231; English Language Expanders - Apply equivalent subjects Search modes - Boolean/Phrase	Interface - EBSCOhost Research Databases Search Screen - Basic Search Database - CINAHL Complete	132
S49	S9 AND S18 AND S28 AND S39	Limiters - Published Date: 19900101-20211231; English Language Expanders - Apply equivalent subjects Search modes - Boolean/Phrase	Interface - EBSCOhost Research Databases Search Screen - Basic Search Database - CINAHL Complete	296
S48	S40 OR S41 OR S42 OR S43 OR S44 OR S45 OR S46 OR S47	Expanders - Apply equivalent subjects Search modes - Boolean/Phrase	Interface - EBSCOhost Research Databases Search Screen - Basic Search Database - CINAHL Complete	222,850
S47	Wii	Expanders - Apply equivalent subjects	Interface - EBSCOhost	0

		Search modes - Boolean/Phrase	Research Databases Search Screen - Basic Search Database - CINAHL Complete	
S46	Fitbit* OR “Smart watch*" OR Garmin OR “Apple watch”	Expanders - Apply equivalent subjects Search modes - Boolean/Phrase	Interface - EBSCOhost Research Databases Search Screen - Basic Search Database - CINAHL Complete	192
S45	(MH "Pedometers") OR pedometer* OR Step count*	Expanders - Apply equivalent subjects Search modes - Boolean/Phrase	Interface - EBSCOhost Research Databases Search Screen - Basic Search Database - CINAHL Complete	3,174
S44	"Accelerometer" OR (MH "Accelerometer*")	Expanders - Apply equivalent subjects Search modes - Boolean/Phrase	Interface - EBSCOhost Research Databases Search Screen - Basic Search Database - CINAHL Complete	5,797
S43	(MH "Monitoring, Physiologic") OR “activity monitor*” OR monitor* OR monitoring OR "heart monitor"	Expanders - Apply equivalent subjects Search modes - Boolean/Phrase	Interface - EBSCOhost Research Databases Search Screen - Basic Search Database - CINAHL Complete	190,712

S42	(MH "Fitness Trackers") OR Fitness tracker* OR Activity tracker*OR “fitness activity tracker*” OR tracker* OR tracking	Expanders - Apply equivalent subjects Search modes - Boolean/Phrase	Interface - EBSCOhost Research Databases Search Screen - Basic Search Database - CINAHL Complete	20,926
S41	(MH "Assistive Technology") OR (MH "Assistive Technology Services")	Expanders - Apply equivalent subjects Search modes - Boolean/Phrase	Interface - EBSCOhost Research Databases Search Screen - Basic Search Database - CINAHL Complete	4,090
S40	(MH "Wearable Sensors") OR wearable* OR “wearable technolog*” OR “wearable fit* technolog*”	Expanders - Apply equivalent subjects Search modes - Boolean/Phrase	Interface - EBSCOhost Research Databases Search Screen - Basic Search Database - CINAHL Complete	4,588
S39	S29 OR S30 OR S31 OR S32 OR S33 OR S34 OR S35 OR S36 OR S37 OR S38	Expanders - Apply equivalent subjects Search modes - Boolean/Phrase	Interface - EBSCOhost Research Databases Search Screen - Basic Search Database - CINAHL Complete	1,497,269
S38	(MH "Internet+") OR "Internet" OR (MH "Internet Connections") OR (MH "Internet-Based Intervention") OR (MH "Internet of Things") OR (MH "World Wide Web Applications+") OR (MH "World Wide Web+") OR world wide web OR www	Expanders - Apply equivalent subjects Search modes - Boolean/Phrase	Interface - EBSCOhost Research Databases Search Screen - Basic Search Database -	171,337

			CINAHL Complete	
S37	App* OR electronic application* OR internet app*	Expanders - Apply equivalent subjects Search modes - Boolean/Phrase	Interface - EBSCOhost Research Databases Search Screen - Basic Search Database - CINAHL Complete	1,214,408
S36	(MH "World Wide Web Applications") OR "Mobile application" OR (MH "Cellular Phone+")	Expanders - Apply equivalent subjects Search modes - Boolean/Phrase	Interface - EBSCOhost Research Databases Search Screen - Basic Search Database - CINAHL Complete	18,517
S35	(MH "Smartphone") OR “Smart phone*”	Expanders - Apply equivalent subjects Search modes - Boolean/Phrase	Interface - EBSCOhost Research Databases Search Screen - Basic Search Database - CINAHL Complete	3,665
S34	(MH "Telerehabilitation") OR Telerehabilitation	Expanders - Apply equivalent subjects Search modes - Boolean/Phrase	Interface - EBSCOhost Research Databases Search Screen - Basic Search Database - CINAHL Complete	656
S33	(MH Technology) OR technolog*	Expanders - Apply equivalent subjects Search modes - Boolean/Phrase	Interface - EBSCOhost Research Databases Search Screen - Basic	192,821

			Search Database - CINAHL Complete	
S32	(MH Telemedicine) OR telemedicine*	Expanders - Apply equivalent subjects Search modes - Boolean/Phrase	Interface - EBSCOhost Research Databases Search Screen - Basic Search Database - CINAHL Complete	21,309
S31	(MH Telehealth) OR telehealth or eHealth OR mHealth	Expanders - Apply equivalent subjects Search modes - Boolean/Phrase	Interface - EBSCOhost Research Databases Search Screen - Basic Search Database - CINAHL Complete	23,693
S30	(MH Digital technology) OR "Digital technolog*)	Expanders - Apply equivalent subjects Search modes - Boolean/Phrase	Interface - EBSCOhost Research Databases Search Screen - Basic Search Database - CINAHL Complete	2,491
S29	Digital health	Expanders - Apply equivalent subjects Search modes - Boolean/Phrase	Interface - EBSCOhost Research Databases Search Screen - Basic Search Database - CINAHL Complete	1,985
S28	S19 OR S20 OR S21 OR S22 OR S23 OR S24 OR S25 OR S26 OR S27	Expanders - Apply equivalent subjects Search modes - Boolean/Phrase	Interface - EBSCOhost Research Databases	89,254

			Search Screen - Basic Search Database - CINAHL Complete	
S27	"Remote Consultation*"	Expanders - Apply equivalent subjects Search modes - Boolean/Phrase	Interface - EBSCOhost Research Databases Search Screen - Basic Search Database - CINAHL Complete	2,520
S26	(Healthcar* AND (rural* OR remote))	Expanders - Apply equivalent subjects Search modes - Boolean/Phrase	Interface - EBSCOhost Research Databases Search Screen - Basic Search Database - CINAHL Complete	6,833
S25	(MH "Remote Consultation") OR "Remote healthcare" OR "remote area*"	Expanders - Apply equivalent subjects Search modes - Boolean/Phrase	Interface - EBSCOhost Research Databases Search Screen - Basic Search Database - CINAHL Complete	4,534
S24	(MH "Rural Health Centers") OR (MH "Rural Health Services")	Expanders - Apply equivalent subjects Search modes - Boolean/Phrase	Interface - EBSCOhost Research Databases Search Screen - Basic Search Database - CINAHL Complete	7,342
S23	(MH "Rural Health Personnel") OR “rural health personnel”	Expanders - Apply equivalent subjects	Interface - EBSCOhost	680

		Search modes - Boolean/Phrase	Research Databases Search Screen - Basic Search Database - CINAHL Complete	
S22	MH Rural population OR “rural population*”	Expanders - Apply equivalent subjects Search modes - Boolean/Phrase	Interface - EBSCOhost Research Databases Search Screen - Basic Search Database - CINAHL Complete	12,741
S21	MH Rural areas OR “rural area*”	Expanders - Apply equivalent subjects Search modes - Boolean/Phrase	Interface - EBSCOhost Research Databases Search Screen - Basic Search Database - CINAHL Complete	32,497
S20	MH Rural health OR “rural health”	Expanders - Apply equivalent subjects Search modes - Boolean/Phrase	Interface - EBSCOhost Research Databases Search Screen - Basic Search Database - CINAHL Complete	17,693
S19	(Rural* OR remote)	Expanders - Apply equivalent subjects Search modes - Boolean/Phrase	Interface - EBSCOhost Research Databases Search Screen - Basic Search Database - CINAHL Complete	89,254

S18	S10 OR S11 OR S12 OR S13 OR S14 OR S15 OR S16 OR S17	Expanders - Apply equivalent subjects<br>Search modes - Boolean/Phrase	Interface - EBSCOhost Research Databases<br>Search Screen - Basic Search<br>Database - CINAHL Complete	379,209
S17	"Fit Homes"	Expanders - Apply equivalent subjects<br>Search modes - Boolean/Phrase	Interface - EBSCOhost Research Databases<br>Search Screen - Basic Search<br>Database - CINAHL Complete	0
S16	(MH "Therapeutic Exercise+") OR "Therapeutic exercise"	Expanders - Apply equivalent subjects<br>Search modes - Boolean/Phrase	Interface - EBSCOhost Research Databases<br>Search Screen - Basic Search<br>Database - CINAHL Complete	57,708
S15	(MH "Home Physical Therapy") OR "Home physical therapy"	Expanders - Apply equivalent subjects<br>Search modes - Boolean/Phrase	Interface - EBSCOhost Research Databases<br>Search Screen - Basic Search<br>Database - CINAHL Complete	529
S14	(MH "Home Rehabilitation+") OR "Home rehabilitation"	Expanders - Apply equivalent subjects<br>Search modes - Boolean/Phrase	Interface - EBSCOhost Research Databases<br>Search Screen - Basic Search<br>Database -	2,345

			CINAHL Complete	
S13	Home-base* AND (intervention OR programme OR rehabilitation)	Expanders - Apply equivalent subjects Search modes - Boolean/Phrase	Interface - EBSCOhost Research Databases Search Screen - Basic Search Database - CINAHL Complete	5,031
S12	"Home-based exercise*"	Expanders - Apply equivalent subjects Search modes - Boolean/Phrase	Interface - EBSCOhost Research Databases Search Screen - Basic Search Database - CINAHL Complete	689
S11	House-care OR in-house care	Expanders - Apply equivalent subjects Search modes - Boolean/Phrase	Interface - EBSCOhost Research Databases Search Screen - Basic Search Database - CINAHL Complete	335
S10	Home* OR home-based OR house* OR indoor OR HB	Expanders - Apply equivalent subjects Search modes - Boolean/Phrase	Interface - EBSCOhost Research Databases Search Screen - Basic Search Database - CINAHL Complete	325,551
S9	S1 OR S2 OR S3 OR S4 OR S5 OR S6 OR S7 OR S8	Expanders - Apply equivalent subjects Search modes - Boolean/Phrase	Interface - EBSCOhost Research Databases Search Screen - Basic	570,180

			Search Database - CINAHL Complete	
S8	(MH "Heart+") OR "heart"	Expanders - Apply equivalent subjects Search modes - Boolean/Phrase	Interface - EBSCOhost Research Databases Search Screen - Basic Search Database - CINAHL Complete	307,865
S7	exCR	Expanders - Apply equivalent subjects Search modes - Boolean/Phrase	Interface - EBSCOhost Research Databases Search Screen - Basic Search Database - CINAHL Complete	2
S6	CR	Expanders - Apply equivalent subjects Search modes - Boolean/Phrase	Interface - EBSCOhost Research Databases Search Screen - Basic Search Database - CINAHL Complete	20,012
S5	"Coronary"	Expanders - Apply equivalent subjects Search modes - Boolean/Phrase	Interface - EBSCOhost Research Databases Search Screen - Basic Search Database - CINAHL Complete	126,869
S4	(MH "Conditioning, Cardiopulmonary") OR "Cardiopulmonary Conditioning"	Expanders - Apply equivalent subjects Search modes - Boolean/Phrase	Interface - EBSCOhost Research Databases	216

			Search Screen - Basic Search Database - CINAHL Complete	
S3	(MH "Rehabilitation, Cardiac+") OR "cardiac disease rehabilitation"	Expanders - Apply equivalent subjects Search modes - Boolean/Phrase	Interface - EBSCOhost Research Databases Search Screen - Basic Search Database - CINAHL Complete	4,675
S2	Cardiac OR "cardiac intervention"	Expanders - Apply equivalent subjects Search modes - Boolean/Phrase	Interface - EBSCOhost Research Databases Search Screen - Basic Search Database - CINAHL Complete	159,044
S1	(MH "Cardiovascular System") OR cardiovascular	Expanders - Apply equivalent subjects Search modes - Boolean/Phrase	Interface - EBSCOhost Research Databases Search Screen - Basic Search Database - CINAHL Complete	190,763

Cochrane Library search

[Note: 456 before filters, 411 after]
Date Run: 09/06/2021 09:55:53
ID Search Hits
#1 "cardiovascular system" OR cardiovascular* OR cardiac OR "cardiac intervention*" OR "cardiac rehabilitation" OR "cardiac disease rehabilitation" OR "cardiopulmonary condition*" OR coronary OR CR OR exCR OR heart 276129

#2 ((home* OR home-based OR house* OR indoor OR HB) AND (intervention OR programme OR rehabilitation or exercise*)) OR “house-care” OR “in house care” OR "home physical therapy" OR "therapeutic exercise*" OR "Fit*Homes" 62102
#3 (rural* OR remote) OR “rural health” OR “rural area*” OR “rural population*” OR “rural health personnel” OR "rural health cent*" OR "rural health service*" OR "remote consultation" OR "remote healthcare" OR "remote area*" OR (healthcar* AND (rural* OR remote)) 16887
#4 digital health OR “digital technolog*” OR telehealth OR eHealth OR mHealth OR telemedicine* OR technolog* OR telerehabilitation OR “smart phone*” "World Wide Web applications" OR "mobile application*" OR "cellular phone*" OR app* OR “electronic application*” OR “internet app*” OR internet OR "internet connections" OR "internet-based intervention" OR "internet of things" OR "World Wide Web application*" OR "World Wide Web" OR www 589418
#5 "wearable sensors" OR wearable* OR “wearable technolog*” OR “wearable fit* technolog*” OR "assistive technolog*" OR "assistive technology service*" OR "fitness tracker*" OR “activity tracker*” OR “fitness activity tracker*” OR tracker* OR tracking OR "physiologic* monitoring" OR “activity monitor*” OR monitor* OR "heart monitor*" OR accelerometer OR pedomet* OR "step count*" OR fitbit OR "smart watch*" OR Garmin OR “Apple watch” OR Wii 114492
#6 #1 AND #2 AND #3 AND #4 AND #5 with Cochrane Library publication date Between Jan 1990 and Jan 2021, in Cochrane Reviews, Trials

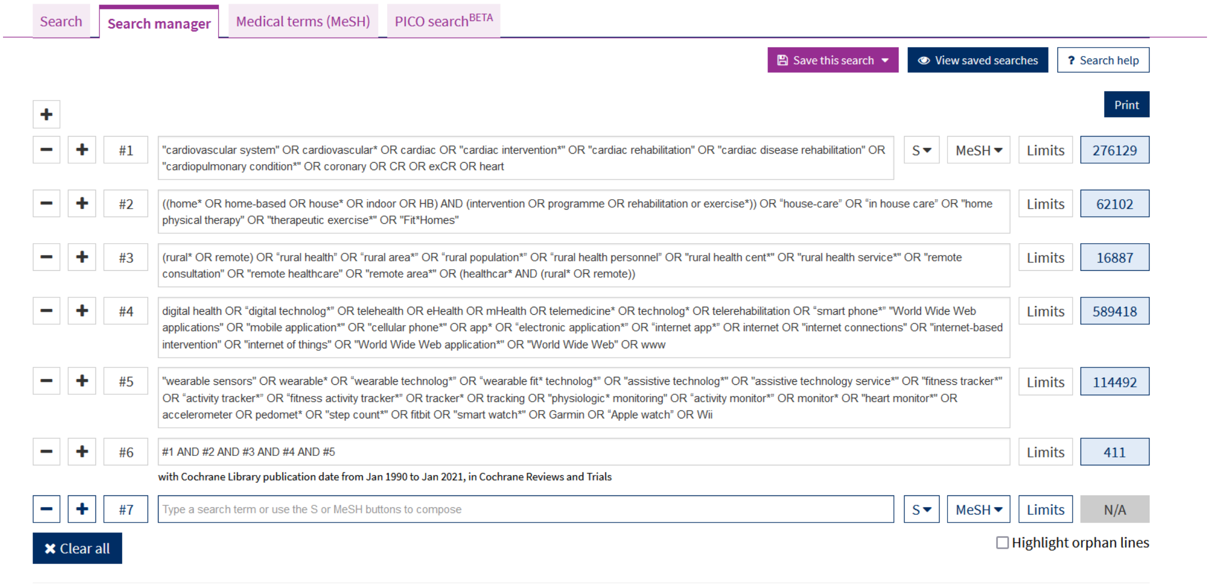

Google Scholar search pattern

<u>Search pattern:</u>

(cardio* OR cardiac OR coronary) AND ((home* OR house*) AND (intervention OR programme OR rehabilitation or exercise*)) AND (rural* OR remote) AND (digital health OR telehealth OR eHealth OR mHealth OR telemedicine* OR telerehabilitation) AND ("wearable sensors" OR “wearable technolog*” OR “OR "fitness tracker*" OR “activity tracker*” OR "physiologic* monitoring" OR

“activity monitor*” OR "heart monitor" OR accelerometer OR pedomet* OR “step count*” OR fitbit* OR “smart watch*" OR Garmin OR “Apple watch” OR Wii)

Notes: 1,590 results before Language and date filters, 1,550 results after.
I imported 658 into RefWorks from this, accounting for the fact that I could only access 1,000 in their system, and the ones I didn't were so obviously off-topic. Erred on the side of caution and keeping them in, but the nature of Google Scholar meant that extraneous content and other elements... essentially things have changed whereby I had to export each citation individually into RefWorks, so culled some pre-emptively that were totally off topic, rather than leave for you to have this discretion.

Embase Search Pattern

1 exp cardiovascular system/ 1700919
2 (cardiovascular or Cardiac or "cardiac intervention").mp. [mp=title, abstract, heading word, drug trade name, original title, device manufacturer, drug manufacturer, device trade name, keyword, floating subheading word, candidate term word] 1872455
3 exp heart rehabilitation/ or Rehabilitation, Cardiac.mp. 12703
4 Conditioning, Cardiopulmonary.mp. 3
5 exp coronary artery constriction/ or Coronary.mp. or exp coronary artery disease/ or exp coronary artery/ 747856
6 CR.mp. 374233
7 exCR.mp. 24
8 Heart.mp. or exp heart/ or exp heart rehabilitation/2554092
9 1 or 2 or 3 or 4 or 5 or 6 or 7 or 8 4413517
10 (Home* or home-based or house* or indoor or HB).mp. [mp=title, abstract, heading word, drug trade name, original title, device manufacturer, drug manufacturer, device trade name, keyword, floating subheading word, candidate term word] 1190602
11 (House-care or in-house care).mp. [mp=title, abstract, heading word, drug trade name, original title, device manufacturer, drug manufacturer, device trade name, keyword, floating subheading word, candidate term word] 42
12 "Home-based exercise*".mp. [mp=title, abstract, heading word, drug trade name, original title, device manufacturer, drug manufacturer, device trade name, keyword, floating subheading word, candidate term word] 1630
13 (Home-base* and (intervention or programme or rehabilitation)).mp. [mp=title, abstract, heading word, drug trade name, original title, device manufacturer, drug manufacturer, device trade name, keyword, floating subheading word, candidate term word] 8207
14 Home Rehabilitation.mp. or exp home rehabilitation/ 1159
15 Home Physical Therapy.mp. or exp home physiotherapy/ 376
16 Therapeutic Exercise.mp. or exp kinesiotherapy/ 84642
17 "Fit Homes".mp. 0
18 10 or 11 or 12 or 13 or 14 or 15 or 16 or 17 1267895

19 (Rural* or remote).mp. [mp=title, abstract, heading word, drug trade name, original title, device manufacturer, drug manufacturer, device trade name, keyword, floating subheading word, candidate term word] 295905
20 Rural health.mp. or exp rural health/ 19696
21 Rural areas.mp. or exp rural area/ 78008
22 Rural population.mp. or exp rural population/ 51849
23 Rural Health Personnel.mp. 13
24 Rural Health Centers.mp. or exp rural health care/ 14129
25 Remote Consultation.mp. or exp teleconsultation/ 11770
26 ("Remote healthcare" or "remote area*").mp. [mp=title, abstract, heading word, drug trade name, original title, device manufacturer, drug manufacturer, device trade name, keyword, floating subheading word, candidate term word] 7177
27 (Healthcar* and (rural* or remote)).mp. [mp=title, abstract, heading word, drug trade name, original title, device manufacturer, drug manufacturer, device trade name, keyword, floating subheading word, candidate term word] 15030
28 19 or 20 or 21 or 22 or 23 or 24 or 25 or 26 or 27 305187
29 Digital health.mp. 3431
30 Digital technolog*.mp. [mp=title, abstract, heading word, drug trade name, original title, device manufacturer, drug manufacturer, device trade name, keyword, floating subheading word, candidate term word] 3443
31 Telehealth.mp. or exp telehealth/ 59520
32 Telemedicine.mp. or exp telemedicine/ 54745
33 (eHealth or mHealth).mp. [mp=title, abstract, heading word, drug trade name, original title, device manufacturer, drug manufacturer, device trade name, keyword, floating subheading word, candidate term word] 9954
34 exp technology/ or Technology.mp. 661199
35 exp smartphone/ or Smartphone.mp. 21763
36 Cellular Phone.mp. 868
37 World Wide Web Applications.mp. 3
38 Mobile application.mp. or exp mobile application/ 16440
39 App.mp. 41608
40 electronic application.mp. 168
41 internet app.mp. 8
42 Internet.mp. or exp Internet/ 143599
43 Internet-Based Intervention.mp. or exp web-based intervention/ 1432
44 ("Internet Connections" or "Internet of Things" or "World Wide Web" or www).mp. [mp=title, abstract, heading word, drug trade name, original title, device manufacturer, drug manufacturer, device trade name, keyword, floating subheading word, candidate term word] 9938
45 29 or 30 or 31 or 32 or 33 or 34 or 35 or 36 or 37 or 38 or 39 or 40 or 41 or 42 or 43 or 44 888115
46 Assistive Technology.mp. or exp assistive technology/ 4784
47 Assistive Technology Services.mp. 49
48 Fitness Trackers.mp. or exp activity tracker/ 3781
49 Monitoring, Physiologic.mp. or exp physiologic monitoring/ 7723
50 Accelerometer.mp. or exp accelerometer/ 20137
51 Pedometers.mp. or exp pedometer/ 3192

52 Step count*.mp. [mp=title, abstract, heading word, drug trade name, original title, device manufacturer, drug manufacturer, device trade name, keyword, floating subheading word, candidate term word] 3302
53 Wii.mp. 1433
54 46 or 47 or 48 or 49 or 50 or 51 or 53 37423
55 9 and 18 and 28 and 45 and 54 53
56 limit 55 to (human and yr="1990 -Current") 48

[In table form]

Ovid® | Wolters Kluwer
My Account Support & Training Contact your Library Service Help Feedback Logoff
Search Journals Books Multimedia My Workspace What's New
▼ Search History (56) View Saved

# ▲	Searches	Results	Type	Actions
1	exp cardiovascular system/	1700919	Advanced	Display Results More
2	(cardiovascular or Cardiac or "cardiac intervention").mp. [mp=title, abstract, heading word, drug trade name, original title, device manufacturer, drug manufacturer, device trade name, keyword, floating subheading word, candidate term word]	1872455	Advanced	Display Results More
3	exp heart rehabilitation/ or Rehabilitation, Cardiac.mp.	12703	Advanced	Display Results More
4	Conditioning, Cardiopulmonary.mp.	3	Advanced	Display Results More
5	exp coronary artery constriction/ or Coronary.mp. or exp coronary artery disease/ or exp coronary artery/	747856	Advanced	Display Results More
6	CR.mp.	374233	Advanced	Display Results More
7	exCR.mp.	24	Advanced	Display Results More
8	Heart.mp. or exp heart/ or exp heart rehabilitation/	2554092	Advanced	Display Results More
9	1 or 2 or 3 or 4 or 5 or 6 or 7 or 8	4413517	Advanced	Display Results More
10	(Home* or home-based or house* or indoor or HB).mp. [mp=title, abstract, heading word, drug trade name, original title, device manufacturer, drug manufacturer, device trade name, keyword, floating subheading word, candidate term word]	1190602	Advanced	Display Results More
11	(House-care or in-house care).mp. [mp=title, abstract, heading word, drug trade name, original title, device manufacturer, drug manufacturer, device trade name, keyword, floating subheading word, candidate term word]	42	Advanced	Display Results More
12	"Home-based exercise*".mp. [mp=title, abstract, heading word, drug trade name, original title, device manufacturer, drug manufacturer, device trade name, keyword, floating subheading word, candidate term word]	1630	Advanced	Display Results More
13	(Home-base* and (intervention or programme or rehabilitation)).mp. [mp=title, abstract, heading word, drug trade name, original title, device manufacturer, drug manufacturer, device trade name, keyword, floating subheading word, candidate term word]	8207	Advanced	Display Results More
14	Home Rehabilitation.mp. or exp home rehabilitation/	1159	Advanced	Display Results More
15	Home Physical Therapy.mp. or exp home physiotherapy/	376	Advanced	Display Results More
16	Therapeutic Exercise.mp. or exp kinesiotherapy/	84642	Advanced	Display Results More
17	"Fit Homes".mp.	0	Advanced	Save More
18	10 or 11 or 12 or 13 or 14 or 15 or 16 or 17	1267895	Advanced	Display Results More
19	(Rural* or remote).mp. [mp=title, abstract, heading word, drug trade name, original title, device manufacturer, drug manufacturer, device trade name, keyword, floating subheading word, candidate term word]	295905	Advanced	Display Results More
20	Rural health.mp. or exp rural health/	19696	Advanced	Display Results More
21	Rural areas.mp. or exp rural area/	78008	Advanced	Display Results More
22	Rural population.mp. or exp rural population/	51849	Advanced	Display Results More
23	Rural Health Personnel.mp.	13	Advanced	Display Results More
24	Rural Health Centers.mp. or exp rural health care/	14129	Advanced	Display Results More
25	Remote Consultation.mp. or exp teleconsultation/	11770	Advanced	Display Results More
26	("Remote healthcare" or "remote area*").mp. [mp=title, abstract, heading word, drug trade name, original title, device manufacturer, drug manufacturer, device trade name, keyword, floating subheading word, candidate term word]	7177	Advanced	Display Results More
27	(Healthcar* and (rural* or remote)).mp. [mp=title, abstract, heading word, drug trade name, original title, device manufacturer, drug manufacturer, device trade name, keyword, floating subheading word, candidate term word]	15030	Advanced	Display Results More
28	19 or 20 or 21 or 22 or 23 or 24 or 25 or 26 or 27	305187	Advanced	Display Results More
29	Digital health.mp.	3431	Advanced	Display Results More
30	Digital technolog*.mp. [mp=title, abstract, heading word, drug trade name, original title, device manufacturer, drug manufacturer, device trade name, keyword, floating subheading word, candidate term word]	3443	Advanced	Display Results More
31	Telehealth.mp. or exp telehealth/	59520	Advanced	Display Results More
32	Telemedicine.mp. or exp telemedicine/	54745	Advanced	Display Results More
33	(eHealth or mHealth).mp. [mp=title, abstract, heading word, drug trade name, original title, device manufacturer, drug manufacturer, device trade name, keyword, floating subheading word, candidate term word]	9954	Advanced	Display Results More
34	exp technology/ or Technology.mp.	661199	Advanced	Display Results More
35	exp smartphone/ or Smartphone.mp.	21763	Advanced	Display Results More
36	Cellular Phone.mp.	868	Advanced	Display Results More
37	World Wide Web Applications.mp.	3	Advanced	Display Results More
38	Mobile application.mp. or exp mobile application/	16440	Advanced	Display Results More
39	App.mp.	41608	Advanced	Display Results More
40	electronic application.mp.	168	Advanced	Display Results More
41	internet app.mp.	8	Advanced	Display Results More
42	Internet.mp. or exp Internet/	143599	Advanced	Display Results More
43	Internet-Based Intervention.mp. or exp web-based intervention/	1432	Advanced	Display Results More
44	("Internet Connections" or "Internet of Things" or "World Wide Web" or www).mp. [mp=title, abstract, heading word, drug trade name, original title, device manufacturer, drug manufacturer, device trade name, keyword, floating subheading word, candidate term word]	9938	Advanced	Display Results More
45	29 or 30 or 31 or 32 or 33 or 34 or 35 or 36 or 37 or 38 or 39 or 40 or 41 or 42 or 43 or 44	888115	Advanced	Display Results More
46	Assistive Technology.mp. or exp assistive technology/	4784	Advanced	Display Results More
47	Assistive Technology Services.mp.	49	Advanced	Display Results More
48	Fitness Trackers.mp. or exp activity tracker/	3781	Advanced	Display Results More
49	Monitoring, Physiologic.mp. or exp physiologic monitoring/	7723	Advanced	Display Results More
50	Accelerometer.mp. or exp accelerometer/	20137	Advanced	Display Results More
51	Pedometers.mp. or exp pedometer/	3192	Advanced	Display Results More
52	Step count*.mp. [mp=title, abstract, heading word, drug trade name, original title, device manufacturer, drug manufacturer, device trade name, keyword, floating subheading word, candidate term word]	3302	Advanced	Display Results More
53	Wii.mp.	1433	Advanced	Display Results More
54	46 or 47 or 48 or 49 or 50 or 51 or 53	37423	Advanced	Display Results More
55	9 and 18 and 28 and 45 and 54	53	Advanced	Display Results More
56	limit 55 to (human and yr="1990 -Current")	48	Advanced	Display Results More

Ovid Medline search pattern

1 Cardiovascular System.mp. or exp Cardiovascular System/ 1289081
2 (Cardiac or "cardiac intervention" or cardiovascular).mp. [mp=title, abstract, original title, name of substance word, subject heading word, floating sub-heading word, keyword

heading word, organism supplementary concept word, protocol supplementary concept word, rare disease supplementary concept word, unique identifier, synonyms] 1296273
3 Rehabilitation, Cardiac.mp. or exp Cardiac Rehabilitation/ 2806
4 "cardiac disease rehabilitation".mp. [mp=title, abstract, original title, name of substance word, subject heading word, floating sub-heading word, keyword heading word, organism supplementary concept word, protocol supplementary concept word, rare disease supplementary concept word, unique identifier, synonyms] 0
5 Cardiopulmonary Conditioning.mp. 10
6 Coronary.mp. 520644
7 CR.mp. 91755
8 exCR.mp. 17
9 Heart.mp. or exp Heart/ 1394197
10 1 or 2 or 3 or 4 or 5 or 6 or 7 or 8 or 9 2896038
11 (Home* or home-based or house* or indoor or HB).mp. [mp=title, abstract, original title, name of substance word, subject heading word, floating sub-heading word, keyword heading word, organism supplementary concept word, protocol supplementary concept word, rare disease supplementary concept word, unique identifier, synonyms] 884252
12 (House-care or in-house care).mp. [mp=title, abstract, original title, name of substance word, subject heading word, floating sub-heading word, keyword heading word, organism supplementary concept word, protocol supplementary concept word, rare disease supplementary concept word, unique identifier, synonyms] 30
13 "Home-based exercise*".mp. [mp=title, abstract, original title, name of substance word, subject heading word, floating sub-heading word, keyword heading word, organism supplementary concept word, protocol supplementary concept word, rare disease supplementary concept word, unique identifier, synonyms] 1158
14 (Home-base* and (intervention or programme or rehabilitation)).mp. [mp=title, abstract, original title, name of substance word, subject heading word, floating sub-heading word, keyword heading word, organism supplementary concept word, protocol supplementary concept word, rare disease supplementary concept word, unique identifier, synonyms] 5924
15 Home Rehabilitation.mp. 421
16 Home Physical Therapy.mp. 27
17 exp Physical Therapy Modalities/ or exp Exercise Therapy/ or Therapeutic Exercise.mp. 161423
18 "Fit Homes".mp. [mp=title, abstract, original title, name of substance word, subject heading word, floating sub-heading word, keyword heading word, organism supplementary concept word, protocol supplementary concept word, rare disease supplementary concept word, unique identifier, synonyms] 0
19 11 or 12 or 13 or 14 or 15 or 16 or 17 or 18 1037142
20 (Rural* or remote).mp. [mp=title, abstract, original title, name of substance word, subject heading word, floating sub-heading word, keyword heading word, organism supplementary concept word, protocol supplementary concept word, rare disease supplementary concept word, unique identifier, synonyms] 253055
21 Rural health.mp. or exp Rural Health/ or exp Health Services Accessibility/152257
22 Rural areas.mp. 33034
23 Rural population.mp. or exp Rural Population/ 66630
24 Rural Health Personnel.mp. 14

25 Rural Health Centers.mp. or exp Rural Health Services/ 13516
26 Remote Consultation.mp. or exp Remote Consultation/ 5583
27 Remote healthcare.mp. or exp Rural Health Services/ 13416
28 (Healthcar* and (rural* or remote)).mp. [mp=title, abstract, original title, name of substance word, subject heading word, floating sub-heading word, keyword heading word, organism supplementary concept word, protocol supplementary concept word, rare disease supplementary concept word, unique identifier, synonyms] 12124
29 20 or 21 or 22 or 23 or 24 or 25 or 26 or 27 or 28 359730
30 Digital health.mp. 3187
31 Digital technology.mp. or exp Digital Technology/ 1756
32 Telehealth.mp. or exp Telemedicine/38039
33 (eHealth or mHealth).mp. [mp=title, abstract, original title, name of substance word, subject heading word, floating sub-heading word, keyword heading word, organism supplementary concept word, protocol supplementary concept word, rare disease supplementary concept word, unique identifier, synonyms] 9958
34 Telemedicine.mp. or exp Telemedicine/ 41250
35 exp Technology/ or Technology.mp. 791122
36 Telerehabilitation.mp. or exp Telerehabilitation/ 1330
37 Smartphone.mp. or exp Cell Phone/ or exp Smartphone/ 24524
38 World Wide Web Applications.mp. 4
39 Mobile application.mp. or exp Mobile Applications/9180
40 (App* or electronic application* or internet app*).mp. [mp=title, abstract, original title, name of substance word, subject heading word, floating sub-heading word, keyword heading word, organism supplementary concept word, protocol supplementary concept word, rare disease supplementary concept word, unique identifier, synonyms] 7123110
41 Internet.mp. or exp Internet Access/ or exp "Internet of Things"/ or exp Internet/ or exp Internet-Based Intervention/ 119383
42 ("World Wide Web Applications" or "World Wide Web" or "world wide web" or www).mp. [mp=title, abstract, original title, name of substance word, subject heading word, floating sub-heading word, keyword heading word, organism supplementary concept word, protocol supplementary concept word, rare disease supplementary concept word, unique identifier, synonyms] 4415
43 30 or 31 or 32 or 33 or 34 or 35 or 36 or 37 or 38 or 39 or 40 or 41 or 42 7682185
44 exp Monitoring, Ambulatory/ or Wearable Sensors.mp. or exp Biosensing Techniques/ or exp Wearable Electronic Devices/ 104048
45 (wearable* or wearable technolog* or wear* fit* technolog*).mp. [mp=title, abstract, original title, name of substance word, subject heading word, floating sub-heading word, keyword heading word, organism supplementary concept word, protocol supplementary concept word, rare disease supplementary concept word, unique identifier, synonyms] 17122
46 Assistive Technology.mp. or exp Self-Help Devices/ 13055
47 exp Self-Help Devices/ or Assistive Technology Services.mp. 12034
48 Fitness Trackers.mp. or exp Fitness Trackers/ 927
49 (Fitness tracker* or Activity tracker* or fitness activity tracker* or tracker* or tracking).mp. [mp=title, abstract, original title, name of substance word, subject heading word, floating sub-heading word, keyword heading word, organism supplementary concept word

word, protocol supplementary concept word, rare disease supplementary concept word, unique identifier, synonyms] 96145
50 Monitoring, Physiologic.mp. or exp Monitoring, Physiologic/ 181516
51 (activity monitor* or monitor* or monitoring or heart monitor).mp. [mp=title, abstract, original title, name of substance word, subject heading word, floating sub-heading word, keyword heading word, organism supplementary concept word, protocol supplementary concept word, rare disease supplementary concept word, unique identifier, synonyms] 1032538
52 exp Accelerometry/ or Accelerometer.mp. 18101
53 (pedometer* or Step count*).mp. [mp=title, abstract, original title, name of substance word, subject heading word, floating sub-heading word, keyword heading word, organism supplementary concept word, protocol supplementary concept word, rare disease supplementary concept word, unique identifier, synonyms] 4308
54 (Fitbit* or Smart watch* or Garmin or Apple watch).mp. [mp=title, abstract, original title, name of substance word, subject heading word, floating sub-heading word, keyword heading word, organism supplementary concept word, protocol supplementary concept word, rare disease supplementary concept word, unique identifier, synonyms] 1157
55 Wii.mp. [mp=title, abstract, original title, name of substance word, subject heading word, floating sub-heading word, keyword heading word, organism supplementary concept word, protocol supplementary concept word, rare disease supplementary concept word, unique identifier, synonyms] 917
56 44 or 45 or 46 or 47 or 48 or 49 or 50 or 51 or 52 or 53 or 54 or 55 1240773
57 10 and 19 and 29 and 43 and 56 488

[Or, screen capture for table}

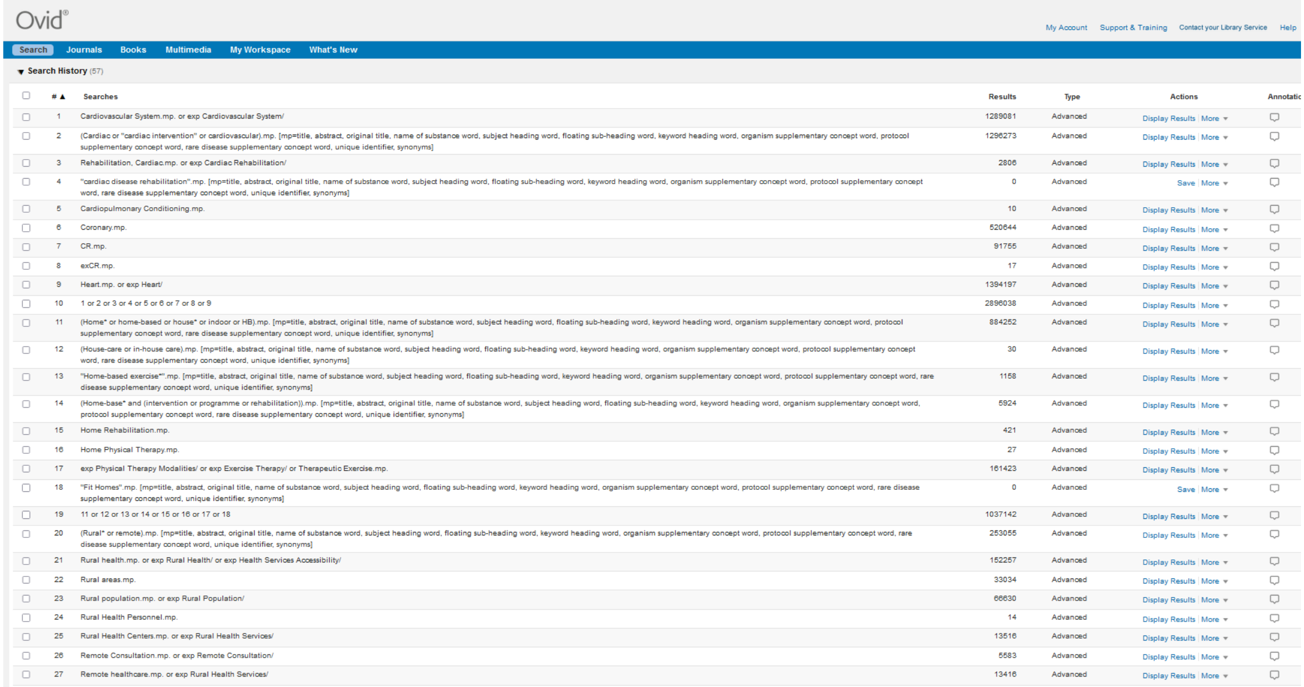

#	Searches	Results	Type	Actions	Annotations
1	Cardiovascular System.mp. or exp Cardiovascular System/	1289081	Advanced	Display Results | More	
2	(Cardiac or "cardiac intervention" or cardiovascular).mp. [mp=title, abstract, original title, name of substance word, subject heading word, floating sub-heading word, keyword heading word, organism supplementary concept word, protocol supplementary concept word, rare disease supplementary concept word, unique identifier, synonyms]	1296273	Advanced	Display Results | More	
3	Rehabilitation, Cardiac.mp. or exp Cardiac Rehabilitation/	2806	Advanced	Display Results | More	
4	"cardiac disease rehabilitation".mp. [mp=title, abstract, original title, name of substance word, subject heading word, floating sub-heading word, keyword heading word, organism supplementary concept word, protocol supplementary concept word, rare disease supplementary concept word, unique identifier, synonyms]	0	Advanced	Save | More	
5	Cardiopulmonary Conditioning.mp.	10	Advanced	Display Results | More	
6	Coronary.mp.	520644	Advanced	Display Results | More	
7	CR.mp.	91755	Advanced	Display Results | More	
8	exCR.mp.	17	Advanced	Display Results | More	
9	Heart.mp. or exp Heart/	1394197	Advanced	Display Results | More	
10	1 or 2 or 3 or 4 or 5 or 6 or 7 or 8 or 9	2896038	Advanced	Display Results | More	
11	(Home* or home-based or house* or indoor or HB).mp. [mp=title, abstract, original title, name of substance word, subject heading word, floating sub-heading word, keyword heading word, organism supplementary concept word, protocol supplementary concept word, rare disease supplementary concept word, unique identifier, synonyms]	884252	Advanced	Display Results | More	
12	(House-care or in-house care).mp. [mp=title, abstract, original title, name of substance word, subject heading word, floating sub-heading word, keyword heading word, organism supplementary concept word, protocol supplementary concept word, rare disease supplementary concept word, unique identifier, synonyms]	30	Advanced	Display Results | More	
13	"Home-based exercise*".mp. [mp=title, abstract, original title, name of substance word, subject heading word, floating sub-heading word, keyword heading word, organism supplementary concept word, protocol supplementary concept word, rare disease supplementary concept word, unique identifier, synonyms]	1158	Advanced	Display Results | More	
14	(Home-base* and (intervention or programme or rehabilitation)).mp. [mp=title, abstract, original title, name of substance word, subject heading word, floating sub-heading word, keyword heading word, organism supplementary concept word, protocol supplementary concept word, rare disease supplementary concept word, unique identifier, synonyms]	5924	Advanced	Display Results | More	
15	Home Rehabilitation.mp.	421	Advanced	Display Results | More	
16	Home Physical Therapy.mp.	27	Advanced	Display Results | More	
17	exp Physical Therapy Modalities/ or exp Exercise Therapy/ or Therapeutic Exercise.mp.	161423	Advanced	Display Results | More	
18	"Fit Homes".mp. [mp=title, abstract, original title, name of substance word, subject heading word, floating sub-heading word, keyword heading word, organism supplementary concept word, protocol supplementary concept word, rare disease supplementary concept word, unique identifier, synonyms]	0	Advanced	Save | More	
19	11 or 12 or 13 or 14 or 15 or 16 or 17 or 18	1037142	Advanced	Display Results | More	
20	(Rural* or remote).mp. [mp=title, abstract, original title, name of substance word, subject heading word, floating sub-heading word, keyword heading word, organism supplementary concept word, protocol supplementary concept word, rare disease supplementary concept word, unique identifier, synonyms]	253055	Advanced	Display Results | More	
21	Rural health.mp. or exp Rural Health/ or exp Health Services Accessibility/	152257	Advanced	Display Results | More	
22	Rural areas.mp.	33034	Advanced	Display Results | More	
23	Rural population.mp. or exp Rural Population/	66630	Advanced	Display Results | More	
24	Rural Health Personnel.mp.	14	Advanced	Display Results | More	
25	Rural Health Centers.mp. or exp Rural Health Services/	13516	Advanced	Display Results | More	
26	Remote Consultation.mp. or exp Remote Consultation/	5583	Advanced	Display Results | More	
27	Remote healthcare.mp. or exp Rural Health Services/	13416	Advanced	Display Results | More	

28	(Healthcar* and (rural* or remote)).mp. [mp=title, abstract, original title, name of substance word, subject heading word, floating sub-heading word, keyword heading word, organism supplementary concept word, protocol supplementary concept word, rare disease supplementary concept word, unique identifier, synonyms]	12124	Advanced	Display Results | More
29	20 or 21 or 22 or 23 or 24 or 25 or 26 or 27 or 28	359730	Advanced	Display Results | More
30	Digital health.mp.	3187	Advanced	Display Results | More
31	Digital technology.mp. or exp Digital Technology/	1756	Advanced	Display Results | More
32	Telehealth.mp. or exp Telemedicine/	38039	Advanced	Display Results | More
33	(eHealth or mHealth).mp. [mp=title, abstract, original title, name of substance word, subject heading word, floating sub-heading word, keyword heading word, organism supplementary concept word, protocol supplementary concept word, rare disease supplementary concept word, unique identifier, synonyms]	9958	Advanced	Display Results | More
34	Telemedicine.mp. or exp Telemedicine/	41250	Advanced	Display Results | More
35	exp Technology/ or Technology.mp.	791122	Advanced	Display Results | More
36	Telerehabilitation.mp. or exp Telerehabilitation/	1330	Advanced	Display Results | More
37	Smartphone.mp. or exp Cell Phone/ or exp Smartphone/	24524	Advanced	Display Results | More
38	World Wide Web Applications.mp.	4	Advanced	Display Results | More
39	Mobile application.mp. or exp Mobile Applications/	9180	Advanced	Display Results | More
40	(App* or electronic application* or internet app*).mp. [mp=title, abstract, original title, name of substance word, subject heading word, floating sub-heading word, keyword heading word, organism supplementary concept word, protocol supplementary concept word, rare disease supplementary concept word, unique identifier, synonyms]	7123110	Advanced	Display Results | More
41	Internet.mp. or exp Internet Access/ or exp "Internet of Things"/ or exp Internet/ or exp Internet-Based Intervention/	119383	Advanced	Display Results | More
42	("World Wide Web Applications" or "World Wide Web" or "world wide web" or www).mp. [mp=title, abstract, original title, name of substance word, subject heading word, floating sub-heading word, keyword heading word, organism supplementary concept word, protocol supplementary concept word, rare disease supplementary concept word, unique identifier, synonyms]	4415	Advanced	Display Results | More
43	30 or 31 or 32 or 33 or 34 or 35 or 36 or 37 or 38 or 39 or 40 or 41 or 42	7682185	Advanced	Display Results | More
44	exp Monitoring, Ambulatory/ or Wearable Sensors.mp. or exp Biosensing Techniques/ or exp Wearable Electronic Devices/	104048	Advanced	Display Results | More
45	(wearable* or wearable technolog* or wear* fit* technolog*).mp. [mp=title, abstract, original title, name of substance word, subject heading word, floating sub-heading word, keyword heading word, organism supplementary concept word, protocol supplementary concept word, rare disease supplementary concept word, unique identifier, synonyms]	17122	Advanced	Display Results | More
46	Assistive Technology.mp. or exp Self-Help Devices/	13055	Advanced	Display Results | More
47	exp Self-Help Devices/ or Assistive Technology Services.mp.	12034	Advanced	Display Results | More
48	Fitness Trackers.mp. or exp Fitness Trackers/	927	Advanced	Display Results | More
49	(Fitness tracker* or Activity tracker* or fitness activity tracker* or tracker* or tracking).mp. [mp=title, abstract, original title, name of substance word, subject heading word, floating sub-heading word, keyword heading word, organism supplementary concept word, protocol supplementary concept word, rare disease supplementary concept word, unique identifier, synonyms]	96145	Advanced	Display Results | More
50	Monitoring, Physiologic.mp. or exp Monitoring, Physiologic/	181516	Advanced	Display Results | More
51	(activity monitor* or monitor* or monitoring or heart monitor).mp. [mp=title, abstract, original title, name of substance word, subject heading word, floating sub-heading word, keyword heading word, organism supplementary concept word, protocol supplementary concept word, rare disease supplementary concept word, unique identifier, synonyms]	1032538	Advanced	Display Results | More
52	exp Accelerometry/ or Accelerometer.mp.	18101	Advanced	Display Results | More
53	(pedometer* or Step count*).mp. [mp=title, abstract, original title, name of substance word, subject heading word, floating sub-heading word, keyword heading word, organism supplementary concept word, protocol supplementary concept word, rare disease supplementary concept word, unique identifier, synonyms]	4308	Advanced	Display Results | More
54	(Fitbit* or Smart watch* or Garmin or Apple watch).mp. [mp=title, abstract, original title, name of substance word, subject heading word, floating sub-heading word, keyword heading word, organism supplementary concept word, protocol supplementary concept word, rare disease supplementary concept word, unique identifier, synonyms]	1157	Advanced	Display Results | More
55	Wii.mp. [mp=title, abstract, original title, name of substance word, subject heading word, floating sub-heading word, keyword heading word, organism supplementary concept word, protocol supplementary concept word, rare disease supplementary concept word, unique identifier, synonyms]	917	Advanced	Display Results | More
56	44 or 45 or 46 or 47 or 48 or 49 or 50 or 51 or 52 or 53 or 54 or 55	1240773	Advanced	Display Results | More
57	10 and 19 and 29 and 43 and 56	488	Advanced	Display Results | More

PubMed Search Pattern

<u>Notes:</u>

- You'll see a "!" next to some of the searches. This can be if there's a syntax error in the search pattern. However, here it is to highlight that some of the search terms yielded no results;
- Searched the "Title/ Abstract" fields of the records, rather than all fields. In PubMed, searching all fields would both pull in many more records, as well as much of the extra being of diminishing relevance to the topic(s). This essentially is a choice of balance.

History and Search Details

Download Delete

Search	Actions	Details	Query	Results	Time
#8	...	! >	Search: **#1 AND #2 AND #3 AND #4 AND #5** Filters: **English, from 1990 - 2021**	43	09:23:15
#7	...	! >	Search: **#1 AND #2 AND #3 AND #4 AND #5** Filters: **English**	43	09:22:51
#6	...	! >	Search: **#1 AND #2 AND #3 AND #4 AND #5**	43	09:21:50
#5	...	! >	Search: **"wearable sensors" OR wearable* OR "wearable technolog*" OR "wearable fit* technolog*" OR "assistive technolog*" OR "assistive technology service*" OR "fitness tracker*" OR "activity tracker*" OR "fitness activity tracker*" OR tracker* OR tracking OR "physiologic* monitoring" OR "activity monitor*" OR monitor* OR "heart monitor" OR accelerometer OR pedomet* OR "step count*" OR fitbit* OR "smart watch*" OR Garmin OR "Apple watch" OR Wii**	1,222,901	09:20:51
#4	...	>	Search: **digital health OR "digital technolog*" OR telehealth OR eHealth OR mHealth OR telemedicine* OR technolog* OR telerehabilitation OR "smart phone*" "World Wide Web applications" [Title/Abstract] OR "mobile application*"[Title/Abstract] OR "cellular phone*"[Title/Abstract] OR app* OR "electronic application*" OR "internet app*" OR internet OR "internet connections"[Title/Abstract] OR "internet-based intervention"[Title/Abstract] OR "internet of things"[Title/Abstract] OR "World Wide Web application*" [Title/Abstract] OR "World Wide Web"[Title/Abstract] OR www**	218,422	09:17:18
#3	...	! >	Search: **(rural*[Title/Abstract] OR remote)[Title/Abstract] OR "rural health"[Title/Abstract] OR "rural area*"[Title/Abstract] OR "rural population*"[Title/Abstract] OR "rural health personnel" [Title/Abstract] OR "rural health cent*"[Title/Abstract] OR "rural health service*"[Title/Abstract] OR "remote consultation" [Title/Abstract] OR "remote healthcare"[Title/Abstract] OR "remote area*"[Title/Abstract] OR (healthcar*[Title/Abstract] AND (rural* [Title/Abstract] OR remote))[Title/Abstract]**	238,810	09:16:43
#2	...	! >	Search: **((home*[Title/Abstract] OR home-based[Title/Abstract] OR house*[Title/Abstract] OR indoor[Title/Abstract] OR HB)[Title/Abstract] AND (intervention[Title/Abstract] OR programme[Title/Abstract] OR rehabilitation[Title/Abstract] OR exercise*))[Title/Abstract] OR "house-care"[Title/Abstract] OR "in-house care"[Title/Abstract] OR "home physical therapy"[Title/Abstract] OR "therapeutic exercise*"[Title/Abstract] OR "Fit*Homes" [Title/Abstract]**	80,757	09:14:35
#1	...	! >	Search: **"cardiovascular system"[Title/Abstract] OR cardiovascular* OR cardiac OR "cardiac intervention*"[Title/Abstract] OR "cardiac rehabilitation"[Title/Abstract] OR "cardiac disease rehabilitation" [Title/Abstract] OR "cardiopulmonary condition*"[Title/Abstract] OR coronary OR CR OR exCR OR heart**	3,510,263	09:14:12

SportDiscuss Search Pattern

[Accessibility Information and Tips]

Print Search History

MY EBSCOhost

Thursday, June 10, 2021 9:42:24 AM

#	Query	Limiters/Expanders	Last Run Via	Results

S27	S4 AND S8 AND S14 AND S19 AND S26	Limiters - Language: English<br>Expanders - Apply equivalent subjects<br>Search modes - Boolean/Phrase	Interface - EBSCOhost Research Databases<br>Search Screen - Basic Search<br>Database - SPORTDiscus with Full Text	9
S26	S20 OR S21 OR S22 OR S23 OR S24 OR S25	Expanders - Apply equivalent subjects<br>Search modes - Boolean/Phrase	Interface - EBSCOhost Research Databases<br>Search Screen - Basic Search<br>Database - SPORTDiscus with Full Text	43,700
S25	Wii	Expanders - Apply equivalent subjects<br>Search modes - Boolean/Phrase	Interface - EBSCOhost Research Databases<br>Search Screen - Basic Search<br>Database - SPORTDiscus with Full Text	492
S24	fitbit*OR “smart watch*" OR Garmin OR “Apple watch”	Expanders - Apply equivalent subjects<br>Search modes - Boolean/Phrase	Interface - EBSCOhost Research Databases<br>Search Screen - Basic Search<br>Database - SPORTDiscus with Full Text	1,051
S23	"physiologic* monitoring" OR “activity monitor*” OR monitor* OR "heart monitor" OR accelerometer OR pedomet* OR “step count*”	Expanders - Apply equivalent subjects<br>Search modes - Boolean/Phrase	Interface - EBSCOhost Research Databases<br>Search Screen - Basic Search<br>Database - SPORTDiscus with Full Text	35,364
S22	"fitness tracker*" OR “activity tracker*” OR “fitness activity tracker*” OR tracker* OR tracking	Expanders - Apply equivalent subjects	Interface - EBSCOhost Research	6,123

		Search modes - Boolean/Phrase	Databases Search Screen - Basic Search Database - SPORTDiscus with Full Text	
S21	"assistive technolog*" OR "assistive technology service*"	Expanders - Apply equivalent subjects Search modes - Boolean/Phrase	Interface - EBSCOhost Research Databases Search Screen - Basic Search Database - SPORTDiscus with Full Text	644
S20	"wearable sensors" OR wearable* OR “wearable technolog*” OR “wearable fit* technolog*”	Expanders - Apply equivalent subjects Search modes - Boolean/Phrase	Interface - EBSCOhost Research Databases Search Screen - Basic Search Database - SPORTDiscus with Full Text	1,827
S19	S15 OR S16 OR S17 OR S18	Expanders - Apply equivalent subjects Search modes - Boolean/Phrase	Interface - EBSCOhost Research Databases Search Screen - Basic Search Database - SPORTDiscus with Full Text	389,226
S18	"World Wide Web applications" OR app* OR “electronic application*” OR “internet app*” OR internet OR "internet connections" OR "internet-based intervention" OR "internet of things" OR "World Wide Web application*" OR "World Wide Web" OR www	Expanders - Apply equivalent subjects Search modes - Boolean/Phrase	Interface - EBSCOhost Research Databases Search Screen - Basic Search Database - SPORTDiscus with Full Text	347,989
S17	“smart phone*” OR "mobile application*" OR "cellular phone*"	Expanders - Apply equivalent subjects Search modes - Boolean/Phrase	Interface - EBSCOhost Research Databases Search Screen - Basic Search	867

			Database - SPORTDiscus with Full Text	
S16	telehealth OR eHealth OR mHealth OR telemedicine*	Expanders - Apply equivalent subjects Search modes - Boolean/Phrase	Interface - EBSCOhost Research Databases Search Screen - Basic Search Database - SPORTDiscus with Full Text	1,278
S15	digital health OR “digital technolog*” OR technolog*	Expanders - Apply equivalent subjects Search modes - Boolean/Phrase	Interface - EBSCOhost Research Databases Search Screen - Basic Search Database - SPORTDiscus with Full Text	59,251
S14	S9 OR S10 OR S11 OR S12 OR S13	Expanders - Apply equivalent subjects Search modes - Boolean/Phrase	Interface - EBSCOhost Research Databases Search Screen - Basic Search Database - SPORTDiscus with Full Text	10,660
S13	"remote consultation" OR "remote healthcare"	Expanders - Apply equivalent subjects Search modes - Boolean/Phrase	Interface - EBSCOhost Research Databases Search Screen - Basic Search Database - SPORTDiscus with Full Text	4
S12	“rural health personnel” OR "rural health cent*" OR "rural health service*"	Expanders - Apply equivalent subjects Search modes - Boolean/Phrase	Interface - EBSCOhost Research Databases Search Screen - Basic Search Database - SPORTDiscus with Full Text	102

S11	(healthcar* AND (rural* OR remote)) OR “rural health”	Expanders - Apply equivalent subjects Search modes - Boolean/Phrase	Interface - EBSCOhost Research Databases Search Screen - Basic Search Database - SPORTDiscus with Full Text	810
S10	“rural area*" OR "remote area*" OR “rural population*”	Expanders - Apply equivalent subjects Search modes - Boolean/Phrase	Interface - EBSCOhost Research Databases Search Screen - Basic Search Database - SPORTDiscus with Full Text	2,409
S9	(rural* OR remote)	Expanders - Apply equivalent subjects Search modes - Boolean/Phrase	Interface - EBSCOhost Research Databases Search Screen - Basic Search Database - SPORTDiscus with Full Text	10,660
S8	S5 OR S6 OR S7	Expanders - Apply equivalent subjects Search modes - Boolean/Phrase	Interface - EBSCOhost Research Databases Search Screen - Basic Search Database - SPORTDiscus with Full Text	19,496
S7	"Fit*Homes"	Expanders - Apply equivalent subjects Search modes - Boolean/Phrase	Interface - EBSCOhost Research Databases Search Screen - Basic Search Database - SPORTDiscus with Full Text	0
S6	“house-care” OR “in-house care” OR "home physical therapy" OR "therapeutic exercise*"	Expanders - Apply equivalent subjects	Interface - EBSCOhost Research	864

		Search modes - Boolean/Phrase	Databases Search Screen - Basic Search Database - SPORTDiscus with Full Text	
S5	((home* OR home-based OR house* OR indoor OR HB) AND (intervention OR programme OR rehabilitation or exercise*))	Expanders - Apply equivalent subjects Search modes - Boolean/Phrase	Interface - EBSCOhost Research Databases Search Screen - Basic Search Database - SPORTDiscus with Full Text	18,699
S4	S1 OR S2 OR S3	Expanders - Apply equivalent subjects Search modes - Boolean/Phrase	Interface - EBSCOhost Research Databases Search Screen - Basic Search Database - SPORTDiscus with Full Text	101,239
S3	"cardiopulmonary condition*" OR coronary OR Heart	Expanders - Apply equivalent subjects Search modes - Boolean/Phrase	Interface - EBSCOhost Research Databases Search Screen - Basic Search Database - SPORTDiscus with Full Text	68,072
S2	"cardiac intervention*" OR "cardiac rehabilitation" OR "cardiac disease rehabilitation" OR CR OR exCR	Expanders - Apply equivalent subjects Search modes - Boolean/Phrase	Interface - EBSCOhost Research Databases Search Screen - Basic Search Database - SPORTDiscus with Full Text	3,113
S1	"cardiovascular system" OR cardiovascular* OR cardiac	Expanders - Apply equivalent subjects Search modes - Boolean/Phrase	Interface - EBSCOhost Research Databases Search Screen - Basic Search	58,979

			Database - SPORTDiscus with Full Text	

Web of Science search pattern

TOPIC: ("cardiovascular system" OR cardiovascular* OR cardiac OR "cardiac intervention*" OR "cardiac rehabilitation" OR "cardiac disease rehabilitation" OR "cardiopulmonary condition*" OR coronary OR CR OR exCR OR heart)
AND
TOPIC: (((home* OR home-based OR house* OR indoor OR HB) AND (intervention OR programme OR rehabilitation or exercise*)) OR "house-care" OR "in-house care" OR "home physical therapy" OR "therapeutic exercise*" OR "Fit*Homes")
AND
TOPIC: ((rural* OR remote) OR "rural health" OR "rural area*" OR "rural population*" OR "rural health personnel" OR "rural health cent*" OR "rural health service*" OR "remote consultation" OR "remote healthcare" OR "remote area*" OR (healthcar* AND (rural* OR remote)))
AND
TOPIC: (digital health OR "digital technolog*" OR telehealth OR eHealth OR mHealth OR telemedicine* OR technolog* OR telerehabilitation OR "smart phone*" OR "World Wide Web applications" OR "mobile application*" OR "cellular phone*" OR app* OR "electronic application*" OR "internet app*" OR internet OR "internet connections" OR "internet-based intervention" OR "internet of things" OR "World Wide Web application*" OR "World Wide Web" OR www)
AND
TOPIC: ("wearable sensors" OR wearable* OR "wearable technolog*" OR "wearable fit* technolog*" OR "assistive technolog*" OR "assistive technology service*" OR "fitness tracker*" OR "activity tracker*" OR "fitness activity tracker*" OR tracker* OR tracking OR "physiologic* monitoring" OR "activity monitor*" OR monitor* OR "heart monitor" OR accelerometer OR pedomet* OR "step count*" OR fitbit*OR "smart watch*" OR Garmin OR "Apple watch" OR Wii)

Refined by: LANGUAGES: (ENGLISH)
Timespan: 1990 - Current

Clusters for Scopus.

1. Cardiac rehabilitation
2. Home-based exercise
3. Remote and rural
4. Technology
5. [Wearable technology]

Cardiac rehabilitation
"cardiovascular system" OR cardiovascular* OR cardiac OR "cardiac intervention*" OR "cardiac rehabilitation" OR "cardiac disease rehabilitation" OR "cardiopulmonary condition*" OR coronary OR CR OR exCR OR heart

Home-based exercise
((home* OR home-based OR house* OR indoor OR HB) AND (intervention OR programme OR rehabilitation or exercise*)) OR "house-care" OR "in-house care" OR "home physical therapy" OR "therapeutic exercise*" OR "Fit*Homes"

Rural and Remote/ rurality:
(rural* OR remote) OR "rural health" OR "rural area*" OR "rural population*" OR "rural health personnel" OR "rural health cent*" OR "rural health service*" OR "remote consultation" OR "remote healthcare" OR "remote area*" OR (healthcar* AND (rural* OR remote))

Technology
digital health OR "digital technolog*" OR telehealth OR eHealth OR mHealth OR telemedicine* OR technolog* OR telerehabilitation OR "smart phone*" "World Wide Web applications" OR "mobile application*" OR "cellular phone*" OR app* OR "electronic application*" OR "internet app*" OR internet OR "internet connections" OR "internet-based intervention" OR "internet of things" OR "World Wide Web application*" OR "World Wide Web" OR www

[Wearable technology]
"wearable sensors" OR wearable* OR "wearable technolog*" OR "wearable fit* technolog*" OR "assistive technolog*" OR "assistive technology service*" OR "fitness tracker*" OR "activity tracker*" OR "fitness activity tracker*" OR tracker* OR tracking OR "physiologic* monitoring" OR "activity monitor*" OR monitor* OR "heart monitor" OR accelerometer OR pedomet* OR "step count*" OR fitbit* OR "smart watch*" OR Garmin OR "Apple watch" OR Wii
