# Supplementary material for: Does Connected Health Technology Improve Health-Related Outcomes in Rural Cardiac Populations? Systematic Review Narrative Synthesis
Source: Int J Environ Res Public Health. 2022 Feb 17;19(4):2302. doi: 10.3390/ijerph19042302 (PMC8871734; doi:10.3390/ijerph19042302)
Supplement: Supplementary file 1 [file ijerph-19-02302-s001.zip › Supplementary material S2.pdf]

Supplementary Material 2 – Search findings

'Home monitoring with IT-supported specialist management versus home monitoring alone in patients with heart failure: Design and baseline results of the SUPPORT-HF 2 randomized trial'. (2019) *American Heart Journal* **208**, 55-64

'Interstage Outcomes in Infants With Single Ventricle Heart Disease Comparing Home Monitoring Technology to Three-Ring Binder Documentation: a Randomized Crossover Study'. (2018) *World journal for pediatric and congenital hearth surgery* **9** (3), 305-314

'Home monitoring cuts cardiac readmissions'. (2011) *Hospital Case Management* **19** (5), 76-77

'Oral Presentation'. (2011) *Pacing & Clinical Electrophysiology* **34** (11), 1307-1361

'Poster Presentations'. (2011) *Pacing & Clinical Electrophysiology* **34** (11), 1362-1451

'Poster Session 1-3: Diagnostic Tools and Risk Stratification Monday, April 3, 2006, 2:00–3:30pm'. (2006) *Pacing & Clinical Electrophysiology* **29**, S50-S59

'Medical equipment. Helping hearts.'. (2004) *Health Service Journal* **114** (5924), 37

'CHF managers make the case for home-monitoring technology.'. (2002) *Disease management advisor* **8** (10), 156-158

'Interactive video program cuts home care costs while slashing readmission rates.'. (1998) *Healthcare demand & disease management* **4** (3), 46-48

'Hello? It's rehab calling: technology makes rehab by phone safe option'. (1995) *Case Management Advisor* **6** (4), 49-47

Abhinav Bassi, B., John, O., Praveen, D. & Pallab, K. 'Current Status and Future Directions of mHealth Interventions for Health System Strengthening in India: Systematic Review'.

Abril, E.P. (2016) 'Tracking myself: Assessing the contribution of mobile technologies for self-trackers of weight, diet, or exercise'. *Journal of health communication* **21** (6), 638-646

Abtahi, F. (2016) 'Towards heart rate variability tools in P-Health'.

Aceto, G., Persico, V. & Pescapé, A. (2018) 'The role of Information and Communication Technologies in healthcare: taxonomies, perspectives, and challenges'. *Journal of Network and Computer Applications* **107**, 125-154

Acheampong, F. & Vimarlund, V. (2015) 'Business models for telemedicine services: a literature review'. *Health Systems* **4** (3), 189-203

ACTRN12609000082202, (2009) 'A research study to evaluate the "current management" of patients with chronic heart failure with the "usual care" plus "additional" remote monitoring by study doctors and a research nurse'. *http://www.who.int/trialsearch/Trial2.aspx?TrialID=ACTRN12609000082202*

ACTRN12610000812099, (2010) 'Getting better at chronic care in North Queensland: a cluster randomized trial of patient-centred care delivered by Indigenous health professionals to Indigenous clients'. *http://www.who.int/trialsearch/Trial2.aspx?TrialID=ACTRN12610000812099*

ACTRN12613000921785, (2013) 'The Which Heart failure Intervention is most Cost-effective in reducing Hospital stay (WHICH? II) trial'. http://www.who.int/trialsearch/Trial2.aspx?TrialID=ACTRN12613000921785

ACTRN12617001408370, (2017) 'Exercise for diagnosis and treatment of heart dysfunction among breast cancer patients'. http://www.who.int/trialsearch/Trial2.aspx?TrialID=ACTRN12617001408370

ACTRN12618001359224, (2018) 'Remote Monitoring impact on cardiac arrhythmia detection in Adults with Implantable Loop Recorders'. http://www.who.int/trialsearch/Trial2.aspx?TrialID=ACTRN12618001359224

ACTRN12619000352101, (2019) 'Salt ALTernatives Study (SALTS): a smartphone app and dietary alternative salt to lower blood pressure for adults with high blood pressure'. http://www.who.int/trialsearch/Trial2.aspx?TrialID=ACTRN12619000352101

ACTRN12619001451190, (2019) 'Intensive Follow-up via Remote Monitoring of Implantable Cardioverter Defibrillators in people with severe left ventricular dysfunction'. http://www.who.int/trialsearch/Trial2.aspx?TrialID=ACTRN12619001451190

ACTRN12620001049965, (2020) 'Home-based blood pressure monitoring of Hypertensive Diseases in Pregnancy - A randomised controlled trial (REMOTE CONTROL trial)'. http://www.who.int/trialsearch/Trial2.aspx?TrialID=ACTRN12620001049965

ACTRN12620001176954, (2020) 'Code STORM: sTandard care Or a Rapid early invasive Management approach to patients with life threatening heart rhythm disorders'. http://www.who.int/trialsearch/Trial2.aspx?TrialID=ACTRN12620001176954

Adams, Z.W., McClure, E.A., Gray, K.M., Danielson, C.K., Treiber, F.A. & Ruggiero, K.J. (2017) 'Mobile devices for the remote acquisition of physiological and behavioral biomarkers in psychiatric clinical research'. *Journal of psychiatric research* **85**, 1-14

Addotey-Delove, M., Scott, R. & Mars, M. (2018) '001 MHEALTH ADOPTION FACTORS FOR PATIENTS IN THE DEVELOPING WORLD. AN INSTRUMENT DEVELOPMENT'. *Российский кардиологический журнал* **23** (10)

Adeola, O. & Evans, O. (2018) 'Digital health: ICT and health in Africa'. *Aktual'ni Problemy Ekonomiky= Actual Problems in Economics* (208), 66-83

Adepoju, I.O., Albersen, B.J.A., De Brouwere, V., van Roosmalen, J. & Zweekhorst, M. (2017) 'mHealth for clinical decision-making in sub-Saharan Africa: a scoping review'. *JMIR mHealth and uHealth* **5** (3), e38

Adlakha, D. (2017) 'Quantifying the modern city: emerging technologies and big data for active living research'. *Frontiers in public health* **5**, 105

Aggarwal, M., Ornish, D., Josephson, R., Brown, T.M., Ostfeld, R.J., Gordon, N., Madan, S., Allen, K., Khetan, A. & Mahmoud, A. (2021) 'Closing Gaps in Lifestyle Adherence for Secondary Prevention of Coronary Heart Disease'. *The American Journal of Cardiology*

Aggarwal, N., Ahmed, M., Basu, S., Curtin, J.J., Evans, B.J., Matheny, M.E., Nundy, S., Sendak, M.P., Shachar, C. & Shah, R.U. (2020) 'Advancing Artificial Intelligence in Health Settings Outside the Hospital and Clinic'. *NAM Perspectives*

Aguirre Gallego, E., Led Ramos, S., López Iturri, P., Azpilicueta Fernández de las Heras, Leyre, Serrano Arriezu, L.J. & Falcone Lanas, F.J. (2016) 'Implementation of context aware e-health environments based on social sensor networks'. *Sensors, 2016, 16 (3), 310*

Ahmed, F.Z., Crosbie, C., Kahn, M. & Motwani, M. (2020) *Protecting the most vulnerable during COVID-19 and beyond: a case report on the remote management of heart failure patients with cardiac implantable electronic devices*

Ahrentzen, S. & Tural, E. (2015) 'The role of building design and interiors in ageing actively at home'. *Building Research & Information* **43** (5), 582-601

Ainsworth, M.C. (2019) *Examining the Role of Perceived Benefits and Barriers in Physical Activity Behavior for Cancer Prevention and Control*

Ajarananda, V. (2018) 'A Novel Wearable Heart Function Analyzer For Screening and Monitoring In Remote and Under Resourced Setting.'. *Global Heart.Conference: World Congress of Cardiology & Cardiovascular Health 2018.Dubai United Arab Emirates* **13** (4), 410

Alam, M.Z., Hoque, M.R., Hu, W. & Barua, Z. (2020) 'Factors influencing the adoption of mHealth services in a developing country: A patient-centric study'. *International Journal of Information Management* **50**, 128-143

Alamäki, A., Nevala, E., Barton, J., Condell, J., Karla, M.E., Nordström, A., Tedesco, S., Kelly, D. & David, H. (2019) 'Wearable technology supported home rehabilitation services in rural areas: emphasis on monitoring structures and activities of functional capacity. Handbook'. *Wearable Technology Supported Home Rehabilitation Services in Rural Areas:–Emphasis on Monitoring Structures and Activities of Functional Capacity Handbook.* , 1-56

Alamäki, A., Nevala, E., Jalovaara, J., Barton, J., Condell, J., Muñoz Esquivel, K., Nordström, A., Kelly, D., Heaney, D. & Gillespie, J. (2021) 'Guidelines on the use of wearable sensor systems in-home rehabilitation combined with remote connections-Flowchart and Practice guideline: Handbook II'.

Al-Badi, A. & Khan, A. (2020) 'Determining the constituents for the advancement of healthcare wearable technologies in Oman'. *International Journal of Electronic Healthcare* **11** (2), 136-156

Albahri, A.S., Alwan, J.K., Taha, Z.K., Ismail, S.F., Hamid, R.A., Zaidan, A., Albahri, O.S., Zaidan, B., Alamoodi, A. & Alsalem, M. (2021) 'IoT-based telemedicine for disease prevention and health promotion: State-of-the-Art'. *Journal of Network and Computer Applications* **173**, 102873

Albahri, A.S., Zaidan, A., Albahri, O.S., Zaidan, B. & Alsalem, M. (2018) 'Real-time fault-tolerant mHealth system: Comprehensive review of healthcare services, opens issues, challenges and methodological aspects'. *Journal of medical systems* **42** (8), 1-56

Albahri, O.S., Albahri, A.S., Mohammed, K., Zaidan, A., Zaidan, B., Hashim, M. & Salman, O.H. (2018) 'Systematic review of real-time remote health monitoring system in triage and priority-based sensor technology: Taxonomy, open challenges, motivation and recommendations'. *Journal of medical systems* **42** (5), 1-27

Albahri, O.S., Albahri, A.S., Zaidan, A., Zaidan, B., Alsalem, M., Mohsin, A., Mohammed, K., Alamoodi, A., Nidhal, S. & Enaizan, O. (2019) 'Fault-tolerant mHealth framework in the context of IoT-based real-time wearable health data sensors'. *IEEE Access* **7**, 50052-50080

Albergoni, A., Hettinga, F.J., Stut, W. & Sartor, F. (2020) 'Factors Influencing Walking and Exercise Adherence in Healthy Older Adults Using Monitoring and Interfacing Technology: Preliminary Evidence'. *International Journal of Environmental Research and Public Health* **17** (17), 6142

Alelyani, T., Shaikh, A., Sulaiman, A.A., Asiri, Y., Alshahrani, H. & Almakdi, S. (2021) 'Research Challenges and Opportunities Towards a Holistic View of Telemedicine Systems: A Systematic Review'. *Enhanced Telemedicine and e-Health: Advanced IoT Enabled Soft Computing Framework* , 3-26

Alfano, C.M., Leach, C.R., Smith, T.G., Miller, K.D., Alcaraz, K.I., Cannady, R.S., Wender, R.C. & Brawley, O.W. (2019) 'Equitably improving outcomes for cancer survivors and supporting caregivers: a blueprint for care delivery, research, education, and policy'. *CA: a cancer journal for clinicians* **69** (1), 35-49

Alghamdi, A.S., Alghamdi, K.A., Jenkins, R.O., Alghamdi, M.N. & Haris, P.I. (2020) 'Impact of Ramadan on physical activity and sleeping patterns in individuals with type 2 diabetes: the first study using Fitbit device'. *Diabetes Therapy* **11** (6), 1331-1346

Ali, H., Villaneouva, B. & Yaqub, R. (2019) 'Design and Implementation of a Low Cost Wireless Ambulatory ECG Monitoring System for Deployment in Rural Communities'.

Allersma, T., Farquhar, C. & Cantineau, A. (2013) 'Natural cycle in vitro fertilisation (IVF) for subfertile couples'. *Cochrane Database of Systematic Reviews* (8)

Allida, S., Du, H., Xu, X., Prichard, R., Chang, S., Hickman, L.D., Davidson, P.M. & Inglis, S.C. (2020) 'mHealth education interventions in heart failure'. *Cochrane Database of Systematic Reviews* (7)

Almarcha, M., Balagué, N. & Torrents, C. (2021) 'Healthy Teleworking: Towards Personalized Exercise Recommendations'. *Sustainability* **13** (6), 3192

Al-Naji, A., Gibson, K. & Chahl, J. (2017) 'Remote sensing of physiological signs using a machine vision system.'. *Journal of medical engineering & technology* **41** (5), 396-405

Al-Qurishi, M., Al-Rakhami, M., Al-Qershi, F., Hassan, M.M., Alamri, A., Khan, H.U. & Xiang, Y. (2015) 'A framework for cloud-based healthcare services to monitor noncommunicable diseases patient'. *International journal of distributed Sensor Networks* **11** (3), 985629

Alsswey, A., Al-Samarraie, H. & Bervell, B. (2021) 'mHealth technology utilization in the Arab world: a systematic review of systems, usage, and challenges'. *Health and Technology* , 1-13

Al-Turjman, F., Nawaz, M.H. & Ulusar, U.D. (2020) 'Intelligence in the Internet of Medical Things era: A systematic review of current and future trends'. *Computer Communications* **150**, 644-660

Alves, T.M.R., Deroco, P.B., Junior, D.W., Vidotto, L.H.B. & Kubota, L.T. (2021) 'Wireless Wearable Electrochemical Sensors: A Review'. *Braz.J.Anal.Chem* , 8

Alzamanan, M.Z., Lim, K., Ismail, M.A. & Ghani, N.A. (2021) 'Self-Management Apps for People With Epilepsy: Systematic Analysis'. *JMIR mHealth and uHealth* **9** (5), e22489

Amadi-Obi, A., Gilligan, P., Owens, N. & O'Donnell, C. (2014) 'Telemedicine in pre-hospital care: a review of telemedicine applications in the pre-hospital environment'. *International journal of emergency medicine* **7** (1), 1-11

Amagasa, S., Fukushima, N., Kikuchi, H., Takamiya, T., Odagiri, Y., Oka, K. & Inoue, S. (2018) 'Drivers are more physically active than non-drivers in older adults'. *International journal of environmental research and public health* **15** (6), 1094

Amara, W., Montagnier, C., Cheggour, S., Boursier, M., Gully, C., Barnay, C., Georger, F., Deplagne, A., Fromentin, S. & Mlotek, M. (2015) 'Strategy of early detection and active management of

supraventricular arrhythmia with remote monitoring: the randomized, multicenter SETAM trial'. *Archives of cardiovascular diseases supplements.* **7** (1), 69

Amara, W., Montagnier, C., Cheggour, S., Boursier, M., Gully, C., Barnay, C., Georger, F., Deplagne, A., Fromentin, S., Mlotek, M., Lazarus, A., Taieb, J. & SETAM Investigators (2017) 'Early Detection and Treatment of Atrial Arrhythmias Alleviates the Arrhythmic Burden in Paced Patients: The SETAM Study.'. *Pacing & Clinical Electrophysiology* **40** (5), 527-536

Amato, S., Di Giovanni, C., Politi, M. & De Salazar, V. (2019) '[The telemedicine as tool of management on chronic disease: the experience of ASL ROMA 3 (Italian Local Authority). Impact, outcomes and results]'. *Igiene e Sanita Pubblica* **75** (3), 231-244

Ames, H., Glenton, C., Lewin, S., Tamrat, T., Akama, E. & Leon, N. (2019) 'Clients’ perceptions and experiences of targeted digital communication accessible via mobile devices for reproductive, maternal, newborn, child, and adolescent health: a qualitative evidence synthesis'. *Cochrane Database of Systematic Reviews* (10)

Amir, O., Ben-Gal, T., Weinstein, J.M., Schliamser, J., Burkhoff, D., Abbo, A. & Abraham, W.T. (2017) 'Evaluation of remote dielectric sensing (ReDS) technology-guided therapy for decreasing heart failure re-hospitalizations.'. *International journal of cardiology* **240**, 279-284

Amir, O., Rappaport, D., Zafrir, B. & Abraham, W.T. (2013) 'A novel approach to monitoring pulmonary congestion in heart failure: initial animal and clinical experiences using remote dielectric sensing technology.'. *Congestive Heart Failure* **19** (3), 149-155

Amorim, V.J., Oliveira, R.A. & da Silva, M.J. (2020) 'Recent Trends in Wearable Computing Research: A Systematic Review'. *arXiv preprint arXiv:2011.13801*

Andersen, T.O., Nielsen, K.D., Moll, J. & Svendsen, J.H. (2019) 'Unpacking telemonitoring work: Workload and telephone calls to patients in implanted cardiac device care'. *International journal of medical informatics* **129**, 381-387

Anderson, L.M., Adeney, K.L., Shinn, C., Safranek, S., Buckner-Brown, J. & Krause, L.K. (2015) 'Community coalition-driven interventions to reduce health disparities among racial and ethnic minority populations'. *Cochrane Database of Systematic Reviews* (6)

Ando, K., Koyama, J., Abe, Y., Sato, T., Shoda, M., Soga, Y., Nobuyoshi, M., Honda, T., Nakao, K., Terata, K., Kadowaki, K., Maeda, A., Ogawa, S., Manaka, T., Hagiwara, N. & Doi, K. (2011) 'Feasibility evaluation of a remote monitoring system for implantable cardiac devices in Japan.'. *International Heart Journal* **52** (1), 39-43

Andres, E., Talha, S., Zulfiqar, A., Hajjam, M., Erve, S., Hajjam, J., Geny, B. & El Hassani, A.H. (2018}) 'Current Research and New Perspectives of Telemedicine in Chronic Heart Failure: Narrative Review and Points of Interest for the Clinician'. *JOURNAL OF CLINICAL MEDICINE}* **7}** (12})

Andria, G., Lanzolla, A.M.L., Cavallo, G., Russo, G., Marinosci, F., Incalzi, R.A. & Benvenuto, M. (2015) 'A novel approach for design and testing digital m-health applications'. In Anonymous (eds.) *2015 IEEE International Symposium on Medical Measurements and Applications (MeMeA) Proceedings,* Held IEEE, 440-444

Andrikopoulou, E. & Scott, P. 'What are the Essential Design Features of PHR to Improve Medication Adherence: Protocol for a Systematic Review.'.

ANEJA, J. & ARORA, S. (2021) 'Telemedicine and ethics: opportunities in India'.

Ansary, A.M., Martinez, J.N. & Scott, J.D. (2019) 'The virtual physical exam in the 21st century'. *Journal of telemedicine and telecare* , 1357633X19878330

Ansbro, E., Homan, T., Prieto Merino, D., Jobanputra, K., Qasem, J., Muhammad, S., Fardous, T. & Perel, P. (2021) 'Clinical outcomes in a primary-level non-communicable disease programme for Syrian refugees and the host population in Jordan: A cohort analysis using routine data.'. *PLoS Medicine / Public Library of Science* **18** (1), e1003279

Antman, E.M., Benjamin, E.J., Harrington, R.A., Houser, S.R., Peterson, E.D., Bauman, M.A., Brown, N., Bufalino, V., Califf, R.M. & Creager, M.A. (2015) 'Acquisition, analysis, and sharing of data in 2015 and beyond: a survey of the landscape: a conference report from the American Heart Association Data Summit 2015'. *Journal of the American Heart Association* **4** (11), e002810

Antón Sáez, D. (2015) 'Kires: a data-centric telerehabilitation system based on kinect'.

Anwer, S., Li, H., Antwi-Afari, M.F., Umer, W. & Wong, A.Y.L. (2021) 'Evaluation of physiological metrics as real-time measurement of physical fatigue in construction workers: State-of-the-art review'. *Journal of Construction Engineering and Management* **147** (5), 03121001

Appiah, K., Sam-Epelle, I. & Osabutey, E.L. (2019) '19 Technology and Health Services Marketing in Africa'. *Health Service Marketing Management in Africa*

Aranki, D. (2017) *Towards Predictive Medicine—On Remote Monitoring, Privacy and Scientific Bias*

Archer, N., Keshavjee, K., Demers, C. & Lee, R. (2014) 'Online self-management interventions for chronically ill patients: cognitive impairment and technology issues'. *International journal of medical informatics* **83** (4), 264-272

Arditi, C., Rège‐Walther, M., Durieux, P. & Burnand, B. (2017) 'Computer‐generated reminders delivered on paper to healthcare professionals: effects on professional practice and healthcare outcomes'. *Cochrane Database of Systematic Reviews* (7)

Arena, R., Ozemek, C., Laddu, D., Campbell, T., Rouleau, C.R., Standley, R., Bond, S., Abril, E.P., Hills, A.P. & Lavie, C.J. (2018) 'Applying precision medicine to healthy living for the prevention and treatment of cardiovascular disease'. *Current problems in cardiology* **43** (12), 448-483

Ariani, A., Koesoema, A.P. & Soegijoko, S. (2017) 'Innovative healthcare applications of ICT for developing countries'. In *Innovative Healthcare Systems for the 21st Century* Springer, 15-70

Arif, M. & Parveen, S. (2021) 'Carcinogenic effects of indoor black carbon and particulate matters ($PM_{2.5}$ and PM_{10}) in rural households of India.'. *Environmental Science & Pollution Research* **28** (2), 2082-2096

Arigo, D., Jake-Schoffman, D.E., Wolin, K., Beckjord, E., Hekler, E.B. & Pagoto, S.L. (2019) 'The history and future of digital health in the field of behavioral medicine'. *Journal of Behavioral Medicine* **42** (1), 67-83

Arikpo, D., Edet, E.S., Chibuzor, M.T., Odey, F. & Caldwell, D.M. (2018) 'Educational interventions for improving primary caregiver complementary feeding practices for children aged 24 months and under'. *Cochrane Database of Systematic Reviews* (5)

Arku, R.E., Birch, A., Shupler, M., Yusuf, S., Hystad, P. & Brauer, M. (2018) 'Characterizing exposure to household air pollution within the Prospective Urban Rural Epidemiology (PURE) study.'. *Environment international* **114**, 307-317

Artico, J., Zecchin, M., Zorzin Fantasia, A., Skerl, G., Ortis, B., Franco, S., Albani, S., Barbati, G., Cristallini, J., Cannata', A. & Sinagra, G. (2019) 'Long-term patient satisfaction with implanted device remote monitoring: a comparison among different systems.'. *Journal of Cardiovascular Medicine* **20** (8), 542-550

Arzbaecher, R., Hampton, D.R., Burke, M.C., Garrett, M.C., Arzbaecher, R., Hampton, D.R., Burke, M.C. & Garrett, M.C. (2010) 'Subcutaneous electrocardiogram monitors and their field of view'. *Journal of electrocardiology* **43** (6), 601-605

Asayama, K., Thijs, L., Brguljan-Hitij, J., Niiranen, T.J., Hozawa, A., Boggia, J., Aparicio, L.S., Hara, A., Johansson, J.K., Ohkubo, T., Tzourio, C., Stergiou, G.S., Sandoya, E., Tsuji, I., Jula, A.M., Imai, Y., Staessen, J.A. & International Database of Home Blood Pressure in Relation to Cardiovascular Outcome (IDHOCO) investigators (2014) 'Risk stratification by self-measured home blood pressure across categories of conventional blood pressure: a participant-level meta-analysis.'. *PLoS Medicine / Public Library of Science* **11** (1), e1001591

Ashdown-Franks, G. (2018) *From Intervention to Evaluation: Assessing the Effectiveness, Acceptability and Feasibility of a Physical Activity Intervention for Individuals with Serious Mental Illness*

Association of Diabetes Care and Education Specialists & Kolb, L. (2021) 'An Effective Model of Diabetes Care and Education: The ADCES7 Self-Care Behaviors™'. *The Science of Diabetes Self-Management and Care* **47** (1), 30-53

Atherton, H., Sawmynaden, P., Sheikh, A., Majeed, A. & Car, J. (2012) 'Email for clinical communication between patients/caregivers and healthcare professionals'. *Cochrane Database of Systematic Reviews* (11)

Atkinson, K. & Mabey, D. (2019) *Revolutionizing Tropical Medicine: Point-of-Care Tests, New Imaging Technologies and Digital Health*John Wiley & Sons

Attridge, M., Creamer, J., Ramsden, M., Cannings-John, R. & Hawthorne, K. (2014) 'Culturally appropriate health education for people in ethnic minority groups with type 2 diabetes mellitus'. *Cochrane Database of Systematic Reviews* (9)

Aturinde, A. (2020) *GIS and Health: Enhancing Disease Surveillance and Intervention through Spatial Epidemiology*

Augustyniak, P. (2011) 'Wearable wireless heart rate monitor for continuous long-term variability studies.'. *Journal of electrocardiology* **44** (2), 195-200

Austin, J., Dodge, H.H., Riley, T., Jacobs, P.G., Thielke, S. & Kaye, J. (2016) 'A smart-home system to unobtrusively and continuously assess loneliness in older adults'. *IEEE journal of translational engineering in health and medicine* **4**, 1-11

Avau, B., Borra, V., Vanhove, A.C., Vandekerckhove, P., De Paepe, P. & De Buck, E. (2018) 'First aid interventions by laypeople for acute oral poisoning'. *Cochrane Database of Systematic Reviews* (12)

Babu A.V. & Gupta, B. (2021) 'Heart rate monitoring of anesthesiology residents during the airway management of COVID-19 suspect patients: An observational study.'. *Indian Journal of Critical Care Medicine.Conference: 27th Annual Conference of Indian Society of Critical Care Medicine, Criticare - ISCCM 2021.Virtual* **25** (SUPPL 1), S50

Badawy, S.M., Cronin, R.M., Hankins, J., Crosby, L., DeBaun, M., Thompson, A.A. & Shah, N. (2018) 'Patient-centered eHealth interventions for children, adolescents, and adults with sickle cell disease: systematic review'. *Journal of medical Internet research* **20** (7), e10940

Badawy, S.M., Morrone, K., Thompson, A. & Palermo, T.M. (2019) 'Computer and mobile technology interventions to promote medication adherence and disease management in people with thalassemia'. *Cochrane Database of Systematic Reviews* (6)

Bahar-Fuchs, A., Martyr, A., Goh, A., Sabates, J. & Clare, L. (2019) 'Cognitive training for people with mild to moderate dementia'. *Cochrane Database of Systematic Reviews* (3)

Bajwah, S., Oluyase, A.O., Yi, D., Gao, W., Evans, C.J., Grande, G., Todd, C., Costantini, M., Murtagh, F.E. & Higginson, I.J. (2020) 'The effectiveness and cost-effectiveness of hospital-based specialist palliative care for adults with advanced illness and their caregivers'. *Cochrane Database of Systematic Reviews* (9)

Baker, P., Francis, D.P., Soares, J., Weightman, A.L. & Foster, C. (2015) 'Community wide interventions for increasing physical activity'. *Cochrane Database of Systematic Reviews* (1)

Bakhshi, S., Li, X., Semenov, N., Apodaca-Madrid, J., Mahoor, M.H., Newman, K.E., Long, C.S. & Neuman, C. (2011) 'Congestive Heart Failure home monitoring pilot study in urban Denver.'. *Annual International Conference Of The IEEE Engineering In Medicine And Biology Society* **2011**, 3150-3153

Bakken, S., Marden, S., Arteaga, S.S., Grossman, L., Keselman, A., Le, P., Creber, R.M., Powell-Wiley, T.M., Schnall, R. & Tabor, D. (2019) 'Behavioral interventions using consumer information technology as tools to advance health equity'. *American Journal of Public Health* **109** (S1), S79-S85

Bale, B. (2010) 'Optimizing hypertension management in underserved rural populations'. *Journal of the National Medical Association* **102** (1), 10-17

Ballesteros, S., Kraft, E., Santana, S. & Tziraki, C. (2015) 'Maintaining older brain functionality: a targeted review'. *Neuroscience & Biobehavioral Reviews* **55**, 453-477

Balsari, S., Fortenko, A., Blaya, J.A., Gropper, A., Jayaram, M., Matthan, R., Sahasranam, R., Shankar, M., Sarbadhikari, S.N. & Bierer, B.E. (2018) 'Reimagining Health Data Exchange: An application programming interface–enabled roadmap for India'. *Journal of medical Internet research* **20** (7), e10725

Banchs, J.E. & Scher, D.L. (2015) 'Emerging role of digital technology and remote monitoring in the care of cardiac patients'. *Medical Clinics of North America* **99** (4), 877-896

Baraković, S., Husic, J.B., Belani, H., García, P.C., Coppola, I., Dantas, C., Ellen, M., Fernández-Vigil, M., Garcia, N. & Geambasu, R. (2019) 'Healthcare'. *State of the art report for smart habitat for older persons* , 171-255

Barbabella, F., Melchiorre, M.G., Quattrini, S., Papa, R. & Lamura, G. (2017)

Barbosa, W., Zhou, K., Waddell, E., Myers, T. & Dorsey, E.R. (2021) 'Improving Access to Care: Telemedicine Across Medical Domains'. *Annual Review of Public Health* **42**, 463-481

Barker, F., Mackenzie, E., Elliott, L., Jones, S. & de Lusignan, S. (2016) 'Interventions to improve hearing aid use in adult auditory rehabilitation'. *Cochrane Database of Systematic Reviews* (8)

BARNETT, K., LIVINGSTON, A., MARGELIS, G., TOMLINSON, G. & YOUNG, R. (2019) 'TECHNOLOGY AGEING AND AGED CARE: LITERATURE REVIEW'.

Bashi, N., Varnfield, M. & Karunanithi, M. (2020) 'A Smartphone App for Patients With Acute Coronary Syndrome (MoTER-ACS): User-Centered Design Approach'. *JMIR formative research* **4** (12), e17542

Bashi, N., Karunanithi, M., Fatehi, F., Ding, H. & Walters, D. (2017) 'Remote Monitoring of Patients With Heart Failure: An Overview of Systematic Reviews'. *Journal of Medical Internet Research* **19** (1), 1-13

Basholli, A., Lagkas, T., Bath, P.A. & Eleftherakis, G. (2021) 'Sensor-based platforms for remote management of chronic diseases in developing regions: A qualitative approach examining the perspectives of healthcare professionals'. *Health Informatics Journal* **27** (1), 1460458220979350

Bashshur, R.L., Shannon, G.W., Smith, B.R. & Woodward, M.A. (2015) 'The empirical evidence for the telemedicine intervention in diabetes management'. *Telemedicine and e-Health* **21** (5), 321-354

Basilakis, J., Lovell, N.H., Redmond, S.J. & Celler, B.G. (2010) 'Design of a decision-support architecture for management of remotely monitored patients.'. *IEEE Transactions on Information Technology in Biomedicine* **14** (5), 1216-1226

Batalin, M., Yuen, E., Dolezal, B., Smith, D., Cooper, C. & Mapar, J. (2013}) 'PHASER: Physiological Health Assessment System for Emergency Responders'. In Anonymous (eds.) *2013 IEEE INTERNATIONAL CONFERENCE ON BODY SENSOR NETWORKS (BSN)},* Held 345 E 47TH ST, NEW YORK, NY 10017 USA}: IEEE}

Batsis, J.A., McClure, A.C., Weintraub, A.B., Kotz, D.F., Rotenberg, S., Cook, S.B., Gilbert-Diamond, D., Curtis, K., Stevens, C.J. & Sette, D. (2019) 'Feasibility and acceptability of a rural, pragmatic, telemedicine-delivered healthy lifestyle programme'. *Obesity science & practice* **5** (6), 521-530

Batsis, J.A., Petersen, C.L., Clark, M.M., Cook, S.B., Lopez-Jimenez, F., Al-Nimr, R.I., Pidgeon, D., Kotz, D., Mackenzie, T.A. & Bartels, S.J. (2021) 'A Weight Loss Intervention Augmented by a Wearable Device in Rural Older Adults With Obesity: A Feasibility Study'. *The Journals of Gerontology: Series A* **76** (1), 95-100

Bauce K., Fahs D.B., Batten J. & Whittemore, R. (2018) 'Videoconferencing for Management of Heart Failure: An Integrative Review.'. *Journal of gerontological nursing* **44** (4), 45-52

Becker, G.A. (2019) *Investigating the Use of Wearable Activity Trackers to Determine Psychological Wellbeing*

Beckie, T.M. (2019) 'Utility of Home-Based Cardiac Rehabilitation for Older Adults'. *Clinics in geriatric medicine* **35** (4), 499-516

Beech, R., Russell, W., Little, R. & Sherlow-Jones, S. (2004) 'An evaluation of a multidisciplinary team for intermediate care at home.'. *International Journal of Integrated Care [Electronic Resource]* **4**, e02

Behar, J.A., Liu, C., Kotzen, K., Tsutsui, K., Corino, V.D., Singh, J., Pimentel, M.A., Warrick, P., Zaunseder, S. & Andreotti, F. (2020) 'Remote health diagnosis and monitoring in the time of COVID-19'. *Physiological Measurement* **41** (10), 10TR01

Behar, J.A., Liu, C., Tsutsui, K., Corino, V.D., Singh, J., Pimentel, M.A., Karlen, W., Warrick, P., Zaunseder, S. & Andreotti, F. (2020) 'Remote health monitoring in the time of COVID-19'. *arXiv preprint arXiv:2005.08537*

Belkacem, A.N., Jamil, N., Palmer, J.A., Ouhbi, S. & Chen, C. (2020) 'Brain computer interfaces for improving the quality of life of older adults and elderly patients'. *Frontiers in Neuroscience* **14**

Benden, M.E., Mancuso, L. & Pickens, A. (2011) 'The Ability of the SenseWear Armband to Assess a Change in Energy Expenditure in Children While Sitting and Standing.'. *Journal of Exercise Physiology Online* **14** (3)

Bendixen, R.M. (2006) *Assessment of a telerehabilitation and a telehomecare program for veterans with chronic illnesses*. Chronic Disease -- RehabilitationRehabilitation -- MethodsTelemedicineAgedArthritis -- ComplicationsBlood PressureBody WeightChronic Disease -- ComplicationsChronic Disease -- MortalityDiabetes Mellitus -- ComplicationsDisabledFunctional StatusHealth Care CostsHeart Diseases -- ComplicationsHome Health Care -- MethodsHome SafetyHospitalization -- EconomicsInterviewsLength of StayMonitoring, Physiologic -- MethodsPatient EducationPatient SatisfactionQualitative StudiesQuality of LifeRandom SampleSelf CareTelemedicine -- EconomicsUnited States Department of Veterans AffairsUnited StatesVeteransHuman, University of Florida

Bennett M.K., Shao M. & Gorodeski, E.Z. (2017) 'Home monitoring of heart failure patients at risk for hospital readmission using a novel under-the-mattress piezoelectric sensor: A preliminary single centre experience.'. *Journal of telemedicine and telecare* **23** (1), 60-67

Bensemmane, S. & Baeten, R. (2019) 'Cross-border telemedicine: practices and challenges'. *Observatoire social européen*

Berglund, M., Larsson, K., Grander, M., Casteleyn, L., Kolossa-Gehring, M., Schwedler, G., Castano, A., Esteban, M., Angerer, J., Koch, H.M., Schindler, B.K., Schoeters, G., Smolders, R., Exley, K., Sepai, O., Blumen, L., Horvat, M., Knudsen, L.E., Morck, T.A., Joas, A., Joas, R., Biot, P., Aerts, D., De Cremer, K., Van Overmeire, I., Katsonouri, A., Hadjipanayis, A., Cerna, M., Krskova, A., Nielsen, J.K.S., Jensen, J.F., Rudnai, P., Kozepesy, S., Griffin, C., Nesbitt, I., Gutleb, A.C., Fischer, M.E., Ligocka, D., Jakubowski, M., Reis, M.F., Namorado, S., Lupsa, I., Gurzau, A.E., Halzlova, K., Jajcaj, M., Mazej, D., Tratnik, J.S., Lopez, A., Canas, A., Lehmann, A., Crettaz, P., Den Hond, E. & Govarts, E. (2015) 'Exposure determinants of cadmium in European mothers and their children.'. *Environmental research* **141**, 69-76

Bergman, H., Kornør, H., Nikolakopoulou, A., Hanssen-Bauer, K., Soares-Weiser, K., Tollefsen, T.K. & Bjørndal, A. (2018) 'Client feedback in psychological therapy for children and adolescents with mental health problems'. *Cochrane Database of Systematic Reviews* (8)

Bernocchi, P., Scalvini, S., Galli, T., Paneroni, M., Baratti, D., Turla, O., La Rovere, M., Volterrani, M. & Vitacca, M. (2016) 'A multidisciplinary telehealth program in patients with combined chronic obstructive pulmonary disease and chronic heart failure: study protocol for a randomized controlled trial'. *Trials* **17** (1)

Bernocchi, P., Vitacca, M., La Rovere, M., Volterrani, M., Galli, T., Baratti, D., Paneroni, M., Campolongo, G., Sposato, B. & Scalvini, S. (2018) 'Home-based telerehabilitation in older patients with chronic obstructive pulmonary disease and heart failure: a randomised controlled trial'. *Age and Ageing* **47** (1), 82-88

Bernocchi, P., Scalvini, S., Galli, T., Paneroni, M., Baratti, D., Turla, O., La Rovere, M.T., Volterrani, M. & Vitacca, M. (2016) 'A multidisciplinary telehealth program in patients with combined chronic obstructive pulmonary disease and chronic heart failure: study protocol for a randomized controlled trial.'. *Trials [Electronic Resource]* **17** (1), 462

Bertoncello, C., Colucci, M., Baldovin, T., Buja, A. & Baldo, V. (2018) 'How does it work? Factors involved in telemedicine home-interventions effectiveness: A review of reviews'. *PLoS One* **13** (11), e0207332

Bertz, J.W., Epstein, D.H. & Preston, K.L. (2018) 'Combining ecological momentary assessment with objective, ambulatory measures of behavior and physiology in substance-use research'. *Addictive Behaviors* **83**, 5-17

Bhan, N., Madhira, P., Muralidharan, A., Kulkarni, B., Murthy, G.V.S., Basu, S. & Kinra, S. (2017}) 'Health needs, access to healthcare, and perceptions of ageing in an urbanizing community in India: a qualitative study'. *BMC GERIATRICS}* **17}**

Bhaskar, S., Banach, M., Weissert, R., Mittoo, S. & Nurtazina, A. 'TELEMEDICINE DURING AND BEYOND COVID-19'.

Bhaskar, S., Bradley, S., Chattu, V.K., Adisesh, A., Nurtazina, A., Kyrykbayeva, S., Sakhamuri, S., Moguilner, S., Pandya, S., Schroeder, S., Banach, M. & Ray, D. (2020) 'Telemedicine as the New Outpatient Clinic Gone Digital: Position Paper From the Pandemic Health System REsilience PROGRAM (REPROGRAM) International Consortium (Part 2)'. *Frontiers in public health* **8**, 410

Bhatia, R. (2021) 'Emerging Health Technologies and How They Can Transform Healthcare Delivery'. *Journal of Health Management* **23** (1), 63-73

Bhatia, G., Huang, Q., Crumley, T., Walters, C., Cluckers, L., Heirman, I., Mixson, L., Rottey, S., Cantor, M. & Benko, C. (2019) 'Mobile health technology captures in-house- data on the outside: an assessment with pharmacological intervention'. *Clinical pharmacology and therapeutics* **105**, S73-

Bhavnani, S.P., Narula, J. & Sengupta, P.P. (2016) 'Mobile technology and the digitization of healthcare'. *European heart journal* **37** (18), 1428-1438

Bhimaraj, A. (2013) 'Remote monitoring of heart failure patients'. *Methodist DeBakey cardiovascular journal* **9** (1), 26-31

Bhuiyan, M.N., Rahman, M.M., Billah, M.M. & Saha, D. (2021) 'Internet of Things (IoT): A review of its enabling technologies in healthcare applications, standards protocols, security and market opportunities'. *IEEE Internet of Things Journal*

Bidargaddi, N. (2017) 'Learning from development of a third-party patient-oriented application using Australian national personal health records system'. *arXiv preprint arXiv:1709.03577*

Biddiss, E., Brownsell, S. & Hawley, M.S. (2009) 'Predicting need for intervention in individuals with congestive heart failure using a home-based telecare system.'. *Journal of Telemedicine & Telecare* **15** (5), 226-231

Bidonde, J., Busch, A.J., Webber, S.C., Schachter, C.L., Danyliw, A., Overend, T.J., Richards, R.S. & Rader, T. (2014) 'Aquatic exercise training for fibromyalgia'. *Cochrane Database of Systematic Reviews* (10)

Biersteker, T.E., Boogers, M.J., de Lind van Wijngaarden,Robert Af, Groenwold, R.H., Trines, S.A., van Alem, A.P., Kirchhof, C.J., van Hof, N., Klautz, R.J., Schalij, M.J. & Treskes, R.W. (2020) 'Use of Smart Technology for the Early Diagnosis of Complications After Cardiac Surgery: The Box 2.0 Study Protocol.'. *JMIR Research Protocols* **9** (4), e16326

Bijlsma, N. & Cohen, M.M. (2016) 'Environmental chemical assessment in clinical practice: Unveiling the elephant in the room'. *International journal of environmental research and public health* **13** (2), 181

Billing, L. (2017) 'The efficacy of affective behavioral strategies for increasing physical activity: Implications for harnessing the dual-mode model'.

Bin, J.P., Doctor, A., Lindner, J., Hendersen, E.M., Le, D.E., Leong-Poi, H., Fisher, N.G., Christiansen, J. & Kaul, S. (2006) 'Effects of nitroglycerin on erythrocyte rheology and oxygen unloading: novel role of S-nitrosohemoglobin in relieving myocardial ischemia'. *Circulation* **113** (21), 2502-2508

Bingler, M., Erickson, L.A., Reid, K.J., Lee, B., O'Brien, J., Apperson, J., Goggin, K. & Shirali, G. (2018) 'Interstage Outcomes in Infants With Single Ventricle Heart Disease Comparing Home Monitoring Technology to Three-Ring Binder Documentation: a Randomized Crossover Study'. *World journal for pediatric & congenital heart surgery* **9** (3), 305-314

Biondi, A., Laiou, P., Bruno, E., Viana, P.F., Schreuder, M., Hart, W., Nurse, E., Pal, D.K. & Richardson, M.P. (2021) 'Remote and Long-Term Self-Monitoring of Electroencephalographic and Noninvasive Measurable Variables at Home in Patients With Epilepsy (EEG@HOME): Protocol for an Observational Study.'. *JMIR Research Protocols* **10** (3), e25309

Biswas, D. (2019) 'Remote Healthcare Technology: A New Paradigm'. In *Health Monitoring Systems* CRC Press, 1-17

Bizopoulos, P. & Koutsouris, D. (2018) 'Deep learning in cardiology'. *IEEE reviews in biomedical engineering* **12**, 168-193

Black, J.T., Romano, P.S., Sadeghi, B., Auerbach, A.D., Ganiats, T.G., Greenfield, S., Kaplan, S.H. & Ong, M.K. (2014) 'A remote monitoring and telephone nurse coaching intervention to reduce readmissions among patients with heart failure: study protocol for the Better Effectiveness After Transition - Heart Failure (BEAT-HF) randomized controlled trial'. *Trials* **15**, 124

Blair, C.K., Harding, E., Herman, C., Boyce, T., Demark-Wahnefried, W., Davis, S., Kinney, A.Y. & Pankratz, V.S. (2020) 'Remote Assessment of Functional Mobility and Strength in Older Cancer Survivors: Protocol for a Validity and Reliability Study'. *JMIR research protocols* **9** (9), e20834

Blair, C.K., Harding, E., Wiggins, C., Kang, H., Schwartz, M., Tarnower, A., Du, R. & Kinney, A.Y. (2021) 'A Home-Based Mobile Health Intervention to Replace Sedentary Time With Light Physical Activity in Older Cancer Survivors: Randomized Controlled Pilot Trial'. *JMIR cancer* **7** (2), e18819

Blanck, Z., Axtell, K., Brodhagen, K., O'Hearn, L., Albelo, T., Ceretto, C., Dhala, A., Sra, J. & Akhtar, M. (2011) 'Inappropriate shocks in patients with Fidelis R lead fractures: impact of remote monitoring and the lead integrity algorithm.'. *Journal of cardiovascular electrophysiology* **22** (10), 1107-1114

Blaya, J.A., Fraser, H.S. & Holt, B. (2010) 'E-health technologies show promise in developing countries'. *Health affairs* **29** (2), 244-251

Blumenthal, J. (2017) *A Mobile Application for Dual-Task Gait Assessment of Cognitive Status*

Bodicoat, D.H., O'Donovan, G., Dalton, A.M., Gray, L.J., Yates, T., Edwardson, C., Hill, S., Webb, D.R., Khunti, K., Davies, M.J. & Jones, A.P. (2014) 'The association between neighbourhood greenspace and type 2 diabetes in a large cross-sectional study'. *BMJ open* **4** (12), e006076-2014-006076

Boele, F.W., Rooney, A.G., Bulbeck, H. & Sherwood, P. (2019) 'Interventions to help support caregivers of people with a brain or spinal cord tumour'. *Cochrane Database of Systematic Reviews* (7)

Bofill Rodriguez, M., Lethaby, A., Farquhar, C. & Duffy, J. (2020) 'Interventions commonly available during pandemics for heavy menstrual bleeding: an overview of Cochrane Reviews'. *Cochrane Database of Systematic Reviews* (7)

Bonato, P. (2009) 'Advances in wearable technology for rehabilitation'. *Studies in health technology and informatics* **145**, 145-159

Bonnevie, T., Gravier, F., Elkins, M., Dupuis, J., Prieur, G., Combret, Y., Viacroze, C., Debeaumont, D., Robleda-Quesada, A., Quieffin, J., Lamia, B., Patout, M., Cuvelier, A., Muir, J., Medrinal, C. & Tardif, C. (2019) 'People undertaking pulmonary rehabilitation are willing and able to provide accurate data via a remote pulse oximetry system: a multicentre observational study'. *Journal of Physiotherapy (Elsevier)* **65** (1), 28-36

Boodoo, C., Perry, J.A., Hunter, P.J., Duta, D.I., Newhook, S.C.P., Leung, G. & Cross, K. (2017) 'Views of patients on using mHealth to monitor and prevent diabetic foot ulcers: qualitative study'. *JMIR diabetes* **2** (2), e22

Borghouts, J., Eikey, E., Mark, G., De Leon, C., Schueller, S.M., Schneider, M., Stadnick, N., Zheng, K., Mukamel, D. & Sorkin, D.H. (2021) 'Barriers to and facilitators of user engagement with digital mental health interventions: systematic review'. *Journal of medical Internet research* **23** (3), e24387

Borzie, K., Jasper, N., Southall, D.P., MacDonald, R., Kola, A.A., Dolo, O., Magnus, A., Watson, S.D., Casement, M., Dahn, B. & Jallah, W. (2020) 'Monitoring intrapartum fetal heart rates by mothers in labour in two public hospitals: an initiative to improve maternal and neonatal healthcare in Liberia.'. *BMC Pregnancy & Childbirth* **20** (1), 362

Bossen, A.L., Kim, H., Williams, K.N., Steinhoff, A.E. & Strieker, M. (2015) 'Emerging roles for telemedicine and smart technologies in dementia care'. *Smart homecare technology and telehealth* **3**, 49-57

Bosworth, H.B., Powers, B.J. & Oddone, E.Z. (2010) 'Patient self-management support: novel strategies in hypertension and heart disease'. *Cardiology clinics* **28** (4), 655-663

Botsis, T. & Hartvigsen, G. (2008) 'Current status and future perspectives in telecare for elderly people suffering from chronic diseases'. *Journal of telemedicine and telecare* **14** (4), 195-203

Bott, N., Kumar, S., Krebs, C., Glenn, J.M., Madero, E.N. & Juusola, J.L. (2018) 'A remote intervention to prevent or delay cognitive impairment in older adults: design, recruitment, and baseline characteristics of the Virtual Cognitive Health (VC Health) study'. *JMIR research protocols* **7** (8), e11368

Boudy, J., Baldinger, J.-., Delavault, F., Muller, M., Farin, I., Andreao, R.V., Torres-Mueller, S., Serra, A., Gaiti, D., Rocaries, F., Dietrich, C., Lacombe, A., Steenkeste, F., Schaff, M., Baer, M., Ozguler, A. & Vaysse, S. (2006}) 'Telemedicine for elderly patient at home: the TelePat project'. In Nugent, C. and Augusto, J. (eds.) *SMART HOMES AND BEYOND},* Held NIEUWE HEMWEG 6B, 1013 BG AMSTERDAM, NETHERLANDS}: IOS PRESS}, 74}

Boyer, S.L., Silka, M.J. & Bar-Cohen, Y. (2015) 'Current practices in the monitoring of cardiac rhythm devices in pediatrics and congenital heart disease.'. *Pediatric cardiology* **36** (4), 821-826

Bradley, E.A., Berman, D. & Daniels, C.J. (2016) *First implantable hemodynamic monitoring device placement in single ventricle fontan anatomy*

Bradley, P.M., Lindsay, B. & Fleeman, N. (2016) 'Care delivery and self management strategies for adults with epilepsy'. *Cochrane Database of Systematic Reviews* (2)

Bradway, M., Gabarron, E., Johansen, M., Zanaboni, P., Jardim, P., Joakimsen, R., Pape-Haugaard, L. & Årsand, E. (2020) 'Methods and Measures Used to Evaluate Patient-Operated Mobile Health Interventions: Scoping Literature Review'. *JMIR mHealth and uHealth* **8** (4), e16814

Brasier N., Geissmann L., Kach M., Mutke M., Hoelz B., De Ieso F. & Eckstein, J. (2020) 'Device-and Analytics-Agnostic Infrastructure for Continuous Inpatient Monitoring: A Technical Note.'. *Digital Biomarkers* **4** (2), 62-68

Bratan, T., Clarke, M., Jones, R., Larkworthy, A. & Paul, R. (2005) 'Evaluation of the practical feasibility and acceptability of home monitoring in residential homes.'. *Journal of Telemedicine & Telecare* **11** (Suppl 1), 29-31

Bravo-Escobar, R., González-Represas, A., Gómez-González, A., Montiel-Trujillo, A., Aguilar-Jimenez, R., Carrasco-Ruíz, R. & Salinas-Sánchez, P. (2017) 'Effectiveness and safety of a home-based cardiac rehabilitation programme of mixed surveillance in patients with ischemic heart disease at moderate cardiovascular risk: a randomised, controlled clinical trial'. *BMC cardiovascular disorders* **17** (1), 66

Bravo-Escobar, R., Gonzalez-Represas, A., Gomez-Gonzalez, A.M. & Heredia-Torres, A. (2021) 'Effectiveness of e-Health cardiac rehabilitation program on quality of life associated with symptoms of anxiety and depression in moderate-risk patients.'. *Scientific Reports* **11** (1), 3760

Brennan, T., Spettell, C., Villagra, V., Ofili, E., McMahill-Walraven, C., Lowy, E.J., Daniels, P., Quarshie, A. & Mayberry, R. (2010) 'Disease management to promote blood pressure control among African Americans.'. *Population Health Management* **13** (2), 65-72

Breteler, M.J.M.M., Huizinga, E., van Loon, K., Leenen, L.P.H., Dohmen, D.A.J., Kalkman, C.J. & Blokhuis, T.J. (2018) 'Reliability of wireless monitoring using a wearable patch sensor in high-risk surgical patients at a step-down unit in the Netherlands: a clinical validation study.'. *BMJ Open* **8** (2), e020162

Briggs, J., Adams, C., Fallahkhair, S., Iluyemi, A. & Prytherch, D. (2012) 'M-health review: joining up healthcare in a wireless world'.

Brijs, J., Sandblom, E., Axelsson, M., Sundell, K., Sundh, H., Kiessling, A., Berg, C. & Grans, A. (2019) 'Remote physiological monitoring provides unique insights on the cardiovascular performance and stress responses of freely swimming rainbow trout in aquaculture.'. *Scientific Reports* **9** (1), 9090

Brook, R.D., Xu, X., Bard, R.L., Dvonch, J.T., Morishita, M., Kaciroti, N., Sun, Q., Harkema, J. & Rajagopalan, S. (2013) 'Reduced metabolic insulin sensitivity following sub-acute exposures to low levels of ambient fine particulate matter air pollution.'. *Science of the Total Environment* **448**, 66-71

Brouwers R.W.M., Kraal J.J., Traa S.C.J., Spee R.F., Oostveen L.M.L.C. & Kemps, H.M.C. (2017) 'Effects of cardiac telerehabilitation in patients with coronary artery disease using a personalised patient-centred web application: Protocol for the SmartCare-CAD randomised controlled trial.'. *BMC Cardiovascular Disorders* **17** (1) (pagination), Arte Number: 46. ate of Pubaton: 31 Jan 2017

Brouwers, R.W., Kraal, J.J., Traa, S.C., Spee, R.F., Oostveen, L.M. & Kemps, H.M. (2017) 'Effects of cardiac telerehabilitation in patients with coronary artery disease using a personalised patient-centred web application: protocol for the SmartCare-CAD randomised controlled trial'. *BMC cardiovascular disorders* **17** (1), 46

Brower, J., LaBarge, M.C., White, L. & Mitchell, M.S. (2020) 'Examining Responsiveness to an Incentive-Based Mobile Health App: Longitudinal Observational Study'. *Journal of medical Internet research* **22** (8), e16797

Brown, S., Rhee, J., Guha, A. & Rao, V.U. (2020) 'Innovation in precision cardio-oncology during the coronavirus pandemic and into a post-pandemic world'. *Frontiers in Cardiovascular Medicine* **7**

Brown, T., Moore, T., Hooper, L., Gao, Y., Zayegh, A., Ijaz, S., Elwenspoek, M., Foxen, S.C., Magee, L. & O'Malley, C. (2019) 'Interventions for preventing obesity in children'. *Cochrane Database of Systematic Reviews* (7)

Browning, S.V., Clark, R.C., Poff, R. & Todd, D. (2011) 'Telehealth monitoring: a smart investment for home care patients with heart failure?'. *Home healthcare nurse* **29** (6), 368-374

Brunetti, N.D., Scalvini, S. & Molinari, G. (2016) 'Innovations in telemedicine for cardiovascular care'. *Expert Review of Cardiovascular Therapy* **14** (3), 267-280

Brunetti, N.D., Scalvini, S. & Molinari, G. (2016) 'Innovations in telemedicine for cardiovascular care.'. *Expert Review of Cardiovascular Therapy* **14** (3), 267-280

Buckingham, S.A., Morrissey, K., Williams, A.J., Price, L. & Harrison, J. (2020) 'The Physical Activity Wearables in the Police Force (PAW-Force) study: acceptability and impact'. *BMC public health* **20** (1), 1-16

Budinger, T.F. (2003) 'Biomonitoring with wireless communications'. *Annual Review of Biomedical Engineering* **5**, 383-412

Bufalino, V.J., Bleser, W.K., Singletary, E.A., Granger, B.B., O'Brien, E.C., Elkind, M.S., Hamilton Lopez, M., Saunders, R.S., McClellan, M.B. & Brown, N. (2020) 'Frontiers of Upstream Stroke Prevention and Reduced Stroke Inequity Through Predicting, Preventing, and Managing Hypertension and Atrial Fibrillation: A Call to Action From the Value in Healthcare Initiative's Predict & Prevent Learning Collaborative'. *Circulation: Cardiovascular Quality and Outcomes* **13** (7), e006780

Bui, A.L., Fonarow, G.C., Bui, A.L. & Fonarow, G.C. (2012) 'Home monitoring for heart failure management'. *Journal of the American College of Cardiology (JACC)* **59** (2), 97-104

Bullard, T. (2019) *Testing a brief, theory-guided video chat intervention for enhancing self-efficacy and lifestyle physical activity among low active working adults*

Buonaguro, F.M., Botti, G., Ascierto, P.A., Pignata, S., Ionna, F., Delrio, P., Petrillo, A., Cavalcanti, E., Di Bonito, M., Perdonà, S., De Laurentiis, M., Fiore, F., Palaia, R., Izzo, F., D'Auria, S., Rossi, V., Menegozzo, S., Piccirillo, M., Celentano, E. & Cuomo, A. (2020) 'The clinical and translational research activities at the INT – IRCCS "Fondazione Pascale" cancer center (Naples, Italy) during the COVID-19 pandemic'. *Infectious Agents & Cancer* **15** (1), N.PAG-N.PAG

Burdea, G., Kim, N., Polistico, K., Kadaru, A., Grampurohit, N., Roll, D. & Damiani, F. (2019) 'Assistive game controller for artificial intelligence-enhanced telerehabilitation post-stroke'. *Assistive Technology* , 1-12

Burgess, K.R., Havryk, A., Newton, S., Tsai, W.H. & Whitelaw, W.A. (2013) 'Targeted case finding for OSA within the primary care setting.'. *Journal of Clinical Sleep Medicine* **9** (7), 681-686

Burns, J., Boogaard, H., Polus, S., Pfadenhauer, L.M., Rohwer, A.C., van Erp, A., Turley, R. & Rehfuess, E. (2019) 'Interventions to reduce ambient particulate matter air pollution and their effect on health'. *Cochrane Database of Systematic Reviews* (5)

Burri, H., Sticherling, C., Wright, D., Makino, K., Smala, A. & Tilden, D. (2013) 'Cost-consequence analysis of daily continuous remote monitoring of implantable cardiac defibrillator and resynchronization devices in the UK'. *EP: Europace* **15** (11), 1601-1608

Burton, S.D., Tanczer, L.M., Vasudevan, S., Hailes, S. & Carr, M. 'TheUKCode ofPracticefor ConsumerIoT Security'.

But-Hadzic, J., Dervisevic, M., Karpljuk, D., Videmsek, M., Dervisevic, E., Paravlic, A., Hadzic, V. & Tomazin, K. (2021) 'Six-Minute Walk Distance in Breast Cancer Survivors—A Systematic Review with Meta-Analysis'. *International journal of environmental research and public health* **18** (5), 2591

Butler Tobah, Y., LeBlanc, A., Branda, M.E., Inselman, J.W., Morris, M.A., Ridgeway, J.L., Finnie, D.M., Theiler, R., Torbenson, V.E. & Brodrick, E.M. (2019) 'Randomized comparison of a reduced-visit prenatal care model enhanced with remote monitoring'. *American Journal of Obstetrics and Gynecology* **221** (6), 638.e1-638.e8

Byambasuren, O. (2020) 'Effectiveness of mHealth apps and their prescribability in general practice'.

Byrne, M.D. (2019) 'Expanding Perianesthesia Practice With Connected Care'. *Journal of perianesthesia nursing : official journal of the American Society of riAnesthesia Nurses* **34** (1), 211-215

Caffrey, M. (2017) 'Omada's Paul Chew, MD: From Treating Chronic Disease to Prevention'. *Omada's Paul Chew, MD: From Treating Chronic Disease to Prevention* , 451

Cahill, L.S., Carey, L.M., Lannin, N.A., Turville, M., Neilson, C.L., Lynch, E.A., McKinstry, C.E., Han, J.X. & O'Connor, D. (2020) 'Implementation interventions to promote the uptake of evidence-based practices in stroke rehabilitation'. *Cochrane Database of Systematic Reviews* (10)

Cajamarca, G., Herskovic, V. & Rossel, P.O. (2020) 'Enabling Older Adults' Health Self-Management through Self-Report and Visualization—A Systematic Literature Review'. *Sensors* **20** (15), 4348

Cajita, M.I. (2017) *mHealth Use in Older People with Heart Failure*

Calano, B.J.D., Cacal, M.J.B., Cal, C.B., Calletor, K.P., Guce, F.I.C.C., Bongar, M.V.V. & Macindo, J.R.B. (2019) 'Effectiveness of a community-based health programme on the blood pressure control, adherence and knowledge of adults with hypertension: A PRECEDE-PROCEED model approach'. *Journal of Clinical Nursing (John Wiley & Sons, Inc.)* **28** (9), 1879-1888

Cameron, I.D., Dyer, S.M., Panagoda, C.E., Murray, G.R., Hill, K.D., Cumming, R.G. & Kerse, N. (2018) 'Interventions for preventing falls in older people in care facilities and hospitals'. *Cochrane Database of Systematic Reviews* (9)

Campbell, K.M. & Rodriguez, J.E. (2016) 'Cardiovascular Health Practices Among Black Patients in an Urban Underserved Clinic.'. *Journal of the National Medical Association* **108** (1), 40-44

Canha, N., Almeida, S.M., Freitas, M.C. & Wolterbeek, H.T. (2014) 'Indoor and outdoor biomonitoring using lichens at urban and rural primary schools.'. *Journal of Toxicology & Environmental Health Part A* **77** (14-16), 900-915

Capozzi, F., Di Palma, A., Adamo, P., Sorrentino, M.C., Giordano, S. & Spagnuolo, V. (2019) 'Indoor vs. outdoor airborne element array: A novel approach using moss bags to explore possible pollution sources.'. *Environmental Pollution* **249**, 566-572

Cardozo Lavoisier, J., Joel, S., Shaun, C., Veeranna, V., Bibban, D. & Marybeth, L. (2011) 'Implementing the chronic disease self management model in vulnerable patient populations: bridging the chasm through telemedicine'. *Advances in Telemedicine: Technologies, Enabling Factors and Scenarios* , 357

Carrazco, C., Golyan, D., Kahen, M., Black, K., Libman, R.B. & Katz, J.M. (2018) 'Prevalence and Risk Factors for Paroxysmal Atrial Fibrillation and Flutter Detection after Cryptogenic Ischemic Stroke.'. *Journal of Stroke & Cerebrovascular Diseases* **27** (1), 203-209

Carretero, S. (2015) 'Mapping of effective technology-based services for independent living for older people at home'. *European Commission-Joint Research Centre-Institute for Prospective Technological Studies.doi* **10**, 395556

Carrington, M.J. & Zimmet, P.Z. (2021) 'Nurse co-ordinated health and lifestyle modification for reducing multiple cardio-metabolic risk factors in regional adults: outcomes from the MODERN randomized controlled trial'. *European Journal of Cardiovascular Nursing*

Carson, K.V., Brinn, M.P., Labiszewski, N.A., Esterman, A.J., Chang, A.B. & Smith, B.J. (2011) 'Community interventions for preventing smoking in young people'. *Cochrane Database of Systematic Reviews* (7)

Carson, K.V., Brinn, M.P., Peters, M., Veale, A., Esterman, A.J. & Smith, B.J. (2012) 'Interventions for smoking cessation in Indigenous populations'. *Cochrane Database of Systematic Reviews* (1)

Carter, D.D., Robinson, K., Forbes, J. & Hayes, S. (2018) 'Experiences of mobile health in promoting physical activity: A qualitative systematic review and meta-ethnography'. *PLoS One* **13** (12), e0208759

Castle-Clarke, S. & Imison, C. (2016) 'The digital patient: transforming primary care'. *London: Nuffield Trust*

Catalan-Matamoros, D., Lopez-Villegas, A., Lappegard, K.T. & Lopez-Liria, R. (2020) 'Assessing Communication during Remote Follow-Up of Users with Pacemakers in Norway: The NORDLAND Study, a Randomized Trial.'. *International Journal of Environmental Research & Public Health [Electronic Resource]* **17** (20), 10 21

Catling, C.J., Medley, N., Foureur, M., Ryan, C., Leap, N., Teate, A. & Homer, C. (2015) 'Group versus conventional antenatal care for women'. *Cochrane Database of Systematic Reviews* (2)

Cattelani, L., Palumbo, P., Palmerini, L., Bandinelli, S., Becker, C., Chesani, F. & Chiari, L. (2015) 'FRAT-up, a Web-based fall-risk assessment tool for elderly people living in the community'. *Journal of medical Internet research* **17** (2), e4064

Caufield, J.H., Sigdel, D., Fu, J., Choi, H., Guevara-Gonzalez, V., Wang, D. & Ping, P. (2021) 'Cardiovascular Informatics: building a bridge to data harmony'. *Cardiovascular research*

Celermajer, D.S., Chow, C.K., Marijon, E., Anstey, N.M. & Woo, K.S. (2012) 'Cardiovascular disease in the developing world: prevalences, patterns, and the potential of early disease detection'. *Journal of the American College of Cardiology* **60** (14), 1207-1216

Celic, L., Trogrlic, D., Paladin, I., Prasek, M. & Magjarevic, R. (2012}) 'Integration of Measurement Devices Supporting Diabetic Patients into a Remote Care System'. In Jobbagy, A. (eds.) *5TH EUROPEAN CONFERENCE OF THE INTERNATIONAL FEDERATION FOR MEDICAL AND BIOLOGICAL ENGINEERING, PTS 1 AND 2},* Held 233 SPRING STREET, NEW YORK, NY 10013, UNITED STATES}: SPRINGER}, 39}

Celler, B.G. & Sparks, R.S. (2014) 'Home telemonitoring of vital signs—technical challenges and future directions'. *IEEE journal of biomedical and health informatics* **19** (1), 82-91

Censi, F., Calcagnini, G., Mattei, E., Calo, L., Curnis, A., D'Onofrio, A., Vaccari, D., Zanotto, G., Morichelli, L., Rovai, N., Gargaro, A. & Ricci, R.P. (2017) 'Seasonal trends in atrial fibrillation episodes and physical activity collected daily with a remote monitoring system for cardiac implantable electronic devices.'. *International journal of cardiology* **234**, 48-52

Centeno Tablante, E., Pachón, H., Guetterman, H.M. & Finkelstein, J.L. (2019) 'Fortification of wheat and maize flour with folic acid for population health outcomes'. *Cochrane Database of Systematic Reviews* (7)

Chamberlain, P., Reed, H., Burton, M. & Mountain, G. (2011) 'Future bathroom: A study of user-centred design principles affecting usability, safety and satisfaction in bathrooms for people living with disabilities'.

Chamberlain, I.J. & Sampson, S. (2013) 'Nidotherapy for people with schizophrenia'. *Cochrane Database of Systematic Reviews* (3)

Chan C., Sounderajah V., Normahani P., Acharya A., Markar S.R., Darzi A., Bicknell C. & Riga, C. (2021) 'Wearable Activity Monitors in Home Based Exercise Therapy for Patients with Intermittent Claudication: A Systematic Review.'. *European Journal of Vascular and Endovascular Surgery* **61** (4), 676-687

Chan, M., Campo, E., Estève, D. & Fourniols, J. (2009) 'Smart homes—current features and future perspectives'. *Maturitas* **64** (2), 90-97

Chan, M., Estève, D., Escriba, C. & Campo, E. (2008) 'A review of smart homes—Present state and future challenges'. *Computer methods and programs in biomedicine* **91** (1), 55-81

Chan, M., Estève, D., Fourniols, J., Escriba, C. & Campo, E. (2012) 'Smart wearable systems: Current status and future challenges'. *Artificial Intelligence in Medicine* **56** (3), 137-156

Chan, A.M., Selvaraj, N., Ferdosi, N. & Narasimhan, R. (2013) 'Wireless patch sensor for remote monitoring of heart rate, respiration, activity, and falls.'. *Annual International Conference Of The IEEE Engineering In Medicine And Biology Society* **2013**, 6115-6118

Chan, C., Sounderajah, V., Normahani, P., Acharya, A., Markar, S.R., Darzi, A., Bicknell, C. & Riga, C. (2021) 'Wearable Activity Monitors in Home Based Exercise Therapy for Patients with Intermittent Claudication: A Systematic Review.'. *European Journal of Vascular & Endovascular Surgery* **61** (4), 676-687

Chang, C., Hsu, Y., Li, F., Tu, Y., Jhang, W., Hsu, C., Huang, C. & Ho, C. (2020) 'Reliability and validity of the physical activity monitor for assessing energy expenditures in sedentary, regularly exercising, non-endurance athlete, and endurance athlete adults'. *PeerJ* **8**, e9717

Chang, L., Chattopadhyay, K., Li, J., Xu, M. & Li, L. (2021) 'Interplay of Support, Comparison, and Surveillance in Social Media Weight Management Interventions: Qualitative Study'. *JMIR mHealth and uHealth* **9** (3), e19239

Chang, L., Chattopadhyay, K., Li, J., Xu, M., Li, L., Balbim, G., Marques, I., Marquez, D., Patel, D. & Sharp, L. (2021) 'JMIR mHealth and uHealth'. *JMIR* **9** (3), 1

Chang, R.C., Lu, H., Yang, P. & Luarn, P. (2016) 'Reciprocal reinforcement between wearable activity trackers and social network services in influencing physical activity behaviors'. *JMIR mHealth and uHealth* **4** (3), e84

Charness, N. (2010) 'The health care challenge: Matching care to people in their home environments'. In Anonymous (eds.) *S. Olson (Rapporteur.), The role of human factors in home health care: A workshop summary,* Held , 73-116

Charness, N., Boot, W.R. & Gray, N. (2020) 'Mobile Monitoring and Intervention (MMI) Technology for Adaptive Aging'. In Anonymous (eds.) *Mobile Technology for Adaptive Aging: Proceedings of a Workshop,* Held National Academies Press

Chehade, M.J., Yadav, L., Jayatilaka, A., Gill, T.K. & Palmer, E. (2020) 'Personal digital health hubs for multiple conditions'. *Bulletin of the World Health Organization* **98** (8), 569

Chen, J., Lin, Y. & Shen, B. (2017) 'Informatics for precision medicine and healthcare'. *Translational Informatics in Smart Healthcare* , 1-20

Chen, Y., Perez-Cueto, F.J., Giboreau, A., Mavridis, I. & Hartwell, H. (2020) 'The Promotion of Eating Behaviour Change through Digital Interventions'. *International Journal of Environmental Research and Public Health* **17** (20), 7488

Chen, H., Zhou, L. & Yu, J. (2010) '[Development and application of a portable device for cardiac remote monitoring]'. *Shengwu Yixue Gongchengxue Zazhi/Journal of Biomedical Engineering* **27** (3), 666-670

Chen, I., Opiyo, N., Tavender, E., Mortazhejri, S., Rader, T., Petkovic, J., Yogasingam, S., Taljaard, M., Agarwal, S. & Laopaiboon, M. (2018) 'Non-clinical interventions for reducing unnecessary caesarean section'. *Cochrane Database of Systematic Reviews* (9)

Chevallier, T., Buzancais, G., Occean, B., Rataboul, P., Boisson, C., Simon, N., Lannelongue, A., Chaniaud, N., Gricourt, Y., Lefrant, J. & Cuvillon, P. (2020) 'Feasibility of remote digital monitoring using wireless Bluetooth monitors, the Smart Angel TM app and an original web platform for patients following outpatient surgery: a prospective observational pilot study.'. *BMC Anesthesiology* **20** (1), 259

Chevallier, T., Buzancais, G., Occean, B., Rataboul, P., Boisson, C., Simon, N., Lannelongue, A., Chaniaud, N., Gricourt, Y., Lefrant, J. & Cuvillon, P. (2020) 'Feasibility of remote digital monitoring using wireless Bluetooth monitors, the Smart Angel™ app and an original web platform for patients following outpatient surgery: a prospective observational pilot study'. *BMC Anesthesiology* **20** (1), N.PAG-N.PAG

Chew, B.H., Vos, R.C., Metzendorf, M.I., Scholten, R. & Rutten, G. (2017) 'Psychological interventions for diabetes-related distress in adults with type 2 diabetes mellitus'. *Cochrane Database of Systematic Reviews* (9)

Chiang, L., Chen, W., Dai, Y. & Ho, Y. (2012}) 'The effectiveness of telehealth care on caregiver burden, mastery of stress, and family function among family caregivers of heart failure patients:

A quasi-experimental study'. *INTERNATIONAL JOURNAL OF NURSING STUDIES}* **49}** (10}), 1230-1242}

Chiang, L., Chen, W., Dai, Y. & Ho, Y. (2012) 'The effectiveness of telehealth care on caregiver burden, mastery of stress, and family function among family caregivers of heart failure patients: a quasi-experimental study.'. *International journal of nursing studies* **49** (10), 1230-1242

ChiCTR-RNR-17012446, (2017) 'An Exploratory Clinical Study on Effect of Home-based Cardiac Exercise Rehabilitation with Remote Electrocardiogram Monitoring in Patients with Chronic Heart Failure'. *http://www.who.int/trialsearch/Trial2.aspx?TrialID=ChiCTR-RNR-17012446*

Chisi, T.F.T. (2019) *Determining the potential of wearable technologies within the disease landscape of sub-Saharan Africa*

Cho, Y., Lee, S., Islam, S.M.S. & Kim, S. (2018) 'Theories applied to m-health interventions for behavior change in low-and middle-income countries: a systematic review'. *Telemedicine and e-Health* **24** (10), 727-741

Choi, L., Majambere, S. & Wilson, A.L. (2019) 'Larviciding to prevent malaria transmission'. *Cochrane Database of Systematic Reviews* (8)

Choi, W.S., Choi, J.H., Oh, J., Shin, I. & Yang, J. (2020}) 'Effects of Remote Monitoring of Blood Pressure in Management of Urban Hypertensive Patients: A Systematic Review and Meta-Analysis'. *TELEMEDICINE AND E-HEALTH}* **26}** (6}), 744-759}

Choi, W.S., Choi, J.H., Oh, J., Shin, I. & Yang, J. (2020) 'Effects of Remote Monitoring of Blood Pressure in Management of Urban Hypertensive Patients: A Systematic Review and Meta-Analysis.'. *Telemedicine Journal & E-Health* **26** (6), 744-759

Chokshi, N.P., Adusumalli, S., Small, D.S., Morris, A., Feingold, J., Ha, Y.P., Lynch, M.D., Rareshide, C.A.L., Hilbert, V. & Patel, M.S. (2018) 'Loss-Framed Financial Incentives and Personalized Goal-Setting to Increase Physical Activity Among Ischemic Heart Disease Patients Using Wearable Devices: The ACTIVE REWARD Randomized Trial'. *Journal of the American Heart Association* **7** (12), e009173. doi: 10.1161/JAHA.118.009173

Christle, J.W., Hershman, S.G., Soto, J.T. & Ashley, E.A. (2020) 'Mobile Health Monitoring of Cardiac Status'.

Chung, K., Kim, S., Lee, E. & Park, J.Y. (2020) 'Mobile App Use for Insomnia Self-Management in Urban Community-Dwelling Older Korean Adults: Retrospective Intervention Study'. *JMIR mHealth and uHealth* **8** (8), e17755

Chung, J., Lee, J.H., Choi, J., Lee, J., Kim, W.G., Sun, K. & Min, B.G. (2004) 'Home care artificial heart monitoring system via internet.'. *International Journal of Artificial Organs* **27** (10), 898-903

Ciapponi, A., Lewin, S., Herrera, C.A., Opiyo, N., Pantoja, T., Paulsen, E., Rada, G., Wiysonge, C.S., Bastías, G. & Dudley, L. (2017) 'Delivery arrangements for health systems in low-income countries: an overview of systematic reviews'. *Cochrane Database of Systematic Reviews* (9)

Ciere, Y., Cartwright, M. & Newman, S.P. (2012) 'A systematic review of the mediating role of knowledge, self-efficacy and self-care behaviour in telehealth patients with heart failure'. *Journal of Telemedicine & Telecare* **18** (7), 384-391

Cikajlo, I., Sprager, S., Erjavec, T. & Zazula, D. (2016}) 'Cardiac arrhythmia alarm from optical interferometric signals during resting or sleeping for early intervention'. *BIOCYBERNETICS AND BIOMEDICAL ENGINEERING}* **36}** (1}), 267-275}

Claes, J., Cornelissen, V., McDermott, C., Moyna, N., Pattyn, N., Cornelis, N., Gallagher, A., McCormack, C., Newton, H. & Gillain, A. (2020) 'Feasibility, acceptability, and clinical effectiveness of a technology-enabled cardiac rehabilitation platform (Physical Activity Toward Health-I): randomized controlled trial'. *Journal of medical Internet research* **22** (2), e14221

Claes, J., Buys, R., Woods, C., Briggs, A., Geue, C., Aitken, M., Moyna, N., Moran, K., McCaffrey, N., Chouvarda, I., Walsh, D., Budts, W., Filos, D., Triantafyllidis, A., Maglaveras, N. & Cornelissen, V.A. (2017) 'PATHway I: design and rationale for the investigation of the feasibility, clinical effectiveness and cost-effectiveness of a technology-enabled cardiac rehabilitation platform'. *BMJ open* **7** (6), e016781-2017-016781

Clanchy, K.M., Tweedy, S.M. & Trost, S.G. (2019) 'The Adapted Physical Activity Program: A Theory-Driven, Evidence-Based Physical Activity Intervention for People with Brain Impairment.'. *Brain Impairment* **20** (1)

Clar, C., Waugh, N. & Thomas, S. (2007) 'Routine hospital admission versus out-patient or home care in children at diagnosis of type 1 diabetes mellitus'. *Cochrane Database of Systematic Reviews* (2)

Clark, C.E., Omboni, S., McDonagh, S.T., McManus, R.J. & Sheppard, J.P. (2020) 'Effective detection and management of hypertension through community pharmacy in England'. *Evaluation* **14**, 34

Clark, R.A., Inglis, S.C., Mcalister, F.A., Ball, J., Lewinter, C., Cullington, D., Stewart, S. & Cleland, J. (2010) 'Remote (non-invasive) monitoring in heart failure: effect on length of stay, quality of life, knowledge, adherance and satisfaction in 8,323 heart failure patients: a systematic review'. *European heart journal* **31**, 944-945

Clark, R.A., Inglis, S.C., Mcalister, F.A., Ball, J., Lewinter, C., Cullington, D., Stewart, S. & Cleland, J. (2010) 'Results from a systematic review and meta-analysis of remote (non-invasive) monitoring in 8,323 heart failure patients on length of stay, quality of life, knowledge, compliance and satisfaction'. *European journal of heart failure, supplement* **9**, S51-S52

Clark, R.A., Inglis, S.C., McAlister, F.A., Cleland, J.G.F. & Stewart, S. (2007) 'Telemonitoring or structured telephone support programmes for patients with chronic heart failure: systematic review and meta-analysis'. *BMJ* **334** (7600), 942

Clarke, J., Proudfoot, J. & Ma, H. (2016) 'Mobile phone and web-based cognitive behavior therapy for depressive symptoms and mental health comorbidities in people living with diabetes: results of a feasibility study'. *JMIR mental health* **3** (2), e5131

Clarke, M., Bratan, T., Kulkarni, S. & Jones, R. (2007) 'The impact of remote patient monitoring in managing silent myocardial infarction in a residential home setting.'. *Anadolu Kardiyoloji Dergisi* **7** (Suppl 1), 186-188

Clasen, T.F., Bostoen, K., Schmidt, W.P., Boisson, S., Fung, I., Jenkins, M.W., Scott, B., Sugden, S. & Cairncross, S. (2010) 'Interventions to improve disposal of human excreta for preventing diarrhoea'. *Cochrane Database of Systematic Reviews* (6)

Claudel, S.E., Tamura, K., Troendle, J., Andrews, M.R., Ceasar, J.N., Mitchell, V.M., Vijayakumar, N. & Powell-Wiley, T.M. (2020) 'Comparing Methods to Identify Wear-Time Intervals for Physical Activity With the Fitbit Charge 2'. *Journal of Aging and Physical Activity* **1** (aop), 1-7

Clevenger, K.A. (2015) *Impact of the Teaching HENRY (Healthy Exercise and Nutrition Recommendations for Youth) Intervention on Physical Activity Knowledge and Behavior in Rural Appalachia*

Cochrane, A., Furlong, M., McGilloway, S., Molloy, D.W., Stevenson, M. & Donnelly, M. (2016) 'Time-limited home-care reablement services for maintaining and improving the functional independence of older adults'. *Cochrane Database of Systematic Reviews* (10)

Coelho Filho, J. & Birks, J. (2001) 'Physostigmine for dementia due to Alzheimer's disease'. *Cochrane Database of Systematic Reviews* (2)

Conn, N.J., Schwarz, K.Q. & Borkholder, D.A. (2019) 'In-Home Cardiovascular Monitoring System for Heart Failure: Comparative Study'. *JMIR mHealth and uHealth* **7** (1), e12419

Conn, N.J., Schwarz, K.Q. & Borkholder, D.A. (2018) 'Nontraditional Electrocardiogram and Algorithms for Inconspicuous In-Home Monitoring: Comparative Study.'. *JMIR MHealth and UHealth* **6** (5), e120

Conway, A., Inglis, S.C. & Clark, R.A. (2014}) 'Effective Technologies for Noninvasive Remote Monitoring in Heart Failure'. *TELEMEDICINE AND E-HEALTH}* **20}** (6}), 531-538}

Cooney, G.M., Dwan, K., Greig, C.A., Lawlor, D.A., Rimer, J., Waugh, F.R., McMurdo, M. & Mead, G.E. (2013) 'Exercise for depression'. *Cochrane Database of Systematic Reviews* (9)

Cooper, C.B., Sirichana, W., Arnold, M.T., Neufeld, V., Eric, Taylor, M., Wang, X. & Dolezal, B.A. (2020}) 'Remote Patient Monitoring for the Detection of COPD Exacerbations'. *INTERNATIONAL JOURNAL OF CHRONIC OBSTRUCTIVE PULMONARY DISEASE}* **15}**, 2005-2013}

Corbett, J., d'Angelo, C., Gangitano, L. & Freeman, J. (2017) 'Future of Health'. *Findings from a Survey of Stakeholders on the Future of Health and Healthcare in England.Cambridge: RAND Europe*

Corder, K., Ekelund, U., Steele, R.M., Wareham, N.J. & Brage, S. (2008) 'Assessment of physical activity in youth'. *Journal of applied physiology*

Coren, E., Hossain, R., Pardo Pardo, J. & Bakker, B. (2016) 'Interventions for promoting reintegration and reducing harmful behaviour and lifestyles in street-connected children and young people'. *Cochrane Database of Systematic Reviews* (1)

Cornelius, C. (2018) 'A Classroom-based Physical Activity Intervention for Adolescents: Is There a Relationship with Self-efficacy, Physical Activity, and On-task Behavior?'.

Corral Gudino, L., Borao Cengotita-Bengoa, M., Jorge Sanchez, R.J. & Garcia Aparicio, J. (2017) '[The patient and the crossing between Primary and Hospital care. Systematic review of trials for the implementation of tools for integration in Spain]'. *Anales del Sistema Sanitario de Navarra* **40** (3), 443-459

Corry, M., Neenan, K., Brabyn, S., Sheaf, G. & Smith, V. (2019) 'Telephone interventions, delivered by healthcare professionals, for providing education and psychosocial support for informal caregivers of adults with diagnosed illnesses'. *Cochrane Database of Systematic Reviews* (5)

Cortez, N. (2013) 'The mobile health revolution'. *UCDL Rev.* **47**, 1173

Coto, J. (2020) 'Isolating Critical Components of a Pediatric Obesity Intervention: Does it Really Take a Village?'.

Coulter, A., Entwistle, V.A., Eccles, A., Ryan, S., Shepperd, S. & Perera, R. (2015) 'Personalised care planning for adults with chronic or long-term health conditions'. *Cochrane Database of Systematic Reviews* (3)

Coulter, E., Hasler, J., McLean, A., Allan, D. & Paul, L. (2015) 'An evaluation of web-based physio for people with spinal cord injury'. *Physiotherapy (united kingdom)* **101**, eS275-eS276

Cox, N.S., Dal Corso, S., Hansen, H., McDonald, C.F., Hill, C.J., Zanaboni, P., Alison, J.A., O'Halloran, P., Macdonald, H. & Holland, A.E. (2021) 'Telerehabilitation for chronic respiratory disease'. *Cochrane Database of Systematic Reviews* (1)

Cradock, A.L. & Duncan, D.T. (2013) 'The role of the built environment in supporting health behavior change'. *The handbook of health behavior change.4th ed.New York: Springer Publishing Company*

Cradock, A.L. & Duncan, D.T. (2013) 'The role of the built environment in supporting health behavior change'. *The handbook of health behavior change.4th ed.New York: Springer Publishing Company*

Crawford, R., Hughes, C., McFadden, S. & Crawford, J. (2021) 'A systematic review examining the clinical and health-care outcomes for congenital heart disease patients using home monitoring programmes.'. *Journal of Telemedicine & Telecare* **1357633X20984052**

Criscuoli de Farias, F.A., Dagostini, C.M., Bicca, Y.d.A., Falavigna, V.F. & Falavigna, A. (2020}) 'Remote Patient Monitoring: A Systematic Review'. *TELEMEDICINE AND E-HEALTH}* **26}** (5}), 576-583}

Cronin, E.M., Ching, E.A., Varma, N., Martin, D.O., Wilkoff, B.L. & Lindsay, B.D. (2012) 'Remote monitoring of cardiovascular devices: a time and activity analysis.'. *Heart Rhythm* **9** (12), 1947-1951

Cross, A.J., Elliott, R.A., Petrie, K., Kuruvilla, L. & George, J. (2020) 'Interventions for improving medication-taking ability and adherence in older adults prescribed multiple medications'. *Cochrane Database of Systematic Reviews* (5)

Crossley, G., Boyle, A., Vitense, H., Sherfesee, L. & Mead, R.H. (2008) 'Trial design of the clinical evaluation of remote notification to reduce time to clinical decision: the Clinical evaluation Of remote NotificatioN to rEduCe Time to clinical decision (CONNECT) study'. *American Heart Journal* **156** (5), 840-846

Cruz, A.M., Monsalve, L., Ladurner, A., Jaime, L.F., Wang, D. & Quiroga, D.A. (2021) 'Information and Communication Technologies for Managing Frailty: A Systematic Literature Review'. *Aging and disease* **12** (3), 914

Cruz-Martinez, R.R., Noort, P.D., Asbjornsen, R.A., van Niekerk, J.M., Wentzel, J., Sanderman, R. & van Gemert-Pijnen, L. (2019) 'Frameworks, Models, and Theories Used in Electronic Health Research and Development to Support Self-Management of Cardiovascular Diseases Through Remote Monitoring Technologies: Protocol for a Metaethnography Review.'. *JMIR Research Protocols* **8** (7), e13334

CTRI/2015/12/006424, (2015) 'A community-based programme for reducing the burden of stroke in rural Gadchiroli, Maharashtra, India'. *http://www.who.int/trialsearch/Trial2.aspx?TrialID=CTRI/2015/12/006424*

Cueto, V. & Sanders, L.M. (2020) 'Telehealth Opportunities and Challenges for Managing Pediatric Obesity'. *Pediatric clinics of North America* **67** (4), 647-654

Cui, Y., Ji, D., Maenhaut, W., Gao, W., Zhang, R. & Wang, Y. (2020) 'Levels and sources of hourly $PM_{2.5}$-related elements during the control period of the COVID-19 pandemic at a rural site between Beijing and Tianjin.'. *Science of the Total Environment* **744**, 140840

Cummings, D.M., Adams, A., Halladay, J., Hinderliter, A., Donahue, K.E., Cene, C.W., Li, Q., Miller, C., Garcia, B., Tillman, J., Little, E. & DeWalt, D. (2019) 'Race-Specific Patterns of Treatment Intensification Among Hypertensive Patients Using Home Blood Pressure Monitoring: Analysis Using Defined Daily Doses in the Heart Healthy Lenoir Study'. *Annals of Pharmacotherapy* **53** (4), 333-340

Cummings, D.M., Adams, A., Halladay, J., Hinderliter, A., Donahue, K.E., Cene, C.W., Li, Q., Miller, C., Garcia, B., Tillman, J., Little, E. & DeWalt, D. (2019) 'Race-Specific Patterns of Treatment Intensification Among Hypertensive Patients Using Home Blood Pressure Monitoring: Analysis Using Defined Daily Doses in the Heart Healthy Lenoir Study.'. *Annals of Pharmacotherapy* **53** (4), 333-340

Curb, J.D., Ceria-Ulep, C.D., Rodriguez, B.L., Grove, J., Guralnik, J., Willcox, B.J., Donlon, T.A., Masaki, K.H. & Chen, R. (2006) 'Performance-based measures of physical function for high-function populations'. *Journal of the American Geriatrics Society* **54** (5), 737-742

Curran, M., Tierney, A.C., Collins, L., Kennedy, L., McDonnell, C., Jurascheck, A.J., Sheikhi, A., Walsh, C., Button, B. & Galvin, R. (2020) 'Steps Ahead: optimising physical activity in adults with cystic fibrosis: Study Protocol for a pilot randomised trial using wearable technology, goal setting and text message feedback.'. *HRB Open Research* **3**

Curtis, R.G., Olds, T., Fraysse, F., Dumuid, D., Hendrie, G.A., Esterman, A., Brown, W.J., Ferguson, T., Lagiseti, R. & Maher, C.A. (2021) 'Annual rhythms in adults' lifestyle and health (ARIA): protocol for a 12-month longitudinal study examining temporal patterns in weight, activity, diet, and wellbeing in Australian adults'. *BMC Public Health* **21** (1), 1-8

Custis, L.M., Hawkins, S.Y. & Thomason, T.R. (2017) 'An innovative capstone health care informatics clinical residency: Interprofessional team collaboration.'. *Health Informatics Journal* **23** (1), 69-79

Dadosky, A., Overbeck, H., Barbetta, L., Bertke, K., Corl, M., Daly, K., Hiles, N., Rector, N., Chung, E. & Menon, S. (2018) 'Telemanagement of Heart Failure Patients Across the Post-Acute Care Continuum.'. *Telemedicine Journal & E-Health* **24** (5), 360-366

Dagan A. & Mechanic, O.J. (2020) 'Use of ultra-low cost fitness trackers as clinical monitors in low resource emergency departments.'. *Clinical and Experimental Emergency Medicine* **7** (3), 144-149

Dahlke, D.V., Lee, S., Smith, M.L., Shubert, T., Popovich, S. & Ory, M.G. (2021) 'Attitudes toward technology and use of fall alert wearables in caregiving: survey study'. *JMIR aging* **4** (1), e23381

Dailloux, Y., Farenc, M., Piocelle, M. & Billonnet, L. (2015) 'An Innovative Technico-Medico-Social Structure and Platform for Services to Elderly'. *Med-e-Tel 2015* , 26

Dalleck, L.C., Schmidt, L.K. & Lueker, R. (2011) 'Cardiac rehabilitation outcomes in a conventional versus telemedicine-based programme.'. *Journal of Telemedicine & Telecare* **17** (5), 217-221

Dallery, J., Kurti, A. & Erb, P. (2015) 'A new frontier: Integrating behavioral and digital technology to promote health behavior'. *The Behavior Analyst* **38** (1), 19-49

Dang, S., Dimmick, S. & Kelkar, G. (2009) 'Evaluating the evidence base for the use of home telehealth remote monitoring in elderly with heart failure'. *Telemedicine Journal & E-Health* **15** (8), 783-796

Daniels, B., Greenwald, P., Hsu, H., Steel, P., Hafeez, B., Watts, B., Goyal, P., Horn, E. & Sharma, R. (2019) '284 Using Community Tele-Paramedicine to Reduce Unnecessary Emergency Department Visits and 30-Day Readmissions Among High-Risk Patients With Heart Failure'. *Annals of Emergency Medicine* **74** (4), S112-S113

Daniels, S.R., Pratt, C.A., Hollister, E.B., Labarthe, D., Cohen, D.A., Walker, J.R., Beech, B.M., Balagopal, P.B., Beebe, D.W., Gillman, M.W., Goodrich, J.M., Jaquish, C., Kit, B., Miller, A.L., Olds, D., Oken, E., Rajakumar, K., Sherwood, N.E., Spruijt-Metz, D., Steinberger, J., Suglia, S.F., Teitelbaum, S.L., Urbina, E.M., Van Horn, L., Ward, D. & Young, M.E. (2019) 'Promoting Cardiovascular Health in Early Childhood and Transitions in Childhood through Adolescence: A Workshop Report'. *The Journal of pediatrics* **209**, 240-251.e1

Dario, C., Delise, P., Gubian, L., Saccavini, C., Brandolino, G. & Mancin, S. (2016) 'Large Controlled Observational Study on Remote Monitoring of Pacemakers and Implantable Cardiac Defibrillators: A Clinical, Economic, and Organizational Evaluation.'. *Interactive Journal of Medical Research* **5** (1), e4

Das, J.K., Hoodbhoy, Z., Salam, R.A., Bhutta, A.Z., Valenzuela-Rubio, N., Weise Prinzo, Z. & Bhutta, Z.A. (2018) 'Lipid-based nutrient supplements for maternal, birth, and infant developmental outcomes'. *Cochrane Database of Systematic Reviews* (8)

Dash, S.P. (2020) 'The Impact of IoT in Healthcare: Global Technological Change & The Roadmap to a Networked Architecture in India'. *Journal of the Indian Institute of Science* , 1-13

Dator, R.P., Solivio, M.J., Villalta, P.W. & Balbo, S. (2019) 'Bioanalytical and Mass Spectrometric Methods for Aldehyde Profiling in Biological Fluids'. *Toxics* **7** (2)

Datta Burton, S., Tanczer, L.M., Vasudevan, S., Hailes, S. & Carr, M. (2021) 'The UK Code of Practice for Consumer IoT Cybersecurity: where we are and what next'.

Datta, S., Barua, R. & Das, J. (2020) 'Application of artificial intelligence in modern healthcare system'. *Alginates—recent uses of this natural polymer*

Dave, G., Hoover, S., Frerichs, L., Hamilton, M., Bess, K., Jessup, H.R., Boone, B., Wynn, M., McFarlin, S. & Corbie-Smith, G. (2019) 'Using community engagement to adapt and imple-ment an evidence-based CVD intervention in rural african american communities'. *Journal of general internal medicine* **34** (2), S399-

Davila, M.I., Kizakevich, P.N., Eckhoff, R., Morgan, J., Meleth, S., Ramirez, D., Morgan, T., Strange, L.B., Lane, M., Weimer, B., Lewis, A., Lewis, G.F. & Hourani, L.I. (2021) 'Use of Mobile Technology Paired with Heart Rate Monitor to Remotely Quantify Behavioral Health Markers among Military Reservists and First Responders'. *Military medicine* **186**, 17-24

Davis, C., Bender, M., Smith, T. & Broad, J. (2015) 'Feasibility and Acute Care Utilization Outcomes of a Post-Acute Transitional Telemonitoring Program for Underserved Chronic Disease Patients.'. *Telemedicine Journal & E-Health* **21** (9), 705-713

de Asmundis, C., Ricciardi, D., Namdar, M., Chierchia, G., Sarkozy, A. & Brugada, P. (2013) 'Role of home monitoring in children with implantable cardioverter defibrillators for Brugada syndrome.'. *Europace* **15** (Suppl 1), 17-25

De Asmundis, C., Ricciardi, D., Namdar, M., Pappaert, G., Rodriguez-Manero, M., Wauters, K., Casado-Arroyo, R., Rao, J.J., Bayrak, F., Chierchia, G., Sarkozy, A. & Brugada, P. (2013) 'ICD function and dysfunction in patients with arrhythmogenic cardiac diseases: the role of home monitoring.'. *Acta Cardiologica* **68** (4), 387-394

De Capua, C., De Falco, S. & Morello, R. (2006}) 'A soft computing-based measurement system for medical applications in diagnosis of cardiac arrhythmias by ECG signals analysis'. In Anonymous (eds.) *PROCEEDINGS OF THE 2006 IEEE INTERNATIONAL CONFERENCE ON COMPUTATIONAL INTELLIGENCE FOR MEASUREMENT SYSTEMS AND APPLICATIONS},* Held 345 E 47TH ST, NEW YORK, NY 10017 USA}: IEEE}, 2}

de Diego, C., Gonzalez-Torres, L., Nunez, J.M., Centurion Inda, R., Martin-Langerwerf, D.A., Sangio, A.D., Chochowski, P., Casasnovas, P., Blazquez, J.C. & Almendral, J. (2018) 'Effects of angiotensin-neprilysin inhibition compared to angiotensin inhibition on ventricular arrhythmias in reduced ejection fraction patients under continuous remote monitoring of implantable defibrillator devices.'. *Heart Rhythm* **15** (3), 395-402

de Jongh, T., Gurol-Urganci, I., Vodopivec-Jamsek, V., Car, J. & Atun, R. (2012) 'Mobile phone messaging for facilitating self-management of long-term illnesses'. *Cochrane Database of Systematic Reviews* (12)

De La Torre, S., Spruijt-Metz, D. & Farias, A.J. (2021) 'Associations Among Wearable Activity Tracker Use, Exercise Motivation, and Physical Activity in a Cohort of Cancer Survivors: Secondary Data Analysis of the Health Information National Trends Survey'. *JMIR cancer* **7** (2), e24828

de Ruvo, E., Sciarra, L., Martino, A.M., Rebecchi, M., Iulianella, R.V., Sebastiani, F., Fagagnini, A., Borrelli, A., Scara, A., Grieco, D., Tota, C., Stirpe, F. & Calo, L. (2016) 'A prospective comparison of remote monitoring systems in implantable cardiac defibrillators: potential effects of frequency of transmissions.'. *Journal of Interventional Cardiac Electrophysiology* **45** (1), 81-90

de Silva, C.W., Xiao, S., Li, M. & de Silva, C.N. (2013) 'TELEMEDICINE–REMOTE SENSORY INTERACTION WITH PATIENTS FOR MEDICAL EVALUATION AND DIAGNOSIS'. *Control and Intelligent Systems* **41** (4)

De Simone, V., Guarise, P., Guardalben, S., Padovani, N., Tondelli, S., Sandrini, D., Visentin, E. & Zanotto, G. (2020) 'Telecardiology during the Covid-19 pandemic: past mistakes and future hopes'. *American Journal of Cardiovascular Disease* **10** (2), 34-47

DeAlleaume, L., Parnes, B., Zittleman, L., Sutter, C., Chavez, R., Bernstein, J., LeBlanc, W., Dickinson, M. & Westfall, J.M. (2015) 'Success in the Achieving CARdiovascular Excellence in Colorado (A CARE) Home Blood Pressure Monitoring Program: A Report from the Shared Networks of Colorado Ambulatory Practices and Partners (SNOCAP).'. *Journal of the American Board of Family Medicine: JABFM* **28** (5), 548-555

Dedov, V.N. & Dedova, I.V. (2015) 'Automated Management of Exercise Intervention at the Point of Care: Application of a Web-Based Leg Training System'. *JMIR rehabilitation and assistive technologies* **2** (2), e11

Dedov, V.N. & Dedova, I.V. (2015) 'Development of the Internet-Enabled System for Exercise Telerehabilitation and Cardiovascular Training'. *Telemedicine journal and e-health : the official journal of the American Telemedicine Association* **21** (7), 575-580

Dedov, V.N. & Dedova, I.V. (2016) 'Application of a Web-Enabled Leg Training System for the Objective Monitoring and Quantitative Analysis of Exercise-Induced Fatigue.'. *JMIR Research Protocols* **5** (3), e171

Dedov, V.N. & Dedova, I.V. (2015) 'Automated Management of Exercise Intervention at the Point of Care: Application of a Web-Based Leg Training System.'. *JMIR Rehabilitation And Assistive Technologies* **2** (2), e11

Deepak, K. (2013) 'ICT for elderly health and independent living-opportunities & challenges'.

Delaney, C. & Apostolidis, B. (2010) 'Pilot testing of a multicomponent home care intervention for older adults with heart failure: an academic clinical partnership'. *Journal of Cardiovascular Nursing* **25** (5), E27-40

Dellasega, C., Orwig, D., Ahern, F. & Lenz, E. (1999) 'Postdischarge medication use of elderly cardiac patients from urban and rural locations.'. *Journals of Gerontology Series A-Biological Sciences & Medical Sciences* **54** (10), M514-20

Delva, S. (2020) *Adoption of mHealth Among Latinos with Cardiometabolic Risk Factors*

Delva, S., Waligora Mendez, K.J., Cajita, M., Koirala, B., Shan, R., Wongvibulsin, S., Vilarino, V., Gilmore, D.R. & Han, H.R. (2021) 'Efficacy of Mobile Health for Self-management of Cardiometabolic Risk Factors: A Theory-Guided Systematic Review'. *The Journal of cardiovascular nursing* **36** (1), 34-55

Dennis, M., Smith, K.A., Grasso, F. & Paris, C. '2015 International Workshop on Personalisation and Adaptation in Technology for Health'.

Depledge, M.H., Stone, R.J. & Bird, W. (2011) 'Can natural and virtual environments be used to promote improved human health and wellbeing?'. *Environmental science & technology* **45** (11), 4660-4665

De-Regil, L., Jefferds, M. & Peña-Rosas, J. (2017) 'Point-of-use fortification of foods with micronutrient powders containing iron in children of preschool and school-age'. *Cochrane Database of Systematic Reviews* (11)

Deshpande, A., Mathur, A. & Krishnamurthy, S. (2016) 'Application of Internet of Things in healthcare sector for bottom of pyramid in India'. *International Journal of Engineering Applied Sciences and Technology* **1** (11), 28-33

Desjardins, D. (2014) 'Remote monitoring exploring new territories'. *Medicine on the Net* **20** (4), 1-3

Di Figlia-Peck, S., Feinstein, R. & Fisher, M. (2020) 'Treatment of children and adolescents who are overweight or obese'. *Current Problems in Pediatric and Adolescent Health Care*

Di Lenarda, A., Casolo, G., Gulizia, M.M., Aspromonte, N., Scalvini, S., Mortara, A., Alunni, G., Ricci, R.P., Mantovan, R., Russo, G., Gensini, G.F. & Romeo, F. (2017) 'The future of telemedicine for the management of heart failure patients: a Consensus Document of the Italian Association of Hospital Cardiologists (A.N.M.C.O), the Italian Society of Cardiology (S.I.C.) and the Italian Society for Telemedicine and eHealth (Digital S.I.T.).'. *European Heart Journal Supplements* **19** (Suppl D), 113-129

Di Lenarda, A., Casolo, G., Gulizia, M.M., Aspromonte, N., Scalvini, S., Mortara, A., Alunni, G., Ricci, R.P., Mantovan, R., Russo, G., Gensini, G.F., Romeo, F. & ANMCO/SIC/SIT (2016) '[ANMCO/SIC/SIT Consensus document: The future of telemedicine in heart failure]'. *Giornale italiano di cardiologia* **17** (6), 491-507

Di Pietrantonj, C., Rivetti, A., Marchione, P., Debalini, M.G. & Demicheli, V. (2020) 'Vaccines for measles, mumps, rubella, and varicella in children'. *Cochrane Database of Systematic Reviews* (4)

Di Rienzo, M., Rizzo, F., Parati, G., Ferratini, M., Brambilla, G. & Castiglioni, P. (2005}) 'A textile-based wearable system for vital sign monitoring: Applicability in cardiac patients'. In Anonymous (eds.) *COMPUTERS IN CARDIOLOGY 2005, VOL 32},* Held 345 E 47TH ST, NEW YORK, NY 10017 USA}: IEEE}, 699-701}

Di Rienzo, M., Rizzo, F., Parati, G., Brambilla, G., Ferratini, M. & Castiglioni, P. (2005) 'MagIC System: a New Textile-Based Wearable Device for Biological Signal Monitoring. Applicability in Daily Life and Clinical Setting.'. *Conference Proceedings: ...Annual International Conference of the IEEE Engineering in Medicine & Biology Society* **2005**, 7167-7169

Diaz-Skeete, Y., Giggins, O.M., McQuaid, D. & Beaney, P. (2020) 'Enablers and obstacles to implementing remote monitoring technology in cardiac care: A report from an interactive workshop'. *Health Informatics Journal* **26** (3), 2280-2288

Dickerson, J.A., Sinkey, M., Jacot, K., Stack, J., Sadilkova, K., Law, Y.M. & Jack, R.M. (2015) 'Tacrolimus and sirolimus in capillary dried blood spots allows for remote monitoring.'. *Pediatric transplantation* **19** (1), 101-106

Dierckx, R., Pellicori, P., Cleland, J.G.F. & Clark, A.L. (2015) 'Telemonitoring in heart failure: Big Brother watching over you'. *Heart failure reviews* **20** (1), 107-116

Dietrich, D., Dekova, R., Davy, S., Fahrni, G. & Geissbühler, A. (2018) 'Applications of space technologies to global health: scoping review'. *Journal of medical Internet research* **20** (6), e230

Dimengo, J. & Stegall, G. (2015}) 'Team-Based Care for External Telemonitoring in Patients with Heart Failure'. *HEART FAILURE CLINICS}* **11}** (3}), 451}

Dinesen, B. & Spindler, H. (2018) 'The use of telerehabilitation technologies for cardiac patients to improve rehabilitation activities and unify organizations: qualitative study'. *JMIR rehabilitation and assistive technologies* **5** (2), e10758

Ding, E.Y., Ensom, E., Hafer, N., Buchholz, B., Picard, M.A., Dunlap, D., Rogers, E., Lawton, C., Koren, A. & Lilly, C. (2019) 'Point-of-care technologies in heart, lung, blood and sleep disorders from the Center for Advancing Point-of-Care Technologies'. *Current opinion in biomedical engineering* **11**, 58-67

Ding, H., Jayasena, R., Maiorana, A., Dowling, A., Chen, S.H., Karunanithi, M., Layland, J. & Edwards, I. (2017) 'Innovative Telemonitoring Enhanced Care Programme for Chronic Heart Failure (ITEC-CHF) to improve guideline compliance and collaborative care: protocol of a multicentre randomised controlled trial'. *BMJ open* **7** (10), e017550

Dobbins, M., Husson, H., DeCorby, K. & LaRocca, R.L. (2013) 'School-based physical activity programs for promoting physical activity and fitness in children and adolescents aged 6 to 18'. *Cochrane Database of Systematic Reviews* (2)

Doering, L.V., Hickey, K., Pickham, D., Chen, B. & Drew, B.J. (2012) 'Remote noninvasive allograft rejection monitoring for heart transplant recipients: study protocol for the novel evaluation with home electrocardiogram and remote transmission (NEW HEART) study.'. *BMC Cardiovascular Disorders* **12**, 14

Dolezal, B.A., Abrazado, M., Batalin, M.A., Smith, D. & Cooper, C.B. (2014) 'Deployment of remote advanced electrocardiography for improved cardiovascular risk assessment in career firefighters.'. *Telemedicine Journal & E-Health* **20** (7), 660-663

Dominey, T. (2019) *Evaluating Digital Health Technologies to Advance Parkinson's Disease Care*

Domingo, M., Lupón, J., González, B., Crespo, E., López, R., Ramos, A., Urrutia, A., Pera, G., Verdú, J. & Bayes-Genis, A. (2011) 'Noninvasive remote telemonitoring for ambulatory patients with heart failure: effect on number of hospitalizations, days in hospital, and quality of life. CARME (CAtalan Remote Management Evaluation) study'. *Revista espanola de cardiologia* **64** (4), 277-285

Domingo, M., Lupon, J., Gonzalez, B., Crespo, E., Lopez, R., Ramos, A., Urrutia, A., Pera, G., Verdu, J.M. & Bayes-Genis, A. (2012}) 'Evaluation of a telemedicine system for heart failure patients: feasibility, acceptance rate, satisfaction and changes in patient behavior: results from the CARME (CAtalan Remote Management Evaluation) study'. *EUROPEAN JOURNAL OF CARDIOVASCULAR NURSING}* **11}** (4}), 410-418}

Donald, M., Beanlands, H., Straus, S.E., Smekal, M., Gil, S., Elliott, M.J., Herrington, G., Harwood, L., Waldvogel, B. & Delgado, M. (2021) 'A web-based self-management support prototype for adults with chronic kidney disease (my kidneys my health): Co-design and usability testing'. *JMIR formative research* **5** (2), e22220

D'Onofrio, A., Solimene, F., Calo, L., Calvi, V., Viscusi, M., Melissano, D., Russo, V.A., Rapacciuolo, A., Campana, A. & Bonfanti, P. (2019) 'Combining home monitoring temporal trends and baseline patient risk profile for predicting impending heart failure hospitalizations. Results from the SELENE HF (BIO.Detect HF IV) study'. *European heart journal* **40**, 1552-

Doraiswamy, P.M., London, E., Varnum, P., Harvey, B., Saxena, S., Tottman, S., Campbell, P., Ibáñez, A.F., Manji, H. & Al Olama, Mohammad Abdul Aziz Sultan (2019) 'Empowering 8 billion minds: enabling better mental health for all via the ethical adoption of technologies'. *NAM Perspectives*

Dor-Haim, H., Katzburg, S. & Leibowitz, D. (2019) 'A Novel Digital Platform for a Monitored Home-based Cardiac Rehabilitation Program'. *Journal of visualized experiments : jove* (146)

Dorje, T., Zhao, G., Scheer, A., Tsokey, L., Wang, J., Chen, Y., Tso, K., Tan, B.K., Ge, J. & Maiorana, A. (2018) 'SMARTphone and social media-based Cardiac Rehabilitation and Secondary Prevention (SMART-CR/SP) for patients with coronary heart disease in China: a randomised controlled trial protocol'. *BMJ open* **8** (6), e021908-2018-021908

Dorsey, E.R., Glidden, A.M., Holloway, M.R., Birbeck, G.L. & Schwamm, L.H. (2018) 'Teleneurology and mobile technologies: the future of neurological care'. *Nature Reviews Neurology* **14** (5), 285

Dorsey, E.R. & Topol, E.J. (2016) 'State of telehealth'. *New England Journal of Medicine* **375** (2), 154-161

Douglas-Oguneko, C. (2018) *Perceptions of Health Providers about the Collection and Use of Data Related to Childhood Obesity by Mobile Health Units*

Dovancescu, S., Saczynski, J.S., Darling, C.E., Riistama, J., Sert Kuniyoshi, F., Meyer, T., Goldberg, R. & McManus, D.D. (2015) 'Detecting Heart Failure Decompensation by Measuring Transthoracic Bioimpedance in the Outpatient Setting: Rationale and Design of the SENTINEL-HF Study.'. *JMIR Research Protocols* **4** (4), e121

Downey, C., Tahir, W., Randell, R., Brown, J. & Jayne, D. (2017) 'Strengths and limitations of early warning scores: a systematic review and narrative synthesis'. *International journal of nursing studies* **76**, 106-119

Dowswell, T., Carroli, G., Duley, L., Gates, S., Gülmezoglu, A., Khan-Neelofur, D. & Piaggio, G. (2015) 'Alternative versus standard packages of antenatal care for low-risk pregnancy'. *Cochrane Database of Systematic Reviews* (7)

Drahota, A., Ward, D., Mackenzie, H., Stores, R., Higgins, B., Gal, D. & Dean, T.P. (2012) 'Sensory environment on health-related outcomes of hospital patients'. *Cochrane Database of Systematic Reviews* (3)

Drewnowski, A., Buszkiewicz, J., Aggarwal, A., Rose, C., Gupta, S. & Bradshaw, A. (2020) 'Obesity and the Built Environment: A Reappraisal'. *Obesity (19307381)* **28** (1), 22-30

Duby, J., Lassi, Z.S. & Bhutta, Z.A. (2019) 'Community-based antibiotic delivery for possible serious bacterial infections in neonates in low- and middle-income countries'. *Cochrane Database of Systematic Reviews* (4)

Duck A.A., Hall K.C., Klamm M., Temple M. & Robinson, J.C. (2021) 'Physical activity and fitness: The feasibility and preliminary effectiveness of wearable activity tracker technology incorporating altruistic motivation in youth.'. *Journal for Specialists in Pediatric Nursing* **26** (1) (pagination), Arte Number: e12313. ate of Pubaton: January 2021

Dunn, J., Runge, R. & Snyder, M. (2018) 'Wearables and the medical revolution'. *Personalized medicine* **15** (5), 429-448

Dunn, J.T. (2017) *Data Integration for Health and Stress Monitoring: Biological Metabolites, Wearables Data, and Self-Reporting*

Dunstan, D.W., Wiesner, G., Eakin, E.G., Neuhaus, M., Owen, N., LaMontagne, A.D., Moodie, M., Winkler, E.A., Fjeldsoe, B.S. & Lawler, S. (2013) 'Reducing office workers' sitting time: rationale and study design for the Stand Up Victoria cluster randomized trial'. *BMC public health* **13** (1), 1-14

Duran-Vega, L.A., Santana-Mancilla, P.C., Buenrostro-Mariscal, R., Contreras-Castillo, J., Anido-Rifon, L.E., Garcia-Ruiz, M.A., Montesinos-Lopez, O.A. & Estrada-Gonzalez, F. (2019) 'An IoT System for Remote Health Monitoring in Elderly Adults Through a Wearable Device and Mobile Application.'. *Geriatrics* **4** (2)

Ebert, D.D., Van Daele, T., Nordgreen, T., Karekla, M., Compare, A., Zarbo, C., Brugnera, A., Øverland, S., Trebbi, G. & Jensen, K.L. (2018) 'Internet-and mobile-based psychological interventions: applications, efficacy, and potential for improving mental health'. *European Psychologist*

Edirippulige, S., Armfielf, N.R., Bensink, M.E., Marasinghe, R. & Wootton, R. (2008) 'Preliminary evaluation of a practical program in e-health education'. *Telemedicine and e-Health* **14** (s1), 111-111

Effken, J.A. & Abbott, P. (2009) 'Health IT-enabled care for underserved rural populations: The role of nursing'. *Journal of the American Medical Informatics Association* **16** (4), 439-445

Ehlers, D.K. & Fanning, J. (2019) 'Strategies for promoting physical activity in middle-aged and older adults.'.

Ehn, M., Johansson, A. & Revenäs, Å. (2019) 'Technology-based motivation support for seniors' physical activity—A qualitative study on seniors' and health care professionals' views'. *International journal of environmental research and public health* **16** (13), 2418

Eisner, E., Drake, R.J., Berry, N., Barrowclough, C., Emsley, R., Machin, M. & Bucci, S. (2019) 'Development and long-term acceptability of ExPRESS, a mobile phone app to monitor basic symptoms and early signs of psychosis relapse'. *JMIR mHealth and uHealth* **7** (3), e11568

Ejofodomi, O., Zara, J. & Ofualagba, G. (2020) 'MEDLINK: a low-cost, portable, verbally interactive and programmable remote patient monitoring (RPM) device'. *Practical Neurology (BMJ Publishing Group)* **20** (5), 151-158

El Amrani, L., Engberink, A.O., Ninot, G., Hayot, M. & Carbonnel, F. (2017) 'Connected health devices for health care in French general medicine practice: cross-sectional study'. *JMIR mHealth and uHealth* **5** (12), e193

El Oualid Amara, M., Montagnier, C., Cheggour, S., Boursier, M., Gully, C., Barnay, C., Georger, F., Deplagne, A., Fromentin, S. & Mlotek, M. (2015) 'Early detection and treatment of supraventricular arrhythmia with remote monitoring can prevent its progression in pacemaker patients: the randomized, multicenter SETAM trial'. *Journal of the american college of cardiology.* **65** (10), A388

Elgendi, M., Howard, N., Lovell, N., Cichocki, A., Brearley, M., Abbott, D. & Adatia, I. (2016) 'A six-step framework on biomedical signal analysis for tackling noncommunicable diseases: Current and future perspectives'. *JMIR Biomedical Engineering* **1** (1), e1

Elgendi, M., Al-Ali, A., Mohamed, A. & Ward, R. (2018) 'Improving Remote Health Monitoring: A Low-Complexity ECG Compression Approach.'. *Diagnostics* **8** (1)

El-Sappagh, S., Ali, F., El-Masri, S., Kim, K., Ali, A. & Kwak, K. (2018) 'Mobile health technologies for diabetes mellitus: current state and future challenges'. *IEEE Access* **7**, 21917-21947

El-Sappagh, S., Ali, F., Hendawi, A., Jang, J. & Kwak, K. (2019) 'A mobile health monitoring-and-treatment system based on integration of the SSN sensor ontology and the HL7 FHIR standard'. *BMC medical informatics and decision making* **19** (1), 97

Emami-Abarghouei, B. (2016) *Self-coach: an intelligent WBAN system for heart disease prediction using non-dominated sorting genetic algorithm*

Epstein, E., Patel, N., Maysent, K. & Taub, P.R. (2021) 'Cardiac Rehab in the COVID Era and Beyond: mHealth and Other Novel Opportunities'. *Current cardiology reports* **23** (5), 42

Eriksson, P.G. (2020) 'Analysis of Physiotherapists Perceptions for Improvement of Digital Innovation'.

Esakia, A. (2017) *Development and Exploratory Findings of a Smartwatch Interface to Facilitate Group Cohesion in a Statewide Health Promotion Program*

Escolar Pérez, V. (2020) 'Prediction Of Heart Failure Decompensations Using Artificial Intelligence-Machine Learning Techniques'.

Eshun-Wilson, I., Siegfried, N., Akena, D.H., Stein, D.J., Obuku, E.A. & Joska, J.A. (2018) 'Antidepressants for depression in adults with HIV infection'. *Cochrane Database of Systematic Reviews* (1)

Espinosa, Á.V., López, J.L.L., Mata, F.M. & Estevez, M.E.E. (2021) 'Application of IoT in Healthcare: Keys to Implementation of the Sustainable Development Goals'. *Sensors* **21** (7), 2330

Estrada-Codecido, J.M. (2020) *Use of Activity Trackers to Count Steps of the Older Emergency Department Patient: A Feasibility, Validity and Reliability Study.*

Etminani, K., Engström, A.T., Göransson, C., Sant'Anna, A. & Nowaczyk, S. (2020) 'How Behavior Change Strategies are Used to Design Digital Interventions to Improve Medication Adherence and Blood Pressure Among Patients With Hypertension: Systematic Review'. *Journal of medical Internet research* **22** (4), e17201

Etminani, K., Göransson, C., Galozy, A., Pejner, M.N. & Nowaczyk, S. (2021) 'Improving Medication Adherence Through Adaptive Digital Interventions (iMedA) in Patients With Hypertension: Protocol for an Interrupted Time Series Study'. *JMIR Research Protocols* **10** (5), e24494

Evangelista, L.S., Jung-Ah Lee, Moore, A.A., Motie, M., Ghasemzadeh, H., Sarrafzadeh, M. & Mangione, C.M. (2015) 'Examining the Effects of Remote Monitoring Systems on Activation, Self-care, and Quality of Life in Older Patients With Chronic Heart Failure'. *Journal of Cardiovascular Nursing* **30** (1), 51-57

Evans, J., Papadopoulos, A., Silvers, C.T., Charness, N., Boot, W.R., Schlachta-Fairchild, L., Crump, C., Martinez, M. & Ent, C.B. (2016) 'Remote Health Monitoring for Older Adults and Those with Heart Failure: adherence and System Usability'. *Telemedicine journal and e-health* **22** (6), 480-488

Eysenbach, G. & Jadad, A.R. (2001) 'Evidence-based patient choice and consumer health informatics in the Internet age'. *Journal of medical Internet research* **3** (2), e19

Fagherazzi, G., Fischer, A., Ismael, M. & Despotovic, V. (2021) 'Voice for Health: The Use of Vocal Biomarkers from Research to Clinical Practice'. *Digital Biomarkers* **5** (1), 78-88

Fairbrother, P., Ure, J., Hanley, J., McCloughan, L., Denvir, M., Sheikh, A., McKinstry, B. & Telescot programme team (2014) 'Telemonitoring for chronic heart failure: the views of patients and healthcare professionals - a qualitative study.'. *Journal of Clinical Nursing* **23** (1-2), 132-144

Falcão Duarte, C. (2019) 'Insight. me: Enabling a collaborative use of healthcare data'.

Fang, J., Huang, B., Xu, D., Li, J. & Au, W.W. (2019) 'Innovative Application of a Home-Based and Remote Sensing Cardiac Rehabilitation Protocol in Chinese Patients After Percutaneous Coronary Intervention'. *Telemedicine journal and e-health* **25** (4), 288-293

Fang, J., Li, J., Li, Z., Xu, D., Chen, C., Xie, B., Chen, H. & Au, W.W. (2016) 'Attitudes towards acceptance of an innovative home-based and remote sensing rehabilitation protocol among cardiovascular patients in Shantou, China.'. *Journal of Geriatric Cardiology* **13** (4), 326-332

Fang, W., Song, W., Liu, L., Chen, G., Ma, L., Liang, Y., Xu, Y., Wang, X., Ji, Y., Zhuang, Y., Boubacar, A.H. & Li, Y. (2020) 'Characteristics of indoor and outdoor fine particles in heating period at urban, suburban, and rural sites in Harbin, China.'. *Environmental Science & Pollution Research* **27** (2), 1825-1834

Fanucci, L., Saponara, S., Bacchillone, T., Donati, M., Barba, P., Sanchez-Tato, I. & Carmona, C. (2013}) 'Sensing Devices and Sensor Signal Processing for Remote Monitoring of Vital Signs in CHF Patients'. *IEEE TRANSACTIONS ON INSTRUMENTATION AND MEASUREMENT}* **62}** (3}), 553-569}

Faragli, A., Abawi, D., Quinn, C., Cvetkovic, M., Schlabs, T., Tahirovic, E., Düngen, H., Pieske, B., Kelle, S. & Edelmann, F. (2020) 'The role of non-invasive devices for the telemonitoring of heart failure patients'. *Heart failure reviews* , 1-18

Farias, F.A.C.d., Dagostini, C.M., Bicca, Y.d.A., Falavigna, V.F. & Falavigna, A. (2020) 'Remote Patient Monitoring: A Systematic Review.'. *Telemedicine Journal & E-Health* **26** (5), 576-583

Farjadian, A.B., Sivak, M.L. & Mavroidis, C. (2013) 'SQUID: sensorized shirt with smartphone interface for exercise monitoring and home rehabilitation.'. *IEEE International Conference on Rehabilitation Robotics* **2013**, 6650451

Farmer, O., Cahill, K. & O'Brien, W. (2020) 'Gaelic4Girls—The Effectiveness of a 10-Week Multicomponent Community Sports-Based Physical Activity Intervention for 8 to 12-Year-Old Girls'. *International Journal of Environmental Research and Public Health* **17** (18), 6928

Fatmi, H., Hussain, S. & Al-Rubaie, A. (2017}) 'Secure and Cost-Effective Remote Monitoring Health-Guard System'. In Anonymous (eds.) *2017 IEEE CANADA INTERNATIONAL HUMANITARIAN TECHNOLOGY CONFERENCE (IHTC)},* Held 345 E 47TH ST, NEW YORK, NY 10017 USA}: IEEE}

Fatmi, Z., Ntani, G. & Coggon, D. (2019) 'Coronary heart disease, hypertension and use of biomass fuel among women: comparative cross-sectional study.'. *BMJ Open* **9** (8), e030881

Feketa, V.P., Gleba, L.A., Palamarchuk, O.S., Savka, J.M. & Kivezhdi, K.B. (2016) '[CORRECTION OF HEART RATE VARIABILITY USING DIAPHRAGMATIC BREATHING MODE BIOFEEDBACK IN HEALTHY PEOPLE.]'. *Fiziologicheskii zhurnal* **62** (4), 66-75

Felder, S., Rasmussen, M.S., King, R., Sklow, B., Kwaan, M., Madoff, R. & Jensen, C. (2019) 'Prolonged thromboprophylaxis with low molecular weight heparin for abdominal or pelvic surgery'. *Cochrane Database of Systematic Reviews* (8)

Ferrante, F.E. (2005) 'Evolving telemedicine/ehealth technology'. *Telemedicine Journal & e-Health* **11** (3), 370-383

Ferri, M., Allara, E., Bo, A., Gasparrini, A. & Faggiano, F. (2013) 'Media campaigns for the prevention of illicit drug use in young people'. *Cochrane Database of Systematic Reviews* (6)

Field, M.J. & Grigsby, J. (2002) 'Telemedicine and remote patient monitoring.'. *JAMA* **288** (4), 423-425

Finkelstein, S.M., Speedie, S.M. & Potthoff, S. (2006) 'Home telehealth improves clinical outcomes at lower cost for home healthcare'. *Telemedicine Journal & e-Health* **12** (2), 128-136

Finkelstein, J., Cisse, P. & Jeong, I.C. (2015) 'Feasibility of Interactive Resistance Chair in Older Adults with Diabetes.'. *Studies in Health Technology & Informatics* **213**, 61-64

Finkelstein, S.M., Lindgren, B.R., Robiner, W., Lindquist, R., Hertz, M., Carlin, B.P. & VanWormer, A. (2013}) 'A Randomized Controlled Trial Comparing Health and Quality of Life of Lung Transplant Recipients Following Nurse and Computer-Based Triage Utilizing Home Spirometry Monitoring'. *TELEMEDICINE AND E-HEALTH}* **19}** (12}), 897-903}

Fisch, M.J., Chung, A.E. & Accordino, M.K. (2016) 'Using technology to improve cancer care: social media, wearables, and electronic health records'. *American Society of Clinical Oncology Educational Book* **36**, 200-208

Fletcher-Smith, J., Walker, M.F., Cobley, C.S., Steultjens, E. & Sackley, C.M. (2013) 'Occupational therapy for care home residents with stroke'. *Cochrane Database of Systematic Reviews* (6)

Floch, J., Zettl, A., Fricke, L., Weisser, T., Grut, L., Vilarinho, T., Stav, E., Ascolese, A. & Schauber, C. (2018) 'User needs in the development of a health app ecosystem for self-management of cystic fibrosis: user-centered development approach'. *JMIR mHealth and uHealth* **6** (5), e113

Flodgren G., Rachas A., Farmer A.J., Inzitari M. & Shepperd, S. (2015) 'Interactive telemedicine: effects on professional practice and health care outcomes.'. *The Cochrane database of systematic reviews* **9**, 002098

Flodgren, G., Conterno, L.O., Mayhew, A., Omar, O., Pereira, C.R. & Shepperd, S. (2013) 'Interventions to improve professional adherence to guidelines for prevention of device-related infections'. *Cochrane Database of Systematic Reviews* (3)

Flodgren, G., Gonçalves-Bradley, D. & Summerbell, C.D. (2017) 'Interventions to change the behaviour of health professionals and the organisation of care to promote weight reduction in children and adults with overweight or obesity'. *Cochrane Database of Systematic Reviews* (11)

Flodgren, G., Rachas, A., Farmer, A.J., Inzitari, M. & Shepperd, S. (2015) 'Interactive telemedicine: effects on professional practice and health care outcomes'. *Cochrane Database of Systematic Reviews* (9)

Flôres, F.S., Rodrigues, L.P., Copetti, F., Lopes, F. & Cordovil, R. (2019) 'Affordances for motor skill development in home, school, and sport environments: A narrative review'. *Perceptual and motor skills* **126** (3), 366-388

Folino, A.F., Breda, R., Calzavara, P., Borghetti, F., Comisso, J., Iliceto, S. & Buja, G. (2013) 'Remote follow-up of pacemakers in a selected population of debilitated elderly patients.'. *Europace* **15** (3), 382-387

Folino, A.F., Chiusso, F., Zanotto, G., Vaccari, D., Gasparini, G., Megna, A., Marras, E., Mantovan, R., Vaglio, A., Boscolo, G., Biancalana, G., Leoni, L., Iliceto, S. & Buja, G. (2011) 'Management of alert messages in the remote monitoring of implantable cardioverter defibrillators and pacemakers: an Italian single-region study.'. *Europace* **13** (9), 1281-1291

Fonseca-Pinto, R., Rijo, R., Assuncao, P., Seco, M.A., Guarino, M.P., Braga-Pontes, C., Gomes, D., Carreira, B., Correia, P., Oliveira, L., Pires, G., Leitao, C., Antunes, A., Januario, F. & Martinho, R. (2020}) 'Prescribe and Monitor Physical Activity Through a Community-Based eHealth Program: MOVIDA Platform'. In Badnjevic, A., Skrbic, R. and Pokvic, L. (eds.) *PROCEEDINGS OF THE INTERNATIONAL CONFERENCE ON MEDICAL AND BIOLOGICAL ENGINEERING, CMBEBIH 2019},* Held 233 SPRING STREET, NEW YORK, NY 10013, UNITED STATES}: SPRINGER}, 13-19}

Ford, K.L. (2020) *An Exploration of Digital Health Academic-industry Collaboration*

Ford, C.D., Kim, M.J., Dancy, B.L., Ford, C.D., Kim, M.J. & Dancy, B.L. (2009) 'Perceptions of hypertension and contributing personal and environmental factors among rural Southern African American women'. *Ethnicity & disease* **19** (4), 407-413

Forkan, A.R.M. & Khalil, I. (2017) 'A clinical decision-making mechanism for context-aware and patient-specific remote monitoring systems using the correlations of multiple vital signs.'. *Computer Methods & Programs in Biomedicine* **139**, 1-16

Forleo, G.B., Solimene, F., Pisanò, E.C., Zanotto, G., Calvi, V., Pignalberi, C., Maglia, G., Iacopino, S., Quartieri, F., Biffi, M., Caravati, F., Curnis, A., Capucci, A., Senatore, G., Santamaria, M., Della

Bella, P., Manzo, M., Giacopelli, D., Gargaro, A. & D'Onofrio, A. (2019) 'Long-term outcomes after prophylactic ICD and CRT-D implantation in nonischemic patients: Analysis from a nationwide database of daily remote-monitoring transmissions'. *Journal of cardiovascular electrophysiology* **30** (9), 1626-1635

Forouzanfar, M., Mabrouk, M., Rajan, S., Bolic, M., Dajani, H.R. & Groza, V.Z. (2017) 'Event Recognition for Contactless Activity Monitoring Using Phase-Modulated Continuous Wave Radar.'. *IEEE Transactions on Biomedical Engineering* **64** (2), 479-491

Foster C., Richards J., Thorogood M. & Hillsdon, M. (2013) 'Remote and web 2.0 interventions for promoting physical activity.'. *Cochrane Database of Systematic Reviews* **2013** (9) (pagination), Arte Number: 010395. ate of Pubaton: 30 Se 2013

Foster, C., Hillsdon, M., Thorogood, M., Kaur, A. & Wedatilake, T. (2005) 'Interventions for promoting physical activity'. *Cochrane Database of Systematic Reviews* (1)

Foster, C., Richards, J., Thorogood, M. & Hillsdon, M. (2013) 'Remote and web 2.0 interventions for promoting physical activity'. *Cochrane Database of Systematic Reviews* (9)

Foster, C.C., Steltzer, M., Snyder, A., Alden, C., Helner, K., Schinasi, D.A., Bohling, K. & Allen, K. (2021) 'Integrated Multimodality Telemedicine to Enhance In-Home Care of Infants During the Interstage Period.'. *Pediatric cardiology* **42** (2), 349-360

Fowler, L.A., Grammer, A.C., Staiano, A.E., Fitzsimmons-Craft, E.E., Chen, L., Yaeger, L.H. & Wilfley, D.E. (2021) 'Harnessing technological solutions for childhood obesity prevention and treatment: a systematic review and meta-analysis of current applications'. *International journal of obesity* , 1-25

Fox, K., Borer, J.S., Camm, A.J., Danchin, N., Ferrari, R., Lopez Sendon, J.L., Steg, P.G., Tardif, J., Tavazzi, L. & Tendera, M. (2007) 'Resting heart rate in cardiovascular disease'. *Journal of the American College of Cardiology* **50** (9), 823-830

Francis, R.P. (2019) 'Examining Healthcare Providers' Acceptance of Data From Patient Self-Monitoring Devices Using Structural Equation Modeling With the UTAUT2 Model'. *International Journal of Healthcare Information Systems and Informatics (IJHISI)* **14** (1), 44-60

Fransen, M., McConnell, S., Harmer, A.R., Van der Esch, M., Simic, M. & Bennell, K.L. (2015) 'Exercise for osteoarthritis of the knee'. *Cochrane Database of Systematic Reviews* (1)

Frazer, K., Callinan, J.E., McHugh, J., van Baarsel, S., Clarke, A., Doherty, K. & Kelleher, C. (2016) 'Legislative smoking bans for reducing harms from secondhand smoke exposure, smoking prevalence and tobacco consumption'. *Cochrane Database of Systematic Reviews* (2)

Freedman, B., Hindricks, G., Banerjee, A., Baranchuk, A., Ching, C.K., Du, X., Fitzsimons, D., Healey, J.S., Ikeda, T. & Lobban, T.C. (2021) 'World Heart Federation Roadmap on Atrial Fibrillation–A 2020 Update'. *Global Heart* **16** (1)

Fritzsche, D., Eitz, T., Grimmig, O., Brensing, A., Minami, K. & Korfer, R. (2004) 'Home monitoring of patients after prosthetic valve surgery -- experimental background and first clinical attempts.'. *Thoracic & Cardiovascular Surgeon* **52** (4), 211-217

Fritzsche, D., Eitz, T., Minami, K., Reber, D., Laczkovics, A., Mehlhorn, U., Horstkotte, D. & Korfer, R. (2005) 'Digital frequency analysis of valve sound phenomena in patients after prosthetic valve surgery: its capability as a true home monitoring of valve function.'. *Journal of Heart Valve Disease* **14** (5), 657-663

Fudge, C., Grant, M. & Wallbaum, H. (2020) 'Transforming cities and health: policy, action, and meaning'. *Cities & Health* **4** (2), 135-151

Galappaththy, G., Tharyan, P. & Kirubakaran, R. (2013) 'Primaquine for preventing relapse in people with Plasmodium vivax malaria treated with chloroquine'. *Cochrane Database of Systematic Reviews* (10)

Galderisi, A., Bruschettini, M., Russo, C., Hall, R. & Trevisanuto, D. (2020) 'Continuous glucose monitoring for the prevention of morbidity and mortality in preterm infants'. *Cochrane Database of Systematic Reviews* (12)

Ganapathy, K. & Ravindra, A. (2008) 'mHealth: A potential tool for health care delivery in India'. *Proceedings of the Making the ehealth Connection: Global Partnerships, Global Solutions*

Gao, W., Lv, X., Xu, X., Zhang, Z., Yan, J., Mao, G. & Xing, W. (2020) 'Telemedicine intervention–reduced blood pressure in a chronic disease population: A meta-analysis'. *Journal of telemedicine and telecare* , 1357633X20959581

Gao, X., Xu, J. & Liu, H. (2021) 'Current Status of Healthcare and Available E-Health Solutions in China'. *E-Business in the 21st Century: Essential Topics and Studies* , 169-199

Garcia Hernandez, M.T., Ruiz Capdevilla, J. & Sanchez Rodriguez, C. (2000) '[Description of the "new" intensive care unit of the Valladolid Rio Hortega Hospital]'. *Enfermeria Intensiva* **11** (4), 170-178

Garcia Martinez, R. (2011) 'E-health experiences in Spain: mapping and analysis'.

Garcia-Casal, M., Peña-Rosas, J., De-Regil, L., Gwirtz, J.A. & Pasricha, S.R. (2018) 'Fortification of maize flour with iron for controlling anaemia and iron deficiency in populations'. *Cochrane Database of Systematic Reviews* (12)

Garcia-Fernandez, F.J., Osca Asensi, J., Romero, R., Fernandez Lozano, I., Larrazabal, J.M., Martinez Ferrer, J., Ortiz, R., Pombo, M., Tornes, F.J. & Moradi Kolbolandi, M. (2019) 'Safety and efficiency of a common and simplified protocol for pacemaker and defibrillator surveillance based on remote monitoring only: a long-term randomized trial (RM-ALONE).'. *European heart journal* **40** (23), 1837-1846

GarciaGancedo L., Kelly M.L., Lavrov A., Parr J., Hart R., Marsden R., Turner M.R., Talbot K., Chiwera T., Shaw C.E. & AlChalabi, A. (2019) 'Objectively Monitoring Amyotrophic Lateral Sclerosis Patient Symptoms During Clinical Trials With Sensors: Observational Study.'. *JMIR mHealth and uHealth* **7** (12), e13433

Garcia-Gancedo, L., Kelly, M.L., Lavrov, A., Parr, J., Hart, R., Marsden, R., Turner, M.R., Talbot, K., Chiwera, T., Shaw, C.E. & Al-Chalabi, A. (2019) 'Objectively Monitoring Amyotrophic Lateral Sclerosis Patient Symptoms During Clinical Trials With Sensors: Observational Study.'. *JMIR MHealth and UHealth* **7** (12), e13433

Garell, C. (2015) 'Health games–healthy in what way?'.

Gaskin, K.L., Wray, J. & Barron, D.J. (2018) 'Acceptability of a parental early warning tool for parents of infants with complex congenital heart disease: a qualitative feasibility study.'. *Archives of Disease in Childhood* **103** (9), 880-886

Gauvin, D.V., Tilley, L.P., Smith, F.W.K.J. & Baird, T.J. (2006) 'Electrocardiogram, hemodynamics, and core body temperatures of the normal freely moving laboratory beagle dog by remote radiotelemetry.'. *Journal of Pharmacological & Toxicological Methods* **53** (2), 128-139

Gehring, K., Kloek, C.J., Aaronson, N.K., Janssen, K.W., Jones, L.W., Sitskoorn, M.M. & Stuiver, M.M. (2018) 'Feasibility of a home-based exercise intervention with remote guidance for patients with stable grade II and III gliomas: a pilot randomized controlled trial.'. *Clinical rehabilitation* **32** (3), 352-366

Gentry, S., van-Velthoven, M., Tudor Car, L. & Car, J. (2013) 'Telephone delivered interventions for reducing morbidity and mortality in people with HIV infection'. *Cochrane Database of Systematic Reviews* (5)

George, J., McNamara, K., Jackson, S., Hughes, J., Peterson, G., Bailey, M., Hsueh, A., Laua, R., Bortoloetto, D. & Stewart, K. (2010) 'The HAPPY trial: a randomised controlled trial of a community pharmacy-based intervention for improving patient adherence to antihypertensive medicines'. *International journal of pharmacy practice* **18**, 22-23

Georgiou, M., Morison, G., Smith, N., Tieges, Z. & Chastin, S. (2021) 'Mechanisms of Impact of Blue Spaces on Human Health: A Systematic Literature Review and Meta-Analysis'. *International journal of environmental research and public health* **18** (5), 2486

Gera, T., Shah, D., Garner, P., Richardson, M. & Sachdev, H.S. (2016) 'Integrated management of childhood illness (IMCI) strategy for children under five'. *Cochrane Database of Systematic Reviews* (6)

Gheorghiu, B. & Ratchford, F. (2015}) 'Scaling Up the Use of Remote Patient Monitoring in Canada'. In Gillis, G., Newsham, D. and Maeder, A. (eds.) *GLOBAL TELEHEALTH 2015: INTEGRATING TECHNOLOGY AND INFORMATION FOR BETTER HEALTHCARE},* Held NIEUWE HEMWEG 6B, 1013 BG AMSTERDAM, NETHERLANDS}: IOS PRESS}, 23-26}

Giansanti, D., Tiberi, Y., Silvestri, G. & Maccioni, G. (2009) 'Toward the integration of novel wearable step-counters in gait telerehabilitation after stroke.'. *Telemedicine Journal & E-Health* **15** (1), 105-111

Gibson, M., Thomson, H., Banas, K., Lutje, V., McKee, M.J., Martin, S.P., Fenton, C., Bambra, C. & Bond, L. (2018) 'Welfare-to-work interventions and their effects on the mental and physical health of lone parents and their children'. *Cochrane Database of Systematic Reviews* (2)

Gillespie, L.D., Robertson, M.C., Gillespie, W.J., Sherrington, C., Gates, S., Clemson, L. & Lamb, S.E. (2012) 'Interventions for preventing falls in older people living in the community'. *Cochrane Database of Systematic Reviews* (9)

Giordano, A., Scalvini, S., Paganoni, A.M., Baraldo, S., Frigerio, M., Vittori, C., Borghi, G., Marzegalli, M. & Agostoni, O. (2013) 'Home-based telesurveillance program in chronic heart failure: effects on clinical status and implications for 1-year prognosis.'. *Telemedicine Journal & E-Health* **19** (8), 605-612

Gleason, A.M. (2021) 'Remote Monitoring of a Work-From-Home Employee to Identify Stress: A Case Report.'. *Workplace Health & Safety* **2165079921997322**

Gleason, A.M. 'Remote Monitoring of a Work-From-Home Employee to Identify Stress: A Case Report'. *WORKPLACE HEALTH \& SAFETY}*

Glendinning, C. (2014) 'Supporting older people and carers'.

Glynn, L.G., Murphy, A.W., Smith, S.M., Schroeder, K. & Fahey, T. (2010) 'Interventions used to improve control of blood pressure in patients with hypertension'. *Cochrane Database of Systematic Reviews* (3)

Godfrey, A., Conway, R., Meagher, D. & ÓLaighin, G. (2008) 'Direct measurement of human movement by accelerometry'. *Medical engineering & physics* **30** (10), 1364-1386

Godinho, M.A., Jonnagaddala, J., Gudi, N., Islam, R., Narasimhan, P. & Liaw, S. (2020) 'mHealth for Integrated People-Centred Health Services in the Western Pacific: A Systematic Review'. *International journal of medical informatics* , 104259

Goff Jr, D.C., Khan, S.S., Lloyd-Jones, D., Arnett, D.K., Carnethon, M.R., Labarthe, D.R., Loop, M.S., Luepker, R.V., McConnell, M.V. & Mensah, G.A. (2021) 'Bending the curve in cardiovascular disease mortality: Bethesda 40 and beyond'. *Circulation* **143** (8), 837-851

Gogineni, J. (2012) *An ingenious decision support system for remote health monitoring of pregnant women*

Gokalp H., de Folter J., Verma V., Fursse J., Jones R. & Clarke, M. (2018) 'Integrated Telehealth and Telecare for Monitoring Frail Elderly with Chronic Disease.'. *Telemedicine journal and e-health : the official journal of the American Telemedicine Association* **24** (12), 940-957

Goldberg, E.M. & Levy, P.D. (2016) 'New approaches to evaluating and monitoring blood pressure'. *Current hypertension reports* **18** (6), 49

Goldstein, C. (2020) 'Current and Future Roles of Consumer Sleep Technologies in Sleep Medicine.'. *Sleep Medicine Clinics* **15** (3), 391-408

Gomes, B., Calanzani, N., Curiale, V., McCrone, P. & Higginson, I.J. (2013) 'Effectiveness and cost-effectiveness of home palliative care services for adults with advanced illness and their caregivers'. *Cochrane Database of Systematic Reviews* (6)

Gómez González, E. & Gómez Gutiérrez, E. (2020) 'Artificial Intelligence in Medicine and Healthcare: applications, availability and societal impact'.

Gooding, H.C., Gidding, S.S., Moran, A.E., Redmond, N., Allen, N.B., Bacha, F., Burns, T.L., Catov, J.M., Grandner, M.A. & Harris, K.M. (2020) 'Challenges and opportunities for the prevention and treatment of cardiovascular disease among young adults: Report from a National Heart, Lung, and Blood Institute Working Group'. *Journal of the American Heart Association* **9** (19), e016115

Goodison, S.E., Barnum, J.D., Vermeer, M.J., Woods, D., Sitar, S.I., Shelton, S.R. & Jackson, B.A. 'Wearable Sensor Technology and Potential Uses Within Law Enforcement'.

Goodspeed, R., Yan, X., Hardy, J., Vydiswaran, V.V., Berrocal, V.J., Clarke, P., Romero, D.M., Gomez-Lopez, I.N. & Veinot, T. (2018) 'Comparing the data quality of global positioning system devices and mobile phones for assessing relationships between place, mobility, and health: field study'. *JMIR mHealth and uHealth* **6** (8), e168

Goonesekera, Y. & Donkin, L. 'Stepping Outside the Therapist's Office: A Call for a Digital Mental Health Strategy in New Zealand'. *He ao kei tua o te awe māpara A new world awaits us on the horizon*

Gorczyca, A.M., Washburn, R.A., Ptomey, L., Mayo, M.S., Sullivan, D.K., Gibson, C.A., Lee, R., Stolte, S. & Donnelly, J.E. (2018) 'Weight management in rural health clinics: The Midwest diet and exercise trial'. *Contemporary clinical trials* **67**, 37-46

Gordon, W.J., Henderson, D., DeSharone, A., Fisher, H.N., Judge, J., Levine, D.M., MacLean, L., Sousa, D., Su, M.Y. & Boxer, R. (2020}) 'Remote Patient Monitoring Program for Hospital Discharged COVID-19 Patients'. *APPLIED CLINICAL INFORMATICS}* **11}** (05}), 792-801}

Goudet, S.M., Bogin, B.A., Madise, N.J. & Griffiths, P.L. (2019) 'Nutritional interventions for preventing stunting in children (birth to 59 months) living in urban slums in low- and middle-income countries (LMIC)'. *Cochrane Database of Systematic Reviews* (6)

Goudswaard, A.N., Furlong, N.J., Valk, G.D., Stolk, R.P. & Rutten, G. (2004) 'Insulin monotherapy versus combinations of insulin with oral hypoglycaemic agents in patients with type 2 diabetes mellitus'. *Cochrane Database of Systematic Reviews* (4)

GOVONI, L. (2012) 'Development of new technologies for the home monitoring and treatment of patients with chronic respiratory disorders'.

Greaves, R.F., Bernardini, S., Ferrari, M., Fortina, P., Gouget, B., Gruson, D., Lang, T., Loh, T.P., Morris, H.A. & Park, J.Y. (2019) 'Key questions about the future of laboratory medicine in the next decade of the 21st century: a report from the IFCC-Emerging Technologies Division'. *Clinica Chimica Acta* **495**, 570-589

Green, T. (2017) 'Trust Me, I'ma Doctor: Understanding Clinician's Experiences of Service Separation and Trust Formation in Telehealth'.

Green, B.B., Cook, A.J., Ralston, J.D., Fishman, P.A., Catz, S.L., Carlson, J., Carrell, D., Tyll, L., Larson, E.B. & Thompson, R.S. (2008) 'Effectiveness of home blood pressure monitoring, Web communication, and pharmacist care on hypertension control: a randomized controlled trial.'. *JAMA* **299** (24), 2857-2867

Greenhalgh, T., A'Court, C. & Shaw, S. (2017}) 'Understanding heart failure; explaining telehealth - a hermeneutic systematic review'. *BMC CARDIOVASCULAR DISORDERS}* **17}**

Griffiths, C.J.G. (2018) *Investigating the use of wearable technology to support safety in the workplace*

Grimsley, S.L. (2018) *The Effects of a School-Sponsored Health and Wellness Program Using Fit Bits in a Rural Community in the Southeastern Part of the United States*

Grobler, L., Marais, B.J. & Mabunda, S. (2015) 'Interventions for increasing the proportion of health professionals practising in rural and other underserved areas'. *Cochrane Database of Systematic Reviews* (6)

Grock, S., Ku, J., Kim, J. & Moin, T. (2017) 'A review of technology-assisted interventions for diabetes prevention'. *Current diabetes reports* **17** (11), 1-12

Groom, L.L., McCarthy, M.M., Stimpfel, A.W. & Brody, A.A. (2021) 'Telemedicine and Telehealth in Nursing Homes: An Integrative Review'. *Journal of the American Medical Directors Association*

Guédon-Moreau, L., Chevalier, P., Marquié, C., Kouakam, C., Klug, D., Lacroix, D., Brigadeau, F. & Kacet, S. (2010) 'Contributions of remote monitoring to the follow-up of implantable cardioverter-defibrillator leads under advisory'. *European heart journal* **31** (18), 2246-2252

Guedon-Moreau, L., Chevalier, P., Marquie, C., Kouakam, C., Klug, D., Lacroix, D., Brigadeau, F., Kacet, S. & ECOST trial Investigators (2010) 'Contributions of remote monitoring to the follow-up of implantable cardioverter-defibrillator leads under advisory.'. *European heart journal* **31** (18), 2246-2252

Guedon-Moreau, L., Lacroix, D., Sadoul, N., Clementy, J., Kouakam, C., Hermida, J., Aliot, E., Boursier, M., Bizeau, O., Kacet, S. & ECOST trial Investigators (2013) 'A randomized study of

remote follow-up of implantable cardioverter defibrillators: safety and efficacy report of the ECOST trial.'. *European heart journal* **34** (8), 605-614

Guerra, F., Malagoli, A., Contadini, D., Baiocco, E., Menditto, A., Bonelli, P., Rossi, L., Sticozzi, C., Zanni, A., Cai, J., Maitra, P., Villani, G.Q. & Capucci, A. (2020) 'Global Longitudinal Strain as a Predictor of First and Subsequent Arrhythmic Events in Remotely Monitored ICD Patients With Structural Heart Disease.'. *Jacc: Cardiovascular Imaging* **13** (1 Pt 1), 1-9

Guidi, G., Pollonini, L., Dacso, C.C. & Iadanza, E. (2015) 'A multi-layer monitoring system for clinical management of Congestive Heart Failure.'. *BMC Medical Informatics & Decision Making* **15** (Suppl 3), S5

Guillen, S., Arredondo, M.T., Traver, V., Garcia, J.M. & Fernandez, C. (2002) 'Multimedia telehomecare system using standard TV set.'. *IEEE Transactions on Biomedical Engineering* **49** (12), 1431-1437

Guimaraes Marcelino, C.A., Monteiro da Cruz, D. & Rueda, L.J. (2013) 'The efficacy of telephone use to assist and improve the wellbeing of family caregivers of persons with chronic diseases: a systematic review protocol'. *JBI Database of Systematic Reviews & Implementation Reports* **11** (2), 330-342

Gulan, L., Zunic, Z.S., Milic, G., Ishikawa, T., Omori, Y., Vuckovic, B., Nikezic, D., Krstic, D. & Bossew, P. (2014) 'First step of indoor thoron mapping of Kosovo and Metohija.'. *Radiation Protection Dosimetry* **162** (1-2), 157-162

Gullon, L., Gutierrez-Gutierrez, J., Sanchez Del Arco, R.T., Rivera, A., Fernandez, I. & Del Canizo, J.F. (2018) 'Development of an automated liver perfusion system: The benefit of a hemofilter.'. *International Journal of Artificial Organs* **41** (11), 723-729

Guo, X., Gu, X., Jiang, J., Li, H., Duan, R., Zhang, Y., Sun, L., Bao, Z., Shen, J. & Chen, F. (2019) 'A Hospital-Community-Family–Based Telehealth Program for Patients With Chronic Heart Failure: Single-Arm, Prospective Feasibility Study'. *JMIR mHealth and uHealth* **7** (12), e13229

Gupta, R. & Wood, D.A. (2019) 'Primary prevention of ischaemic heart disease: populations, individuals, and health professionals'. *The Lancet* **394** (10199), 685-696

Gurel, N.Z., Wittbrodt, M.T., Jung, H., Ladd, S.L., Shah, A.J., Vaccarino, V., Bremner, J.D. & Inan, O.T. (2020) 'Automatic Detection of Target Engagement in Transcutaneous Cervical Vagal Nerve Stimulation for Traumatic Stress Triggers.'. *IEEE Journal of Biomedical & Health Informatics* **24** (7), 1917-1925

Guzman-Clark, J., Farmer, M.M., Wakefield, B.J., Viernes, B., Yefimova, M., Lee, M.L. & Hahn, T.J. (2021) 'Why patients stop using their home telehealth technologies over time: Predictors of discontinuation in Veterans with heart failure'. *Nursing outlook* **69** (2), 159-166

Guzman-Clark, J., Wakefield, B.J., Farmer, M.M., Yefimova, M., Viernes, B., Lee, M.L. & Hahn, T.J. (2020) 'Adherence to the Use of Home Telehealth Technologies and Emergency Room Visits in Veterans with Heart Failure.'. *Telemedicine Journal & E-Health*

Hadar, E., Wolff, L., Tenenbaum-Gavish, K., Eisner, M., Shmueli, A., Barbash-Hazan, S., Bergel, R., Shmuel, E., Houri, O., Dollinger, S., Brzezinski-Sinai, N.A., Sukenik, S., Pardo, A., Navon, I., Wilk, Y., Zafrir-Danieli, H. & Wiznitzer, A. (2021) 'Mobile Self-Operated Home Ultrasound System for Remote Fetal Assessment During Pregnancy.'. *Telemedicine Journal & E-Health*

Halamandaris, V.J. (2004) 'Telemedicine revolution makes the home the center of health care'. *Caring* **23** (7), 52-55

Halberg, F., Cornelissen, G., Regal, P., Otsuka, K., Wang, Z., Katinas, G.S., Siegelova, J., Homolka, P., Prikryl, P., Chibisov, S.M., Holley, D.C., Wendt, H.W., Bingham, C., Palm, S.L., Sonkowsky, R.P., Sothern, R.B., Pales, E., Mikulecky, M., Tarquini, R., Perfetto, F., Salti, R., Maggioni, C., Jozsa, R., Konradov, A.A., Kharlitskaya, E.V., Revillam, M., Wan, C., Herold, M., Syutkina, E.V., Masalov, A.V., Faraone, P., Singh, R.B., Singh, R.K., Kumar, A., Singhs, R., Sundaram, S., Sarabandi, T., Pantaleoni, G., Watanabe, Y., Kumagai, Y., Gubin, D., Uezono, K., Olah, A., Borer, K., Kanabrockia, E.A., Bathina, S., Haus, E., Hillman, D., Schwartzkopff, O., Bakken, E.E. & Zeman, M. (2004) 'Chronoastrobiology: proposal, nine conferences, heliogeomagnetics, transyears, near-weeks, near-decades, phylogenetic and ontogenetic memories.'. *Biomedicine & Pharmacotherapy* **58** (Suppl 1), S150-87

Halimi, F., Clémenty, J., Attuel, P., Dessenne, X. & Amara, W. (2008) 'Optimized post-operative surveillance of permanent pacemakers by home monitoring: the OEDIPE trial'. *Europace : European pacing, arrhythmias, and cardiac electrophysiology : journal of the working groups on cardiac pacing, arrhythmias, and cardiac cellular electrophysiology of the European Society of Cardiology* **10** (12), 1392-1399

Hall, A.K., Dodd, V., Harris, A., McArthur, K., Dacso, C. & Colton, L.M. (2014) 'Heart failure patients' perceptions and use of technology to manage disease symptoms.'. *Telemedicine Journal & E-Health* **20** (4), 324-331

Hall, T., Lie, D.Y.C., Nguyen, T.Q., Mayeda, J.C., Lie, P.E., Lopez, J. & Banister, R.E. (2017) 'Non-Contact Sensor for Long-Term Continuous Vital Signs Monitoring: A Review on Intelligent Phased-Array Doppler Sensor Design'. *Sensors* **17** (11)

Halladay, J.R., Donahue, K.E., Hinderliter, A.L., Cummings, D.M., Cene, C.W., Miller, C.L., Garcia, B.A., Tillman, J. & Dewalt, D. (2013) 'The Heart Healthy Lenoir project--an intervention to reduce disparities in hypertension control: study protocol'. *BMC Health Services Research* **13** (1), 441-441

Halligan, J., Whelan, M.E., Roberts, N. & Farmer, A.J. (2021) 'Reducing weight and BMI following gestational diabetes: a systematic review and meta-analysis of digital and telemedicine interventions'. *BMJ open diabetes research & care* **9** (1), 10.1136/bmjdrc-2020-002077

Hameed, S.S., Rawal, I., Soni, D., Ajay, V.S., Goenka, S. & Prabhakaran, D. (2016) 'Technology for diagnosis, treatment, and prevention of cardiometabolic disease in India'. *Progress in cardiovascular diseases* **58** (6), 620-629

Hamilton, S., Mills, B., McRae, S. & Thompson, S. (2018) 'Evidence to service gap: cardiac rehabilitation and secondary prevention in rural and remote Western Australia.'. *BMC Health Services Research* **18** (1), 64

Handford, C., Tynan, A.M., Rackal, J.M. & Glazier, R. (2006) 'Setting and organization of care for persons living with HIV/AIDS'. *Cochrane Database of Systematic Reviews* (3)

Hargreaves S., Hawley M.S., Haywood A. & Enderby, P.M. (2017) 'Informing the Design of "Lifestyle Monitoring" Technology for the Detection of Health Deterioration in Long-Term Conditions: A Qualitative Study of People Living With Heart Failure.'. *Journal of medical Internet research* **19** (6), e231

Harky, A., Adan, A., Mohamed, M., Elmi, A. & Theologou, T. (2020) 'Technology and cardiovascular diseases in the era of COVID-19'. *Journal of cardiac surgery* **35** (12), 3551-3554

Harno, K., Kauppinen-Mäkelin, R. & Syrjäläinen, J. (2006) 'Managing diabetes care using an integrated regional e-health approach'. *Journal of Telemedicine & Telecare* **12** (1), S1:13-5

Harrington, R.A., Califf, R.M., Balamurugan, A., Brown, N., Benjamin, R.M., Braund, W.E., Hipp, J., Konig, M., Sanchez, E. & Joynt Maddox, K.E. (2020) 'Call to action: rural health: a presidential advisory from the American Heart Association and American Stroke Association'. *Circulation* **141** (10), e615-e644

Harst, L., Otto, L., Timpel, P., Richter, P., Lantzsch, H., Wollschlaeger, B., Winkler, K. & Schlieter, H. (2021) 'An empirically sound telemedicine taxonomy–applying the CAFE methodology'. *Journal of Public Health* , 1-12

Harst, L., Timpel, P., Otto, L., Richter, P., Wollschlaeger, B., Winkler, K. & Schlieter, H. (2019) 'Identifying barriers in telemedicine-supported integrated care research: scoping reviews and qualitative content analysis'. *Journal of Public Health* , 1-12

Hartasanchez, S.A., Heen, A.F., Kunneman, M., García-Bautista, A., Hargraves, I.G., Prokop, L.J., May, C.R. & Montori, V.M. (2021) 'Remote shared decision making through telemedicine: A systematic review of the literature'. *Patient education and counseling*

Hartin, P.J., Nugent, C.D., McClean, S.I., Cleland, I., Tschanz, J.T., Clark, C.J. & Norton, M.C. (2016) 'The empowering role of mobile apps in behavior change interventions: the Gray Matters randomized controlled trial'. *JMIR mHealth and uHealth* **4** (3), e93

Harzand, A., Witbrodt, B., Davis-Watts, M.L., Alrohaibani, A., Goese, D., Wenger, N.K., Shah, A.J. & Zafari, A.M. (2018) 'Feasibility of a Smartphone-enabled Cardiac Rehabilitation Program in Male Veterans With Previous Clinical Evidence of Coronary Heart Disease'. *The American Journal of Cardiology* **122** (9), 1471-1476

Hassan, S., Heinkel, S., Burton, A., Blackburn, R., McCloud, T., Ross, J., Osborn, D. & Walters, K. (2020) 'A qualitative study exploring the barriers and facilitators of implementing a cardiovascular disease risk reducing intervention for people with severe mental illness into primary care contexts across England: the 'PRIMROSE' trial.'. *BMC Health Services Research* **20** (1), 753

Hassanien, M.A. & El Shahawy, A.M. (2010}) 'ENVIRONMENTAL HEAVY METALS AND MENTAL DISORDERS OF CHILDREN IN DEVELOPING COUNTRIES'. In Simeonov, L., Kochubovski, M. and Simeonova, B. (eds.) *ENVIRONMENTAL HEAVY METAL POLLUTION AND EFFECTS ON CHILD MENTAL DEVELOPMENT: RISK ASSESSMENT AND PREVENTION STRATEGIES},* Held PO BOX 17, 3300 AA DORDRECHT, NETHERLANDS}: SPRINGER}, 1}

Hay, J., Maximova, K., Durksen, A., Carson, V., Rinaldi, R.L., Torrance, B., Ball, G.D., Majumdar, S.R., Plotnikoff, R.C. & Veugelers, P. (2012) 'Physical activity intensity and cardiometabolic risk in youth'. *Archives of Pediatrics & Adolescent Medicine* **166** (11), 1022-1029

Haya, N., Feiner, B., Baessler, K., Christmann-Schmid, C. & Maher, C. (2018) 'Perioperative interventions in pelvic organ prolapse surgery'. *Cochrane Database of Systematic Reviews* (8)

Hayes, S.L., Mann, M.K., Morgan, F.M., Kelly, M.J. & Weightman, A.L. (2012) 'Collaboration between local health and local government agencies for health improvement'. *Cochrane Database of Systematic Reviews* (10)

Haynes, S. & Kim, K.K. (2016) 'A Mobile Care Coordination System for the Management of Complex Chronic Disease.'. *Studies in Health Technology & Informatics* **225**, 505-509

He, F.J., Zhang, P., Luo, R., Li, Y., Chen, F., Zhao, Y., Zhao, W., Li, D., Chen, H., Wu, T., Yao, J., Li, J., Zhou, S., Liu, Y., Li, X., Wang, C. & MacGregor, G.A. (2019) 'An Application-based programme to reinforce and maintain lower salt intake (AppSalt) in schoolchildren and their families in China'. *BMJ open* **9** (7), e027793-2018-027793

He, F.J., Zhang, P., Luo, R., Li, Y., Chen, F., Zhao, Y., Zhao, W., Li, D., Chen, H., Wu, T., Yao, J., Li, J., Zhou, S., Liu, Y., Li, X., Wang, C. & MacGregor, G.A. (2019) 'An Application-based programme to reinforce and maintain lower salt intake (AppSalt) in schoolchildren and their families in China.'. *BMJ Open* **9** (7), e027793

Heidbuchel, H., Hindricks, G., Broadhurst, P., Van Erven, L., Fernandez-Lozano, I., Rivero-Ayerza, M., Malinowski, K., Marek, A., Romero Garrido, R.F., Loscher, S., Beeton, I., Garcia, E., Cross, S., Vijgen, J., Koivisto, U., Peinado, R., Smala, A. & Annemans, L. (2015) 'EuroEco (European Health Economic Trial on Home Monitoring in ICD Patients): a provider perspective in five European countries on costs and net financial impact of follow-up with or without remote monitoring.'. *European heart journal* **36** (3), 158-169

Helms, T.M., Zugck, C., Pelleter, J., Ronneberger, D.L. & Korb, H. (2007) '[Telemonitoring of cardiovascular diseases in Germany. Standing position and perspectives]'. *Herz* **32** (8), 641-649

Henning, O. & Thörn, C. (2020) 'User Experience Design of a Virtual Hospital: Increasing self-care through measurement and visualization of medical data'.

Heras-Mosteiro, J., Monge-Maillo, B., Pinart, M., Lopez Pereira, P., Reveiz, L., Garcia-Carrasco, E., Campuzano Cuadrado, P., Royuela, A., Mendez Roman, I. & López-Vélez, R. (2017) 'Interventions for Old World cutaneous leishmaniasis'. *Cochrane Database of Systematic Reviews* (12)

Hernández-Quiles, C., Garcia-Serrano, R., Bernabeu-Witte, M., Ruiz-Cantero, A., Colmenero-Camacho, M. & Ollero-Baturone, M. (2016) 'A randomized clinical trial for remote telemonitoring into an integrated care program for high complexity patients. Atlan-TIC project'. *International Journal of Integrated Care (IJIC)* **16** (6), 1-2

Hernández-Reyes, A., Cámara-Martos, F., Molina-Luque, R. & Moreno-Rojas, R. (2020) 'Effect of an mHealth Intervention Using a Pedometer App With Full In-Person Counseling on Body Composition of Overweight Adults: Randomized Controlled Weight Loss Trial'. *JMIR mHealth and uHealth* **8** (5), e16999

Heron, K.E. & Smyth, J.M. (2010) 'Ecological momentary interventions: incorporating mobile technology into psychosocial and health behaviour treatments'. *British journal of health psychology* **15** (1), 1-39

Herrera, C.A., Lewin, S., Paulsen, E., Ciapponi, A., Opiyo, N., Pantoja, T., Rada, G., Wiysonge, C.S., Bastías, G. & Garcia Marti, S. (2017) 'Governance arrangements for health systems in low-income countries: an overview of systematic reviews'. *Cochrane Database of Systematic Reviews* (9)

Hetrick, S.E., Cox, G.R., Witt, K.G., Bir, J.J. & Merry, S.N. (2016) 'Cognitive behavioural therapy (CBT), third-wave CBT and interpersonal therapy (IPT) based interventions for preventing depression in children and adolescents'. *Cochrane Database of Systematic Reviews* (8)

Hignett, S., Hogervorst, E., Griffiths, P. & Midlands, C.E. (2018) 'Research with and for Older People at Loughborough University'. *East Midlands Research into Ageing Network (EMRAN) Discussion Paper Series* (19)

Hilbel, T., Helms, T.M., Mikus, G., Katus, H.A. & Zugck, C. (2008) '[Telemetry in the clinical setting]'. *Herzschrittmachertherapie und Elektrophysiologie* **19** (3), 146-154

Hiltunen, P. 'Green exercise for families–What is already known?'.

Hilty, D.M., Armstrong, C.M., Edwards-Stewart, A., Gentry, M.T., Luxton, D.D. & Krupinski, E.A. (2021) 'Sensor, wearable, and remote patient monitoring competencies for clinical care and training: Scoping review'. *Journal of Technology in Behavioral Science* , 1-26

Hilty, D.M., Randhawa, K., Maheu, M.M., McKean, A.J., Pantera, R. & Mishkind, M.C. (2020) 'A review of telepresence, virtual reality, and augmented reality applied to clinical care'. *Journal of Technology in Behavioral Science* , 1-28

Hindricks, G., Elsner, C., Piorkowski, C., Taborsky, M., Geller, J.C., Schumacher, B., Bytesnik, J. & Kottkamp, H. (2014) 'Quarterly vs. yearly clinical follow-up of remotely monitored recipients of prophylactic implantable cardioverter-defibrillators: results of the REFORM trial.'. *European heart journal* **35** (2), 98-105

Hindricks, G., Varma, N., Kacet, S., Lewalter, T., Sogaard, P., Guedon-Moreau, L., Proff, J., Gerds, T.A., Anker, S.D. & Torp-Pedersen, C. (2017) 'Daily remote monitoring of implantable cardioverter-defibrillators: insights from the pooled patient-level data from three randomized controlled trials (IN-TIME, ECOST, TRUST)'. *European heart journal* **38** (22), 1749-1755

Ho, K., Kim-Sing, C., Ackenhusen, M., Wong, H., Marsden, J., Nasiopoulos, P., Mitton, C., Christenson, J., Lauscher, H.N., Grafstein, E., Abu Laban, R., Hawkins, N., Nguyen, J., Cordeiro, J., Cuthbertson, L., Da Silva, B., Chang, T., McGavin, C., Kisch, D., Kisch, I., Wattling, D., Seeley, K., Malovec, S., Gaspranov, M., Harps, H., Yngreso, A., Tuason, M., Wilkinson, S., Sawyer, D. & TEC4Home Healthcare Innovation Com (2016}) 'Supporting Heart Failure Patient Transitions From Acute to Community Care With Home Telemonitoring Technology: A Protocol for a Provincial Randomized Controlled Trial (TEC4Home)'. *JMIR RESEARCH PROTOCOLS}* **5}** (4})

Hofmann, R., Völler, H., Nagels, K., Bindl, D., Vettorazzi, E., Dittmar, R., Wohlgemuth, W., Neumann, T., Störk, S. & Bruder, O. (2015) 'First outline and baseline data of a randomized, controlled multicenter trial to evaluate the health economic impact of home telemonitoring in chronic heart failure - CardioBBEAT'. *Trials* **16**, 343

Holland, A.E., Cox, N.S., Houchen-Wolloff, L., Rochester, C.L., Garvey, C., ZuWallack, R., Nici, L., Limberg, T., Lareau, S.C. & Yawn, B.P. (2021) 'Defining Modern Pulmonary Rehabilitation. An Official American Thoracic Society Workshop Report'. *Annals of the American Thoracic Society* **18** (5), e12-e29

Hollands, G.J., Carter, P., Anwer, S., King, S.E., Jebb, S.A., Ogilvie, D., Shemilt, I., Higgins, J. & Marteau, T.M. (2019) 'Altering the availability or proximity of food, alcohol, and tobacco products to change their selection and consumption'. *Cochrane Database of Systematic Reviews* (9)

Hollands, G.J., Shemilt, I., Marteau, T.M., Jebb, S.A., Lewis, H.B., Wei, Y., Higgins, J. & Ogilvie, D. (2015) 'Portion, package or tableware size for changing selection and consumption of food, alcohol and tobacco'. *Cochrane Database of Systematic Reviews* (9)

Holthe, H. & Serrano, J.A. (2015) 'ePoint.telemed--An Open Web-based Platform for Home Monitoring of Patients with Chronic Heart Failure'. *Studies in health technology and informatics* **216**, 74-78

Holzel, L.P., Harter, M. & Hull, M. (2017) '[Multiprofessional outpatient psychosocial treatment for elderly patients with mental disorders]'. *Nervenarzt* **88** (11), 1227-1233

Hooper, G., Yellowlees, P., Marwick, T., Currie, P. & Bidstrup, B. (2001) 'Telehealth and the diagnosis and management of cardiac disease'. *Journal of telemedicine and telecare* **7** (5), 249-256

Hooper, L., Abdelhamid, A., Bunn, D., Brown, T., Summerbell, C.D. & Skeaff, C.M. (2015) 'Effects of total fat intake on body weight'. *Cochrane Database of Systematic Reviews* (8)

Horgan, D., Romao, M., Morre, S.A. & Kalra, D. (2019) 'Artificial Intelligence: Power for Civilisation - and for Better Healthcare'. *Public health genomics* **22** (5-6), 145-161

Horton, D.B., Bhullar, H., Carty, L., Cunningham, F., Ogdie, A., Sultana, J. & Trifirò, G. (2019) 'Electronic health record databases'. *Pharmacoepidemiology* , 241-289

Hosseiniravandi, M., Kahlaee, A.H., Karim, H., Ghamkhar, L. & Safdari, R. (2020) 'Home-based telerehabilitation software systems for remote supervising: a systematic review.'. *International Journal of Technology Assessment in Health Care* **36** (2), 113-125

Hou, I., Lan, M., Shen, S., Tsai, P.Y., Chang, K.J., Tai, H., Tsai, A., Chang, P., Wang, T. & Sheu, S. (2020) 'The development of a mobile health app for breast cancer self-management support in Taiwan: design thinking approach'. *JMIR mHealth and uHealth* **8** (4), e15780

Hoult J.P., Kramer K.M., Seifert S. & Benzo, R. (2017) 'Feasibility of home-based pulmonary rehabilitation in patients with COPD.'. *American Journal of Respiratory and Critical Care Medicine* **Conference**, Ameran

Houze de l'Aulnoit, A., Boudet, S., Genin, M., Gautier, P., Schiro, J., Houze de l'Aulnoit, D. & Beuscart, R. (2018) 'Development of a Smart Mobile Data Module for Fetal Monitoring in E-Healthcare.'. *Journal of medical systems* **42** (5), 83

Hu, F., Jiang, M., Wagner, M. & Dong, D. (2007) 'Privacy-preserving telecardiology sensor networks: toward a low-cost portable wireless hardware/software codesign.'. *IEEE Transactions on Information Technology in Biomedicine* **11** (6), 619-627

Huang, C., Chen, Y., Hung, C., Lee, J., Hsu, T., Wu, H., Chuang, P., Chen, M. & Ho, Y. (2021) 'The Association Between Short-term Exposure to Ambient Air Pollution and Patient-Level Home Blood Pressure Among Patients With Chronic Cardiovascular Diseases in a Web-Based Synchronous Telehealth Care Program: Retrospective Study.'. *JMIR Public Health and Surveillance* **7** (6), e26605

Huang, W., Chen, C., Chang, Y., Chen, Y., Huang, J., Yang, C.M. & Yang, T.L. (2008) 'Exquisite textiles sensors and wireless sensor network device for home health care.'. *Annual International Conference Of The IEEE Engineering In Medicine And Biology Society* **2008**, 546-549

Hudson, D.L. & Cohen, M.E. (2010}) 'Intelligent agents in home healthcare'. *ANNALS OF TELECOMMUNICATIONS}* **65}** (9-10}), 593-600}

Hull, S., Tailor, V., Balduzzi, S., Rahi, J., Schmucker, C., Virgili, G. & Dahlmann-Noor, A. (2017) 'Tests for detecting strabismus in children aged 1 to 6 years in the community'. *Cochrane Database of Systematic Reviews* (11)

Hummers, E. (2021) 'Digital medicine and e-health in primary care. Selected abstracts from the virtual 90th EGPRN conference, 16–17 October 2020'. *EUROPEAN JOURNAL OF GENERAL PRACTICE* **27** (1), 10-18

Hung, K. & Zhang, Y. (2003) 'Implementation of a WAP-based telemedicine system for patient monitoring.'. *IEEE Transactions on Information Technology in Biomedicine* **7** (2), 101-107

Hunt, H., Stanworth, S., Curry, N., Woolley, T., Cooper, C., Ukoumunne, O., Zhelev, Z. & Hyde, C. (2015) 'Thromboelastography (TEG) and rotational thromboelastometry (ROTEM) for

trauma-induced coagulopathy in adult trauma patients with bleeding'. *Cochrane Database of Systematic Reviews* (2)

Huntington, M.K., Guzman, A.I., Roemen, A., Fieldsend, J. & Saloum, H. (2013) 'Hospital-to-Home: a hospital readmission reduction program for congestive heart failure.'. *South Dakota Medicine: The Journal of the South Dakota State Medical Association* **66** (9), 370-373

Hussain, M.S., Li, J., Brindal, E., van Kasteren, Y., Varnfield, M., Reeson, A., Berkovsky, S. & Freyne, J. (2017) 'Supporting the delivery of total knee replacements care for both patients and their clinicians with a mobile app and web-based tool: randomized controlled trial protocol'. *JMIR research protocols* **6** (3), e32

Hutchinson, K., Pellicori, P., Dierckx, R., Cleland, J.G.F. & Clark, A.L. (2014) 'Remote telemonitoring for patients with heart failure: might monitoring pulmonary artery pressure become routine?'. *Expert Review of Cardiovascular Therapy* **12** (8), 1025-1033

Ibaida, A. & Khalil, I. (2013) 'Wavelet-Based ECG Steganography for Protecting Patient Confidential Information in Point-of-Care Systems.'. *IEEE Transactions on Biomedical Engineering* **60** (12), 3322-3330

Idris, S., Degheim, G., Ghalayini, W., Larsen, T.R., Nejad, D. & David, S. (2015) 'Home Telemedicine in Heart Failure: a Pilot Study of Integrated Telemonitoring and Virtual Provider Appointments'. *Reviews in cardiovascular medicine* **16** (2), 156-162

Iellamo, F., Sposato, B. & Volterrani, M. (2020) 'Telemonitoring for the Management of Patients with Heart Failure'. *Cardiac Failure Review* **6**, e07

Ilhan, B., Bock, T., Linner, T., Iturralde, K., Pan, W. & Hu, R. (2019) '13 Innovative robotics and automation for offsite manufacturing'. *Offsite production and manufacturing for innovative construction: People, process and technology* , 309

Imdad, A., Bautista, R., Senen, K., Uy, M., Mantaring III, J. & Bhutta, Z.A. (2013) 'Umbilical cord antiseptics for preventing sepsis and death among newborns'. *Cochrane Database of Systematic Reviews* (5)

Imre, Ö. & Wass, S. (2019) 'Trends in Patient Generated Data–An Initial Review'.

Indraratna, P., Biswas, U., Yu, J., Schreier, G., Ooi, S.Y., Lovell, N.H. & Redmond, S.J. (2021) 'Trials and Tribulations: mHealth Clinical Trials in the COVID-19 Pandemic'. *Yearbook of medical informatics*

Indraratna, P., Biswas, U., Yu, J., Schreier, G., Ooi, S., Lovell, N.H. & Redmond, S.J. (2021) 'Trials and Tribulations: mHealth Clinical Trials in the COVID-19 Pandemic.'. *Yearbook of Medical Informatics*

Inglis, S.C., Clark, R.A., Dierckx, R., Prieto-Merino, D. & Cleland, J. (2015) 'Structured telephone support or non-invasive telemonitoring for patients with heart failure'. *Cochrane Database of Systematic Reviews* (10)

Inglis, S.C., Clark, R.A., McAlister, F.A., Stewart, S. & Cleland, J.G.F. (2011) 'Which components of heart failure programmes are effective? A systematic review and meta-analysis of the outcomes of structured telephone support or telemonitoring as the primary component of chronic heart failure management in 8323 patients: Abridged Cochrane Review.'. *European Journal of Heart Failure* **13** (9), 1028-1040

Inglis, S.C., Conway, A., Cleland, J.G.F. & Clark, R.A. (2015}) 'Is age a factor in the success or failure of remote monitoring in heart failure? Telemonitoring and structured telephone support in elderly heart failure patients'. *EUROPEAN JOURNAL OF CARDIOVASCULAR NURSING}* **14}** (3}), 248-255}

Iqbal, F.M., Joshi, M., Davies, G., Khan, S., Ashrafian, H. & Darzi, A. (2021) 'Design of the pilot, proof of concept REMOTE-COVID trial: remote monitoring use in suspected cases of COVID-19 (SARS-CoV-2).'. *Pilot & Feasibility Studies* **7** (1), 62

Iqbal, F.M., Joshi, M., Davies, G., Khan, S., Ashrafian, H. & Darzi, A. (2021) 'The pilot, proof of concept REMOTE-COVID trial: remote monitoring use in suspected cases of COVID-19 (SARS-CoV 2).'. *BMC Public Health* **21** (1), 638

Iqbal, M., Chowdhury Aa, M., Baherjee, S., Islam, S., Iqbal, S., Hossain, R., Islam, M., Chaudhury, S., Hassan, M. & Adhikary, D. (2020) 'SUN-138 Etiology and Reno - Cardio -Metabolic Risk Factors Prevailing in Hypertensive Patients of Rural Bangladesh'. *Kidney international reports* **5** (3), S258-

IRCT2012071710311N1, (2012) 'Does extension of remote ischemic preconditioning have cardioprotective effect on patients underwent CABG surgeries?'. *http://www.who.int/trialsearch/Trial2.aspx?TrialID=IRCT2012071710311N1*

IRCT20161018030366N3, (2019) 'The effect telenursing care on caregivers burden in family with Chronic Heart failure'. *http://www.who.int/trialsearch/Trial2.aspx?TrialID=IRCT20161018030366N3*

Ishani, A., Christopher, J., Palmer, D., Otterness, S., Clothier, B., Nugent, S., Nelson, D., Rosenberg, M.E. & Ctr Innovative Kidney Care (2016}) 'Telehealth by an Interprofessional Team in Patients With CKD: A Randomized Controlled Trial'. *AMERICAN JOURNAL OF KIDNEY DISEASES}* **68}** (1}), 41-49}

Isik, A.H. & Guler, I. (2012) 'Pulse oximeter based mobile biotelemetry application.'. *Studies in Health Technology & Informatics* **181**, 197-201

Islam, S.M.S., Ahmed, S., Uddin, R., Siddiqui, M.U., Malekahmadi, M., Al Mamun, A., Alizadehsani, R., Khosravi, A. & Nahavandi, S. (2021) 'Cardiovascular diseases risk prediction in patients with diabetes: Posthoc analysis from a matched case-control study in Bangladesh'. *Journal of Diabetes & Metabolic Disorders* , 1-9

Islam, S.M.S., Cartledge, S., Karmakar, C., Rawstorn, J.C., Fraser, S.F., Chow, C. & Maddison, R. (2019) 'Validation and acceptability of a cuffless wrist-worn wearable blood pressure monitoring device among users and health care professionals: mixed methods study'. *JMIR mHealth and uHealth* **7** (10), e14706

Islam, S.M.S. & Maddison, R. (2020) 'A Comparison of Blood Pressure Data Obtained From Wearable, Ambulatory, and Home Blood Pressure Monitoring Devices: Prospective Validation Study'. *JMIR Data* **1** (1), e22436

ISRCTN10469580, (2018) 'Lifestyle physical activity counselling for in-patients with major depressive disorder'. *http://www.who.int/trialsearch/Trial2.aspx?TrialID=ISRCTN10469580*

ISRCTN13472393, (2020) 'Isometric exercise for people with raised blood pressure'. *http://www.who.int/trialsearch/Trial2.aspx?TrialID=ISRCTN13472393*

ISRCTN15394285, (2018) 'Manchester Intermittent and Daily diet Diabetes App Study'. http://www.who.int/trialsearch/Trial2.aspx?TrialID=ISRCTN15394285

ISRCTN23905886, (2012) 'Enhanced diabetes-cardiovascular management through primary health care in Pakistan'. http://www.who.int/trialsearch/Trial2.aspx?TrialID=ISRCTN23905886

ISRCTN41238563, (2016) 'Effects of remote patient monitoring on chronic disease management'. http://www.who.int/trialsearch/Trial2.aspx?TrialID=ISRCTN41238563

ISRCTN41424840, (2008) 'A new model for continuous care of chronic patients - eCare and eLearning for patients with chronic obstructive pulmonary disease (COPD)'. http://www.who.int/trialsearch/Trial2.aspx?TrialID=ISRCTN41424840

ISRCTN50086114, (2010) 'Prescribing exercise for diabetes prevention'. http://www.who.int/trialsearch/Trial2.aspx?TrialID=ISRCTN50086114

ISRCTN82391603, (2018) 'Cardiovascular effects of empagliflozin in diabetes mellitus'. http://www.who.int/trialsearch/Trial2.aspx?TrialID=ISRCTN82391603

ISRCTN83809392, (2002) 'Improvement in diabetes control with diabetes monitoring system through remote log-on with Vtech CV 8300'. http://www.who.int/trialsearch/Trial2.aspx?TrialID=ISRCTN83809392

Iversen, M.M., Espehaug, B., Hausken, M.F., Graue, M., Østbye, T., Skeie, S., Cooper, J.G., Tell, G.S., Günther, B.E. & Dale, H. (2016) 'Telemedicine versus standard follow-up care for diabetes-related foot ulcers: protocol for a cluster randomized controlled noninferiority trial (DiaFOTo)'. *JMIR Research protocols* **5** (3), e5646

Iwaya, L.H., Ahmad, A. & Babar, M.A. (2020) 'Security and Privacy for mHealth and uHealth Systems: a Systematic Mapping Study'. *IEEE Access* **8**, 150081-150112

Iyawa, G.E., Ondiek, C.O. & Osakwe, J.O. (2020) 'mHealth: A Low-Cost Approach for Effective Disease Diagnosis, Prediction, Monitoring and Management–Effective Disease Diagnosis'. *Smart Medical Data Sensing and IoT Systems Design in Healthcare* , 1-21

Iyngkaran, P., Toukhsati, S., Biddagardi, N., Zimmet, H., Atherton, J.J. & Hare, D. (2015) 'Technology-assisted congestive heart failure care'. *Current heart failure reports* **12** (2), 173-186

Ja’mese, V.B., Messiah, S.E., Hansen, E., Nardi, M.I., Hawver, E., Patel, H.H., Kling, H., Okeke, D. & D’Agostino, E.M. (2021) 'Objective Measurement of Physical Activity Attributed to a Park-Based Afterschool Program'. *Journal of Physical Activity and Health* **18** (3), 329-336

Jaana, M. & Sherrard, H. (2019}) 'Rural-Urban Comparison of Telehome Monitoring for Patients with Chronic Heart Failure'. *TELEMEDICINE AND E-HEALTH}* **25}** (2}), 101-108}

Jackson, G.L., Oddone, E.Z., Olsen, M.K., Powers, B.J., Grubber, J.M., McCant, F. & Bosworth, H.B. (2012) 'Racial differences in the effect of a telephone-delivered hypertension disease management program'. *Journal of general internal medicine* **27** (12), 1682-1689

Jackson, G.L., Oddone, E.Z., Olsen, M.K., Powers, B.J., Grubber, J.M., McCant, F., Bosworth, H.B., Jackson, G.L., Oddone, E.Z., Olsen, M.K., Powers, B.J., Grubber, J.M., McCant, F. & Bosworth, H.B. (2012) 'Racial differences in the effect of a telephone-delivered hypertension disease management program'. *JGIM: Journal of General Internal Medicine* **27** (12), 1682-1689

Jackson, G.L., Oddone, E.Z., Olsen, M.K., Powers, B.J., Grubber, J.M., McCant, F. & Bosworth, H.B. (2012) 'Racial differences in the effect of a telephone-delivered hypertension disease management program.'. *Journal of General Internal Medicine* **27** (12), 1682-1689

Jacobs, S.E., Berg, M., Hunt, R., Tarnow-Mordi, W., Inder, T.E. & Davis, P.G. (2013) 'Cooling for newborns with hypoxic ischaemic encephalopathy'. *Cochrane Database of Systematic Reviews* (1)

Jacobson Vann, J., Jacobson, R.M., Coyne-Beasley, T., Asafu-Adjei, J. & Szilagyi, P.G. (2018) 'Patient reminder and recall interventions to improve immunization rates'. *Cochrane Database of Systematic Reviews* (1)

Jacobson, C. & Karjalainen, P. (2019) *EMBRACING INTERNET OF MEDICAL THINGS A multiple case study of contextual factors' influence on the implementation of IoT healthcare solutions*

Jago, R., Edwards, M.J., Urbanski, C.R. & Sebire, S.J. (2013) 'General and specific approaches to media parenting: a systematic review of current measures, associations with screen-viewing, and measurement implications'. *Childhood obesity* **9** (s1), S-51-S-72

Jancey, J., James, A., Lee, A., Howat, P., Hills, A.P., Anderson, A.S., Bordin, C. & Blackford, K. (2019) 'Metabolic syndrome in rural Australia: an opportunity for primary health care'. *The Australian Journal of Rural Health* **27** (3), 210-215

Jaworek, J. & Augustyniak, P. (2012}) 'Heart Rate Monitoring System Dedicated for Cardiac Telerehabilitation'. In Jobbagy, A. (eds.) *5TH EUROPEAN CONFERENCE OF THE INTERNATIONAL FEDERATION FOR MEDICAL AND BIOLOGICAL ENGINEERING, PTS 1 AND 2},* Held 233 SPRING STREET, NEW YORK, NY 10013, UNITED STATES}: SPRINGER}, 462-465}

Jay, M., Orstad, S.L., Wali, S., Wylie-Rosett, J., Tseng, C.H., Sweat, V., Wittleder, S., Shu, S.B., Goldstein, N.J. & Ladapo, J.A. (2019) 'Goal-directed versus outcome-based financial incentives for weight loss among low-income patients with obesity: rationale and design of the Financial Incentives foR Weight Reduction (FIReWoRk) randomised controlled trial'. *BMJ open* **9** (4), e025278-2018-025278

Jefferson, T., Jones, M.A., Doshi, P., Del Mar, C., Hama, R., Thompson, M.J., Spencer, E.A., Onakpoya, I.J., Mahtani, K.R. & Nunan, D. (2014) 'Neuraminidase inhibitors for preventing and treating influenza in adults and children'. *Cochrane Database of Systematic Reviews* (4)

Jehn, M., Prescher, S., Koehler, K., von Haehling, S., Winkler, S., Deckwart, O., Honold, M., Sechtem, U., Baumann, G., Halle, M., Anker, S.D. & Koehler, F. (2013) 'Tele-accelerometry as a novel technique for assessing functional status in patients with heart failure: feasibility, reliability and patient safety.'. *International journal of cardiology* **168** (5), 4723-4728

Jelkic, N., Canji, T., Ivanovic, V., Srdanovic, I., Mirkovic, M. & Vujin, B. (2003) '[Telemedicine and cardiology]'. *Medicinski pregled* **56** (3-4), 187-192

Jenkins, R.L. & McSweeney, M. (2001) 'Assessing elderly patients with congestive heart failure via in-home interactive telecommunication.'. *Journal of gerontological nursing* **27** (1), 21-27

Jennett, P., Hall, L., Hailey, D., Ohinmaa, A., Anderson, C., Thomas, R., Young, B., Lorenzetti, D. & Scott, R. (2003}) 'The socio-economic impact of telehealth: a systematic review'. *JOURNAL OF TELEMEDICINE AND TELECARE}* **9}** (6}), 311-320}

Jeong, C., Salehi, S., Wu, J., North, M.L., Kim, J.S., Chow, C. & Evans, G.J. (2019) 'Indoor measurements of air pollutants in residential houses in urban and suburban areas: Indoor versus ambient concentrations.'. *Science of the Total Environment* **693**, 133446

Jiamjariyaporn, T., Ingsathit, A., Tungsanga, K., Banchuin, C., Vipattawat, K., Kanchanakorn, S., Leesmidt, V., Watcharasaksilp, W., Saetie, A. & Pachotikarn, C. (2014) 'Effectiveness of integrated care on delaying chronic kidney disease progression in rural communities of Thailand (ESCORT study): rationale and design of the study'. *BMC nephrology* **15**, 99

Jiamjariyaporn, T., Ingsathit, A., Tungsanga, K., Banchuin, C., Vipattawat, K., Kanchanakorn, S., Leesmidt, V., Watcharasaksilp, W., Saetie, A., Pachotikarn, C., Taechangam, S., Teerapornlertratt, T., Chantarojsiri, T. & Sitprija, V. (2014) 'Effectiveness of integrated care on delaying chronic kidney disease progression in rural communities of Thailand (ESCORT study): rationale and design of the study [NCT01978951]'. *BMC Nephrology* **15** (1), 99-99

Jiang, Y., Chao, S., Liu, J., Yang, Y., Chen, Y., Zhang, A. & Cao, H. (2017) 'Source apportionment and health risk assessment of heavy metals in soil for a township in Jiangsu Province, China.'. *Chemosphere* **168**, 1658-1668

Jin, M.L., Brown, M.M., Dhir, P., Nirmalan, A. & Edwards, P.A. (2021) 'Telemedicine, Telementoring, and Telesurgery for Surgical Practices'. *Current problems in surgery* , 100987

Jin-Gang, W., Xiao-Bo, S., Ping, W., Wei, H. & Cui-Lian, D. (2005) 'Remote heart sound monitoring system.'. *Conference Proceedings: ...Annual International Conference of the IEEE Engineering in Medicine & Biology Society* **2005**, 2138-2140

Jirong, Y., Yang, X., Wu, T., Defen, S. & Dong, B. (2004) 'Zhiling decoction for vascular dementia'. *Cochrane Database of Systematic Reviews* (4)

John, O. & Jha, V. (2019}) 'Remote Patient Management in Peritoneal Dialysis: An Answer to an Unmet Clinical Need'. In *REMOTE PATIENT MANAGEMENT IN PERITONEAL DIALYSIS}*, ed. by Ronco, C., Crepaldi, C. & Rosner, M.,. POSTFACH, CH-4009 BASEL, SWITZERLAND}: KARGER}, 99-112}

Johnson, K.E. (2017) 'A comparison of video conferencing and in-person health coaching approaches in combination with mHealth devices on weight loss, physical activity, and glycemic control'.

Johnson, P. (1998) 'The clinical and economic advantages of remote, community-based physiological assessment.'. *Journal of Telemedicine & Telecare* **4** (Suppl 1), 64-66

Johnson, S., Henschke, N., Maayan, N., Mills, I., Buckley, B.S., Kakourou, A. & Marshall, R. (2018) 'Ribavirin for treating Crimean Congo haemorrhagic fever'. *Cochrane Database of Systematic Reviews* (6)

Jokanović, V. (2020) 'Smart Healthcare in Smart Cities'. *Towards Smart World: Homes to Cities Using Internet of Things* , 45

Jonasson, O. (1989) 'Waiting in line: should selected patients ever be moved up?.'. *Transplantation proceedings* **21** (3), 3390-3394

Jones, G.C. (2015) *Validating step count of wearable physical activity trackers*

Joosen, P., Piette, D., Buekers, J., Taelman, J., Berckmans, D. & De Boever, P. (2019) 'A smartphone-based solution to monitor daily physical activity in a care home.'. *Journal of Telemedicine & Telecare* **25** (10), 611-622

Joshi, R., Agrawal, T., Fathima, F., Usha, T., Thomas, T., Misquith, D., Kalantri, S., Chidambaram, N., Raj, T. & Singamani, A. (2019) 'Cardiovascular risk factor reduction by community health workers in rural India: a cluster randomized trial'. *American Heart Journal* **216**, 9-19

Joyce, P., Moore, Z. & Christie, J. (2018) 'Organisation of health services for preventing and treating pressure ulcers'. *Cochrane Database of Systematic Reviews* (12)

JPRN-UMIN000024785, (2016) 'The effectiveness of remote medical support service using an information and communication technology (ICT) in patients with diabetic nephropathy: a pilot study with non-randomized control trial'. *http://www.who.int/trialsearch/Trial2.aspx?TrialID=JPRN-UMIN000024785*

JPRN-UMIN000027060, (2017) 'A Study on Ability to Detect Early Deterioration of Heart Failure with Respiratory Instability Telemonitoring - Aided by Innovative Tele-Monitoring Environment To Halt Ongoing Deterioration of Heart Failure: ITMETHOD-HF -'. *http://www.who.int/trialsearch/Trial2.aspx?TrialID=JPRN-UMIN000027060*

JPRN-UMIN000028937, (2017) 'Reduction of Uncontrolled Hypertension by Remote Monitoring and Telemedicine (REMOTE) Study'. *http://www.who.int/trialsearch/Trial2.aspx?TrialID=JPRN-UMIN000028937*

JPRN-UMIN000039007, (2019) 'Remote lifestyle modification to control risk factors for secondary prevention in ischemic heart disease'. *http://www.who.int/trialsearch/Trial2.aspx?TrialID=JPRN-UMIN000039007*

JPRN-UMIN000039325, (2020) 'Verification of the usefulness of a remote exereise program by coordination of medical team and nursing staff'. *http://www.who.int/trialsearch/Trial2.aspx?TrialID=JPRN-UMIN000039325*

Kahlert, D. & Ehrhardt, N. (2020) 'Out-of-Home Mobility and Social Participation of Older People: a Photo-Based Ambulatory Assessment Study'. *Journal of Population Ageing* , 1-14

Kalafat, E., Benlioglu, C., Thilaganathan, B. & Khalil, A. (2020) 'Home blood pressure monitoring in the antenatal and postpartum period: A systematic review meta-analysis.'. *Pregnancy Hypertension* **19**, 44-51

Kalid, N., Zaidan, A., Zaidan, B., Salman, O.H., Hashim, M. & Muzammil, H. (2018) 'Based real time remote health monitoring systems: A review on patients prioritization and related" big data" using body sensors information and communication technology'. *Journal of medical systems* **42** (2), 30

Kallstrom, T.J. (2018) 'Can't We Do Better for Our Patients?'. *AARC Times* **42** (3), 12-13

Kalra, D., Vijayaraghavan, K., Sikand, G., Desai, N.R., Joshi, P.H., Mehta, A., Karmally, W., Vani, A., Sitafalwalla, S.J. & Puri, R. (2021) 'Prevention of atherosclerotic cardiovascular disease in South Asians in the US: A clinical perspective from the National Lipid Association'. *Journal of Clinical Lipidology*

Kalter-Leibovici, O., Freimark, D., Freedman, L.S., Kaufman, G., Ziv, A., Murad, H., Benderly, M., Silverman, B.G., Friedman, N., Cukierman-Yaffe, T., Asher, E., Grupper, A., Goldman, D., Amitai, M., Matetzky, S., Shani, M., Silber, H. & Israel Heart Failure Disease Management Study (IHF-DMS) investigators (2017) 'Disease management in the treatment of patients with chronic heart failure who have universal access to health care: a randomized controlled trial.'. *BMC Medicine* **15** (1), 90

Kamel Boulos, M.N., Lou, R.C., Anastasiou, A., Nugent, C.D., Alexandersson, J., Zimmermann, G., Cortes, U. & Casas, R. (2009) 'Connectivity for healthcare and well-being management: examples from six European projects'. *International journal of environmental research and public health* **6** (7), 1947-1971

Kaner, J., Maestre, R., Lameski, P., Isaacson, M., Taveter, K., Tomesone, S., Maresova, P., Burnard, M. & Melero, F. (2019) 'SHELDON Smart habitat for the elderly.'.

Kang, H.G., Mahoney, D.F., Hoenig, H., Hirth, V.A., Bonato, P., Hajjar, I., Lipsitz, L.A. & Center for Integration of Medicine and Innovative Technology Working Group on Advanced Approaches to Physiologic Monitoring for the Aged (2010) 'In situ monitoring of health in older adults: technologies and issues.'. *Journal of the American Geriatrics Society* **58** (8), 1579-1586

Kang, Y. (2014) *Patient-related characteristics associated with rehospitalization in Medicare recipients with heart failure receiving telehomecare*. Heart FailureHome Health CareMedicareReadmissionTelehealthCross Sectional StudiesDecision TreesDescriptive StatisticsHumanMultiple Logistic RegressionNonexperimental StudiesOutcome AssessmentSecondary AnalysisSurvival Analysis, University of Pennsylvania

Kaplan, R.M. & Stone, A.A. (2013) 'Bringing the laboratory and clinic to the community: mobile technologies for health promotion and disease prevention'. *Annual Review of Psychology* **64**, 471-498

Kargul, W. (2009) '[Remote telemetric monitoring of patients with implanted ICD or ICD-CRT - technical novelty or clinical practical application?]'. *Kardiologia polska* **67** (5), 512

Karhula, T., Vuorinen, A.L., Rääpysjärvi, K., Pakanen, M., Itkonen, P., Tepponen, M., Junno, U.M., Jokinen, T., van Gils, M. & Lähteenmäki, J. (2015) 'Telemonitoring and Mobile Phone-Based Health Coaching Among Finnish Diabetic and Heart Disease Patients: randomized Controlled Trial'. *Journal of medical Internet research* **17** (6), e153

Karim, R.A., Zakaria, N.F., Zulkifley, M.A., Mustafa, M.M., Sagap, I. & Latar, N.H.M. (2013) 'Telepointer technology in telemedicine: a review'. *Biomedical engineering online* **12** (1), 1-19

Karimi-Shahanjarini, A., Shakibazadeh, E., Rashidian, A., Hajimiri, K., Glenton, C., Noyes, J., Lewin, S., Laurant, M. & Colvin, C.J. (2019) 'Barriers and facilitators to the implementation of doctor-nurse substitution strategies in primary care: a qualitative evidence synthesis'. *Cochrane Database of Systematic Reviews* (4)

Kario, K. (2018) 'Nocturnal hypertension: new technology and evidence'. *Hypertension* **71** (6), 997-1009

Kario, K. (2020) 'Management of Hypertension in the Digital Era: Small Wearable Monitoring Devices for Remote Blood Pressure Monitoring'. *Hypertension (0194911X)* **76** (3), 640-650

Kart, Ö., Mevsim, V., Kut, A., Yürek, İ., Altın, A.Ö. & Yılmaz, O. (2017) 'A mobile and web-based clinical decision support and monitoring system for diabetes mellitus patients in primary care: a study protocol for a randomized controlled trial'. *BMC medical informatics and decision making* **17** (1), 1-10

Katapally, T.R. (2019) 'The smart framework: integration of citizen science, community-based participatory research, and systems science for population health science in the digital age'. *JMIR mHealth and uHealth* **7** (8), e14056

Katra, R.P., Chakravarthy, N. & Libbus, I. (2011) 'Remote at-home detection and monitoring of functional chronotropic incompetence in heart failure patients.'. *Journal of Cardiovascular Translational Research* **4** (1), 14-20

Katzmarzyk, P.T. & Janssen, I. (2004) 'The economic costs associated with physical inactivity and obesity in Canada: an update'. *Canadian journal of applied physiology* **29** (1), 90-115

Kaufman, N., Clements, M. & Mel, E. (2021) 'Using Digital Health Technology to Prevent and Treat Diabetes'. *Diabetes Technology & Therapeutics* **23** (S2), S-85-S-102

Kauw, D., Huisma, P.R., Medlock, S.K., Koole, M.A.C., Wierda, E., Abu-Hanna, A., Schijven, M.P., Mulder, B.J.M., Bouma, B.J., Winter, M.M. & Schuuring, M.J. (2020) 'Mobile health in cardiac patients: an overview on experiences and challenges of stakeholders involved in daily use and development'. *Practical Neurology (BMJ Publishing Group)* **20** (5), 184-191

Kayikcioglu, I., Akdeniz, F., Kayikcioglu, T. & Kaya, I. (2017}) 'Real-Time Monitoring of ST Change for Telemedicine'. In Badnjevic, A. (eds.) *PROCEEDINGS OF THE INTERNATIONAL CONFERENCE ON MEDICAL AND BIOLOGICAL ENGINEERING 2017 (CMBEBIH 2017)},* Held 152 BEACH ROAD, \#21-01/04 GATEWAY EAST, SINGAPORE, 189721, SINGAPORE}: SPRINGER-VERLAG SINGAPORE PTE LTD}, 671-677}

Keat, Y.W. (2016) *Sustainable and mobility technologies for assistive healthcare and monitoring*

Keath, A. (2017) '# Physical Activity: Influencing Parent Behavior Change Through Social Media'.

Keikhosrokiani, P. (2021) 'IoT for Enhanced Decision-Making in Medical Information Systems: A Systematic Review'. *Enhanced Telemedicine and e-Health: Advanced IoT Enabled Soft Computing Framework* , 119-140

Keldsen, L.M. (2020) *Expanding Post-stroke Telerehabilitation: A Qualitative Study of User Experience Piloting VA Secure Messaging Use in a Telerehabilitation Format*

Kennedy, A.B. & Hales, S.B. (2018) 'Tools Clinicians Can Use to Help Get Patients Active'. *Current sports medicine reports* **17** (8), 271-276

Kepper, M., Walsh-Bailey, C., Staiano, A., Fowler, L.A., Gacad, A., Blackwood, A., Fowler, S.A. & Kelley, M. (2021) 'Health Information Technology Use Among Healthcare Providers Treating Children and Adolescents With Obesity: a Systematic Review'. *Current Epidemiology Reports* , 1-21

Kepper, M., Walsh-Bailey, C., Staiano, A., Fowler, L.A., Gacad, A., Blackwood, A., Fowler, S.A. & Kelley, M. (2021) 'Health Information Technology Use Among Healthcare Providers Treating Children and Adolescents With Obesity: a Systematic Review'. *Current Epidemiology Reports* , 1-21

Kerz, M., Folarin, A., Meyer, N., Begale, M., MacCabe, J. & Dobson, R.J. (2016}) 'SleepSight: A wearables-based relapse prevention system for Schizophrenia'. In Anonymous (eds.) *UBICOMP'16 ADJUNCT: PROCEEDINGS OF THE 2016 ACM INTERNATIONAL JOINT CONFERENCE ON PERVASIVE AND UBIQUITOUS COMPUTING},* Held 1515 BROADWAY, NEW YORK, NY 10036-9998 USA}: ASSOC COMPUTING MACHINERY}, 113-116}

Keytsman, C., Van Noten, P., Spaas, J., Nieste, I., Van Asch, P. & Eijnde, B.O. (2019) 'Periodized home-based training: A new strategy to improve high intensity exercise therapy adherence in mildly affected patients with Multiple Sclerosis'. *Multiple sclerosis and related disorders* **28**, 91-97

Keytsman, C., Van Noten, P., Spaas, J., Nieste, I., Van Asch, P. & Eijnde, B.O. (2019}) 'Periodized home-based training: A new strategy to improve high intensity exercise therapy adherence in mildly affected patients with Multiple Sclerosis'. *MULTIPLE SCLEROSIS AND RELATED DISORDERS}* **28}**, 91-97}

Keytsman, C., Van Noten, P., Spaas, J., Nieste, I., Van Asch, P. & Eijnde, B.O. (2019) 'Periodized home-based training: A new strategy to improve high intensity exercise therapy adherence in

mildly affected patients with Multiple Sclerosis.'. *Multiple Sclerosis and Related Disorders* **28**, 91-97

Khakurel, J. (2018) 'Enhancing the adoption of quantified self-tracking devices'.

Khalil, H., Bell, B., Chambers, H., Sheikh, A. & Avery, A.J. (2017) 'Professional, structural and organisational interventions in primary care for reducing medication errors'. *Cochrane Database of Systematic Reviews* (10)

Khan, N. (2020) 'Mobile Health Technology to Enhance Healthcare Service Delivery in Developing Nations (Saudi Arabia)'.

Khan, N., Qureshi, M., Mustapha, I., Irum, S. & Arshad, R. (2020) 'A systematic literature review paper on online medical mobile applications in Malaysia'.

Khera, A., Baum, S.J., Gluckman, T.J., Gulati, M., Martin, S.S., Michos, E.D., Navar, A.M., Taub, P.R., Toth, P.P. & Virani, S.S. (2020) 'Continuity of care and outpatient management for patients with and at high risk for cardiovascular disease during the COVID-19 pandemic: a scientific statement from the American Society for Preventive Cardiology'. *American Journal of Preventive Cardiology* **1**, 100009

Khoo, J. & Srinivasan, U. (2019) 'Harnessing the full spectrum of digital data to support the delivery of personalised services across the health care continuum'.

Khreiss, M., Zaarour, I. & Mcheick, H. (2016) 'SMILE: smart monitoring intelligent learning engine: an ontology-based context-aware system for supporting patients subjected to severe emergencies'. *International Journal of Healthcare Technology & Management* **15** (3), 194-209

Kidholm, K., Rasmussen, M.K., Andreasen, J.J., Hansen, J., Nielsen, G., Spindler, H. & Dinesen, B. (2016) 'Cost-Utility Analysis of a Cardiac Telerehabilitation Program: The Teledialog Project.'. *Telemedicine Journal & E-Health* **22** (7), 553-563

Kikuchi, A., Taniguchi, T., Nakamoto, K., Sera, F., Ohtani, T., Yamada, T. & Sakata, Y. (2021) 'Feasibility of home-based cardiac rehabilitation using an integrated telerehabilitation platform in elderly patients with heart failure: A pilot study.'. *Journal of cardiology* **78** (1), 66-71

Kim, B. (2020) 'PUSHING THE BOUNDARIES OF CONSUMER GRADE WEARABLE DEVICES IN HEALTH CARE FOR OLDER ADULTS'.

Kim, H., Cho, N., Kim, J., Kim, K.M., Kang, M., Choi, Y., Kim, M., You, H., Nam, S.I. & Shin, S. (2020) 'Implementation of a Home-Based mHealth App Intervention Program With Human Mediation for Swallowing Tongue Pressure Strengthening Exercises in Older Adults: Longitudinal Observational Study'. *JMIR mHealth and uHealth* **8** (10), e22080

Kirley, K. & Sachdev, N. (2018) 'Digital Health–Supported Lifestyle Change Programs to Prevent Type 2 Diabetes'. *Diabetes Spectrum* **31** (4), 303-309

Kirtava, Z., Gegenava, T., Gegenava, M., Matoshvili, Z., Kasradze, S. & Kasradze, P. (2012) 'Mobile telemonitoring for arrhythmias in outpatients in the Republic of Georgia: a brief report of a pilot study.'. *Telemedicine Journal & E-Health* **18** (7), 570-571

Kitsiou S., Pare G. & Jaana, M. (2015) 'Effects of home telemonitoring interventions on patients with chronic heart failure: an overview of systematic reviews.'. *Journal of medical Internet research* **17** (3), e63

Kitsiou, S., Gerber, B.S., Kansal, M.M., Buchholz, S.W., Chen, J., Ruppar, T., Arrington, J., Owoyemi, A., Leigh, J. & Pressler, S.J. (2021) 'Patient-centered mobile health technology intervention to improve self-care in patients with chronic heart failure: Protocol for a feasibility randomized controlled trial'. *Contemporary Clinical Trials* , 106433

Kitsiou, S., Pare, G. & Jaana, M. (2015}) 'Effects of Home Telemonitoring Interventions on Patients With Chronic Heart Failure: An Overview of Systematic Reviews'. *JOURNAL OF MEDICAL INTERNET RESEARCH}* **17}** (3})

Kivipelto, M., Mangialasche, F., Snyder, H.M., Allegri, R., Andrieu, S., Arai, H., Baker, L., Belleville, S., Brodaty, H. & Brucki, S.M. (2020) 'World-Wide FINGERS Network: A global approach to risk reduction and prevention of dementia'. *Alzheimer's & Dementia* **16** (7), 1078-1094

Klaas, V.C. (2018) *Monitoring outpatients in palliative care through wearable devices*

Klaassen, B., van Beijnum, B.J.F. & Hermens, H.J. (2016) 'Usability in telemedicine systems-A literature survey'. *International journal of medical informatics* **93**, 57-69

Klersy, C., De Silvestri, A., Gabutti, G., Regoli, F. & Auricchio, A. (2009) 'A meta-analysis of remote monitoring of heart failure patients'. *Journal of the American College of Cardiology* **54** (18), 1683-1694

Klersy, C., De Silvestri, A., Gabutti, G., Raisaro, A., Curti, M., Regoli, F. & Auricchio, A. (2011}) 'Economic impact of remote patient monitoring: an integrated economic model derived from a meta-analysis of randomized controlled trials in heart failure'. *EUROPEAN JOURNAL OF HEART FAILURE}* **13}** (4}), 450-459}

Klimis, H. & Chow, C.K. (2021) 'Are Digital Health Services the Key to Bridging the Gap in Medication Adherence and Optimisation?'. *Heart, lung & circulation* **30** (7), 943-946

Klinger, C.A., Howell, D., Marshall, D., Zakus, D., Brazil, K. & Deber, R.B. (2013}) 'Resource utilization and cost analyses of home-based palliative care service provision: The Niagara West End-of-Life Shared-Care Project'. *PALLIATIVE MEDICINE}* **27}** (2}), 115-122}

Klompmaker, F., Senft, B., Nebe, K., Busch, C. & Willemsen, D. (2011}) 'USER CENTERED DESIGN PROCESS OF OSAMI-D Developing User Interfaces for a Remote Ergometer Training Application'. In Traver, V., Fred, A., Filipe, J. and Gamboa, H. (eds.) *HEALTHINF 2011: PROCEEDINGS OF THE INTERNATIONAL CONFERENCE ON HEALTH INFORMATICS},* Held AV D MANUELL, 27A 2 ESQ, SETUBAL, 2910-595, PORTUGAL}: SCITEPRESS}, 268-273}

Kloosterman, E.M., Rosenbaum, M., La Starza, B., Wilcox, J. & Rosman, J. (2019) 'Remote Control of Cardiac Implantable Electronic Devices: Exploring the New Frontier-First Clinical Application of Real-time Remote-control Management of Cardiac Devices Before and After Magnetic Resonance Imaging.'. *The Journal of Innovations in Cardiac Rhythm Management* **10** (1), 3477-3484

Knight E., Stuckey M.I. & Petrella, R.J. (2013) 'Physical activity prescription and remote self-monitoring technologies: Can we reduce cardiovascular disease risk?.'. *Canadian Journal of Diabetes.Conference: 16th Annual Canadian Diabetes Association/Canadian Society of Endocrinology and Metabolism Professional Conference and Annual Meetings.Montreal, QC Canada.Conference Publication: (var.pagings)* **37** (SUPPL. 4), S48

Knight, E., Stuckey, M.I. & Petrella, R.J. (2013) 'Physical activity prescription and remote self-monitoring technologies: can we reduce cardiovascular disease risk?'. *Canadian journal of diabetes.* **37**, S48

Ko, S.Q., Hooi, B.M.Y., Koo, C.Y., Chor, D.W.P., Ling, Z.J., Chee, Y.L. & Jen, W.Y. (2020) 'Remote monitoring of marginalised populations affected by COVID-19: a retrospective review'. *BMJ open* **10** (12), e042647-2020-042647

Ko, S.Q., Hooi, B.M.Y., Koo, C., Chor, D.W.P., Ling, Z.J., Chee, Y. & Jen, W. (2020) 'Remote monitoring of marginalised populations affected by COVID-19: a retrospective review.'. *BMJ Open* **10** (12), e042647

Kobb, R., Hoffman, N., Lodge, R. & Kline, S. (2003) 'Enhancing elder chronic care through technology and care coordination: report from a pilot.'. *Telemedicine Journal & E-Health* **9** (2), 189-195

Koch, S. (2006) 'Home telehealth—current state and future trends'. *International journal of medical informatics* **75** (8), 565-576

Kodama, R., Arora, S., Anand, S., Choudhary, A., Weingarten, J., Francesco, N., Chiricolo, G., Silber, S. & Mehta, P.H. (2020) 'Reengineering the Discharge Transition Process of COVID-19 Patients Using Telemedicine, Remote Patient Monitoring, and Around-the-Clock Remote Patient Monitoring from the Emergency Department and Inpatient Units.'. *Telemedicine Journal & E-Health*

Koehler, F., Winkler, S., Schieber, M., Sechtem, U., Stangl, K., Boehm, M., Boll, H., Kim, S.S., Koehler, K., Luecke, S., Honold, M., Heinze, P., Schweizer, T., Braecklein, M., Kirwan, B., Gelbrich, G., Anker, S.D. & TIM-HF Investigators (2010}) 'Telemedical Interventional Monitoring in Heart Failure (TIM-HF), a randomized, controlled intervention trial investigating the impact of telemedicine on mortality in ambulatory patients with heart failure: study design'. *EUROPEAN JOURNAL OF HEART FAILURE}* **12}** (12}), 1354-1362}

Koekemoer, H.L. & Scheffer, C. (2008) 'Heart sound and electrocardiogram recording devices for telemedicine environments.'. *Annual International Conference Of The IEEE Engineering In Medicine And Biology Society* **2008**, 4867-4870

Koepp, J., Baron, M.V., Martins, P.R.H., Brandenburg, C., Kira, A.T.F., Trindade, V.D., Dominguez, L.M.L., Carneiro, M., Frozza, R. & Possuelo, L.G. (2020) 'The Quality of Mobile Apps Used for the Identification of Pressure Ulcers in Adults: Systematic Survey and Review of Apps in App Stores'. *JMIR mHealth and uHealth* **8** (6), e14266

Kogutt, B.K. & Sheffield, J.S. (2020) 'Feasibility of remote monitoring to confirm exercise adherence in high risk pregnant women'. *Obstetrics and gynecology* **135**, 149S-

Kolominsky-Rabas, P.L., Kriza, C., Djanatliev, A., Meier, F., Uffenorde, S., Radeleff, J., Baumgartel, P., Leb, I., Sedlmayr, M., Gaiser, S. & Adamson, P.B. (2016) 'Health Economic Impact of a Pulmonary Artery Pressure Sensor for Heart Failure Telemonitoring: A Dynamic Simulation.'. *Telemedicine Journal & E-Health* **22** (10), 798-808

Kolt G.S., Schofield G.M., Kerse N., Garrett N., Schluter P.J., Ashton T. & Patel, A. (2009) 'The healthy steps study: A randomized controlled trial of a pedometer-based green prescription for older adults. Trial protocol.'. *BMC Public Health* **9** (pagination), Arte Number: 404. ate of Pubaton: 2009

Kondo, M.C., Oyekanmi, K.O., Gibson, A., South, E.C., Bocarro, J. & Hipp, J.A. (2020) 'Nature prescriptions for health: A review of evidence and research opportunities'. *International journal of environmental research and public health* **17** (12), 4213

Kongkaew, C., Methaneethorn, J., Mongkhon, P., Dechanont, S. & Taburee, W. (2021) 'Drug-Related Problems Identified at Patients' Home: A Prospective Observational Study in a Rural Area of Thailand'. *Journal of Patient Safety* **17** (1), 8-14

Korc-Grodzicki, B., Holmes, H.M. & Shahrokni, A. (2015) 'Geriatric assessment for oncologists'. *Cancer biology & medicine* **12** (4), 261-274

Kornej, J., Börschel, C.S., Benjamin, E.J. & Schnabel, R.B. (2020) 'Epidemiology of atrial fibrillation in the 21st century: novel methods and new insights'. *Circulation research* **127** (1), 4-20

Kornowski, R., Zlochiver, S., Botzer, L., Tirosh, R., Abboud, S. & Misan, S. (2003) 'Validation of vital signs recorded via a new telecare system.'. *Journal of Telemedicine & Telecare* **9** (6), 328-333

Koshimizu, H., Kojima, R. & Okuno, Y. (2020) 'Future possibilities for artificial intelligence in the practical management of hypertension'. *Hypertension Research* , 1-11

Kosulwat, V. (2002) 'The nutrition and health transition in Thailand'. *Public health nutrition* **5** (1), 183-189

Kovacs, F., Torok, M., Horvath, C., Balogh, A.T., Zsedrovits, T., Nagy, A. & Hosszu, G. (2010) 'A new, phonocardiography-based telemetric fetal home monitoring system.'. *Telemedicine Journal & E-Health* **16** (8), 878-882

Koydemir, H.C. & Ozcan, A. (2018) 'Wearable and implantable sensors for biomedical applications'. *Annual Review of Analytical Chemistry* **11**, 127-146

Krakoff, L.R. (2011}) 'Management of Cardiovascular Risk Factors Is Leaving the Office: Potential Impact of Telemedicine'. *JOURNAL OF CLINICAL HYPERTENSION}* **13}** (11}), 791-794}

Krakoff, L.R. (2011) 'Management of cardiovascular risk factors is leaving the office: potential impact of telemedicine.'. *Journal of clinical hypertension* **13** (11), 791-794

Kramer, C.V., Zhang, F., Sinclair, D. & Olliaro, P.L. (2014) 'Drugs for treating urinary schistosomiasis'. *Cochrane Database of Systematic Reviews* (8)

Kramer, F., Butler, J., Shah, S.J., Jung, C., Nodari, S., Rosenkranz, S., Senni, M., Bamber, L., Cichos, S., Dori, C., Karakoyun, T., Kohler, G.J., Patel, K., Piraino, P., Viethen, T., Chennuru, P., Paydar, A., Sims, J., Clark, R., van Lummel, R., Muller, A., Gwaltney, C., Smajlovic, S., Dungen, H. & Dinh, W. (2020) 'Real-Life Multimarker Monitoring in Patients with Heart Failure: Continuous Remote Monitoring of Mobility and Patient-Reported Outcomes as Digital End Points in Future Heart-Failure Trials.'. *Digital Biomarkers* **4** (2), 45-59

Kramer, M.S. & Kakuma, R. (2012) 'Optimal duration of exclusive breastfeeding'. *Cochrane Database of Systematic Reviews* (8)

Kredo, T., Ford, N., Adeniyi, F.B. & Garner, P. (2013) 'Decentralising HIV treatment in lower- and middle-income countries'. *Cochrane Database of Systematic Reviews* (6)

Krep, H., Bottiger, B.W., Bock, C., Kerskens, C.M., Radermacher, B., Fischer, M., Hoehn, M. & Hossmann, K.A. (2003) 'Time course of circulatory and metabolic recovery of cat brain after cardiac arrest assessed by perfusion- and diffusion-weighted imaging and MR-spectroscopy.'. *Resuscitation* **58** (3), 337-348

Krishnaswami, A., Beavers, C., Dorsch, M.P., Dodson, J.A., Masterson Creber, R., Kitsiou, S., Goyal, P., Maurer, M.S., Wenger, N.K. & Croy, D.S. (2020) 'Gerotechnology for Older Adults With Cardiovascular Diseases: JACC State-of-the-Art Review'. *Journal of the American College of Cardiology* **76** (22), 2650-2670

Kristjansson, B., Petticrew, M., MacDonald, B., Krasevec, J., Janzen, L., Greenhalgh, T., Wells, G.A., MacGowan, J., Farmer, A.P. & Shea, B. (2007) 'School feeding for improving the physical and psychosocial health of disadvantaged students'. *Cochrane Database of Systematic Reviews* (1)

Kristjansson, E., Francis, D.K., Liberato, S., Benkhalti Jandu, M., Welch, V., Batal, M., Greenhalgh, T., Rader, T., Noonan, E. & Shea, B. (2015) 'Food supplementation for improving the physical and psychosocial health of socio-economically disadvantaged children aged three months to five years'. *Cochrane Database of Systematic Reviews* (3)

Kruizinga, M.D., Heide, N.v.d., Moll, A., Zhuparris, A., Yavuz, Y., Kam, M.L.d., Stuurman, F.E., Cohen, A.F. & Driessen, G.J.A. (2021) 'Towards remote monitoring in pediatric care and clinical trials-Tolerability, repeatability and reference values of candidate digital endpoints derived from physical activity, heart rate and sleep in healthy children.'. *PLoS ONE [Electronic Resource]* **16** (1), e0244877

Krzesinski, P., Sobotnicki, A., Gacek, A., Siebert, J., Walczak, A., Murawski, P. & Gielerak, G. (2021) 'Noninvasive Bioimpedance Methods From the Viewpoint of Remote Monitoring in Heart Failure.'. *JMIR MHealth and UHealth* **9** (5), e25937

Kubicek, J., Fiedorova, K., Vilimek, D., Cerny, M., Penhaker, M., Janura, M. & Rosicky, J. (2020) 'Recent Trends, Construction and Applications of Smart Textiles and Clothing for Monitoring of Health Activity: A Comprehensive Multidisciplinary Review'. *IEEE Reviews in Biomedical Engineering*

Kulshreshtha, A., Kvedar, J.C., Goyal, A., Halpern, E.F. & Watson, A.J. (2010) 'Use of remote monitoring to improve outcomes in patients with heart failure: a pilot trial'. *International journal of telemedicine and applications*

Kümm, A.J. (2018) *Feasibility of a smartphone application to identify young children at risk for Autism Spectrum Disorder in a low-income community setting in South Africa*

Kuras, E.R., Richardson, M.B., Calkins, M.M., Ebi, K.L., Hess, J.J., Kintziger, K.W., Jagger, M.A., Middel, A., Scott, A.A. & Spector, J.T. (2017) 'Opportunities and challenges for personal heat exposure research'. *Environmental health perspectives* **125** (8), 085001

Kutyifa, V., Zima, E., Molnar, L., Kuehne, C., Theiss, S., Herrmann, G., Geller, L. & Merkely, B. (2013) 'Direct comparison of steroid and non-steroid eluting small surface pacing leads: randomized, multicenter clinical trial.'. *Cardiology Journal* **20** (4), 431-438

Kwan, K.P. (2019) *Examining and investigating home modifications and smart home technologies to reduce fall injury among older adults.*

Lakshmi Dhevi, B., Vishvaksenan, K.S., Senthamil Selvan, K. & Rajalakshmi, A. (2018) 'Patient Monitoring System Using Cognitive Internet of Things.'. *Journal of medical systems* **42** (11), 229

Lamaison, D., Motreff, P., Jean, F., Geoffroy, E., Rodriguez, R., Souteyrand, G., Chanseaume, S., Terrazoni, S. & Cassagnes, J. (2004) '[The place of telemedicine in rhythmology and cardiac pacing]'. *Archives des Maladies du Coeur et des Vaisseaux* **97** (11), 1160-1164

Lamberigts, M., Van Hoof, L., Proesmans, T., Vandervoort, P., Grieten, L., Haemers, P. & Rega, F. (2021) 'Remote Heart Rhythm Monitoring by Photoplethysmography-Based Smartphone Technology After Cardiac Surgery: Prospective Observational Study'. *JMIR mHealth and uHealth* **9** (4), e26519

Lancellotti, P. (2017) 'Left ventricular filling pressure, diastolic function, and heart rate'. *Dialogues in Cardiovascular Medicine* **23** (1), 83-87

Lancellotti, P. (2017) 'Severe secondary mitral regurgitation in heart failure: A holistic approach'. *Dialogues in Cardiovascular Medicine* **22** (3), 19-20

Langford, R., Bonell, C.P., Jones, H.E., Pouliou, T., Murphy, S.M., Waters, E., Komro, K.A., Gibbs, L.F., Magnus, D. & Campbell, R. (2014) 'The WHO Health Promoting School framework for improving the health and well-being of students and their academic achievement'. *Cochrane Database of Systematic Reviews* (4)

Langhorne, P. & Baylan, S. (2017) 'Early supported discharge services for people with acute stroke'. *Cochrane Database of Systematic Reviews* (7)

Lappa, A. & Goumopoulos, C. (2017}) 'A Home-based Early Risk Detection System for Congestive Heart Failure using a Bayesian Reasoning Network'. In Rocker, C., ODonoghue, J., Ziefle, M., Maciaszek, L. and Molloy, W. (eds.) *PROCEEDINGS OF THE 3RD INTERNATIONAL CONFERENCE ON INFORMATION AND COMMUNICATION TECHNOLOGIES FOR AGEING WELL AND E-HEALTH (ICT4AWE), VOL 1},* Held AV D MANUELL, 27A 2 ESQ, SETUBAL, 2910-595, PORTUGAL}: SCITEPRESS}, 58-69}

Laranjo, L., Lau, A., Oldenburg, B., Gabarron, E., O'Neill, A., Chan, S. & Coiera, E. (2015) 'mHealth technologies for chronic disease prevention and management'.

Larsen, C. (2020) 'Piloting a Smartphone-Based Sedentary Behavior Reduction Intervention for Adults With Overweight or Obesity: Take a STAND 4 Health'.

Lassi, Z.S. & Bhutta, Z.A. (2015) 'Community-based intervention packages for reducing maternal and neonatal morbidity and mortality and improving neonatal outcomes'. *Cochrane Database of Systematic Reviews* (3)

Lavallee, D.C., Chenok, K.E., Love, R.M., Petersen, C., Holve, E., Segal, C.D. & Franklin, P.D. (2016) 'Incorporating patient-reported outcomes into health care to engage patients and enhance care'. *Health affairs* **35** (4), 575-582

Laver, K.E., Adey-Wakeling, Z., Crotty, M., Lannin, N.A., George, S. & Sherrington, C. (2020) 'Telerehabilitation services for stroke'. *Cochrane Database of Systematic Reviews* (1)

Lavie, C.J., Milani, R.V. & Ventura, H.O. (2009) 'Obesity and cardiovascular disease: risk factor, paradox, and impact of weight loss'. *Journal of the American College of Cardiology* **53** (21), 1925-1932

Lavon, O. & Sagi, R. (2013) '[Cholinesterase inhibitor poisoning: a complicated medical challenge]'. *Harefuah* **152** (7), 395-397

Lawrenson, J.G., Graham-Rowe, E., Lorencatto, F., Burr, J., Bunce, C., Francis, J.J., Aluko, P., Rice, S., Vale, L. & Peto, T. (2018) 'Interventions to increase attendance for diabetic retinopathy screening'. *Cochrane Database of Systematic Reviews* (1)

Lazarus, A. (2007) 'Remote, wireless, ambulatory monitoring of implantable pacemakers, cardioverter defibrillators, and cardiac resynchronization therapy systems: analysis of a worldwide database.'. *Pacing & Clinical Electrophysiology* **30** (Suppl 1), S2-S12

Leahy, R.A. & Davenport, E.E. (2015) 'Home Monitoring for Cardiovascular Implantable Electronic Devices: Benefits to Patients and to Their Follow-up Clinic'. *AACN Advanced Critical Care* **26** (4), 343-355

LeBaron, V., Hayes, J., Gordon, K., Alam, R., Homdee, N., Martinez, Y., Ogunjirin, E., Thomas, T., Jones, R. & Blackhall, L. (2019) 'Leveraging smart health technology to empower patients and family caregivers in managing cancer pain: protocol for a feasibility study'. *JMIR research protocols* **8** (12), e16178

Lee, J.H., Park, Y.R., Kweon, S., Kim, S., Ji, W. & Choi, C. (2018) 'A Cardiopulmonary Monitoring System for Patient Transport Within Hospitals Using Mobile Internet of Things Technology: Observational Validation Study'. *JMIR mHealth and uHealth* **6** (11), e12048

Lee, J.Y., Wong, C.P. & Lee, S.W.H. (2020) 'm-Health views and perception among Malaysian: findings from a survey among individuals living in Selangor'. *MHealth* **6**

Lefler, L.L., Rhoads, S.J., Harris, M., Funderburg, A.E., Lubin, S.A., Martel, I.D., Faulkner, J.L., Rooker, J.L., Bell, D.K., Marshall, H. & Beverly, C.J. (2018) 'Evaluating the Use of Mobile Health Technology in Older Adults With Heart Failure: Mixed-Methods Study.'. *24058297* **1** (2), e12178; e 04

Legg, L.A., Quinn, T.J., Mahmood, F., Weir, C.J., Tierney, J., Stott, D.J., Smith, L.N. & Langhorne, P. (2011) 'Non-pharmacological interventions for caregivers of stroke survivors'. *Cochrane Database of Systematic Reviews* (10)

Leng Chow, W., Aung, C., Tong, S.C., Goh, G.S., Lee, S., MacDonald, M.R., Ng, A.N., Cao, Y., Ahmad, A.E. & Yap, M.F. (2020) 'Effectiveness of telemonitoring-enhanced support over structured telephone support in reducing heart failure-related healthcare utilization in a multi-ethnic Asian setting'. *Journal of telemedicine and telecare* **26** (6), 332-340

Leng Chow, W., Aung, C.Y.K., Tong, S.C., Goh, G.S.L., Lee, S., MacDonald, M.R., Ng, A.N.K., Cao, Y., Ahmad, A.E., Yap, M.F., Leong, G., Bruege, A., Tesanovic, A., Riistama, J., Pang, S.Y. & Erazo, F. (2020) 'Effectiveness of telemonitoring-enhanced support over structured telephone support in reducing heart failure-related healthcare utilization in a multi-ethnic Asian setting'. *Journal of Telemedicine & Telecare* **26** (6), 332-340

Leon, N., Balakrishna, Y., Hohlfeld, A., Odendaal, W.A., Schmidt, B., Zweigenthal, V., Anstey Watkins, J. & Daniels, K. (2020) 'Routine Health Information System (RHIS) improvements for strengthened health system management'. *Cochrane Database of Systematic Reviews* (8)

Leroux, A., Rzasa-Lynn, R., Crainiceanu, C. & Sharma, T. (2021) 'Wearable Devices: Current Status and Opportunities in Pain Assessment and Management'. *Digital Biomarkers* **5** (1), 89-102

Leung, A.Y., Chau, P.H., Leung, I.S., Tse, M., Wong, P.L., Tam, W.M. & Leung, D.Y. (2019) 'Motivating diabetic and hypertensive patients to engage in regular physical activity: A multi-component intervention derived from the concept of photovoice'. *International journal of environmental research and public health* **16** (7), 1219

Leung, R., Guo, H. & Pan, X. (2018) 'Social media users' perception of telemedicine and mHealth in China: Exploratory study'. *JMIR mHealth and uHealth* **6** (9), e181

Levin, S.R., Dittus, R., Aronsky, D., Weinger, M.B., Han, J., Boord, J. & France, D. (2008) 'Optimizing cardiology capacity to reduce emergency department boarding: a systems engineering approach.'. *American Heart Journal* **156** (6), 1202-1209

Levine, D.M., Pian, J., Mahendrakumar, K., Saenz, A. & Schnipper, J.L. (2019) 'Hospital-level care at home for acutely ill adults: a qualitative evaluation of a randomized controlled trial'. *Journal of general internal medicine* **34** (2), S241-S242

Lewalter, T. & Brodherr, T. (2015) 'Remote monitoring of implantable cardioverter-defibrillators: financial impact for providers and benefits to patients.'. *European heart journal* **36** (3), 143-144

Lewinski, A.A., Bosworth, H.B., Goldstein, K.M., Gierisch, J.M., Jazowski, S., McCant, F., White-Clark, C., Smith, V.A. & Zullig, L.L. (2021) 'Improving cardiovascular outcomes by using team-supported, EHR-leveraged, active management: Disseminating a successful quality improvement project.'. *Contemporary Clinical Trials Communications* **21**, 100705

Lewis, B.A., Napolitano, M.A., Buman, M.P., Williams, D.M. & Nigg, C.R. (2017) 'Future directions in physical activity intervention research: expanding our focus to sedentary behaviors, technology, and dissemination'. *Journal of Behavioral Medicine* **40** (1), 112-126

Li, C., Neugroschl, J., Zhu, C.W., Aloysi, A., Schimming, C.A., Cai, D., Grossman, H., Martin, J., Sewell, M. & Loizos, M. (2020) 'Design Considerations for Mobile Health Applications Targeting Older Adults'. *Journal of Alzheimer's Disease* (Preprint), 1-8

Li, F. (2021) 'Design of an Interactive Two-Way Telemedicine Service System for Smart Home Care for the Elderly'. *Journal of Healthcare Engineering* **2021**

Li, J., Rapin, J., Rosier, A., Smith, S., Fleureau, Y., Taboulet, P. & Groupe, G. (2016) 'European Congress on eCardiology & eHealth October 2016, Selected Abstracts'. *Cardiology* **23** (2S), 41-55

Li, L., Huang, J., Wu, J., Jiang, C., Chen, S., Xie, G., Ren, J., Tao, J., Chan, C.C. & Chen, L. (2020) 'A mobile health app for the collection of functional outcomes after inpatient stroke rehabilitation: Pilot randomized controlled trial'. *JMIR mHealth and uHealth* **8** (5), e17219

Li, J., Yang, P., Fu, D., Ye, X., Zhang, L., Chen, G., Yang, Y., Luo, H., Chen, L. & Shao, M. (2019) 'Effects of home-based cardiac exercise rehabilitation with remote electrocardiogram monitoring in patients with chronic heart failure: a study protocol for a randomised controlled trial'. *BMJ open* **9** (3)

Liachenko, S., Tang, P., Hamilton, R.L. & Xu, Y. (1998) 'A reproducible model of circulatory arrest and remote resuscitation in rats for NMR investigation'. *Stroke (00392499)* **29** (6), 1229-1238

Liao, Y., Bang, D., Cosgrove, S., Dulin, R., Harris, Z., Taylor, A., White, S., Yatabe, G., Liburd, L., Giles, W., Division of Adult and Community Health,National Center for Chronic Disease Prevention and Health Promotion & Centers for Disease Control and Prevention (CDC) (2011) 'Surveillance of health status in minority communities - Racial and Ethnic Approaches to Community Health Across the U.S. (REACH U.S.) Risk Factor Survey, United States, 2009.'. *Morbidity & Mortality Weekly Report.Surveillance Summaries* **60** (6), 1-44

Liao, Y., Tucker, P., Okoro, C.A., Giles, W.H., Mokdad, A.H., Harris, V.B. & Division of Adult and Community Health, National Center for Chronic Disease Prevention and Health Promotion,Centers for Disease Control and Prevention (CDC) (2004) 'REACH 2010 Surveillance for Health Status in Minority Communities --- United States, 2001--2002.'. *Morbidity & Mortality Weekly Report.Surveillance Summaries* **53** (6), 1-36

Liaw, S., Ansari, S., Jonnagaddala, J., Narasimhan, P., Ashraf, M.M. & Harris, M. (2018) 'A review of the use of mHealth to promote healthy ageing and support the delivery of age-friendly health and long-term care services'.

Liberska, A., Kowalski, O., Mazurek, M., Lenarczyk, R., Jedrzejczyk-Patej, E., Przybylska-Siedlecka, K., Koziel, M., Morawski, S., Podolecki, T., Kowalczyk, J., Pruszkowska, P., Pluta, S., Sokal, A. & Kalarus, Z. (2016) 'Day by day telemetric care of patients treated with cardiac resynchronisation therapy: first Polish experience.'. *Kardiologia polska* **74** (8), 741-748

Liew, S.M., Abdullah, A., Abdullah, N. & Chia, Y.C. (2013) 'Health innovation in cardiovascular diseases.'. *The Australasian Medical Journal* **6** (1), 67-69

Lim, W., Liang, C., Assantachai, P., Auyeung, T.W., Kang, L., Lee, W., Lim, J., Sugimoto, K., Akishita, M. & Chia, S. (2020) 'COVID-19 and older people in Asia: Asian Working Group for Sarcopenia calls to action'. *Geriatrics & gerontology international* **20** (6), 547-558

Lin, B., Wong, A.M. & Tseng, K.C. (2016) 'Community-based ECG monitoring system for patients with cardiovascular diseases'. *Journal of medical systems* **40** (4), 80

Lin, B., Prickett, C. & Woltering, S. (2021) 'Feasibility of using a biofeedback device in mindfulness training-a pilot randomized controlled trial'. *Pilot and feasibility studies* **7** (1), 1-13

Lin, W., Wen, J. & Chen, G. (2020) 'The clinical outcomes and effectiveness of mHealth interventions for diabetes and hypertension: a systematic review and meta-analysis'. *medRxiv*

Lin, P.H., Intille, S., Bennett, G., Bosworth, H.B., Corsino, L., Voils, C., Grambow, S., Lazenka, T., Batch, B.C. & Tyson, C. (2015) 'Adaptive intervention design in mobile health: intervention design and development in the Cell Phone Intervention for You trial'. *Clinical trials (London, England)* **12** (6), 634-645

Lin, W., Ma, X., Deng, D. & Li, Y. (2015) 'Hemodynamics in the Circle of Willis with Internal Carotid Artery Stenosis under Cervical Rotatory Manipulation: A Finite Element Analysis.'. *Medical Science Monitor* **21**, 1820-1826

Lindegren, M.L., Kennedy, C.E., Bain-Brickley, D., Azman, H., Creanga, A.A., Butler, L.M., Spaulding, A.B., Horvath, T. & Kennedy, G.E. (2012) 'Integration of HIV/AIDS services with maternal, neonatal and child health, nutrition, and family planning services'. *Cochrane Database of Systematic Reviews* (9)

Linder, S.M., Rosenfeldt, A.B., Reiss, A., Buchanan, S., Sahu, K., Bay, C.R., Wolf, S.L. & Alberts, J.L. (2013) 'The home stroke rehabilitation and monitoring system trial: a randomized controlled trial'. *International journal of stroke* **8** (1), 46-53

Lindfors, P. (2020) 'User Interface Design and Development Process for Cyber Therapy Device'.

Lindson, N., Thompson, T.P., Ferrey, A., Lambert, J.D. & Aveyard, P. (2019) 'Motivational interviewing for smoking cessation'. *Cochrane Database of Systematic Reviews* (7)

Linkous, J.D. (2002) 'Telemedicine: an overview.'. *Journal of Medical Practice Management* **18** (1), 24-27

Linn, N., Goetzinger, C., Regnaux, J., Schmitz, S., Dessenne, C., Fagherazzi, G. & Aguayo, G.A. (2021) 'Digital Health Interventions Among People Living With Frailty: A Scoping Review'. *Journal of the American Medical Directors Association*

Liu, S., Weismiller, J., Strange, K., Forster-Coull, L., Bradbury, J., Warshawski, T. & Naylor, P. (2020) 'Evaluation of the scale-up and implementation of mind, exercise, nutrition… do it!(MEND) in British Columbia: a hybrid trial type 3 evaluation'. *BMC pediatrics* **20** (1), 1-11

Liu, D., Xu, F., Li, Y. & Zhang, Y. (2019) 'Home blood pressure tele-monitoring for hypertension management: advantages and prospects'. *Chinese journal of evidence-based medicine* **19** (11), 1349-1352

Liu, H.H., Ezekowitz, M.D., Columbo, M., Khan, O., Martin, J., Spahr, J., Yaron, D., Cushinotto, L. & Kapelusznik, L. (2021) 'Testing the feasibility of operationalizing a prospective, randomized trial with remote cardiac safety EKG monitoring during a pandemic.'. *Journal of Interventional Cardiac Electrophysiology*

Liu, J.P., Zhang, M., Wang, W. & Grimsgaard, S. (2002) 'Chinese herbal medicines for type 2 diabetes mellitus'. *Cochrane Database of Systematic Reviews* (3)

Liu, Q., Abba, K., Alejandria, M.M., Sinclair, D., Balanag, V.M. & Lansang, M. (2014) 'Reminder systems to improve patient adherence to tuberculosis clinic appointments for diagnosis and treatment'. *Cochrane Database of Systematic Reviews* (11)

Lloyd, T., Buck, H., Foy, A., Black, S., Pinter, A., Pogash, R., Eismann, B., Balaban, E., Chan, J., Kunselman, A., Smyth, J. & Boehmer, J. (2019) 'The Penn State Heart Assistant: A pilot study of a web-based intervention to improve self-care of heart failure patients'. *Health informatics journal* **25** (2), 292-303

Lloyd, T., Buck, H., Foy, A., Black, S., Pinter, A., Pogash, R., Eismann, B., Balaban, E., Chan, J., Kunselman, A., Smyth, J. & Boehmer, J. (2019) 'The Penn State Heart Assistant: A pilot study of a web-based intervention to improve self-care of heart failure patients.'. *Health Informatics Journal* **25** (2), 292-303

Locati, E.T. & Lunati, M. (2014) 'Effectiveness of remote monitoring of cardiac implantable electronic devices in detection and treatment of clinical and device-related cardiovascular events in daily practice: the HomeGuide Registry...Europace. 2013 Jul;15(7):970-7'. *EP: Europace* **16** (7), 1099-1099

Locati, E.T., Vargiu, S., Mulargia, E., Ardito, C., Schirru, M., Pedretti, S., Negrini, F. & Lunati, M. (2012) '[New implantable devices for patient management: role and perspectives of remote monitoring of implantable cardioverter-defibrillators]'. *Giornale italiano di cardiologia* **13** (10 Suppl 2), 36S-40S

Lopes, Marcelo Antônio Cartaxo Queiroga, Oliveira, Gláucia Maria Moraes de, Ribeiro, A.L.P., Pinto, F.J., Rey, H.C.V., Zimerman, L.I., Rochitte, C.E., Bacal, F., Polanczyk, C.A. & Halperin, C. (2019) 'Guideline of the Brazilian Society of Cardiology on Telemedicine in Cardiology-2019'. *Arquivos Brasileiros de Cardiologia* **113** (5), 1006-1056

Lopez, L.M., Grey, T.W., Chen, M., Tolley, E.E. & Stockton, L.L. (2016) 'Theory-based interventions for contraception'. *Cochrane Database of Systematic Reviews* (11)

Lopez-Villegas, A., Catalan-Matamoros, D., Robles-Musso, E., Bautista-Mesa, R. & Peiro, S. (2019) 'Cost-utility analysis on telemonitoring of users with pacemakers: The PONIENTE study.'. *Journal of Telemedicine & Telecare* **25** (4), 204-212

Loring, P.A., Duffy, L.K. & Murray, M.S. (2010) 'A risk-benefit analysis of wild fish consumption for various species in Alaska reveals shortcomings in data and monitoring needs.'. *Science of the Total Environment* **408** (20), 4532-4541

Lounaskorpi, E. (2019) 'Developing new VR possibilities for the Sports and Fitness Sector in Finland'.

Loveday, A., Sherar, L.B., Sanders, J.P., Sanderson, P.W. & Esliger, D.W. (2015) 'Technologies that assess the location of physical activity and sedentary behavior: a systematic review'. *Journal of medical Internet research* **17** (8), e192

Loveman, E., Royle, P. & Waugh, N. (2003) 'Specialist nurses in diabetes mellitus'. *Cochrane Database of Systematic Reviews* (2)

Lovo Grona, S. (2018) *Advancing Interprofessional Primary Health Care Services in Rural Settings for People with Chronic Low Back Disorders: A Team and Technology Approach*

Lu, H., Fang, G. & Wu, Y. (2006) 'Estimating the frequency distributions of particulate matter and their metal elements in a temple.'. *Journal of the Air & Waste Management Association* **56** (7), 1033-1040

Lu, J.F., Chen, C.M. & Hsu, C.Y. (2019) 'Effect of home telehealth care on blood pressure control: A public healthcare centre model'. *Journal of telemedicine and telecare* **25** (1), 35-45

Lucas, P., McIntosh, K., Petticrew, M., Roberts, H.M. & Shiell, A. (2008) 'Financial benefits for child health and well-being in low income or socially disadvantaged families in developed world countries'. *Cochrane Database of Systematic Reviews* (2)

Luiza, V.L., Chaves, L.A., Silva, R.M., Emmerick, I., Chaves, G.C., Fonseca de Araújo, S., Moraes, E.L. & Oxman, A.D. (2015) 'Pharmaceutical policies: effects of cap and co-payment on rational use of medicines'. *Cochrane Database of Systematic Reviews* (5)

Lujan, H.L. & DiCarlo, S.E. (2013) 'Cardiac output, at rest and during exercise, before and during myocardial ischemia, reperfusion, and infarction in conscious mice.'. *American Journal of Physiology - Regulatory Integrative & Comparative Physiology* **304** (4), R286-95

Lupton, D. (2021) 'Young People’s Use of Digital Health Technologies in the Global North: Narrative Review'. *Journal of Medical Internet Research* **23** (1), e18286

Luscher, T.F. (2015) 'Arrhythmias and pacing.'. *European heart journal* **36** (3), 131-132

Luthje, L., Vollmann, D., Seegers, J., Dorenkamp, M., Sohns, C., Hasenfuss, G. & Zabel, M. (2011) 'Remote magnetic versus manual catheter navigation for circumferential pulmonary vein ablation in patients with atrial fibrillation.'. *Clinical Research in Cardiology* **100** (11), 1003-1011

Lv, Q., Jiang, Y., Qi, J., Zhang, Y., Zhang, X., Fang, L., Tu, L., Yang, M., Liao, Z. & Zhao, M. (2019) 'Using mobile apps for health management: a new health care mode in China'. *JMIR mHealth and uHealth* **7** (6), e10299

Lyamina N., Kotelnikova E., Senchikhin V. & Lipchanskaya, T. (2018) 'Remote model in the system of outpatient cardioreabilitation in subjects with chronic heart failure.'. *European Journal of Heart Failure* **Conference**, Heart

M Bublitz, F., Oetomo, A., S Sahu, K., Kuang, A., X Fadrique, L., E Velmovitsky, P., M Nobrega, R. & P Morita, P. (2019) 'Disruptive technologies for environment and health research: an overview of artificial intelligence, blockchain, and internet of things'. *International journal of environmental research and public health* **16** (20), 3847

Ma, J., Li, Y., Liu, Y., Wang, X., Lin, C. & Cheng, H. (2020) 'Metal(loid) bioaccessibility and children's health risk assessment of soil and indoor dust from rural and urban school and residential areas.'. *Environmental Geochemistry & Health* **42** (5), 1291-1303

Mabote, T., Wong, K. & Cleland, J.G.F. (2014) 'The utility of novel non-invasive technologies for remote hemodynamic monitoring in chronic heart failure.'. *Expert Review of Cardiovascular Therapy* **12** (8), 923-928

MacArthur, G., Caldwell, D.M., Redmore, J., Watkins, S.H., Kipping, R., White, J., Chittleborough, C., Langford, R., Er, V. & Lingam, R. (2018) 'Individual-, family-, and school-level interventions targeting multiple risk behaviours in young people'. *Cochrane Database of Systematic Reviews* (10)

Mackintosh, M.A., Niehaus, J., Taft, C.T., Marx, B.P., Grubbs, K. & Morland, L.A. (2017) 'Using a Mobile Application in the Treatment of Dysregulated Anger Among Veterans'. *Military medicine* **182** (11), e1941-e1949

Mackintosh, M., Niehaus, J., Taft, C.T., Marx, B.P., Grubbs, K. & Morland, L.A. (2017) 'Using a Mobile Application in the Treatment of Dysregulated Anger Among Veterans.'. *Military medicine* **182** (11), e1941-e1949

Mackintosh, N.J., Davis, R.E., Easter, A., Rayment-Jones, H., Sevdalis, N., Wilson, S., Adams, M. & Sandall, J. (2020) 'Interventions to increase patient and family involvement in escalation of care for acute life-threatening illness in community health and hospital settings'. *Cochrane Database of Systematic Reviews* (12)

Maddison R., Rawstorn J.C., Rolleston A., Whittaker R., Stewart R., Benatar J., Warren I., Jiang Y. & Gant, N. (2014) 'The remote exercise monitoring trial for exercise-based cardiac rehabilitation (REMOTE-CR): a randomised controlled trial protocol.'. *BMC public health* **14**, 1236

Maddison, R., Rawstorn, J.C., Stewart, R.A.H., Benatar, J., Whittaker, R., Rolleston, A., Jiang, Y., Gao, L., Moodie, M., Warren, I., Meads, A. & Gant, N. (2019) 'Effects and costs of real-time cardiac telerehabilitation: randomised controlled non-inferiority trial.'. *Heart* **105** (2), 122-129

Maggio, M.G., Naro, A., La Rosa, G., Cambria, A., Lauria, P., Billeri, L., Latella, D., Manuli, A. & Calabrò, R.S. (2020) 'Virtual Reality Based Cognitive Rehabilitation in Minimally Conscious State: A Case Report with EEG Findings and Systematic Literature Review'. *Brain Sciences* **10** (7), 414

Maglaveras, N., Chouvarda, I., Koutkias, V.G., Gogou, G., Lekka, I., Goulis, D., Avramidis, A., Karvounis, C., Louridas, G. & Balas, E.A. (2005) 'The Citizen Health System (CHS): a modular medical contact center providing quality telemedicine services'. *IEEE Transactions on Information Technology in Biomedicine* **9** (3), 353-362

Maglaveras, N. & Reiter, H. (2011) 'Towards closed-loop personal health systems in cardiology: the HeartCycle approach.'. *Annual International Conference Of The IEEE Engineering In Medicine And Biology Society* **2011**, 892-895

Magnocavallo, M., Bernardini, A., Mariani, M.V., Piro, A., Marini, M., Nicosia, A., Adduci, C., Rapacciuolo, A., Saporito, D., Grossi, S., Santarpia, G., Vaccaro, P., Rordorf, R., Pentimalli, F., Giunta, G., Campari, M., Valsecchi, S. & Lavalle, C. (2021) 'Home delivery of the communicator for remote monitoring of cardiac implantable devices: A multicenter experience during the covid-19 lockdown.'. *Pacing & Clinical Electrophysiology*

Maheu, M.M., Pulier, M.L., McMenamin, J.P. & Posen, L. (2012) 'Future of telepsychology, telehealth, and various technologies in psychological research and practice.'. *Professional Psychology: Research and Practice* **43** (6), 613

Mahmud, K. & Lenz, J. (1995) 'The personal telemedicine system. A new tool for the delivery of health care.'. *Journal of Telemedicine & Telecare* **1** (3), 173-177

Maier, S.K.G., Paule, S., Jung, W., Koller, M., Ventura, R., Quesada, A., Bordachar, P., Garcia-Fernandez, F.J., Schumacher, B., Lobitz, N., Takizawa, K., Ando, K., Adachi, K. & Shoda, M. (2019) 'Evaluation of thoracic impedance trends for implant-based remote monitoring in heart failure patients - Results from the (J-)HomeCARE-II Study.'. *Journal of electrocardiology* **53**, 100-108

Maier, S.K.G., Paule, S., Jung, W., Koller, M., Ventura, R., Quesada, A., Bordachar, P., García-Fernández, F.J., Schumacher, B., Lobitz, N., Takizawa, K., Ando, K., Adachi, K. & Shoda, M. (2019) 'Evaluation of thoracic impedance trends for implant-based remote monitoring in heart failure patients - Results from the (J-)HomeCARE-II Study'. *Journal of electrocardiology* **53**, 100-108

Maiolo, C., Mohamed, E.I., Fiorani, C.M. & De Lorenzo, A. (2003) 'Home telemonitoring for patients with severe respiratory illness: the Italian experience.'. *Journal of Telemedicine & Telecare* **9** (2), 67-71

Maitland, J. & Chalmers, M. (2010) 'Self-monitoring, self-awareness, and self-determination in cardiac rehabilitation'. In Anonymous (eds.) *Proceedings of the SIGCHI conference on human factors in computing systems,* Held , 1213-1222

Majorin, F., Torondel, B., Ka Seen Chan, G. & Clasen, T. (2019) 'Interventions to improve disposal of child faeces for preventing diarrhoea and soil-transmitted helminth infection'. *Cochrane Database of Systematic Reviews* (9)

Majumder, S. & Deen, M.J. (2019) 'Smartphone sensors for health monitoring and diagnosis'. *Sensors* **19** (9), 2164

Makhlouf, A., Boudouane, I., Saadia, N. & Cherif, A.R. (2019}) 'Ambient assistance service for fall and heart problem detection'. *JOURNAL OF AMBIENT INTELLIGENCE AND HUMANIZED COMPUTING}* **10}** (4, SI}), 1527-1546}

Malasinghe, L.P., Ramzan, N. & Dahal, K. (2019) 'Remote patient monitoring: a comprehensive study'. *Journal of Ambient Intelligence and Humanized Computing* **10** (1), 57-76

Malloy, L.E., Gingerich, J., Olson, M.D. & Atkins, D.L. (2014}) 'Remote Monitoring of Cardiovascular Implantable Devices in the Pediatric Population Improves Detection of Adverse Events'. *PEDIATRIC CARDIOLOGY}* **35}** (2}), 301-306}

Manta C., Jain S.S., Coravos A., Mendelsohn D. & Izmailova, E.S. (2020) 'An Evaluation of Biometric Monitoring Technologies for Vital Signs in the Era of COVID-19.'. *Clinical and Translational Science* **13** (6), 1034-1044

Manyazewal, T., Woldeamanuel, Y., Blumberg, H.M., Fekadu, A. & Marconi, V.C. (2021) 'The potential of digital health technologies in African context, Ethiopia'. *medRxiv*

Mao, Y., Lin, W., Wen, J. & Chen, G. (2020) 'Impact and efficacy of mobile health intervention in the management of diabetes and hypertension: a systematic review and meta-analysis'. *BMJ open diabetes research & care* **8** (1), 10.1136/bmjdrc-2020-001225

Marahatta, K., Samuel, R., Sharma, P., Dixit, L. & Shrestha, B.R. (2017) 'Suicide burden and prevention in Nepal: The need for a national strategy'. *WHO South-East Asia journal of public health* **6** (1), 45-49

Marani, R., Gelao, G. & Perri, A.G. (2010) 'High quality heart and lung auscultation system for diagnostic use on remote patients in real time.'. *The Open Biomedical Engineering Journal* **4**, 250-256

Marcelli, E., Capucci, A., Minardi, G. & Cercenelli, L. (2017) 'Multi-Sense CardioPatch: A Wearable Patch for Remote Monitoring of Electro-Mechanical Cardiac Activity.'. *ASAIO Journal* **63** (1), 73-79

Marcolino, M.S., Alkmim, M.B.M., e Silva, Maira Viana Rego Souza, Figueira, R.M., de Resende, R.E., Silva, L.B. & Ribeiro, A.L. (2021) 'Telecardiology'. In *Telemedicine, Telehealth and Telepresence* Springer, 379-400

Margelis, G. (2019) 'We are living in a new era for the delivery of Aged and Community Services.'.

Margolis, K.L., Sperl-Hillen, J., Crain, L.A., Ziegenfuss, J.Y., Solberg, L.I., Bergdall, A.R., Beran, M.S., Anderson, J.P., Pawloski, P.A. & Rehrauer, D.J. (2020) 'Improvement in self-monitoring frequency, electronic data sharing, and medication changes in a pragmatic cluster-randomized trial of home blood pressure telemonitoring and pharmacist care (hyperlink 3)'. *Hypertension* **76**

Markert, M., Trautmann, T., Krause, F., Cioaga, M., Mouriot, S., Wetzel, M. & Guth, B.D. (2018) 'A new telemetry-based system for assessing cardiovascular function in group-housed large animals. Taking the 3Rs to a new level with the evaluation of remote measurement via cloud data transmission.'. *Journal of Pharmacological & Toxicological Methods* **93**, 90-97

Marques, G., Roque Ferreira, C. & Pitarma, R. (2018) 'A System Based on the Internet of Things for Real-Time Particle Monitoring in Buildings.'. *International Journal of Environmental Research & Public Health [Electronic Resource]* **15** (4), 04 21

Mars, M. (2017) 'Fourth European Congress on eCardiology and eHealth-8-10 November, 2017-Berlin, Germany'. *Journal of the International Society for Telemedicine and eHealth* **5**, eS2 (1-57)

Mars, M. (2017) 'Med-e-Tel 2017'. *Journal of the International Society for Telemedicine and eHealth* **5**, eS1 (1-92)

Marthick, M., Dhillon, H.M., Alison, J.A., Cheema, B.S. & Shaw, T. (2018) 'An interactive web portal for tracking oncology patient physical activity and symptoms: prospective cohort study'. *JMIR cancer* **4** (2), e11978

Martin, A., Caon, M., Adorni, F., Andreoni, G., Ascolese, A., Atkinson, S., Bul, K., Carrion, C., Castell, C. & Ciociola, V. (2020) 'A Mobile Phone Intervention to Improve Obesity-Related Health Behaviors of Adolescents Across Europe: Iterative Co-Design and Feasibility Study'. *JMIR mHealth and uHealth* **8** (3), e14118

Martinez, E.A. (2017) 'APN Perceptions of Telemedicine and Homecare for Heart Failure Patients'. *APN Perceptions of Telemedicine & Homecare for Heart Failure Patients* , 1-1

Martins C., Machado da Silva J., Guimaraes D., Martins L. & Vaz Da Silva, M. (2021) 'MONITORIA: The start of a new era of ambulatory heart failure monitoring? Part II - Design.'. *Revista Portuguesa de Cardiologia* **40** (5), 343-351

Martyushev-Poklad, A. & Yankevich, D. 'Patent Landscape of Automated Systems for Personalized Health Management: Features, Shortcomings, and Implications for Developing an Optimal ASHM'.

Marwati, T. & Solikhah, S. (2018) 'Health worker compliance associated with decreasing surgical site infection’s both the case and cost'.

Marzegalli, M., Lunati, M., Landolina, M., Perego, G.B., Ricci, R.P., Guenzati, G., Schirru, M., Belvito, C., Brambilla, R., Masella, C., Di Stasi, F., Valsecchi, S. & Santini, M. (2008) 'Remote monitoring of CRT-ICD: the multicenter Italian CareLink evaluation--ease of use, acceptance, and organizational implications.'. *Pacing & Clinical Electrophysiology* **31** (10), 1259-1264

Marziniak, M., Brichetto, G., Feys, P., Meyding-Lamadé, U., Vernon, K. & Meuth, S.G. (2018) 'The use of digital and remote communication technologies as a tool for multiple sclerosis management: narrative review'. *JMIR rehabilitation and assistive technologies* **5** (1), e5

Masek, M., Lee, C.S., Lam, C.P., Tan, K.T. & Fyneman, A. (2009}) 'Remote Home-Based Ante and Post Natal Care'. In Anonymous (eds.) *2009 11TH INTERNATIONAL CONFERENCE ON E-HEALTH NETWORKING, APPLICATIONS AND SERVICES (HEALTHCOM 2009)},* Held 345 E 47TH ST, NEW YORK, NY 10017 USA}: IEEE}, 60}

Matovic, M., Jankovic, S., Jeremic, M., Vlajkovic, M. & Matovic, V. (2012}) 'Online Remote Monitoring of Patients with Differentiated Thyroid Carcinomas and Neuroendocrine Tumors Treated with High Doses of Radionuclides'. *TELEMEDICINE AND E-HEALTH}* **18}** (4}), 264-270}

Matricardi, P.M., Dramburg, S., Alvarez-Perea, A., Antolín-Amérigo, D., Apfelbacher, C., Atanaskovic-Markovic, M., Berger, U., Blaiss, M.S., Blank, S. & Boni, E. (2020) 'The role of mobile health technologies in allergy care: An EAACI position paper'. *Allergy* **75** (2), 259-272

Matsuzaki, M., Sherr, K., Augusto, O., Kawakatsu, Y., Asbjornsdottir, K., Chale, F., Covele, A., Manaca, N., Muanido, A., Wagenaar, B.H., Mocumbi, A.O., Gimbel, S. & InCoMaS Study Team (2020) 'The prevalence of hypertension and its distribution by sociodemographic factors in Central Mozambique: a cross sectional study.'. *BMC Public Health* **20** (1), 1843

Matteucci, I. (2017) 'Information about ourselves from ourselves: young users of wearable technologies in secondary school'. *European Journal of Interdisciplinary Studies* **3** (4), 42-48

Matteucci, A., Bonanni, M., Centioni, M., Zanin, F., Geuna, F., Massaro, G. & Sangiorgi, G. (2021) 'Home Management of Heart Failure and Arrhythmias in Patients with Cardiac Devices during Pandemic.'. *Journal of Clinical Medicine* **10** (8)

Mattila, J., Ding, H., Mattila, E. & Särelä, A. (2009) 'Mobile tools for home-based cardiac rehabilitation based on heart rate and movement activity analysis'. *Annual International Conference of the IEEE Engineering in Medicine and Biology Society.IEEE Engineering in Medicine and Biology Society.Annual International Conference* **2009**, 6448-6452

Mayo-Wilson, E., Junior, J.A., Imdad, A., Dean, S., Chan, X., Chan, E.S., Jaswal, A. & Bhutta, Z.A. (2014) 'Zinc supplementation for preventing mortality, morbidity, and growth failure in children aged 6 months to 12 years of age'. *Cochrane Database of Systematic Reviews* (5)

Mayo-Wilson, E. & Montgomery, P. (2013) 'Media-delivered cognitive behavioural therapy and behavioural therapy (self-help) for anxiety disorders in adults'. *Cochrane Database of Systematic Reviews* (9)

McAlister, F., Lewinter, C., Ball, J., Clark, R., Stewart, S., Cleland, J., Inglis, S. & Cullington, D. (2010) 'Structured telephone support or telemonitoring programmes for patients with chronic heart failure'.

McCabe, C., McCann, M. & Brady, A.M. (2017) 'Computer and mobile technology interventions for self-management in chronic obstructive pulmonary disease'. *Cochrane Database of Systematic Reviews* (5)

McCauley, M.E., van den Broek, N., Dou, L. & Othman, M. (2015) 'Vitamin A supplementation during pregnancy for maternal and newborn outcomes'. *Cochrane Database of Systematic Reviews* (10)

McColl-Kennedy, J.R., Snyder, H., Elg, M., Witell, L., Helkkula, A., Hogan, S.J. & Anderson, L. (2017) 'The changing role of the health care customer: review, synthesis and research agenda'. *Journal of Service Management*

McDermott, M.M. (2017}) 'Exercise training for intermittent claudication'. *JOURNAL OF VASCULAR SURGERY}* **66}** (5}), 1612-1620}

McDonagh, R.J.M. & Sheppard, J.P. 'Effective detection and management of hypertension through community pharmacy in England'.

McElroy, I., Sareh, S., Zhu, A., Miranda, G., Wu, H., Nguyen, M., Shemin, R. & Benharash, P. (2016}) 'Use of digital health kits to reduce readmission after cardiac surgery'. *JOURNAL OF SURGICAL RESEARCH}* **204}** (1}), 1-7}

McGillion, M.H., Duceppe, E., Allan, K., Marcucci, M., Yang, S., Johnson, A.P., Ross-Howe, S., Peter, E., Scott, T. & Ouellette, C. (2018) 'Postoperative remote automated monitoring: need for and state of the science'. *Canadian journal of Cardiology* **34** (7), 850-862

McGillion, M., Ouellette, C., Good, A., Bird, M., Henry, S., Clyne, W., Turner, A., Ritvo, P., Ritvo, S. & Dvirnik, N. (2020) 'Postoperative Remote Automated Monitoring and Virtual Hospital-to-Home Care System Following Cardiac and Major Vascular Surgery: user Testing Study'. *Journal of medical Internet research* **22** (3), e15548-

McGillion, M., Yost, J., Turner, A., Bender, D., Scott, T., Carroll, S., Ritvo, P., Peter, E., Lamy, A., Furze, G., Krull, K., Dunlop, V., Good, A., Dvirnik, N., Bedini, D., Naus, F., Pettit, S., Henry, S., Probst, C., Mills, J., Gossage, E., Travale, I., Duquette, J., Taberner, C., Bhavnani, S., Khan, J.S., Cowan, D., Romeril, E., Lee, J., Colella, T., Choiniere, M., Busse, J., Katz, J., Victor, J.C., Hoch, J., Isaranuwatchai, W., Kaasalainen, S., Ladak, S., O'Keefe-McCarthy, S., Parry, M., Sessler, D.I., Stacey, M., Stevens, B., Stremler, R., Thabane, L., Watt-Watson, J., Whitlock, R., MacDermid, J.C., Leegaard, M., McKelvie, R., Hillmer, M., Cooper, L., Arthur, G., Sider, K., Oliver, S., Boyajian, K., Farrow, M., Lawton, C., Gamble, D., Walsh, J., Field, M., LeFort, S., Clyne, W., Ricupero, M., Poole, L., Russell-Wood, K., Weber, M., McNeil, J., Alpert, R., Sharpe, S., Bhella, S., Mohajer, D., Ponnambalam, S., Lakhani, N., Khan, R., Liu, P. & Devereaux, P.J. (2016) 'Technology-Enabled Remote Monitoring and Self-Management - Vision for Patient Empowerment Following Cardiac and Vascular Surgery: User Testing and Randomized Controlled Trial Protocol.'. *JMIR Research Protocols* **5** (3), e149

McKinnon, K.J. (2017) *Telemedicine: An Augmentation Strategy to Mitigate Primary Care Shortage*

McLaren, L., Sumar, N., Barberio, A.M., Trieu, K., Lorenzetti, D.L., Tarasuk, V., Webster, J. & Campbell, N. (2016) 'Population-level interventions in government jurisdictions for dietary sodium reduction'. *Cochrane Database of Systematic Reviews* (9)

McLean, S., Chandler, D., Nurmatov, U., Liu, J., Pagliari, C., Car, J. & Sheikh, A. (2010) 'Telehealthcare for asthma'. *Cochrane Database of Systematic Reviews* (10)

McLean, S., Nurmatov, U., Liu, J., Pagliari, C., Car, J. & Sheikh, A. (2011) 'Telehealthcare for chronic obstructive pulmonary disease'. *Cochrane Database of Systematic Reviews* (7)

McManus, R.J., Bray, E.P., Mant, J., Holder, R., Greenfield, S., Bryan, S., Jones, M.I., Little, P., Williams, B., Hobbs, F.D., McManus, R.J., Bray, E.P., Mant, J., Holder, R., Greenfield, S., Bryan, S., Jones, M.I., Little, P., Williams, B. & Hobbs, F.D.R. (2009) 'Protocol for a randomised

controlled trial of telemonitoring and self-management in the control of hypertension: telemonitoring and self-management in hypertension. [ISRCTN17585681]'. *BMC Cardiovascular Disorders* **9**, 6-6

McNeil, J., Fahim, M., Stone, C.R., O'Reilly, R., Courneya, K.S. & Friedenreich, C.M. (2021) 'Adherence to a lower versus higher intensity physical activity intervention in the Breast Cancer & Physical Activity Level (BC-PAL) Trial'. *Journal of cancer survivorship : research and practice*

McNicholas, L.F. (2010) 'Poster Abstracts from the AAAP 20th Annual Meeting and Symposium'. *American Journal on Addictions* **19** (4), 368-381

McPheeters, M.L., Kripalani, S., Peterson, N.B., Idowu, R.T., Jerome, R.N., Potter, S.A. & Andrews, J.C. (2012) 'Closing the quality gap: revisiting the state of the science (vol. 3: quality improvement interventions to address health disparities)'. *Evidence Report/Technology Assessment.(208*

McVeigh, J.A., Ellis, J., Ross, C., Tang, K., Wan, P., Halse, R.E., Dhaliwal, S.S., Kerr, D.A. & Straker, L. (2021) 'Convergent Validity of the Fitbit Charge 2 to Measure Sedentary Behavior and Physical Activity in Overweight and Obese Adults'. *Journal for the Measurement of Physical Behaviour* **4** (1), 39-46

Mead, E., Brown, T., Rees, K., Azevedo, L.B., Whittaker, V., Jones, D., Olajide, J., Mainardi, G.M., Corpeleijn, E. & O'Malley, C. (2017) 'Diet, physical activity and behavioural interventions for the treatment of overweight or obese children from the age of 6 to 11 years'. *Cochrane Database of Systematic Reviews* (6)

Mehta, P., Dhar, R. & Messias, E. (2020) 'Successful aging'. In *Positive psychiatry, psychotherapy and psychology* Springer, 73-90

Melchiorre, M.G., Lamura, G., Barbabella, F. & ICARE4EU Consortium (2018) 'eHealth for people with multimorbidity: Results from the ICARE4EU project and insights from the "10 e's" by Gunther Eysenbach'. *PloS one* **13** (11), e0207292

Melchiorre, M.G., Papa, R., Rijken, M., van Ginneken, E., Hujala, A. & Barbabella, F. (2018) 'eHealth in integrated care programs for people with multimorbidity in Europe: Insights from the ICARE4EU project'. *Health policy* **122** (1), 53-63

Messiah, S.E., Sacher, P.M., Yudkin, J., Ofori, A., Qureshi, F.G., Schneider, B., Hoelscher, D.M., De la Cruz-Munoz, N. & Barlow, S.E. (2020) 'Application and effectiveness of eHealth strategies for metabolic and bariatric surgery patients: a systematic review'. *Digital health* **6**, 2055207619898987

Meyer, C., Waite, M., Atkins, J., Scarinci, N., Cowan, R. & Hickson, L. (2019) 'Promoting patient-centered hearing care through the use of eHealth: Current status and future possibilities'. *Perspectives of the ASHA Special Interest Groups* **4** (2), 331-344

Meystre, S. (2005) 'The current state of telemonitoring: a comment on the literature'. *Telemedicine Journal & e-Health* **11** (1), 63-69

Mhajna, M., Schwartz, N., Levit-Rosen, L., Warsof, S., Lipschuetz, M., Jakobs, M., Rychik, J., Sohn, C. & Yagel, S. (2020) 'Wireless, remote solution for home fetal and maternal heart rate monitoring.'. *American Journal of Obstetrics & Gynecology MFM* **2** (2), 100101

Mhimbira, F.A., Cuevas, L.E., Dacombe, R., Mkopi, A. & Sinclair, D. (2017) 'Interventions to increase tuberculosis case detection at primary healthcare or community-level services'. *Cochrane Database of Systematic Reviews* (11)

Miah, S.J., Gammack, J. & Hasan, N. (2017) 'Extending the framework for mobile health information systems Research: A content analysis'. *Information Systems* **69**, 1-24

Michaud, T.L., Siahpush, M., Estabrooks, P., Schwab, R.J., LeVan, T.D., Grimm, B., Ramos, A.K., Johansson, P., Scoggins, D. & Su, D. (2020}) 'Association Between Weight Loss and Glycemic Outcomes: A Post Hoc Analysis of a Remote Patient Monitoring Program for Diabetes Management'. *TELEMEDICINE AND E-HEALTH}* **26}** (5}), 621-628}

Michaud, T.L., Siahpush, M., King, K.M., Ramos, A.K., Robbins, R.E., Schwab, R.J., Clarke, M.A. & Su, D. (2020}) 'Program completion and glycemic control in a remote patient monitoring program for diabetes management: Does gender matter?'. *DIABETES RESEARCH AND CLINICAL PRACTICE}* **159}** (SI})

Michaud, T.L., Siahpush, M., Schwab, R.J., Eiland, L.A., DeVany, M., Hansen, G., Slachetka, T.S., Boilesen, E., Tak, H.J., Wilson, F.A., Wang, H., Pagan, J.A. & Su, D. (2018}) 'Remote Patient Monitoring and Clinical Outcomes for Postdischarge Patients with Type 2 Diabetes'. *POPULATION HEALTH MANAGEMENT}* **21}** (5}), 387-394}

Mika, M., Kenneth, S., Orvalho, A., Yoshito, K., Kristjana, Á., Falume, C., Alfredo, C., Nelia, M., Alberto, M., Wagenaar, B.H., Mocumbi, A.O., Sarah, G., the InCoMaS, S.T., Manuel, J.L., Hicks, L., Mahumane, A., Pfeiffer, J., Gloyd, S., Cuembelo, F. & Nhumba, M. (2020) 'The prevalence of hypertension and its distribution by sociodemographic factors in Central Mozambique: a cross sectional study'. *BMC Public Health* **20** (1), N.PAG-N.PAG

Miller, H.J. & Tolle, K. (2016) 'Big data for healthy cities: Using location-aware technologies, open data and 3D urban models to design healthier built environments'. *Built Environment* **42** (3), 441-456

Miller, J.C., Skoll, D. & Saxon, L.A. (2020) 'Home Monitoring of Cardiac Devices in the Era of COVID-19'. *Current cardiology reports* **23** (1), 1

Miralles, I., Granell, C., Díaz-Sanahuja, L., Van Woensel, W., Bretón-López, J., Mira, A., Castilla, D. & Casteleyn, S. (2020) 'Smartphone apps for the treatment of mental disorders: systematic review'. *JMIR mHealth and uHealth* **8** (4), e14897

Mocanu, I., Schpor, O., Cramariuc, B. & Rusu, L. (2017) 'Mobile@ Old: A Smart Home Platform for Enhancing the Elderly Mobility'. *Advances in Electrical and Computer Engineering* **17** (4), 19-26

Model, Cerebral Emergencies Physician-Focused Payment 'ACCESS Telemedicine: An Alternative Healthcare Delivery Model for Rural Cerebral Emergencies Physician-Focused Payment Model Environmental Scan'.

Mohamad, M. & Cresswell, M. (2019) 'Technology as tool to overcome barriers'.

Mohamad, M. & Cresswell, M. (2019) 'How technology enhances fitness facility practices: a health behaviour perspective'. *Proceedings of the British Academy of Management* , 1-31

Molina-Recio, G., Molina-Luque, R., Jiménez-García, A.M., Ventura-Puertos, P.E., Hernández-Reyes, A. & Romero-Saldaña, M. (2020) 'Proposal for the User-Centered Design Approach for Health Apps Based on Successful Experiences: Integrative Review'. *JMIR mHealth and uHealth* **8** (4), e14376

Molina-Recio, G., Molina-Luque, R. & Romero-Saldaña, M. (2021) 'The importance of knowing and listening to all those involved in the design and use of nutrition mobile apps. Getting to know the Great GApp.'. *Nutricion Hospitalaria* **38** (3)

Molinari, F., Meiburger, K.M. & Suri, J. (2011) 'Automated high-performance cIMT measurement techniques using patented AtheroEdge TM: a screening and home monitoring system.'. *Annual International Conference Of The IEEE Engineering In Medicine And Biology Society* **2011**, 6651-6654

Molyneaux, E., Howard, L.M., McGeown, H.R., Karia, A.M. & Trevillion, K. (2014) 'Antidepressant treatment for postnatal depression'. *Cochrane Database of Systematic Reviews* (9)

Mönninghoff, A., Kramer, J.N., Hess, A.J., Ismailova, K., Teepe, G.W., Car, L.T., Müller-Riemenschneider, F. & Kowatsch, T. (2021) 'Long-term Effectiveness of mHealth Physical Activity Interventions: Systematic Review and Meta-analysis of Randomized Controlled Trials'. *Journal of medical Internet research* **23** (4), e26699

Moon J.K., Smith B., Tan Y. & Barden, C. (2010) 'Developing the smartphone sensor suite for obesity research.'. *Obesity.Conference: 28th Annual Scientific Meeting of the Obesity Society, OBESITY 2010.San Diego, CA United States.Conference Publication: (var.pagings)* **18** (SUPPL. 2), S203

Moore, G.C. & Benbasat, I. (1991) 'Development of an instrument to measure the perceptions of adopting an information technology innovation'. *Information systems research* **2** (3), 192-222

Moore, G., Wilding, H., Gray, K. & Castle, D. (2019) 'Participatory methods to engage health service users in the development of electronic health resources: systematic review'. *Journal of participatory medicine* **11** (1), e11474

Moore, E.R., Bergman, N., Anderson, G.C. & Medley, N. (2016) 'Early skin-to-skin contact for mothers and their healthy newborn infants'. *Cochrane Database of Systematic Reviews* (11)

Moreno-Gutierrez, S., Postigo-Martin, P., Damas, M., Pomares, H., Banos, O., Arroyo-Morales, M. & Cantarero-Villanueva, I. (2021}) 'ATOPE plus : An mHealth System to Support Personalized Therapeutic Exercise Interventions in Patients With Cancer'. *IEEE ACCESS}* **9}**, 16878-16898}

Morgan, E.H., Schoonees, A., Sriram, U., Faure, M. & Seguin-Fowler, R. (2020) 'Caregiver involvement in interventions for improving children's dietary intake and physical activity behaviors'. *Cochrane Database of Systematic Reviews* (1)

Morgan, E.H., Graham, M.L., Folta, S.C. & Seguin, R.A. (2016) 'A qualitative study of factors related to cardiometabolic risk in rural men'. *BMC Public Health* **16** (1), 1-10

Morosetti M., Peccerillo M. & Fama, M.I. (2020) 'Clinical and social advantages of remote patient monitoring in home dialysis.'. *Giornale italiano di nefrologia : organo ufficiale della Societa italiana di nefrologia* **37** (2) (pagination), ate of Pubaton: 09 Ar 2020

Morris-Paxton, A.A., Rheeder, P., Ewing, R.G. & Woods, D. (2018) 'Detection, referral and control of diabetes and hypertension in the rural Eastern Cape Province of South Africa by community health outreach workers in the rural primary healthcare project: Health in Every Hut.'. *African Journal of Primary Health Care & Family Medicine* **10** (1), e1-e8

Morriss, R., Kaylor-Hughes, C., Rawsthorne, M., Coulson, N., Simpson, S., Guo, B., James, M., Lathe, J., Moran, P. & Williams, L. (2021) 'A Direct-to-Public Peer Support Program (Big White Wall) Versus Web-Based Information to Aid the Self-management of Depression and Anxiety: Results

and Challenges of an Automated Randomized Controlled Trial'. *Journal of medical Internet research* **23** (4), e23487

Morse, R.S., Lambden, K., Quinn, E., Ngoma, T., Mushi, B., Ho, Y.X., Ngoma, M., Mahuna, H., Sagan, S.B. & Mmari, J. (2021) 'A Mobile App to Improve Symptom Control and Information Exchange Among Specialists and Local Health Workers Treating Tanzanian Cancer Patients: Human-Centered Design Approach'. *JMIR cancer* **7** (1), e24062

Mortara, A., Oliva, F. & Di Lenarda, A. (2010) '[Current perspectives in telemonitoring and devices in chronic heart failure patients: lights and shadows]'. *Giornale italiano di cardiologia* **11** (5 Suppl 2), 33S-37S

Mosa, A.S.M., Yoo, I. & Sheets, L. (2012) 'A systematic review of healthcare applications for smartphones'. *BMC medical informatics and decision making* **12** (1), 1-31

Mosdøl, A., Lidal, I.B., Straumann, G.H. & Vist, G.E. (2017) 'Targeted mass media interventions promoting healthy behaviours to reduce risk of non-communicable diseases in adult, ethnic minorities'. *Cochrane Database of Systematic Reviews* (2)

Mosnaim, G., Safioti, G., Brown, R., DePietro, M., Szefler, S.J., Lang, D.M., Portnoy, J.M., Bukstein, D.A., Bacharier, L.B. & Merchant, R.K. (2021) 'Digital Health Technology in Asthma: A Comprehensive Scoping Review'. *The Journal of Allergy and Clinical Immunology: In Practice*

Motta, L.P., Silva, P.P.F.d., Borguezan, B.M., Amaral, J.L.M.d., Milagres, L.G., Boia, M.N., Ferraz, M.R., Mogami, R., Nunes, R.A. & Melo, P.L.d. (2021) 'An emergency system for monitoring pulse oximetry, peak expiratory flow, and body temperature of patients with COVID-19 at home: Development and preliminary application.'. *PLoS ONE [Electronic Resource]* **16** (3), e0247635

Mueller, M., Knop, M., Niehaves, B. & Adarkwah, C.C. (2020) 'Investigating the Acceptance of Video Consultation by Patients in Rural Primary Care: Empirical Comparison of Preusers and Actual Users'. *JMIR medical informatics* **8** (10), e20813

Mukai, K., Yonezawa, Y., Ogawa, H., Maki, H. & Caldwell, W.M. (2009) 'A remote monitor of bed patient cardiac vibration, respiration and movement.'. *Annual International Conference Of The IEEE Engineering In Medicine And Biology Society* **2009**, 5191-5194

Mukkamala, R., Hahn, J., Inan, O.T., Mestha, L.K., Kim, C., Töreyin, H. & Kyal, S. (2015) 'Toward ubiquitous blood pressure monitoring via pulse transit time: theory and practice'. *IEEE Transactions on Biomedical Engineering* **62** (8), 1879-1901

Müller, A.M., Alley, S., Schoeppe, S. & Vandelanotte, C. (2016) 'The effectiveness of e-& mHealth interventions to promote physical activity and healthy diets in developing countries: a systematic review'. *International Journal of Behavioral Nutrition and Physical Activity* **13** (1), 1-14

Muller, A.E., Ormstad, S.S., Jardim, P., Johansen, T.B. & Berg, R. (2020) 'Managing chronic illnesses with remote patient monitoring in primary health care'.

Muller, A., Goette, A., Perings, C., Nagele, H., Konorza, T., Spitzer, W., Schulz, S., von Bary, C., Hoffmann, M., Albani, M., Sack, S., Niederlohner, A. & Lewalter, T. (2013) 'Potential role of telemedical service centers in managing remote monitoring data transmitted daily by cardiac implantable electronic devices: results of the early detection of cardiovascular events in device patients with heart failure (detecT-Pilot) study.'. *Telemedicine Journal & E-Health* **19** (6), 460-466

Mulvaney, C.A., Smith, S., Watson, M.C., Parkin, J., Coupland, C., Miller, P., Kendrick, D. & McClintock, H. (2015) 'Cycling infrastructure for reducing cycling injuries in cyclists'. *Cochrane Database of Systematic Reviews* (12)

Münzel, T., Sørensen, M., Lelieveld, J., Hahad, O., Al-Kindi, S., Nieuwenhuijsen, M., Giles-Corti, B., Daiber, A. & Rajagopalan, S. (2021) 'Heart healthy cities: genetics loads the gun but the environment pulls the trigger'. *European heart journal*

Murray, M.F., Evans, J.P., Angrist, M., Chan, K., Uhlmann, W., Doyle, D.L., Fullerton, S.M., Ganiats, T.G., Hagenkord, J. & Imhof, S. (2018) 'A proposed approach for implementing genomics-based screening programs for healthy adults'. *NAM Perspectives* **10**

Murray, E., Burns, J., See Tai, S., Lai, R. & Nazareth, I. (2005) 'Interactive Health Communication Applications for people with chronic disease'. *Cochrane Database of Systematic Reviews* (4)

Mutz, M., Müller, J. & Reimers, A.K. (2021) 'Use of Digital Media for Home-Based Sports Activities during the COVID-19 Pandemic: Results from the German SPOVID Survey'. *International Journal of Environmental Research and Public Health* **18** (9), 4409

Mykkänen, M. (2021) 'Wearable motion sensors in measuring the activity of a rehabilitation patient'.

Nabutovsky, I., Ashri, S., Nachshon, A., Tesler, R., Shapiro, Y., Wright, E., Vadasz, B., Offer, A., Grosman-Rimon, L. & Klempfner, R. (2020) 'Feasibility, Safety, and Effectiveness of a Mobile Application in Cardiac Rehabilitation'. *The Israel Medical Association journal : IMAJ* **22** (6), 357-363

Naef, R.W.3., Perry, K.G.J., Magann, E.F., McLaughlin, B.N., Chauhan, S.P. & Morrison, J.C. (1998) 'Home blood pressure monitoring for pregnant patients with hypertension.'. *Journal of Perinatology* **18** (3), 226-229

Nagele, H., Lipoldova, J., Oswald, H., Klein, G., Elvan, A., Vester, E., Bauer, W., Bondke, H., Reif, S., Daub, C., Menzel, F., Schrader, J. & Zach, G. (2015) 'Home monitoring of implantable cardioverter-defibrillators: interpretation reliability of the second-generation "IEGM Online" system.'. *Europace* **17** (4), 584-590

Nagy, A., Lipoldova, J., Novak, M. & Stepanova, R. (2017}) 'Occurrence of implantable cardioverter-defibrillator therapy in clinical practice'. *COR ET VASA}* **59}** (3}), E215-E221}

Najafi, B., Veranyan, N., Zulbaran-Rojas, A., Park, C., Nguyen, H., Nakahara, Q.K., Elizondo-Adamchik, H., Chung, J., Mills, J.L. & Montero-Baker, M. (2020) 'Association between wearable device–based measures of physical frailty and major adverse events following lower extremity revascularization'. *JAMA Network Open* **3** (11), e2020161-e2020161

Nakamura, N., Koga, T. & Iseki, H. (2014}) 'A meta-analysis of remote patient monitoring for chronic heart failure patients'. *JOURNAL OF TELEMEDICINE AND TELECARE}* **20}** (1}), 11-17}

Nasi, G., Cucciniello, M. & Guerrazzi, C. (2015) 'The role of mobile technologies in health care processes: the case of cancer supportive care'. *Journal of medical Internet research* **17** (2), e3757

National Academies of Sciences, Engineering, and Medicine (2019) 'Implementing strategies to enhance public health surveillance of physical activity in the United States'.

Naude, Y. & Rohwer, E.R. (2012) 'Two multidimensional chromatographic methods for enantiomeric analysis of o,p'-DDT and o,p'-DDD in contaminated soil and air in a malaria area of South Africa.'. *Analytica Chimica Acta* **730**, 120-126

Nazir, S., Ali, Y., Ullah, N. & García-Magariño, I. (2019) 'Internet of things for healthcare using effects of mobile computing: a systematic literature review'. *Wireless Communications and Mobile Computing* **2019**

NCT00386048, (2006) 'Pain Management Protocol for Pediatric Sickle Cell Disease'. *https://clinicaltrials.gov/show/NCT00386048*

NCT00415545, (2006) 'Educational Program to Improve Heart Failure Outcomes in Adults Living in Rural Areas'. *https://clinicaltrials.gov/show/NCT00415545*

NCT00471705, (2007) 'Efficacy and Safety of Miltefosine or Thermotherapy for Cutaneous Leishmaniasis in Colombia'. *https://clinicaltrials.gov/show/NCT00471705*

NCT00683813, (2008) 'Trial of a Cardiac Rehabilitation Program Delivered Remotely Through the Internet'. *https://clinicaltrials.gov/show/NCT00683813*

NCT00850551, (2009) 'Early Intervention in Pulmonary Exacerbation in Cystic Fibrosis'. *https://clinicaltrials.gov/show/NCT00850551*

NCT00885677, (2009) 'MOnitoring REsynchronization deviCes and cARdiac patiEnts'. *https://clinicaltrials.gov/show/NCT00885677*

NCT00989417, (2009) 'Benefits of Implantable Cardioverter Defibrillator Follow-up Using Remote Monitoring'. *https://clinicaltrials.gov/show/NCT00989417*

NCT01135381, (2010) 'IVR-Enhanced Care Transition Support for Complex Patients'. *https://clinicaltrials.gov/show/NCT01135381*

NCT01250600, (2010) 'Effects of Intelligent Tele-Cardiopulmonary Exercise System on Rehabilitation for Patients With Myocardial Infarction'. *https://clinicaltrials.gov/show/NCT01250600*

NCT01550757, (2012) 'Aligning Resources to Care for Homeless Veterans'. *https://clinicaltrials.gov/show/NCT01550757*

NCT01820338, (2013) 'Extension Family Lifestyle Intervention Project (E-FLIP for Kids)'. *https://clinicaltrials.gov/show/NCT01820338*

NCT01881009, (2013) 'Medtronic Minimed Overnight Closed-Loop System'. *https://clinicaltrials.gov/show/NCT01881009*

NCT01969604, (2013) 'Reduction of Daily Sitting Time in Patients With Rheumatoid Arthritis'. *https://clinicaltrials.gov/show/NCT01969604*

NCT02035566, (2013) 'Telehome Monitoring for Chronic Disease Management'. *https://clinicaltrials.gov/show/NCT02035566*

NCT02048748, (2013) 'Congestive Heart Failure Home Telemonitoring'. *https://clinicaltrials.gov/show/NCT02048748*

NCT02159885, (2014) 'Telemedici ne Management of Veterans With Newly Diagnosed Obstructiv e Sleep Apnea (OSA)'. *https://clinicaltrials.gov/show/NCT02159885*

NCT02188732, (2014) 'Self-Management Training and Automated Telehealth to Improve SMI Health Outcomes'. *https://clinicaltrials.gov/show/NCT02188732*

NCT02221063, (2014) 'Thiamin Fortified Fish Sauce as a Means of Combating Infantile Beriberi in Rural Cambodia'. *https://clinicaltrials.gov/show/NCT02221063*

NCT02279901, (2014) 'Impact of Automated Education and Follow-up Mechanisms on Patient Engagement'. *https://clinicaltrials.gov/show/NCT02279901*

NCT02293252, (2014) 'CardioBBEAT - Randomized Controled Trial to Evaluate the Health Economic Impact of Remote Patient Monitoring'. *https://clinicaltrials.gov/show/NCT02293252*

NCT02409225, (2015) 'Remote Supervision to Decrease Hospitalization Rate'. *https://clinicaltrials.gov/show/NCT02409225*

NCT02446873, (2015) 'Cracking the Egg Potential: improving Young Child Nutrition in Ecuador'. *https://clinicaltrials.gov/show/NCT02446873*

NCT02489370, (2015) 'CHF Home Telemonitoring: a Home Telemonitoring Service for Chronic Heart Failure Patients on Trial'. *https://clinicaltrials.gov/show/NCT02489370*

NCT02551640, (2015) 'Improving Physical Activity Through a mHealth Intervention in Cardio-metabolic Risk Patients'. *https://clinicaltrials.gov/show/NCT02551640*

NCT02568137, (2015) 'Phone-based Intervention Under Nurse Guidance After Stroke'. *https://clinicaltrials.gov/show/NCT02568137*

NCT02708654, (2016) 'Automated Hovering for Congestive Heart Failure Patients'. *https://clinicaltrials.gov/show/NCT02708654*

NCT02711631, (2016) 'Feasibility and Effectiveness of Remote Virtual Reality-Based Cardiac Rehabilitation'. *https://clinicaltrials.gov/show/NCT02711631*

NCT02786940, (2016) 'Remote Cardiac Monitoring of Higher-Risk Emergency Department Syncope Patients After Discharge'. *https://clinicaltrials.gov/show/NCT02786940*

NCT03002311, (2016) 'Improving Medical Care With Electronic Interventions Based on Automated Text and Phone Messages'. *https://clinicaltrials.gov/show/NCT03002311*

NCT03007745, (2017) 'Remote Sleep Apnea Management'. *https://clinicaltrials.gov/show/NCT03007745*

NCT03072693, (2017) 'Daily Ambulatory Remote Monitoring System For Post-Dischage Management Of ADHF'. *https://clinicaltrials.gov/show/NCT03072693*

NCT03096951, (2017) 'Prehabilitation in Colorectal Cancer'. *https://clinicaltrials.gov/show/NCT03096951*

NCT03253549, (2017) 'THE SMArTVIEW, CoVeRed'. *https://clinicaltrials.gov/show/NCT03253549*

NCT03273140, (2017) 'The Effectiveness of Gamification Diabetes Education Program for Poorly Controlled Type 2 Diabetic Patients'. *https://clinicaltrials.gov/show/NCT03273140*

NCT03368417, (2017) 'WIreless Monitoring and Financial Incentives for Uncontrolled HYpertension (WIFHY) Study'. *https://clinicaltrials.gov/show/NCT03368417*

NCT03415841, (2018) 'Kardia - A Smartphone-based Care Model for Outpatient Cardiac Rehabilitation'. *https://clinicaltrials.gov/show/NCT03415841*

NCT03451630, (2018) 'Integrated Care Models to Improve Patient Centered-Outcomes'. *https://clinicaltrials.gov/show/NCT03451630*

NCT03479957, (2018) 'Remotely Monitored and Coached Cardiac Rehabilitation Northern Sweden'. *https://clinicaltrials.gov/show/NCT03479957*

NCT03492203, (2018) 'Remote Cognitive Remediation for Depression'. *https://clinicaltrials.gov/show/NCT03492203*

NCT03496948, (2018) 'Telephone Health Coaching and Remote Exercise Monitoring for Peripheral Artery Disease'. *https://clinicaltrials.gov/show/NCT03496948*

NCT03503812, (2018) 'Mitigating the Health Effects of Desert Dust Storms Using Exposure-Reduction Approaches'. *https://clinicaltrials.gov/show/NCT03503812*

NCT03548181, (2018) 'Feasibility & Effect of a Tele-rehabilitation Program in Idiopathic Pulmonary Fibrosis (IPF)'. *https://clinicaltrials.gov/show/NCT03548181*

NCT03569384, (2018) 'Feasibility & Effect of a Tele-rehabilitation Program for Chronic Obstructive Pulmonary Disease vs. Standard Rehabilitation'. *https://clinicaltrials.gov/show/NCT03569384*

NCT03788915, (2018) 'Weight Loss and Reversing T2D Through eHealth Coaching'. *https://clinicaltrials.gov/show/NCT03788915*

NCT03872856, (2019) 'Blood Pressure-Improving Control Among Alaska Native People" (BP-ICAN)'. *https://clinicaltrials.gov/show/NCT03872856*

NCT03986931, (2019) 'A Pharmacist Intervention for Monitoring and Treating Hypertension Using Bidirectional Texting'. *https://clinicaltrials.gov/show/NCT03986931*

NCT04011800, (2019) 'Catheter Ablation vs. Risk Factor Modification'. *https://clinicaltrials.gov/show/NCT04011800*

NCT04022590, (2019) 'Cardiopulmonary Training in the Victims With Multiple Morbidities by Application of Novel Heart Rate Sensing Clothes'. *https://clinicaltrials.gov/show/NCT04022590*

NCT04046471, (2019) 'Weight Loss Physical Disabilities'. *https://clinicaltrials.gov/show/NCT04046471*

NCT04076280, (2019) 'Sodium Watchers Program - Hypertension'. *https://clinicaltrials.gov/show/NCT04076280*

NCT04083885, (2019) 'Virtual Reality Training to Increase Rehabilitative Exercise in Seniors'. *https://clinicaltrials.gov/show/NCT04083885*

NCT04086992, (2019) 'Decreasing Parental Stress of Caregivers of Infants With Infantile Spasms by Using Telemedicine Technology'. https://clinicaltrials.gov/show/NCT04086992

NCT04098354, (2019) 'Telemonitoring of Hypertensive Patients With Chronic Kidney Disease'. https://clinicaltrials.gov/show/NCT04098354

NCT04113057, (2019) 'Home-based walking program with smart devices'. https://clinicaltrials.gov/show/NCT04113057

NCT04181983, (2019) 'Personalized Exercise Programs for Older Adults - a Digital User-friendly Application'. https://clinicaltrials.gov/show/NCT04181983

NCT04205513, (2019) 'Use of Telemonitoring to Facilitate Heart Failure Medication Titration'. https://clinicaltrials.gov/show/NCT04205513

NCT04273854, (2020) 'Physician Optimised Post-partum Hypertension Treatment Trial'. https://clinicaltrials.gov/show/NCT04273854

NCT04306770, (2020) 'Optimizing Care of Patients Via Telehealth In Monitoring and Augmenting Their Control of Diabetes Mellitus'. https://clinicaltrials.gov/show/NCT04306770

NCT04309461, (2020) 'Make Better Choices 2 for Rural Appalachians'. https://clinicaltrials.gov/show/NCT04309461

NCT04323631, (2020) 'Hydroxychloroquine for the Treatment of Patients With Mild to Moderate COVID-19 to Prevent Progression to Severe Infection or Death'. https://clinicaltrials.gov/show/NCT04323631

NCT04340960, (2020) 'Home Monitoring for Thoracic Surgery Patients'. https://clinicaltrials.gov/show/NCT04340960

NCT04343794, (2020) 'Using Biovitals® Sentinel to Monitor Disease Progression in Subjects Quarantined for Suspected COVID-19'. https://clinicaltrials.gov/show/NCT04343794

NCT04466852, (2020) 'Brazilian Heart Insufficiency With Telemedicine'. https://clinicaltrials.gov/show/NCT04466852

NCT04480931, (2020) 'Pilot Testing a Mobile App to Designed to Increase Physical Activity Among Pregnant and Postpartum Women'. https://clinicaltrials.gov/show/NCT04480931

NCT04545489, (2020) 'A Nurse-led Intervention to Extend the Veteran HIV Treatment Cascade for Cardiovascular Disease Prevention'. https://clinicaltrials.gov/show/NCT04545489

NCT04607876, (2020) 'Family at Risk Study'. https://clinicaltrials.gov/show/NCT04607876

NCT04614428, (2020) 'Improving Outcomes in Patients With Cardiovascular Seasonality'. https://clinicaltrials.gov/show/NCT04614428

NCT04614545, (2020) 'Comparing a Virtual vs. Face to Face Weight Management Program Using Phentermine for Patients With Overweight or Obesity'. https://clinicaltrials.gov/show/NCT04614545

NCT04629066, (2020) 'Efficacy and Safety of ICD Remote Monitored Exercise Testing to Improve Heart Failure Outcomes: REMOTE HF-ACTION'. https://clinicaltrials.gov/show/NCT04629066

NCT04659746, (2020) 'MejoraCare-Paraguay: mHealth Solution for Chronic Patients During the COVID-19 Outbreak in Paraguay'. https://clinicaltrials.gov/show/NCT04659746

Nenov, V. & Klopp, J. (1996) 'Remote analysis of physiological data from neurosurgical ICU patients.'. *Journal of the American Medical Informatics Association* **3** (5), 318-327

Neo S.X.M. & Chung, C.L. (2019) 'Innovative delivery of a home-based gamified rehabilitation for early Parkinson's disease - A protocol for a usability evaluation of a digitalized healthcare approach.'. *Journal of Parkinson's Disease.Conference: 5th World Parkinson Congress, WPC 2019.Kyoto Japan* **9** (1), 132-133

Nerlich, M. & Kretschmer, R. (1999) 'Assessing the impact of telemedicine on health care management'. *The impact of telemedicine on health care management* **64**, 46

Neupane, D., McLachlan, C.S., Christensen, B., Karki, A., Perry, H.B. & Kallestrup, P. (2016) 'Community-based intervention for blood pressure reduction in Nepal (COBIN trial): study protocol for a cluster-randomized controlled trial'. *Trials* **17** (1), 292

Neuzil, P., Taborsky, M., Holy, F. & Wallbrueck, K. (2008) 'Early automatic remote detection of combined lead insulation defect and ICD damage.'. *Europace* **10** (5), 556-557

Ngogang, J.Y. & Tambo, E. (2018) 'Wearable nutrition and dietetics technology on health nutrition paradigm shift in low and middle income countries'. *International Journal of Nutrition and Metabolism* **10** (5), 31-36

Ni, Z., Atluri, N., Shaw, R.J., Tan, J., Khan, K., Merk, H., Ge, Y., Shrestha, S., Shrestha, A., Vasudevan, L., Karmacharya, B. & Yan, L.L. (2020) 'Evaluating the Feasibility and Acceptability of a Mobile Health-Based Female Community Health Volunteer Program for Hypertension Control in Rural Nepal: Cross-Sectional Study.'. *JMIR MHealth and UHealth* **8** (3), e15419

Nicolucci, A., Cercone, S., Chiriatti, A., Muscas, F. & Gensini, G. (2015) 'A Randomized Trial on Home Telemonitoring for the Management of Metabolic and Cardiovascular Risk in Patients with Type 2 Diabetes.'. *Diabetes Technology & Therapeutics* **17** (8), 563-570

Nieuwlaat, R., Wilczynski, N., Navarro, T., Hobson, N., Jeffery, R., Keepanasseril, A., Agoritsas, T., Mistry, N., Iorio, A. & Jack, S. (2014) 'Interventions for enhancing medication adherence'. *Cochrane Database of Systematic Reviews* (11)

Nilsson, L. & Norling, J. (2018) 'Investigation of E-health solutions for chronic diseases and the cost benefits in Swedish Regions/County councils'.

Noel, H.C., Vogel, D.C., Erdos, J.J., Cornwall, D. & Levin, F. (2004) 'Home telehealth reduces healthcare costs'. *Telemedicine Journal & e-Health* **10** (2), 170-183

Nolte, E. & World Health Organization (2018) 'How do we ensure that innovation in health service delivery and organization is implemented, sustained and spread?'.

Noon, G.P. & Loebe, M. (2010) 'Current status of the MicroMed DeBakey Noon Ventricular Assist Device.'. *Texas Heart Institute Journal* **37** (6), 652-653

Norman, G.J., Patrick, K. & Marshall, S.J. 'Wireless Technology for Health Behavior Change Measurement & Intervention'. *Lecture Notes on Wireless Healthcare Research* , 33

Notley, C., Gentry, S., Livingstone-Banks, J., Bauld, L., Perera, R. & Hartmann-Boyce, J. (2019) 'Incentives for smoking cessation'. *Cochrane Database of Systematic Reviews* (7)

Novak Lauscher, H., Ho, K., Cordeiro, J.L., Bhullar, A., Abu Laban, R., Christenson, J., Harps, H., Hawkins, N., Karim, E. & Kim Sing, C. (2018) 'TEC4Home heart failure: using home telemonitoring to decrease ED readmissions and clinical flow'. *Canadian journal of emergency medicine* **20**, S56-S57

Nowell, W.B., Merkel, P.A., McBurney, R.N., Young, K., Venkatachalam, S., Shaw, D.G., Dobes, A., Cerciello, E., Kolaczkowski, L. & Curtis, J.R. (2021) 'Patient-Powered Research Networks of the Autoimmune Research Collaborative: Rationale, Capacity, and Future Directions'. *The Patient-Patient-Centered Outcomes Research* , 1-12

NTR2592, (2010) 'Integrated telemonitoring and telecare for patients with heart failure'. *http://www.who.int/trialsearch/Trial2.aspx?TrialID=NTR2592*

NTR4553, (2014) 'Prevention of dehydration of independently living elderly people at risk by education and technological support'. *http://www.who.int/trialsearch/Trial2.aspx?TrialID=NTR4553*

Nuara, A., Fabbri-Destro, M., Scalona, E., Lenzi, S.E., Rizzolatti, G. & Avanzini, P. (2021) 'Telerehabilitation in response to constrained physical distance: An opportunity to rethink neurorehabilitative routines'. *Journal of neurology* , 1-12

Nundy, S., Dick, J.J., Chou, C., Nocon, R.S., Chin, M.H. & Peek, M.E. (2014) 'Mobile phone diabetes project led to improved glycemic control and net savings for Chicago plan participants'. *Health affairs* **33** (2), 265-272

Nussbaum, R., Kelly, C., Quinby, E., Mac, A., Parmanto, B. & Dicianno, B.E. (2019) 'Systematic review of mobile health applications in rehabilitation'. *Archives of Physical Medicine and Rehabilitation* **100** (1), 115-127

O'brien, T., Troutman-Jordan, M., Hathaway, D., Armstrong, S. & Moore, M. (2015) 'Acceptability of wristband activity trackers among community dwelling older adults'. *Geriatric nursing* **36** (2), S21-S25

O'Cathail, M., Sivanandan, M.A., Diver, C., Patel, P. & Christian, J. (2020) 'The use of patient-facing teleconsultations in the national health service: scoping review'. *JMIR medical informatics* **8** (3), e15380

Odaga, J., Sinclair, D., Lokong, J.A., Donegan, S., Hopkins, H. & Garner, P. (2014) 'Rapid diagnostic tests versus clinical diagnosis for managing people with fever in malaria endemic settings'. *Cochrane Database of Systematic Reviews* (4)

Odeh, B., Kayyali, R., Nabhani, S., Philip, N., Wallace, C., Wigmore, B., Robinson, P. & Griffiths, C. (2013) 'Impact of telehealth on patients' outcomes-A patients' perceptions evaluation study'. *International journal of pharmacy practice* **21**, 104-105

Odendaal, W.A., Anstey Watkins, J., Leon, N., Goudge, J., Griffiths, F., Tomlinson, M. & Daniels, K. (2020) 'Health workers’ perceptions and experiences of using mHealth technologies to deliver primary healthcare services: a qualitative evidence synthesis'. *Cochrane Database of Systematic Reviews* (3)

Oh-Ici, D., Wespi, P., Busch, J., Wissmann, L., Krajewski, M., Weiss, K., Sigfridsson, A., Messroghli, D. & Kozerke, S. (2016) 'Hyperpolarized Metabolic MR Imaging of Acute Myocardial Changes and Recovery after Ischemia-Reperfusion in a Small-Animal Model.'. *Radiology* **278** (3), 742-751

Okwundu, C.I., Nagpal, S., Musekiwa, A. & Sinclair, D. (2013) 'Home- or community-based programmes for treating malaria'. *Cochrane Database of Systematic Reviews* (5)

Olla, P. (2007) 'Mobile health technology of the future: creation of an M-Health taxonomy based on proximity'. *International Journal of Healthcare Technology and Management* **8** (3-4), 370-387

O'Mahony A.M., Garvey J.F. & McNicholas, W.T. (2020) 'Technologic advances in the assessment and management of obstructive sleep apnoea beyond the apnoea-hypopnoea index: A narrative review.'. *Journal of Thoracic Disease* **12** (9), 5020-5038

Omboni, S., McManus, R., Bosworth, H., Chappell, L., Green, B., Kario, K., Logan, A. & Magid, D. 'Evidence and recommendations on the use of telemedicine for the management of arterial hypertension'.

Omboni, S. (2019) 'Connected health in hypertension management'. *Frontiers in cardiovascular medicine* **6**, 76

Omboni, S., McManus, R.J., Bosworth, H.B., Chappell, L.C., Green, B.B., Kario, K., Logan, A.G., Magid, D.J., Mckinstry, B. & Margolis, K.L. (2020) 'Evidence and recommendations on the use of telemedicine for the management of arterial hypertension: an international expert position paper'. *Hypertension* **76** (5), 1368-1383

Omboni, S. (2019}) 'Connected Health in Hypertension Management'. *FRONTIERS IN CARDIOVASCULAR MEDICINE}* **6}**

Omboni, S., Mancinelli, A., Rizzi, F., Parati, G., Palatini, P., Benetti, E., Carretta, R., Cocco, F., D'Amico, F., Del Giudice, A., de Tschudy, A., Ferrara, A., Garavelli, G., Guadagni, C., Mazzocca, G., Pini, C., Rodighiero, M.P., Saladini, F., Vaccarella, A., Velardi, A., Veronesi, M., Di Guardo, A., Profeta, G., Filippi, A. & TEMPLAR TEleMonitoring Blood (2019}) 'Telemonitoring of 24-Hour Blood Pressure in Local Pharmacies and Blood Pressure Control in the Community: The Templar Project'. *AMERICAN JOURNAL OF HYPERTENSION}* **32}** (7}), 629-639}

Omboni, S., Panzeri, E. & Campolo, L. (2020) 'E-Health in Hypertension Management: an Insight into the Current and Future Role of Blood Pressure Telemonitoring'. *Current hypertension reports* **22** (6), 42

O'Meara, S., Al-Kurdi, D., Ologun, Y., Ovington, L.G., Martyn-St James, M. & Richardson, R. (2014) 'Antibiotics and antiseptics for venous leg ulcers'. *Cochrane Database of Systematic Reviews* (1)

Ong, M.K., Romano, P.S., Edgington, S., Aronow, H.U., Auerbach, A.D., Black, J.T., De Marco, T., Escarce, J.J., Evangelista, L.S., Hanna, B., Ganiats, T.G., Greenberg, B.H., Greenfield, S., Kaplan, S.H., Kimchi, A., Liu, H., Lombardo, D., Mangione, C.M., Sadeghi, B., Sadeghi, B., Sarrafzadeh, M., Tong, K., Fonarow, G.C. & Better Effectiveness After (2016}) 'Effectiveness of Remote Patient Monitoring After Discharge of Hospitalized Patients With Heart Failure The Better Effectiveness After Transition-Heart Failure (BEAT-HF) Randomized Clinical Trial'. *JAMA INTERNAL MEDICINE}* **176}** (3}), 310-318}

Osei, E. & Mashamba-Thompson, T.P. (2021) 'Mobile health applications for disease screening and treatment support in low-and middle-income countries: A narrative review'. *Heliyon* **7** (3), e06639

Ošmera, O. & Bulava, A. (2014) 'The benefits of remote monitoring in long-term care for patients with implantable cardioverter-defibrillators'. *Neuro endocrinology letters* **35 Suppl 1**, 40-48

Ospina-Pinillos, L., Davenport, T.A., Ricci, C.S., Milton, A.C., Scott, E.M. & Hickie, I.B. (2018) 'Developing a mental health eClinic to improve access to and quality of mental health care for young people: using participatory design as research methodologies'. *Journal of medical Internet research* **20** (5), e188

Ota, E., da Silva Lopes, K., Middleton, P., Flenady, V., Wariki, W., Rahman, M.O., Tobe-Gai, R. & Mori, R. (2020) 'Antenatal interventions for preventing stillbirth, fetal loss and perinatal death: an overview of Cochrane systematic reviews'. *Cochrane Database of Systematic Reviews* (12)

Otinwa, G.O. (2020) 'Epidemiology and Technology-The Past and Present: justification for modifiers in major non-communicable diseases'.

Ouellette, C. (2020) *A PROCESS MONITORING EVALUATION OF A NURSE-LED REMOTE AUTOMATED MONITORING AND VIRTUAL CARE INTERVENTION*

Owens, A.P. (2020) 'The Role of Heart Rate Variability in the Future of Remote Digital Biomarkers'. *Frontiers in Neuroscience* **14**, 582145

Oyo-Ita, A., Wiysonge, C.S., Oringanje, C., Nwachukwu, C.E., Oduwole, O. & Meremikwu, M.M. (2016) 'Interventions for improving coverage of childhood immunisation in low- and middle-income countries'. *Cochrane Database of Systematic Reviews* (7)

Ozdemir, V. (2019) 'The Big Picture on the "AI Turn" for Digital Health: The Internet of Things and Cyber-Physical Systems.'. *Omics a Journal of Integrative Biology* **23** (6), 308-311

Padeletti, L., Botto, G.L., Curnis, A., De Ruvo, E., D'Onofrio, A., Gronda, E., Ricci, R.P., Vado, A., Zanotto, G., Zecchin, M., Antoniou, X. & Gargaro, A. (2015) 'Selection of potential predictors of worsening heart failure: rational and design of the SELENE HF study.'. *Journal of Cardiovascular Medicine* **16** (11), 782-789

Paglialonga, A., Mastropietro, A., Scalco, E. & Rizzo, G. (2019) 'The mHealth'. In *m_Health Current and Future Applications* Springer, 5-17

Pahl, A., Young, L., Buus-Frank, M., Marcellus, L. & Soll, R. (2020) 'Non-pharmacological care for opioid withdrawal in newborns'. *Cochrane Database of Systematic Reviews* (12)

Pal, K., Eastwood, S.V., Michie, S., Farmer, A.J., Barnard, M.L., Peacock, R., Wood, B., Inniss, J.D. & Murray, E. (2013) 'Computer-based diabetes self-management interventions for adults with type 2 diabetes mellitus'. *Cochrane Database of Systematic Reviews* (3)

Palaniswamy, C., Mishkin, A., Aronow, W.S., Kalra, A. & Frishman, W.H. (2013}) 'Remote Patient Monitoring in Chronic Heart Failure'. *CARDIOLOGY IN REVIEW}* **21}** (3}), 141-150}

Paldan, K., Sauer, H. & Wagner, N. (2018) 'Promoting inequality? Self-monitoring applications and the problem of social justice'. *AI & SOCIETY* , 1-11

Palmer, K. & Parrott, J. (2007) 'Nine years' experience with home telehealth for cardiac surgery patients'. *Journal of Telemedicine & Telecare* **13**, S3:67-70

Pan, A., Lin, X., Hemler, E. & Hu, F.B. (2018) 'Diet and cardiovascular disease: advances and challenges in population-based studies'. *Cell metabolism* **27** (3), 489-496

Pandor, A., Thokala, P., Gomersall, T., Baalbaki, H., Stevens, J.W., Wang, J., Wong, R., Brennan, A. & Fitzgerald, P. (2013) 'Home telemonitoring or structured telephone support programmes after recent discharge in patients with heart failure: systematic review and economic evaluation'. *Health technology assessment (Winchester, England)* **17** (32), 1-207

Pandor, A., Thokala, P., Gomersall, T., Baalbaki, H., Stevens, J., Wang, J., Wong, R., Brennan, A. & Fitzgerald, P. (2013) 'Home telemonitoring or structured telephone support programmes after

recent discharge in patients with heart failure: systematic review and economic evaluation'. *Health technology assessment* **17** (32), 1-208

Pantoja, T., Abalos, E., Chapman, E., Vera, C. & Serrano, V.P. (2016) 'Oxytocin for preventing postpartum haemorrhage (PPH) in non-facility birth settings'. *Cochrane Database of Systematic Reviews* (4)

Pantoja, T., Opiyo, N., Lewin, S., Paulsen, E., Ciapponi, A., Wiysonge, C.S., Herrera, C.A., Rada, G., Peñaloza, B. & Dudley, L. (2017) 'Implementation strategies for health systems in low-income countries: an overview of systematic reviews'. *Cochrane Database of Systematic Reviews* (9)

Paras L., James E., Morgan P. & Lynagh, M. (2012) 'The FamilyFIT Program: Feasibility of an innovative familybased intervention designed to increase physical activity levels.'. *Journal of Science and Medicine in Sport.Conference: Be Active 2012.Sydney, NSW Australia.Conference Publication: (var.pagings)* **15** (SUPPL.1), S316

Parati, G., Dolan, E., McManus, R.J. & Omboni, S. (2018) 'Home blood pressure telemonitoring in the 21st century'. *Journal of clinical hypertension* **20** (7), 1128-1132

Parati, G. & Omboni, S. (2010}) 'Role of home blood pressure telemonitoring in hypertension management: an update'. *BLOOD PRESSURE MONITORING}* **15}** (6}), 285-295}

Park, L.G., Dracup, K., Whooley, M.A., McCulloch, C., Lai, S. & Howie-Esquivel, J. (2019) 'Sedentary lifestyle associated with mortality in rural patients with heart failure'. *European Journal of Cardiovascular Nursing* **18** (4), 318-324

Park, S., Bak, A., Kim, S., Nam, Y., Yoo, D. & Moon, M. (2020) 'Animal-Assisted and Pet-Robot Interventions for Ameliorating Behavioral and Psychological Symptoms of Dementia: A Systematic Review and Meta-Analysis'. *Biomedicines* **8** (6), 150

Park, C., Otobo, E., Ullman, J., Rogers, J., Fasihuddin, F., Garg, S., Kakkar, S., Goldstein, M., Chandrasekhar, S.V., Pinney, S. & Atreja, A. (2019) 'Impact on Readmission Reduction Among Heart Failure Patients Using Digital Health Monitoring: Feasibility and Adoptability Study'. *JMIR medical informatics* **7** (4), e13353

Park, D.K., Jung, E., Park, R.W., Lee, Y.H., Hwang, H.J., Son, I.A. & Hu, M. (2011) 'Telecare system for cardiac surgery patients: implementation and effectiveness.'. *Healthcare Informatics Research* **17** (2), 93-100

Parthiban, N., Esterman, A., Mahajan, R., Twomey, D.J., Pathak, R.K., Lau, D.H., Roberts-Thomson, K., Young, G.D., Sanders, P. & Ganesan, A.N. (2015) 'Remote Monitoring of Implantable Cardioverter-Defibrillators: A Systematic Review and Meta-Analysis of Clinical Outcomes'. *Journal of the American College of Cardiology (JACC)* **65** (24), 2591-2600

Pasco, D. & Roure, C. (2019) 'Impact of a technology-based physical education learning task on ninth grade students' situational interest'.

Patel, S., Park, H., Bonato, P., Chan, L. & Rodgers, M. (2012) 'A review of wearable sensors and systems with application in rehabilitation'. *Journal of neuroengineering and rehabilitation* **9** (1), 1-17

Payakachat, N., Rhoads, S., McCoy, H., Dajani, N., Eswaran, H. & Lowery, C. (2020) 'Using mHealth in postpartum women with pre-eclampsia: Lessons learned from a qualitative study'. *International Journal of Gynecology & Obstetrics* **149** (3), 339-346

Peahl, A.F., Novara, A., Heisler, M., Dalton, V.K., Moniz, M.H. & Smith, R.D. (2020) 'Patient Preferences for Prenatal and Postpartum Care Delivery: A Survey of Postpartum Women'. *Obstetrics & Gynecology* **135** (5), 1038-1046

Peahl, A.F., Novara, A., Heisler, M., Dalton, V.K., Moniz, M.H. & Smith, R.D. (2020) 'Patient Preferences for Prenatal and Postpartum Care Delivery: A Survey of Postpartum Women.'. *Obstetrics & Gynecology* **135** (5), 1038-1046

Pecchia, L., Bracale, U. & Bracale, M. (2009}) 'Health Technology Assessment of Home Monitoring for the Continuity of Care of patient suffering from congestive heart failure'. In Dossel, O. and Schlegel, W. (eds.) *WORLD CONGRESS ON MEDICAL PHYSICS AND BIOMEDICAL ENGINEERING, VOL 25, PT 12},* Held 233 SPRING STREET, NEW YORK, NY 10013, UNITED STATES}: SPRINGER}, 184}

Pecchia, L., Melillo, P. & Bracale, M. (2011) 'Remote health monitoring of heart failure with data mining via CART method on HRV features.'. *IEEE Transactions on Biomedical Engineering* **58** (3), 800-804

Pedretti, R.F., Fattirolli, F., Griffo, R., Ambrosetti, M., Angelino, E., Brazzo, S., Corrà, U., Dasseni, N., Faggiano, P. & Favretto, G. (2018) 'Cardiac Prevention and Rehabilitation "3.0": From acute to chronic phase. Position Paper of the Italian Association for Cardiovascular Prevention and Rehabilitation (GICR-IACPR)'. *Monaldi Archives for Chest Disease* **88** (3)

Peiris, D., Praveen, D., Mogulluru, K., Ameer, M.A., Raghu, A., Li, Q., Heritier, S., MacMahon, S., Prabhakaran, D. & Clifford, G.D. (2019) 'SMARThealth India: a stepped-wedge, cluster randomised controlled trial of a community health worker managed mobile health intervention for people assessed at high cardiovascular disease risk in rural India'. *PloS one* **14** (3), e0213708

Pekmezaris, R., Mitzner, I., Pecinka, K.R., Nouryan, C.N., Lesser, M.L., Siegel, M., Swiderski, J.W., Moise, G., Younker, R.S. & Smolich, K. (2012) 'The impact of remote patient monitoring (telehealth) upon Medicare beneficiaries with heart failure.'. *Telemedicine Journal & E-Health* **18** (2), 101-108

Peña-Rosas, J., Mithra, P., Unnikrishnan, B., Kumar, N., De-Regil, L., Nair, N.S., Garcia-Casal, M. & Solon, J.A. (2019) 'Fortification of rice with vitamins and minerals for addressing micronutrient malnutrition'. *Cochrane Database of Systematic Reviews* (10)

Penteado, S.P., Bento, R.F., Battistella, L.R., Silva, S.M. & Sooful, P. (2014) 'Use of the satisfaction with amplification in daily life questionnaire to assess patient satisfaction following remote hearing aid adjustments (telefitting)'. *JMIR Medical Informatics* **2** (2), e2769

Pépin, J., Jullian-Desayes, I., Sapène, M., Treptow, E., Joyeux-Faure, M., Benmerad, M., Bailly, S., Grillet, Y., Stach, B., Richard, P., Lévy, P., Muir, J. & Tamisier, R. (2019) 'Multimodal Remote Monitoring of High Cardiovascular Risk Patients With OSA Initiating CPAP: A Randomized Trial'. *Chest* **155** (4), 730-739

Perakslis, E.D. & Stanley, M. (2021) *Digital Health: Understanding the Benefit-risk Patient-provider Framework*Oxford University Press

Perales, C.L., Van Spall, H.G., Maeda, S., Jimenez, A., Laţcu, D.G., Milman, A., Kirakoya-Samadoulougou, F., Mamas, M.A., Muser, D. & Arroyo, R.C. (2020) 'Mobile health applications for the detection of atrial fibrillation: a systematic review'. *EP Europace*

Pereira, C., Kunczik, J., Bleich, A., Haeger, C., Kiessling, F., Thum, T., Tolba, R., Lindauer, U., Treue, S. & Czaplik, M. (2019) 'Perspective review of optical imaging in welfare assessment in animal-based research'. *Journal of Biomedical Optics* **24** (7), 1-11

Pereira, T., Pires, G., Jorge, D. & Santos, D. (2020) 'Telehealth monitoring of a hypertensive elderly patient with the new VITASENIOR-MT system: a case study.'. *Blood pressure monitoring* **25** (4), 227-230

Peretz, D., Arnaert, A. & Ponzoni, N.N. (2018) 'Determining the cost of implementing and operating a remote patient monitoring programme for the elderly with chronic conditions: A systematic review of economic evaluations'. *Journal of Telemedicine & Telecare* **24** (1), 13-21

Perings, C., Bauer, W.R., Bondke, H., Mewis, C., James, M., Bocker, D., Broadhurst, P., Korte, T., Toft, E., Hintringer, F., Clementy, J. & Schwab, J.O. (2011) 'Remote monitoring of implantable-cardioverter defibrillators: results from the Reliability of IEGM Online Interpretation (RIONI) study.'. *Europace* **13** (2), 221-229

Perings, S.M., Perings, C., Smetak, N., Meyer, C., Shin, D., Kelm, M. & Brachmann, J. (2013) 'Home Monitoring technology and integrated follow-up care of ICD patients.'. *Acta Cardiologica* **68** (4), 381-386

Perrin, T., Boveda, S., Defaye, P., Rosier, A., Sadoul, N., Bordachar, P., Klug, D., Ritter, P., Belhameche, M., Babuty, D., Mansourati, J., Lazarus, A. & Deharo, J. (2019) 'Role of medical reaction in management of inappropriate ventricular arrhythmia diagnosis: the inappropriate Therapy and HOme monitoRiNg (THORN) registry.'. *Europace* **21** (4), 607-615

Perry, T.T., Halterman, J.S., Brown, R.H., Hunter, C.R., Randle, S.M., Tilford, J.M. & Rettiganti, M. (2015) 'Breath connection: a school-based telemedicine program for rural children with asthma'. *The Journal of allergy and clinical immunology* **135** (2), AB169

Perry, T.T., Halterman, J.S., Brown, R.H., Luo, C., Randle, S.M., Hunter, C.R. & Rettiganti, M. (2018) 'Results of an asthma education program delivered via telemedicine in rural schools'. *Annals of allergy, asthma & immunology* **120** (4), 401-408

Peytremann-Bridevaux, I., Arditi, C., Gex, G., Bridevaux, P.O. & Burnand, B. (2015) 'Chronic disease management programmes for adults with asthma'. *Cochrane Database of Systematic Reviews* (5)

Phillips, S.A., Martino, S. & Arena, R. (2017) 'Research opportunities and challenges in the era of healthy living medicine: unlocking the potential'. *Progress in cardiovascular diseases* **59** (5), 498-505

Phoenix, C. & Tulle, E. (2017) 'Physical activity and ageing'. *The Routledge handbook of physical activity policy and practice.London: Routledge*

PIERGIOVANNI, L. & NINATTI, L. (2019) 'Digital transformation of the treatment of eating disorders: matching digital technologies and professionals' psychological determinants'.

Pigini, L., Bovi, G., Panzarino, C., Gower, V., Ferratini, M., andreoni, G., Sassi, R., Rivolta, M.W. & Ferrarin, M. (2017) 'Pilot Test of a New Personal Health System Integrating Environmental and Wearable Sensors for Telemonitoring and Care of Elderly People at Home (SMARTA Project)'. *Gerontology* **63** (3), 281-286

Piotrowicz, E., Pencina, M.J., Opolski, G., Zareba, W., Banach, M., Kowalik, I., Orzechowski, P., Szalewska, D., Pluta, S. & Glówczynska, R. (2020) 'Effects of a 9-Week Hybrid Comprehensive Telerehabilitation Program on Long-term Outcomes in Patients With Heart Failure: the

Telerehabilitation in Heart Failure Patients (TELEREH-HF) Randomized Clinical Trial'. *JAMA cardiology* **5** (3), 300-308

Piotrowicz, E., Piotrowicz, R., Opolski, G., Pencina, M., Banach, M. & Zaręba, W. (2019) 'Hybrid comprehensive telerehabilitation in heart failure patients (TELEREH-HF): a randomized, multicenter, prospective, open-label, parallel group controlled trial-Study design and description of the intervention'. *American Heart Journal* **217**, 148-158

Piotrowicz, E. (2017) 'The management of patients with chronic heart failure: the growing role of e-Health'. *Expert Review of Medical Devices* **14** (4), 271-277

Piotrowicz, E., Pencina, M.J., Opolski, G., Zareba, W., Banach, M., Kowalik, I., Orzechowski, P., Szalewska, D., Pluta, S., Glowczynska, R., Irzmanski, R., Oreziak, A., Kalarus, Z., Lewicka, E., Cacko, A., Mierzynska, A. & Piotrowicz, R. (2020) 'Effects of a 9-Week Hybrid Comprehensive Telerehabilitation Program on Long-term Outcomes in Patients With Heart Failure: The Telerehabilitation in Heart Failure Patients (TELEREH-HF) Randomized Clinical Trial.'. *JAMA Cardiology* **5** (3), 300-308

Piro, A., Magnocavallo, M., Della Rocca, D.G., Neccia, M., Manzi, G., Mariani, M.V., Straito, M., Bernardini, A., Severino, P., Iannucci, G., Giunta, G., Chimenti, C., Natale, A., Fedele, F. & Lavalle, C. (2020) 'Management of cardiac implantable electronic device follow-up in COVID-19 pandemic: Lessons learned during Italian lockdown'. *Journal of cardiovascular electrophysiology* **31** (11), 2814-2823

Pisetta, V., Morganti, E., Mase, M., Marsili, I.A., Adami, A. & Nollo, G. (2016}) 'The e-Cardiac Rehabilitation Service An integrated system for Home-Care Cardiac Rehabilitation'. In Anonymous (eds.) *IEEE SECOND INTERNATIONAL SMART CITIES CONFERENCE (ISC2 2016)},* Held 345 E 47TH ST, NEW YORK, NY 10017 USA}: IEEE}, 741-744}

Pistorius, C. (2017) 'Developments in emerging digital health technologies'. *DeltaHedron Innovation Insight* (1.1/17)

Plum, A., Tanniru, M. & Khuntia, J. (2020) 'An innovation platform for diffusing public health practices across a global network'. *Health Policy and Technology* **9** (2), 225-234

Poirot, E., Skarbinski, J., Sinclair, D., Kachur, S.P., Slutsker, L. & Hwang, J. (2013) 'Mass drug administration for malaria'. *Cochrane Database of Systematic Reviews* (12)

Polhemus, A.M., Novak, J., Ferrao, J., Simblett, S., Radaelli, M., Locatelli, P., Matcham, F., Kerz, M., Weyer, J., Burke, P., Huang, V., Dockendorf, M.F., Temesi, G., Wykes, T., Comi, G., Myin-Germeys, I., Folarin, A., Dobson, R., Manyakov, N.V., Narayan, V.A. & Hotopf, M. (2020) 'Human-Centered Design Strategies for Device Selection in mHealth Programs: Development of a Novel Framework and Case Study.'. *JMIR MHealth and UHealth* **8** (5), e16043

Pollock, A., Campbell, P., Cheyne, J., Cowie, J., Davis, B., McCallum, J., McGill, K., Elders, A., Hagen, S. & McClurg, D. (2020) 'Interventions to support the resilience and mental health of frontline health and social care professionals during and after a disease outbreak, epidemic or pandemic: a mixed methods systematic review'. *Cochrane Database of Systematic Reviews* (11)

Poppe, L. (2019) *Self-regulation-based eHealth promoting an active lifestyle in adults: a focus on users with type 2 diabetes*

Portaro, S., Calabro, R.S., Bramanti, P., Silvestri, G., Torrisi, M., Conti-Nibali, V., Caliri, S., Lunetta, C., Alagna, B., Naro, A. & Bramanti, A. (2018) 'Telemedicine for Facio-Scapulo-Humeral Muscular

Dystrophy: A multidisciplinary approach to improve quality of life and reduce hospitalization rate?.'. *Disability & Health Journal* **11** (2), 306-309

Poudyal, A., van Heerden, A., Hagaman, A., Maharjan, S.M., Byanjankar, P., Subba, P. & Kohrt, B.A. (2019) 'Wearable digital sensors to identify risks of postpartum depression and personalize psychological treatment for adolescent mothers: protocol for a mixed methods exploratory study in rural Nepal'. *JMIR research protocols* **8** (9), e14734

Pouwer, A.W., Farquhar, C. & Kremer, J. (2015) 'Long-acting FSH versus daily FSH for women undergoing assisted reproduction'. *Cochrane Database of Systematic Reviews* (7)

Prabhu, G., O'Connor, N.E. & Moran, K. (2020}) 'Recognition and Repetition Counting for Local Muscular Endurance Exercises in Exercise-Based Rehabilitation: A Comparative Study Using Artificial Intelligence Models'. *SENSORS}* **20}** (17})

Prabhu, G., O'Connor, N.E. & Moran, K. (2020) 'Recognition and Repetition Counting for Local Muscular Endurance Exercises in Exercise-Based Rehabilitation: A Comparative Study Using Artificial Intelligence Models.'. *Sensors* **20** (17)

Prabhu, S.R. (2016) 'The real value of IoT at home'. *Health management technology* **37** (6), 17-17

Prabhune, A.G., Mallawaram, A., Bhat, S. & Shagufta, A.M. (2021) 'Situational Analysis of the impact of COVID-19 pandemic on digital health research initiatives in South Asia'.

Pradhan, B., Bhattacharyya, S. & Pal, K. (2021) 'IoT-Based Applications in Healthcare Devices'. *Journal of healthcare engineering* **2021**

Pratap, A. (2019) *Assessing the utility of digital health technology to improve our capacity to assess and intervene in depression*

Prescher, S., Koehler, J. & Koehler, F. (2020) 'e-Health in cardiology: remote patient management of heart failure patients'. *E-Journal of Cardiology Practice* **18**, 26

Prescher, S., Schoebel, C., Koehler, K., Deckwart, O., Wellge, B., Honold, M., Hartmann, O., Winkler, S. & Koehler, F. (2016) 'Prognostic value of serial six-minute walk tests using tele-accelerometry in patients with chronic heart failure: A pre-specified sub-study of the TIM-HF-Trial.'. *European Journal of Preventive Cardiology* **23** (2 suppl), 21-26

Pron, G., Ieraci, L., Kaulback, K. & Medical Advisory Secretariat, Health Quality Ontario (2012) 'Internet-based device-assisted remote monitoring of cardiovascular implantable electronic devices: an evidence-based analysis'. *Ontario health technology assessment series* **12** (1), 1-86

Pryce, J., Richardson, M. & Lengeler, C. (2018) 'Insecticide-treated nets for preventing malaria'. *Cochrane Database of Systematic Reviews* (11)

Przybylski, A., Zakrzewska-Koperska, J., Maciag, A., Derejko, P., Orczykowski, M., Szumowski, L. & Walczak, F. (2009) 'Technical and practical aspects of remote monitoring of implantable cardioverter-defibrillator patients in Poland - preliminary results.'. *Kardiologia polska* **67** (5), 505-511

Ptomey, L.T., Washburn, R.A., Lee, J., Greene, J.L., Szabo-Reed, A., Sherman, J.R., Danon, J.C., Osborne, L.N., Little, T.D. & Donnelly, J.E. (2019) 'Individual and family-based approaches to increase physical activity in adolescents with intellectual and developmental disabilities: rationale and design for an 18 month randomized trial'. *Contemporary clinical trials* **84**, 105817

Ptomey, L.T., Washburn, R.A., Mayo, M.S., Greene, J.L., Lee, R.H., Szabo-Reed, A., Honas, J.J., Sherman, J.R. & Donnelly, J.E. (2018) 'Remote delivery of weight management for adults with intellectual and developmental disabilities: rationale and design for a 24 month randomized trial'. *Contemporary clinical trials* **73**, 16-26

Ptomey, L.T., Szabo, A.N., Willis, E.A., Greene, J.L., Danon, J.C., Washburn, R.A., Forsha, D.E. & Donnelly, J.E. (2018) 'Remote Exercise for Adults with Down Syndrome.'. *Translational Journal of the American College of Sports Medicine* **3** (8), 60-65

Pujdak, K. (2016) '[Implantable loop recorder of the Confirm family (St. Jude Medical)]'. *Herzschrittmachertherapie und Elektrophysiologie* **27** (4), 341-344

Pulantara, I.W., Parmanto, B. & Germain, A. (2018) 'Development of a Just-in-Time adaptive mHealth intervention for insomnia: usability study'. *JMIR human factors* **5** (2), e21

Puri, A., Kim, B., Nguyen, O., Stolee, P., Tung, J. & Lee, J. (2017) 'User acceptance of wrist-worn activity trackers among community-dwelling older adults: mixed method study'. *JMIR mHealth and uHealth* **5** (11), e173

Qiu, L., Xiang, M. & Wang, J. (2016) '[Experiences of applying home monitoring for cardiac implantable electronic devices]'. *Chung-Hua Hsin Hsueh Kuan Ping Tsa Chih [Chinese Journal of Cardiology]* **44** (1), 55-59

Queiros, A., Alvarelhao, J., Cerqueira, M., Silva, A.G., Santos, M. & Rocha, N.P. (2018}) 'Remote Care Technology: A Systematic Review of Reviews and Meta-Analyses'. *TECHNOLOGIES}* **6}** (1})

Quijoux, F., Bertin-Hugault, F., Zawieja, P., Lefèvre, M., Vidal, P. & Ricard, D. (2021) 'Postadychute-AG, Detection, and Prevention of the Risk of Falling Among Elderly People in Nursing Homes: Protocol of a Multicentre and Prospective Intervention Study'. *Frontiers in Digital Health* **2**, 59

Quinn, A., Ae-Ngibise, K., Wylie, B., Boamah, E., Schwartz, J., Mujtaba, M., Shimbo, D., Enuameh, Y., Abdalla, M. & Owusu-Agyei, S. (2015) 'Assessing the impact of household air pollution on health: feasibility of ambulatory blood pressure monitoring and repeat-assessment "home" blood pressure monitoring in a rural ghanaian setting'. *The American Journal of Tropical Medicine and Hygiene* **93** (4), 399-400

Quintiliani, L.M., Whiteley, J.A., Murillo, J., Lara, R., Jean, C., Quinn, E.K., Kane, J., Crouter, S.E., Heeren, T.C. & Bowen, D.J. (2021) 'Community health worker-delivered weight management intervention among public housing residents: A feasibility study'. *Preventive Medicine Reports* **22**, 101360

Quix, C., Barnickel, J., Geisler, S., Hassani, M., Kim, S., Li, X., Lorenz, A., Quadflieg, T., Gries, T. & Jarke, M. (2013) 'Healthnet: A system for mobile and wearable health information management'. *UMIC Research Cluster at RWTH Aachen University, Germany, db.disi.unitn.eu/pages/VLDBProgram/pdf/IMMo/paper8.pdf*

Rabipour, S. & Davidson, P.S. (2020) '31 Using Digital Technology for Cognitive Assessment and Enhancement in Older Adults'. *The Oxford Handbook of Digital Technologies and Mental Health* , 358

Rackal, J.M., Tynan, A.M., Handford, C.D., Rzeznikiewiz, D., Agha, A. & Glazier, R. (2011) 'Provider training and experience for people living with HIV/AIDS'. *Cochrane Database of Systematic Reviews* (6)

Radovic, A., McCarty, C.A., Katzman, K. & Richardson, L.P. (2018) 'Adolescents' perspectives on using technology for health: Qualitative study'. *JMIR pediatrics and parenting* **1** (1), e8677

Radovic, A. & Badawy, S.M. (2020) 'Technology Use for Adolescent Health and Wellness'. *Pediatrics* **145** (Suppl 2), S186-S194

Rahimi, K., Nazarzadeh, M., Pinho-Gomes, A., Woodward, M., Salimi-Khorshidi, G., Ohkuma, T., Fitzpatrick, R., Tarassenko, L., Denis, M., Cleland, J. & SUPPORT-HF2 Study Group (2020) 'Home monitoring with technology-supported management in chronic heart failure: a randomised trial.'. *Heart* **106** (20), 1573-1578

Raman, P., Shepherd, E., Dowswell, T., Middleton, P. & Crowther, C.A. (2017) 'Different methods and settings for glucose monitoring for gestational diabetes during pregnancy'. *Cochrane Database of Systematic Reviews* (10)

Randriambelonoro, M.M. (2020) *Self-monitoring technologies to promote healthy behavior in the long term*

Rangachari, P., Mushiana, S.S. & Herbert, K. (2021) 'A Narrative Review of Factors Historically Influencing Telehealth Use across Six Medical Specialties in the United States'. *International journal of environmental research and public health* **18** (9), 4995

Rangachari, P., Mushiana, S.S. & Herbert, K. (2021) 'Specialty-level Factors Historically Influencing Telehealth Use Can Provide Implications for Sustainability in a Post-Pandemic Era: A Systematic Review and Narrative Synthesis'.

Ranjan, K.R., Rohit, S., Dash, R. & Singh, R. (2021) 'Thinking, feeling and coping by BoP healthcare consumers: policy-based intervention in an emerging market'. *Journal of Marketing Management* , 1-48

Ranky, R.G. (2009) *Design & implementation of biomedical devices for evaluation & rehabilitation highlighting rapid prototyping tools & processes*

Rantz, M.J., Skubic, M., Alexander, G., Aud, M.A., Wakefield, B.J., Galambos, C., Koopman, R.J. & Miller, S.J. (2010) 'Improving nurse care coordination with technology'. *CIN: Computers, Informatics, Nursing* **28** (6), 325-332

Rao, C., Soemantri, S., Djaja, S., Suhardi, Adair, T., Wiryawan, Y., Pangaribuan, L., Irianto, J., Kosen, S. & Lopez, A.D. (2010) 'Mortality in Central Java: results from the indonesian mortality registration system strengthening project.'. *BMC Research Notes* **3**, 325

Rao, S., Thankachan, P., Amrutur, B., Washington, M. & Mony, P.K. (2018) 'Continuous, real-time monitoring of neonatal position and temperature during Kangaroo Mother Care using a wearable sensor: a techno-feasibility pilot study.'. *Pilot & Feasibility Studies* **4**, 99

Rao, U., de Vries, B., Ross, G.P. & Gordon, A. (2019) 'Fetal biometry for guiding the medical management of women with gestational diabetes mellitus for improving maternal and perinatal health'. *Cochrane Database of Systematic Reviews* (9)

Raposo, A., Marques, L., Correia, R., Melo, F., Valente, J., Pereira, T., Rosario, L.B., Froes, F., Sanches, J. & Silva, H.P.d. (2021) 'e-CoVig: A Novel mHealth System for Remote Monitoring of Symptoms in COVID-19.'. *Sensors* **21** (10)

Rashid, R.b.A., Ch'ng, H.S., Alias, M.A. & Fisal, N. (2005}) 'Real time medical data acquisition over wireless ad-hoc network'. In Esa, M., Habash, R., Baba, N., *et al*. (eds.) *APACE: 2005 ASIA-*

PACIFIC CONFERENCE ON APPLIED ELECTROMAGNETICS, PROCEEDINGS}, Held 345 E 47TH ST, NEW YORK, NY 10017 USA}: IEEE}, 383-387}

Rashidian, A., Omidvari, A.H., Vali, Y., Sturm, H. & Oxman, A.D. (2015) 'Pharmaceutical policies: effects of financial incentives for prescribers'. *Cochrane Database of Systematic Reviews* (8)

Rawstorn, J. (2016) *Breaking accessibility barriers: development & evaluation of an mHealth platform for remotely delivered exercise-based cardiac rehabilitation*

Rawstorn, J.C., Gant, N., Warren, I., Doughty, R.N., Lever, N., Poppe, K.K. & Maddison, R. (2015) 'Measurement and Data Transmission Validity of a Multi-Biosensor System for Real-Time Remote Exercise Monitoring Among Cardiac Patients'. *JMIR rehabilitation and assistive technologies* **2** (1), e2

Rawstorn, J.C., Gant, N., Meads, A., Warren, I. & Maddison, R. (2016) 'Remotely Delivered Exercise-Based Cardiac Rehabilitation: Design and Content Development of a Novel mHealth Platform.'. *JMIR MHealth and UHealth* **4** (2), e57

Rawstorn, J.C., Gant, N., Rolleston, A., Whittaker, R., Stewart, R., Benatar, J., Warren, I., Meads, A., Jiang, Y. & Maddison, R. (2018) 'End Users Want Alternative Intervention Delivery Models: Usability and Acceptability of the REMOTE-CR Exercise-Based Cardiac Telerehabilitation Program.'. *Archives of Physical Medicine & Rehabilitation* **99** (11), 2373-2377

Ray, M.K., McMichael, A., Rivera-Santana, M., Noel, J. & Hershey, T. (2021) 'Technological Ecological Momentary Assessment Tools to Study Type 1 Diabetes in Youth: Viewpoint of Methodologies'. *JMIR diabetes* **6** (2), e27027

Ray, P., Li, J., Ariani, A. & Kapadia, V. (2017) 'Tablet-based well-being check for the elderly: development and evaluation of usability and acceptability'. *JMIR human factors* **4** (2), e12

Ray, S. & Ray, A. (2016) 'Non-surgical interventions for treating heavy menstrual bleeding (menorrhagia) in women with bleeding disorders'. *Cochrane Database of Systematic Reviews* (11)

Raywood, E., Douglas, H., Kapoor, K., Filipow, N., Murray, N., O'Connor, R., Stott, L., Saul, G., Kuzhagaliyev, T., Davies, G., Liakhovich, O., Van Schaik, T., Furtuna, B., Booth, J., Shannon, H., Bryon, M. & Main, E. (2020) 'Protocol for Project Fizzyo, an analytic longitudinal observational cohort study of physiotherapy for children and young people with cystic fibrosis, with interrupted time-series design.'. *BMJ Open* **10** (10), e039587

Realdon, O., Rossetto, F., Nalin, M., Baroni, I., Romano, M., Catania, F., Mancastroppa, S., Frontini, D., Bandini, S. & Nemni, R. (2016) 'Experienced usability and adherence to treatment in a technology-enhanced continuum of care program with respect to usual care in MCI and outpatients with Alzheimer: the ability program'. *Journal of Alzheimer's Disease* **52**, S83-S84

Recio-Rodriguez, J.I., Agudo Conde, C., Calvo-Aponte, M.J., Gonzalez-Viejo, N., Fernandez-Alonso, C., Mendizabal-Gallastegui, N., Rodriguez-Martin, B., Maderuelo-Fernandez, J.A., Rodriguez-Sanchez, E. & Gomez-Marcos, M.A. (2018) 'The effectiveness of a smartphone application on modifying the intakes of macro and micronutrients in primary care: a randomized controlled trial. The EVIDENT II study'. *Nutrients* **10** (10), 1473

Redmond, P., Grimes, T.C., McDonnell, R., Boland, F., Hughes, C. & Fahey, T. (2018) 'Impact of medication reconciliation for improving transitions of care'. *Cochrane Database of Systematic Reviews* (8)

Redmond, S.J., Lovell, N.H., Basilakis, J. & Celler, B.G. (2008) 'ECG quality measures in telecare monitoring.'. *Annual International Conference Of The IEEE Engineering In Medicine And Biology Society* **2008**, 2869-2872

Reichert, M., Giurgiu, M., Koch, E., Wieland, L.M., Lautenbach, S., Neubauer, A.B., von Haaren-Mack, B., Schilling, R., Timm, I. & Notthoff, N. (2020) 'Ambulatory assessment for physical activity research: state of the science, best practices and future directions'. *Psychology of Sport and Exercise* , 101742

Reilly, E.D., Robinson, S.A., Petrakis, B.A., Kuhn, E., Pigeon, W.R., Wiener, R.S., McInnes, D.K. & Quigley, K.S. (2019) 'Mobile app use for insomnia self-management: pilot findings on sleep outcomes in veterans'. *Interactive journal of medical research* **8** (3), e12408

Reinkensmeyer, D.J., Bonato, P., Boninger, M.L., Chan, L., Cowan, R.E., Fregly, B.J. & Rodgers, M.M. (2011) 'European research and development in mobility technology for people with disabilities'. *Renner, CI, Outermans, J., Ludwig, R., Brendel, C., Kwakkel, G., & Hummelsheim, H.(2016).Group therapy task training versus individual task training during inpatient stroke rehabilitation: A randomised controlled trial.Clinical rehabilitation* **30** (7), 637-648

Remond, M.G.W., Stewart, S., Carrington, M.J., Marwick, T.H., Kingwell, B.A., Meikle, P., O'Brien, D., Marshall, N.S. & Maguire, G.P. (2017) 'Better Indigenous Risk stratification for Cardiac Health study (BIRCH) protocol: rationale and design of a cross-sectional and prospective cohort study to identify novel cardiovascular risk indicators in Aboriginal Australian and Torres Strait Islander adults.'. *BMC Cardiovascular Disorders* **17** (1), 228

Ren, X., Apostolakos, C., Vo, T.H., Shaw, R.E., Shields, K., Banki, N.M., Zuckermann, D.W., Flint, A.C., Hongo, R.H. & Goldschlager, N.F. (2013) 'Remote monitoring of implantable pacemakers: in-office setup significantly improves successful data transmission.'. *Clinical cardiology* **36** (10), 634-637

Res, J.C.J., Theuns, D.A.M.J. & Jordaens, L. (2006) 'The role of remote monitoring in the reduction of inappropriate implantable cardioverter defibrillator therapies'. *Clinical Research in Cardiology* **95** (Suppl 3), 17-21

Rezvani F., Harter M. & Dirmaier, J. (2018) 'Promoting a home-based walking exercise using telephone-based health coaching and activity monitoring for patients with intermittent claudication (TeGeCoach): Protocol for a randomized controlled trial.'. *PPmP Psychotherapie Psychosomatik Medizinische Psychologie.Conference: Global Health Challenges to Community-Based and Individual Psychosocial Intervention Strategies - Gemeinsamer Kongress der DGMP und der DGMS.Leipzig Germany* **68** (8), e43

Ricci, R.P., Morichelli, L. & Santini, M. (2009) 'Remote control of implanted devices through Home Monitoring technology improves detection and clinical management of atrial fibrillation'. *Europace : European pacing, arrhythmias, and cardiac electrophysiology : journal of the working groups on cardiac pacing, arrhythmias, and cardiac cellular electrophysiology of the European Society of Cardiology* **11** (1), 54-61

Ricci, R.P., Morichelli, L. & Santini, M. (2008) 'Home monitoring remote control of pacemaker and implantable cardioverter defibrillator patients in clinical practice: impact on medical management and health-care resource utilization'. *Europace : European pacing, arrhythmias, and cardiac electrophysiology : journal of the working groups on cardiac pacing, arrhythmias, and cardiac cellular electrophysiology of the European Society of Cardiology* **10** (2), 164-170

Ricci, R.P., Morichelli, L., Gargaro, A., Laudadio, M.T. & Santini, M. (2009) 'Home monitoring in patients with implantable cardiac devices: is there a potential reduction of stroke risk? Results

from a computer model tested through monte carlo simulations.'. *Journal of cardiovascular electrophysiology* **20** (11), 1244-1251

Ricci, R.P., Vicentini, A., D'Onofrio, A., Sagone, A., Vincenti, A., Padeletti, L., Morichelli, L., Fusco, A., Vecchione, F., Lo Presti, F., Denaro, A., Pollastrelli, A. & Santini, M. (2013) 'Impact of in-clinic follow-up visits in patients with implantable cardioverter defibrillators: demographic and socioeconomic analysis of the TARIFF study population.'. *Journal of Interventional Cardiac Electrophysiology* **38** (2), 101-106

Ricci, R.P. (2013) 'Disease management: atrial fibrillation and home monitoring.'. *Europace* **15** (Suppl 1), 35-39

Ricci, R.P. & Glotzer, T.V. (2015) 'Home Screening for Detecting Subclinical Atrial Fibrillation'. *Journal of Atrial Fibrillation* **8** (4), 1326

Ricci, R.P. & Morichelli, L. (2013) 'Workflow, time and patient satisfaction from the perspectives of home monitoring.'. *Europace* **15** (Suppl 1), 49-53

Ricci, R.P., Morichelli, L., D'onofrio, A., CalÒ, L., Vaccari, D., Zanotto, G., Curnis, A., Buja, G., Rovai, N. & Gargaro, A. (2014) 'Manpower and Outpatient Clinic Workload for Remote Monitoring of Patients with Cardiac Implantable Electronic Devices: Data from the HomeGuide Registry'. *Journal of cardiovascular electrophysiology* **25** (11), 1216-1223

Ricci, R.P., Morichelli, L., D'Onofrio, A., Calò, L., Vaccari, D., Zanotto, G., Curnis, A., Buja, G., Rovai, N. & Gargaro, A. (2013) 'Effectiveness of remote monitoring of CIEDs in detection and treatment of clinical and device-related cardiovascular events in daily practice: the HomeGuide Registry'. *EP: Europace* **15** (7), 970-977

Ricci, R.P., Morichelli, L., D'Onofrio, A., Calo, L., Vaccari, D., Zanotto, G., Curnis, A., Buja, G., Rovai, N. & Gargaro, A. (2014) 'Manpower and outpatient clinic workload for remote monitoring of patients with cardiac implantable electronic devices: data from the HomeGuide Registry.'. *Journal of cardiovascular electrophysiology* **25** (11), 1216-1223

Ricci, R.P., Morichelli, L., Quarta, L., Porfili, A., Magris, B., Giovene, L., Torcinaro, S. & Gargaro, A. (2015) 'Effect of daily remote monitoring on pacemaker longevity: a retrospective analysis.'. *Heart Rhythm* **12** (2), 330-337

Ricci, R.P., Morichelli, L., Quarta, L., Sassi, A., Porfili, A., Laudadio, M.T., Gargaro, A. & Santini, M. (2010) 'Long-term patient acceptance of and satisfaction with implanted device remote monitoring.'. *Europace* **12** (5), 674-679

Ricci, R.P., Morichelli, L. & Santini, M. (2009) 'Remote control of implanted devices through Home Monitoring technology improves detection and clinical management of atrial fibrillation.'. *Europace* **11** (1), 54-61

Ricci, R.P., Morichelli, L. & Santini, M. (2008) 'Home monitoring remote control of pacemaker and implantable cardioverter defibrillator patients in clinical practice: impact on medical management and health-care resource utilization.'. *Europace* **10** (2), 164-170

Ricci, R.P., Vaccari, D., Morichelli, L., Zanotto, G., Calo, L., D'Onofrio, A., Curnis, A., Pisano, E.C.L., Nangah, R., Brieda, M., Calzolari, V., Melissano, D., Rovai, N. & Gargaro, A. (2016) 'Stroke incidence in patients with cardiac implantable electronic devices remotely controlled with automatic alerts of atrial fibrillation. A sub-analysis of the HomeGuide study.'. *International journal of cardiology* **219**, 251-256

Richards, J., Hillsdon, M., Thorogood, M. & Foster, C. (2013) 'Face-to-face interventions for promoting physical activity'. *Cochrane Database of Systematic Reviews* (9)

Richardson, V.F. & King, A.S. (1995) 'A new method of infant monitoring: for use at home and hospital.'. *New Zealand Medical Journal* **108** (994), 61-62

Richter, B., Hemmingsen, B., Metzendorf, M.I. & Takwoingi, Y. (2018) 'Development of type 2 diabetes mellitus in people with intermediate hyperglycaemia'. *Cochrane Database of Systematic Reviews* (10)

Richter, B. & Neises, G. (2005) ''Human' insulin versus animal insulin in people with diabetes mellitus'. *Cochrane Database of Systematic Reviews* (1)

Riegel, B., Moser, D.K., Buck, H.G., Dickson, V.V., Dunbar, S.B., Lee, C.S., Lennie, T.A., Lindenfeld, J., Mitchell, J.E. & Treat-Jacobson, D.J. (2017) 'Self-care for the prevention and management of cardiovascular disease and stroke: A scientific statement for healthcare professionals from the American Heart Association'. *Journal of the American Heart Association* **6** (9), e006997

Riley W.T., Keberlein P., Sorenson G., Mohler S., Tye B., Ramirez A.S. & Carroll, M. (2015) 'Program evaluation of remote heart failure monitoring: healthcare utilization analysis in a rural regional medical center.'. *Telemedicine journal and e-health : the official journal of the American Telemedicine Association* **21** (3), 157-162

Riley, W.T., Keberlein, P., Sorenson, G., Mohler, S., Tye, B., Ramirez, A.S. & Carroll, M. (2015) 'Program evaluation of remote heart failure monitoring: healthcare utilization analysis in a rural regional medical center.'. *Telemedicine Journal & E-Health* **21** (3), 157-162

Rini, C., Williams, D.A., Broderick, J.E. & Keefe, F.J. (2012) 'Meeting them where they are: using the internet to deliver behavioral medicine interventions for pain'. *Translational behavioral medicine* **2** (1), 82-92

Rivas, C., Ramsay, J., Sadowski, L., Davidson, L.L., Dunne, D., Eldridge, S., Hegarty, K., Taft, A. & Feder, G. (2015) 'Advocacy interventions to reduce or eliminate violence and promote the physical and psychosocial well-being of women who experience intimate partner abuse'. *Cochrane Database of Systematic Reviews* (12)

Rivas, C., Vigurs, C., Cameron, J. & Yeo, L. (2019) 'A realist review of which advocacy interventions work for which abused women under what circumstances'. *Cochrane Database of Systematic Reviews* (6)

Robbins, R., Affouf, M., Weaver, M.D., Czeisler, M.É., Barger, L.K., Quan, S.F. & Czeisler, C.A. (2021) 'Estimated Sleep Duration Before and During the COVID-19 Pandemic in Major Metropolitan Areas on Different Continents: Observational Study of Smartphone App Data'. *Journal of medical Internet research* **23** (2), e20546

Roberts, L., Ahmed, I. & Davison, A. (2009) 'Intercessory prayer for the alleviation of ill health'. *Cochrane Database of Systematic Reviews* (2)

Robertson, S.A.H. (2021) *Factors Affecting Adoption of Wearable Biometric-Tracking Devices: A Predictive Study*

Robertson, L. & Strachan, J. (2017) 'Subcutaneous unfractionated heparin for the initial treatment of venous thromboembolism'. *Cochrane Database of Systematic Reviews* (2)

Robinson, A., Oksuz, U., Slight, R., Slight, S. & Husband, A. (2020) 'Digital and Mobile Technologies to Promote Physical Health Behavior Change and Provide Psychological Support for Patients Undergoing Elective Surgery: Meta-Ethnography and Systematic Review'. *JMIR mHealth and uHealth* **8** (12), e19237

Robinson, N.L., Cottier, T.V. & Kavanagh, D.J. (2019) 'Psychosocial health interventions by social robots: systematic review of randomized controlled trials'. *Journal of medical Internet research* **21** (5), e13203

Rodler, S., Buchner, A., Stief, C.G., Heinemann, V., Staehler, M. & Casuscelli, J. (2021) 'Patients' perspective on digital technologies in advanced genitourinary cancers'. *Clinical Genitourinary Cancer* **19** (1), 76-82. e6

Rohwer, A.C., Oladapo, O.T. & Hofmeyr, G.J. (2020) 'Strategies for optimising antenatal corticosteroid administration for women with anticipated preterm birth'. *Cochrane Database of Systematic Reviews* (5)

Rojas-Reyes, M., Granados Rugeles, C. & Charry-Anzola, L. (2014) 'Oxygen therapy for lower respiratory tract infections in children between 3 months and 15 years of age'. *Cochrane Database of Systematic Reviews* (12)

Rolfe, A., Cash-Gibson, L., Car, J., Sheikh, A. & McKinstry, B. (2014) 'Interventions for improving patients' trust in doctors and groups of doctors'. *Cochrane Database of Systematic Reviews* (3)

Ronco, C., Davenport, A. & Gura, V. (2011) 'The future of the artificial kidney: moving towards wearable and miniaturized devices'. *Nefrologia* **31** (1), 9-16

Rooij, T.v. & Marsh, S. (2016) 'eHealth: past and future perspectives'. *Personalized Medicine* **13** (1), 57-70

Rosa, C., Marsch, L.A., Winstanley, E.L., Brunner, M. & Campbell, A.N. (2021) 'Using digital technologies in clinical trials: Current and future applications'. *Contemporary Clinical Trials* **100**, 106219

Rosen, J.M., Adams, L.V., Geiling, J., Curtis, K.M., Mosher, R.E., Ball, P.A., Grigg, E.B., Hebert, K.A., Grodan, J.R., Jurmain, J.C., Loucks, C., Macedonia, C.R. & Kun, L. (2021) 'Telehealth's New Horizon: Providing Smart Hospital-Level Care in the Home.'. *Telemedicine Journal & E-Health*

Rösner, S., Englbrecht, C., Wehrle, R., Hajak, G. & Soyka, M. (2018) 'Eszopiclone for insomnia'. *Cochrane Database of Systematic Reviews* (10)

Rosner, M.H., Lew, S.Q., Conway, P., Ehrlich, J., Jarrin, R., Patel, U.D., Rheuban, K., Robey, R.B., Sikka, N., Wallace, E., Brophy, P. & Sloand, J. (2017) 'Perspectives from the Kidney Health Initiative on Advancing Technologies to Facilitate Remote Monitoring of Patient Self-Care in RRT'. *Clinical Journal of The American Society of Nephrology: CJASN* **12** (11), 1900-1909

Rössler, R., Bridenbaugh, S.A., Engelter, S.T., Weibel, R., Infanger, D., Giannouli, E., Sofios, A., Iendra, L., Portegijs, E. & Rantanen, T. (2020) 'Recovery of mobility function and life-space mobility after ischemic stroke: the MOBITEC-Stroke study protocol'. *BMC neurology* **20** (1), 1-11

Rotter, T., Kinsman, L., James, E.L., Machotta, A., Gothe, H., Willis, J., Snow, P. & Kugler, J. (2010) 'Clinical pathways: effects on professional practice, patient outcomes, length of stay and hospital costs'. *Cochrane Database of Systematic Reviews* (3)

Rovaris, G., Solimene, F., D'Onofrio, A., Zanotto, G., Ricci, R.P., Mazzella, T., Iacopino, S., Della Bella, P., Maglia, G., Senatore, G., Quartieri, F., Biffi, M., Curnis, A., Calvi, V., Rapacciuolo, A., Santamaria, M., Capucci, A., Giammaria, M., Campana, A. & Caravati, F. (2018) 'Does the CHA2DS2-VASc score reliably predict atrial arrhythmias? Analysis of a nationwide database of remote monitoring data transmitted daily from cardiac implantable electronic devices'. *Heart Rhythm* **15** (3), N.PAG-N.PAG

Rowlands, D. (2020) 'Australian Digital Health Agency'.

Roy, M. 'Digital Health Evolution and propagation in Rural Healthcare in India: A Review'.

Rubin, D.S., Severin, R., Arena, R. & Bond, S. (2020) 'Leveraging technology to move more and sit less'. *Progress in cardiovascular diseases*

Rumsfeld, J.S., Brooks, S.C., Aufderheide, T.P., Leary, M., Bradley, S.M., Nkonde-Price, C., Schwamm, L.H., Jessup, M., Ferrer, J.M.E. & Merchant, R.M. (2016) 'Use of mobile devices, social media, and crowdsourcing as digital strategies to improve emergency cardiovascular care: a scientific statement from the American Heart Association'. *Circulation* **134** (8), e87-e108

Rundo, L., Pirrone, R., Vitabile, S., Sala, E. & Gambino, O. (2020) 'Recent advances of HCI in decision-making tasks for optimized clinical workflows and precision medicine'. *Journal of Biomedical Informatics* , 103479

Russak, A.J., Chaudhry, F., De Freitas, J.K., Baron, G., Chaudhry, F.F., Bienstock, S., Paranjpe, I., Vaid, A., Ali, M. & Zhao, S. (2020) 'Machine Learning in Cardiology—Ensuring Clinical Impact Lives Up to the Hype'. *Journal of cardiovascular pharmacology and therapeutics* **25** (5), 379-390

Rylance, J., Gordon, S.B., Naeher, L.P., Patel, A., Balmes, J.R., Adetona, O., Rogalsky, D.K. & Martin, I.,William J. (2013}) 'Household air pollution: a call for studies into biomarkers of exposure and predictors of respiratory disease'. *AMERICAN JOURNAL OF PHYSIOLOGY-LUNG CELLULAR AND MOLECULAR PHYSIOLOGY}* **304}** (9}), 571-578}

Sacher, F., Probst, V., Bessouet, M., Wright, M., Maluski, A., Abbey, S., Bordachar, P., Deplagne, A., Ploux, S., Lande, G., Jais, P., Hocini, M., Haissaguerre, M., Le Marec, H. & Clementy, J. (2009) 'Remote implantable cardioverter defibrillator monitoring in a Brugada syndrome population.'. *Europace* **11** (4), 489-494

Sachpazidis, I. (2008) *Image and medical data communication protocols for telemedicine and teleradiology*

Sadeh, M., Brauer, M., Dankner, R., Fulman, N. & Chudnovsky, A. (2021) 'Remote sensing metrics to assess exposure to residential greenness in epidemiological studies: A population case study from the Eastern Mediterranean.'. *Environment international* **146**, 106270

Sadek, I. & Mohktari, M. (2018) 'Nonintrusive Remote Monitoring of Sleep in Home-Based Situation.'. *Journal of medical systems* **42** (4), 64

Saeterdal, I., Lewin, S., Austvoll-Dahlgren, A., Glenton, C. & Munabi-Babigumira, S. (2014) 'Interventions aimed at communities to inform and/or educate about early childhood vaccination'. *Cochrane Database of Systematic Reviews* (11)

Safarikova, I., Bulava, A. & Hajek, P. (2020) 'Remote monitoring of implantable cardioverters defibrillators: a comparison of acceptance between octogenarians and younger patients.'. *Journal of Geriatric Cardiology* **17** (7), 417-426

Sahu, M.L., Atulkar, M. & Ahirwal, M.K. (2021) 'IOT-Based Smart Healthcare System: A Review on Constituent Technologies'. *Journal of Circuits, Systems and Computers* , 2130008

Samhan, B., Crampton, T. & Ruane, R. (2018) 'The Trajectory of IT in Healthcare at HICSS: A Literature Review, Analysis, and Future Directions'. *Communications of the Association for Information Systems* **43** (1), 41

Sana, F., Isselbacher, E.M., Singh, J.P., Heist, E.K., Pathik, B. & Armoundas, A.A. (2020) 'Wearable Devices for Ambulatory Cardiac Monitoring: JACC State-of-the-Art Review'. *Journal of the American College of Cardiology (JACC)* **75** (13), 1582-1592

Sandifer, P.A., Knapp, L.C., Lichtveld, M.Y., Manley, R.E., Abramson, D., Caffey, R., Cochran, D., Collier, T.K., Ebi, K. & Engel, L. (2020) 'Framework for a community health observing system for the Gulf of Mexico region: preparing for future disasters'. *Frontiers in public health* **8**, 588

Saner, H., Morger, C., Eser, P. & von Planta, M. (2013) 'Dual dispatch early defibrillation in out-of-hospital cardiac arrest in a mixed urban-rural population.'. *Resuscitation* **84** (9), 1197-1202

Sa-Ngasoongsong, A., Kunthong, J., Sarangan, V., Cai, X. & Bukkapatnam, S.T. (2012) 'A low-cost, portable, high-throughput wireless sensor system for phonocardiography applications'. *Sensors (Basel, Switzerland)* **12** (8), 10851-10870

Sangster, J., Furber, S., Allman-Farinelli, M., Phongsavan, P., Redfern, J., Haas, M., Church, J., Mark, A. & Bauman, A. (2015) 'Effectiveness of a pedometer-based telephone coaching program on weight and physical activity for people referred to a cardiac rehabilitation program: a randomized controlled trial.'. *Journal of Cardiopulmonary Rehabilitation & Prevention* **35** (2), 124-129

Santamore, W.P., Homko, C.J., Kashem, A., McConnell, T.R., Menapace, F.J. & Bove, A.A. (2008) 'Accuracy of blood pressure measurements transmitted through a telemedicine system in underserved populations.'. *Telemedicine Journal & E-Health* **14** (4), 333-338

Santiago de Araújo Pio, C, Chaves, G., Davies, P., Taylor, R.S. & Grace, S.L. (2019) 'Interventions to promote patient utilisation of cardiac rehabilitation'. *Cochrane Database of Systematic Reviews* (2)

Santini, M., Ricci, R.P., Lunati, M., Landolina, M., Perego, G.B., Marzegalli, M., Schirru, M., Belvito, C., Brambilla, R., Guenzati, G., Gilardi, S., Valsecchi, S., Santini, M., Ricci, R.P., Lunati, M., Landolina, M., Perego, G.B., Marzegalli, M., Schirru, M. & Belvito, C. (2009) 'Remote monitoring of patients with biventricular defibrillators through the CareLink system improves clinical management of arrhythmias and heart failure episodes'. *Journal of Interventional Cardiac Electrophysiology* **24** (1), 53-61

Santos, M.A., Munoz, R., Olivares, R., Rebouças Filho, P.P., Del Ser, J. & de Albuquerque, Victor Hugo C (2020) 'Online heart monitoring systems on the internet of health things environments: A survey, a reference model and an outlook'. *Information Fusion* **53**, 222-239

Saran T., Pedrycz A. & Mucha, D. (2018) 'Follow-up monitoring of physical activity after rehabilitation by means of a mobile application: Effectiveness of measurements in different age groups.'. *Advances in Clinical and Experimental Medicine* **27** (8), 1037-1044

Särelä, A., Korhonen, I., Salminen, J., Koskinen, E., Kirkeby, O. & Walters, D. (2009) 'A home-based care model for outpatient cardiac rehabilitation based on mobile technologies'. In Anonymous (eds.) *2009 3rd International Conference on Pervasive Computing Technologies for Healthcare,* Held IEEE, 1-8

Satava, R., Angood, P.B., Harnett, B., Macedonia, C. & Merrell, R. (2000) 'The physiologic cipher at altitude: telemedicine and real-time monitoring of climbers on Mount Everest.'. *Telemedicine Journal & E-Health* **6** (3), 303-313

Sato, A., Asayama, K., Ohkubo, T., Kikuya, M., Obara, T., Metoki, H., Inoue, R., Hara, A., Hoshi, H., Hashimoto, J., Totsune, K., Satoh, H., Oka, Y. & Imai, Y. (2008) 'Optimal cutoff point of waist circumference and use of home blood pressure as a definition of metabolic syndrome: the Ohasama study.'. *American Journal of Hypertension* **21** (5), 514-520

Satou, G.M., Rheuban, K., Alverson, D., Lewin, M., Mahnke, C., Marcin, J., Martin, G.R., Mazur, L.S., Sahn, D.J., Shah, S., Tuckson, R., Webb, C.L., Sable, C.A., Council Cardiovasc Dis Young & Council Quality Care Outcomes Res (2017}) 'Telemedicine in Pediatric Cardiology A Scientific Statement From the American Heart Association'. *CIRCULATION}* **135}** (11}), E648-E678}

Sawmynaden, P., Atherton, H., Majeed, A. & Car, J. (2012) 'Email for the provision of information on disease prevention and health promotion'. *Cochrane Database of Systematic Reviews* (11)

Scalvini, S., Capomolla, S., Zanelli, E., Benigno, M., Domenighini, D., Paletta, L., Glisenti, F. & Giordano, A. (2005) 'Effect of home-based telecardiology on chronic heart failure: costs and outcomes.'. *Journal of Telemedicine & Telecare* **11** (Suppl 1), 16-18

Scalvini, S., Martinelli, G., Baratti, D., Domenighini, D., Benigno, M., Paletta, L., Zanelli, E. & Giordano, A. (2005) 'Telecardiology: one-lead electrocardiogram monitoring and nurse triage in chronic heart failure.'. *Journal of Telemedicine & Telecare* **11** (Suppl 1), 18-20

Scalvini, S., Zanelli, E., Volterrani, M., Martinelli, G., Baratti, D., Buscaya, O., Baiardo, P., Glisenti, F. & Giordano, A. (2004) 'A pilot study of nurse-led, home-based telecardiology for patients with chronic heart failure'. *Journal of Telemedicine & Telecare* **10** (2), 113-117

Scalvini, S. (2005) '[Telecardiology and general medicine]'. *Monaldi Archives for Chest Disease* **64** (2), 147-148

Scalvini, S., Comini, L. & Bernocchi, P. (2020) 'How can multidisciplinary management with remote monitoring improve the outcome of patients with chronic cardiac diseases?.'. *Expert Review of Medical Devices* **17** (3), 153-157

Scarpato, N., Pieroni, A., Di Nunzio, L. & Fallucchi, F. (2017) 'E-health-IoT universe: A review'. *management* **21** (44), 46

Schaefer, S.E., Gomez-Camacho, R., Martinez, L., Sadeghi, B., German, J.B. & de la Torre, A. (2016) 'Social and environmental determinants of child physical activity in a rural Mexican-origin community'. *Journal of community health* **41** (2), 409-416

Schenkel, F.A., Barr, M.L., McCloskey, C.C., Possemato, T., O'Conner, J., Sadeghi, R., Bembi, M., Duong, M., Patel, J., Hackmann, A.E. & Ganesh, S. (2020}) 'Use of a Bluetooth tablet-based technology to improve outcomes in lung transplantation: A pilot study'. *AMERICAN JOURNAL OF TRANSPLANTATION}* **20}** (12}), 3649-3657}

Schickler, M., Pryss, R., Schobel, J. & Reichert, M. (2017}) 'Supporting Remote Therapeutic Interventions with Mobile Processes'. In Tata, S. and Mao, Z. (eds.) *2017 IEEE 6TH INTERNATIONAL CONFERENCE ON AI \& MOBILE SERVICES (AIMS)},* Held 345 E 47TH ST, NEW YORK, NY 10017 USA}: IEEE}, 30-37}

Schmidt, B., Wenitong, M., Esterman, A., Hoy, W., Segal, L., Taylor, S., Preece, C., Sticpewich, A. & McDermott, R. (2012) 'Getting better at chronic care in remote communities: study protocol for a

pragmatic cluster randomised controlled of community based management'. *BMC public health [computer file]* **12**, 1017

Schmidt, B., Durao, S., Toews, I., Bavuma, C.M., Hohlfeld, A., Nury, E., Meerpohl, J.J. & Kredo, T. (2020) 'Screening strategies for hypertension'. *Cochrane Database of Systematic Reviews* (5)

Schmitz-Grosz, K. (2021) 'Changes in Medical Processes Due to Digitalization: Examples from Telemedicine'. *Digitalization in Healthcare* , 73

Schoenberg, N., Dunfee, M., Yeager, H., Rutledge, M., Pfammatter, A. & Spring, B. (2021) 'Rural Residents' Perspectives on an mHealth or Personalized Health Coaching Intervention: Qualitative Study With Focus Groups and Key Informant Interviews'. *JMIR Formative Research* **5** (2), e18853

Schoenfeld, M.H., Compton, S.J., Mead, R.H., Weiss, D.N., Sherfesee, L., Englund, J. & Mongeon, L.R. (2004) 'Remote monitoring of implantable cardioverter defibrillators: a prospective analysis'. *Pacing & Clinical Electrophysiology* **27** (6), 757-763

Schofield-Robinson, O., Lewis, S.R., Smith, A.F., McPeake, J. & Alderson, P. (2018) 'Follow-up services for improving long-term outcomes in intensive care unit (ICU) survivors'. *Cochrane Database of Systematic Reviews* (11)

Scholz, M., Haase, R., Schriefer, D., Voigt, I. & Ziemssen, T. (2021) *Electronic Health Interventions in the Case of Multiple Sclerosis: From Theory to Practice.Brain Sci.2021, 11, 180*

Scholz, M., Haase, R., Schriefer, D., Voigt, I. & Ziemssen, T. (2021) 'Electronic health interventions in the case of multiple sclerosis: from theory to practice'. *Brain Sciences* **11** (2), 180

Schuchert, A. (2009) '[Telemedicine in pacemaker therapy and follow-up]'. *Herzschrittmachertherapie und Elektrophysiologie* **20** (4), 164-172

Schwaab, B. (2007) '[Telemedicine in cardiac rehabilitation--a literature review and critical appraisement]'. *Rehabilitation* **46** (5), 276-282

Schwamm, L.H., Chumbler, N., Brown, E., Fonarow, G.C., Berube, D., Nystrom, K., Suter, R., Zavala, M., Polsky, D. & Radhakrishnan, K. (2017) 'Recommendations for the implementation of telehealth in cardiovascular and stroke care: a policy statement from the American Heart Association'. *Circulation* **135** (7), e24-e44

Scoley, G., Gordon, A. & Morrison, S.J. (2019) 'The effect of calf jacket usage on performance, behaviour and physiological responses of group-housed dairy calves.'. *Animal* **13** (12), 2876-2884

Secker-Walker, R., Gnich, W., Platt, S. & Lancaster, T. (2002) 'Community interventions for reducing smoking among adults'. *Cochrane Database of Systematic Reviews* (2)

Seixas, A., Conners, C., Chung, A., Donley, T. & Jean-Louis, G. (2020) 'A Pantheoretical Framework to Optimize Adherence to Healthy Lifestyle Behaviors and Medication Adherence: The Use of Personalized Approaches to Overcome Barriers and Optimize Facilitators to Achieve Adherence'. *JMIR mHealth and uHealth* **8** (6), e16429

Sejdić, E., Godfrey, A., McIlroy, W. & Montero-Odasso, M. (2019) 'Engineering Human Gait and the Potential Role of Wearable'. *Falls and Cognition in Older Persons: Fundamentals, Assessment and Therapeutic Options* , 401

Semenova, Y., Zhunussov, Y., Pivina, L., Abisheva, A., Tinkov, A., Belikhina, T., Skalny, A., Zhanaspayev, M., Bulegenov, T., Glushkova, N., Lipikhina, A., Dauletyarova, M., Zhunussova, T. & Bjorklund, G. (2019) 'Trace element biomonitoring in hair and blood of occupationally unexposed population residing in polluted areas of East Kazakhstan and Pavlodar regions.'. *Journal of Trace Elements in Medicine & Biology* **56**, 31-37

Senanayake, M.G.B. & Sunil, G. 'Journal of Mobile Technology in Medicine'.

Sengupta, P.P. (2013) 'Intelligent platforms for disease assessment: novel approaches in functional echocardiography'. *JACC: Cardiovascular Imaging* **6** (11), 1206-1211

Sen-Gupta, E., Wright, D.E., Caccese, J.W., Wright, J.A.J., Jortberg, E., Bhatkar, V., Ceruolo, M., Ghaffari, R., Clason, D.L., Maynard, J.P. & Combs, A.H. (2019) 'A Pivotal Study to Validate the Performance of a Novel Wearable Sensor and System for Biometric Monitoring in Clinical and Remote Environments.'. *Digital Biomarkers* **3** (1), 1-13

Serrano, J.A. & Holthe, H. (2015) 'Development and trial of ePoint.telemed - An open web-based platform for home monitoring of chronic heart failure patients.'. *Studies in Health Technology & Informatics* **210**, 311-315

Seto, E. (2008) 'Cost comparison between telemonitoring and usual care of heart failure: a systematic review'. *Telemedicine and e-Health* **14** (7), 679-686

Seto, E., Leonard, K.J., Masino, C., Cafazzo, J.A., Barnsley, J. & Ross, H.J. (2010) 'Attitudes of heart failure patients and health care providers towards mobile phone-based remote monitoring'. *Journal of Medical Internet Research* **12** (4), e55-e55

Sezgin, E. & Lin, S. (2019) 'Technology-based interventions, assessments, and solutions for safe driving training for adolescents: rapid review'. *JMIR mHealth and uHealth* **7** (1), e11942

Shah, M., Zimmer, R., Kollefrath, M. & Khandwalla, R. (2020) 'Digital Technologies in Heart Failure Management'. *Current Cardiovascular Risk Reports* **14** (8), 1-8

Shah, N., Ali, Y., Ullah, N. & García-Magariño, I. (2019) 'Internet of Things for Healthcare Using Effects of Mobile Computing: A Systematic Literature Review'. *Wireless Communications & Mobile Computing (Online)* **2019**

Shah, D., Sachdev, H.S., Gera, T., De-Regil, L. & Peña-Rosas, J. (2016) 'Fortification of staple foods with zinc for improving zinc status and other health outcomes in the general population'. *Cochrane Database of Systematic Reviews* (6)

Shah, S.A., Velardo, C., Farmer, A. & Tarassenko, L. (2017}) 'Exacerbations in Chronic Obstructive Pulmonary Disease: Identification and Prediction Using a Digital Health System'. *JOURNAL OF MEDICAL INTERNET RESEARCH}* **19}** (3})

Sharp, L.K., Biggers, A., Perez, R., Henkins, J., Tilton, J. & Gerber, B.S. (2021) 'A Pharmacist and Health Coach–Delivered Mobile Health Intervention for Type 2 Diabetes: Protocol for a Randomized Controlled Crossover Study'. *JMIR research protocols* **10** (3), e17170

Shcherbakova, N. & Desselle, S. (2020) 'Looking Back at US Pharmacy’s Past to Help Discern Its Future'. *Annals of Pharmacotherapy* **54** (9), 907-920

Shey, M.S., Kongnyuy, E.J., Alobwede, S.M. & Wiysonge, C.S. (2013) 'Co-formulated abacavir-lamivudine-zidovudine for initial treatment of HIV infection and AIDS'. *Cochrane Database of Systematic Reviews* (3)

Shimada, M., Akaishi, M., Asakura, K., Baba, A., Iwanaga, S., Asakura, Y., Miyazaki, T., Mitamura, H. & Ogawa, S. (1996) '[Usefulness of the newly developed transtelephonic electrocardiogram and computer-supported response system]'. *Journal of cardiology* **27** (4), 211-217

Shinbane, J.S. & Saxon, L.A. (2016) 'Digital monitoring and care: Virtual medicine'. *Trends in cardiovascular medicine* **26** (8), 722-730

Shirali, G., Erickson, L., Apperson, J., Goggin, K., Williams, D., Reid, K., Bradley-Ewing, A., Tucker, D., Bingler, M., Spertus, J., Rabbitt, L. & Stroup, R. (2016) 'Harnessing Teams and Technology to Improve Outcomes in Infants With Single Ventricle.'. *Circulation.Cardiovascular Quality & Outcomes* **9** (3), 303-311

Shrivastava, S., Trung, T.Q. & Lee, N. (2020) 'Recent progress, challenges, and prospects of fully integrated mobile and wearable point-of-care testing systems for self-testing'. *Chemical Society Reviews* **49** (6), 1812-1866

Shufelt, C., Dzubur, E., Joung, S., Fuller, G., Mouapi, K.N., Van Den Broek, I., Lopez, M., Dhawan, S., Arnold, C.W., Speier, W., Mastali, M., Fu, Q., Van Eyk, J.E., Spiegel, B. & Merz, C.N.B. (2019}) 'A protocol integrating remote patient monitoring patient reported outcomes and cardiovascular biomarkers'. *NPJ DIGITAL MEDICINE}* **2}**

Sibley, L.M., Sipe, T.A. & Barry, D. (2012) 'Traditional birth attendant training for improving health behaviours and pregnancy outcomes'. *Cochrane Database of Systematic Reviews* (8)

Siegel, P.Z., Brackbill, R.M. & Heath, G.W. (1995) 'The epidemiology of walking for exercise: implications for promoting activity among sedentary groups'. *American Journal of Public Health* **85** (5), 706-710

Siegfried, N., Pienaar, D.C., Ataguba, J.E., Volmink, J., Kredo, T., Jere, M. & Parry, C. (2014) 'Restricting or banning alcohol advertising to reduce alcohol consumption in adults and adolescents'. *Cochrane Database of Systematic Reviews* (11)

Signorini, M.G., Lanzola, G., Torti, E., Fanelli, A. & Magenes, G. (2018}) 'Antepartum Fetal Monitoring through a Wearable System and a Mobile Application'. *TECHNOLOGIES}* **6}** (2})

Silva, Francisco Mateus Valente Matos (2020) *Digital platform for psychological assessment supported by sensors and efficiency algorithms*

Simon, P. (2014) 'Medical home telemonitoring of chronically ill patients. 1) lessons learnt from large international studies'. *European research in telemedicine* **3** (2), 85-93

Singh, D.N., Hashmi, M.F. & Sharma, S.K. (2020}) 'Predictive analytics \& modeling for modern health care system for cerebral palsy patients'. *MULTIMEDIA TOOLS AND APPLICATIONS}* **79}** (15-16}), 10285-10307}

Sirichana, W., Patel, M.H., Wang, X., Taylor, M., Barjaktarevic, I., Kleerup, E.C. & Cooper, C.B. (2014) 'Choices of spirometry measures for remote patient monitoring in COPD'. *American journal of respiratory and critical care medicine* **189**

Sjölinder, M. & Nöu, A.A. (2014) 'Indoor and outdoor social alarms: Understanding users' perspectives'. *JMIR mHealth and uHealth* **2** (1), e9

Skobel, E., Knackstedt, C., Martinez-Romero, A., Salvi, D., Vera-Munoz, C., Napp, A., Luprano, J., Bover, R., Glöggler, S. & Bjarnason-Wehrens, B. (2017) 'Internet-based training of coronary artery patients: the Heart Cycle Trial'. *Heart and vessels* **32** (4), 408-418

SLCTR/2016/005, (2016) 'Control of Blood Pressure and Risk Attenuation- Bangladesh, Pakistan & Sri Lanka'. http://www.who.int/trialsearch/Trial2.aspx?TrialID=SLCTR/2016/005

Slemon, K. (2018) *Constructing Deserving Patients: An exploration of self-tracking with high blood pressure using discourse analysis*

Slepian, M.J. (2011) 'The SynCardia temporary total artificial heart-evolving clinical role and future status.'. *US Cardiology* **8** (1), 39-46

Smith, C.A. & Keselman, A. (2020) 'People Engaging with Health Information Technology'. *Consumer Health Informatics: Enabling Digital Health for Everyone* , 91

Smith, S.M., Wallace, E., O'Dowd, T. & Fortin, M. (2021) 'Interventions for improving outcomes in patients with multimorbidity in primary care and community settings'. *Cochrane Database of Systematic Reviews* (1)

Soares-Weiser, K., Bergman, H., Henschke, N., Pitan, F. & Cunliffe, N. (2019) 'Vaccines for preventing rotavirus diarrhoea: vaccines in use'. *Cochrane Database of Systematic Reviews* (10)

Society, A.G., Society, G., Of, A.A. & On Falls Prevention, Orthopaedic Surgeons Panel (2001) 'Guideline for the prevention of falls in older persons'. *Journal of the American Geriatrics Society* **49** (5), 664-672

Søgaard, P., Behrens, S., Konyi, A., Taborsky, M., Christiansen, P.D., Jacobsen, P.K., Faluközy, J., Malczynski, J., Brachmann, J., Samol, A., Kluding, M., Schrader, J., Bloch Thomsen, P. & Jøns, C. (2019) 'Transmission and loss of ECG snapshots: Remote monitoring in implantable cardiac monitors'. *Journal of electrocardiology* **56**, 24-28

Solbiati, M., Costantino, G., Casazza, G., Dipaola, F., Galli, A., Furlan, R., Montano, N. & Sheldon, R. (2016) 'Implantable loop recorder versus conventional diagnostic workup for unexplained recurrent syncope'. *Cochrane Database of Systematic Reviews* (4)

Son, C., Hegde, S., Smith, A., Wang, X. & Sasangohar, F. (2020) 'Effects of COVID-19 on college students' mental health in the United States: Interview survey study'. *Journal of medical internet research* **22** (9), e21279

Soopramanien, A., Jamwal, S. & Thomas, P.W. (2020) 'Digital health rehabilitation can improve access to care in spinal cord injury in the UK: a proposed solution'. *International journal of telerehabilitation* **12** (1), 3

Soto-Martinez, M. (2019) 'Strategies to minimize the effects of air pollution on respiratory health'. *Pediatric pulmonology* **54**, S65-S67

Souza Filho, Erito Marques de, Fernandes, F.d.A., Soares, Celine Lacerda de Abreu, Seixas, F.L., Santos, A.A.S., Gismondi, R.A., Mesquita, E.T. & Mesquita, C.T. (2019) 'Artificial Intelligence in Cardiology: Concepts, Tools and Challenges-"The Horse is the One Who Runs, You Must Be the Jockey"'. *Arquivos Brasileiros de Cardiologia* (AHEAD)

Spanakis, E.G., Santana, S., Tsiknakis, M., Marias, K., Sakkalis, V., Teixeira, A., Janssen, J.H., de Jong, H. & Tziraki, C. (2016) 'Technology-based innovations to foster personalized healthy lifestyles and well-being: a targeted review'. *Journal of medical Internet research* **18** (6), e128

Spangelsteinr, G., Kurgen, J. & Darcy, N. (2014) 'Relationship Between Stem Cell Pluripotency and Physical Activity Levels In Of-fice Workers: Rationale and Study Design for the Stand Up Stem Cells (SUSC) Randomized Trial'. *Int J Stem Cell Res Transplant* **2** (01), 40-51

Sparkes, J., Valaitis, R. & McKibbon, A. (2012) 'A usability study of patients setting up a cardiac event loop recorder and BlackBerry gateway for remote monitoring at home.'. *Telemedicine Journal & E-Health* **18** (6), 484-490

Sparks, K.E., Shaw, D.K., III, J.H. & Quinn, L.M. (1998) 'Cardiovascular complications of outpatient cardiac rehabilitation programs utilizing transtelephonic exercise monitoring'. *Cardiopulmonary Physical Therapy Journal (American Physical Therapy Association, Cardiopulmonary Section)* **9** (4), 3-6

Spencker, S., Coban, N., Koch, L., Schirdewan, A. & Muller, D. (2010) 'Limitations of the past and latest evolutions of home monitoring: arrhythmia electrograms transmitted automatically improve the efficacy of remote monitoring.'. *Aging-Clinical & Experimental Research* **22** (5-6), 459-465

Spencker, S., Coban, N., Koch, L., Schirdewan, A. & Muller, D. (2009) 'Potential role of home monitoring to reduce inappropriate shocks in implantable cardioverter-defibrillator patients due to lead failure.'. *Europace* **11** (4), 483-488

Spinsante, S., Antonicelli, R., Mazzanti, I. & Gambi, E. (2012) 'Technological approaches to remote monitoring of elderly people in cardiology: a usability perspective.'. *International Journal of Telemedicine & Applications* **2012**, 104561

Spinsante, S., Ricciuti, M. & Scalise, L. (2018}) 'Contactless Measurement of Heart Rate for Exergames Applications'. In Anonymous (eds.) *2018 IEEE INTERNATIONAL SYMPOSIUM ON MEDICAL MEASUREMENTS AND APPLICATIONS (MEMEA)},* Held 345 E 47TH ST, NEW YORK, NY 10017 USA}: IEEE}, 890-895}

SpruijtMetz D., Emken A., Li M., Tatte G., Lee S., Vathsangam H., Sukhatme G., Mitra U., Annavaram M. & Narayanan, S. (2010) 'Mobile health technologies to better understand, prevent and treat childhood obesity in latino youth: A pilot field trial of KNOWME NETWORKS.'. *Obesity.Conference: 28th Annual Scientific Meeting of the Obesity Society, OBESITY 2010.San Diego, CA United States.Conference Publication: (var.pagings)* **18** (SUPPL. 2), S175

Squires, R.W., Miller, T.D., Harn, T., Micheels, T.A. & Palma, T.A. (1991) 'Transtelephonic electrocardiographic monitoring of cardiac rehabilitation exercise sessions in coronary artery disease.'. *American Journal of Cardiology* **67** (11), 962-964

St Fleur, R.G., St George, S.M., Leite, R., Kobayashi, M., Agosto, Y. & Jake-Schoffman, D.E. (2021) 'Use of Fitbit Devices in Physical Activity Intervention Studies Across the Life Course: Narrative Review'. *JMIR mHealth and uHealth* **9** (5), e23411

Stammer, S.E. (2018) 'Reducing Home Health COPD-Related 30-Day Hospital Readmissions Using Telehealth Technology'.

Starner, T. & Paradiso, J.A. (2004) 'Human generated power for mobile electronics'. *Low-power electronics design* **45**, 1-35

Stauss, M., Floyd, L., Becker, S., Ponnusamy, A. & Woywodt, A. (2021) 'Opportunities in the cloud or pie in the sky? Current status and future perspectives of telemedicine in nephrology'. *Clinical kidney journal* **14** (2), 492-506

Stavropoulos, T.G., Papastergiou, A., Mpaltadoros, L., Nikolopoulos, S. & Kompatsiaris, I. (2020) 'IoT Wearable Sensors and Devices in Elderly Care: A Literature Review'. *Sensors* **20** (10)

Steed, L., Sohanpal, R., Todd, A., Madurasinghe, V.W., Rivas, C., Edwards, E.A., Summerbell, C.D., Taylor, S. & Walton, R.T. (2019) 'Community pharmacy interventions for health promotion: effects on professional practice and health outcomes'. *Cochrane Database of Systematic Reviews* (12)

Stein, N. & Brooks, K. (2017) 'A fully automated conversational artificial intelligence for weight loss: longitudinal observational study among overweight and obese adults'. *JMIR diabetes* **2** (2), e8590

Steinberg, J.S., Varma, N., Cygankiewicz, I., Aziz, P., Balsam, P., Baranchuk, A., Cantillon, D.J., Dilaveris, P., Dubner, S.J. & El-Sherif, N. (2017) '2017 ISHNE-HRS expert consensus statement on ambulatory ECG and external cardiac monitoring/telemetry'. *Heart Rhythm* **14** (7), e55-e96

Steinkamp, J.M., Goldblatt, N., Borodovsky, J.T., LaVertu, A., Kronish, I.M., Marsch, L.A. & Schuman-Olivier, Z. (2019) 'Technological interventions for medication adherence in adult mental health and substance use disorders: a systematic review'. *JMIR mental health* **6** (3), e12493

Stengel, D., Leisterer, J., Ferrada, P., Ekkernkamp, A., Mutze, S. & Hoenning, A. (2018) 'Point-of-care ultrasonography for diagnosing thoracoabdominal injuries in patients with blunt trauma'. *Cochrane Database of Systematic Reviews* (12)

Stetson, B.A. & Dubbert, P.M. (2019) 'Cognitive and Behavioral Approaches to Enhancing Physical Activity Participation and Decreasing Sedentary Behavior'. *Lifestyle Medicine* , 253

Steventon, A., Ariti, C., Fisher, E. & Bardsley, M. (2016) 'Effect of telehealth on hospital utilisation and mortality in routine clinical practice: a matched control cohort study in an early adopter site.'. *BMJ Open* **6** (2), e009221

Stiles-Shields, C., Potthoff, L.M., Bounds, D.T., Burns, M.T., Draxler, J.M., Otwell, C.H., Wolodiger, E.D., Westrick, J. & Karnik, N.S. (2020) 'Harnessing Phones to Target Pediatric Populations with Socially Complex Needs: Systematic Review'. *JMIR pediatrics and parenting* **3** (2), e19269

Storebø, O., Stoffers-Winterling, J., Völlm, B., Kongerslev, M.T., Mattivi, J.T., Jørgensen, M., Faltinsen, E., Todorovac, A., Sales, C.P. & Callesen, H.E. (2020) 'Psychological therapies for people with borderline personality disorder'. *Cochrane Database of Systematic Reviews* (5)

Stuckey M.I., RussellMinda E., Fulkerson R., Read E., Munoz C. & Petrella, R.J. (2010) 'Remote health monitoring technologies and physical activity to modify markers of metabolic syndrome.'. *Diabetes* **Conference: 70th Scientific Sessions of the American Diabetes Association. Orlando, FL United States. Conference Publication** (var.pagings), ate of Pubaton: 2010

Stuckey, M.I., Shapiro, S., Gill, D.P. & Petrella, R.J. (2013) 'A lifestyle intervention supported by mobile health technologies to improve the cardiometabolic risk profile of individuals at risk for cardiovascular disease and type 2 diabetes: study rationale and protocol.'. *BMC Public Health* **13**, 1051

Stylianou, A., McCormack, H. & Kokmotou, R. (2013) 'applying mHealth solutions'. *Eur J ePractice* **20**, 2-4

Su, D., Michaud, T.L., Estabrooks, P., Schwab, R.J., Eiland, L.A., Hansen, G., DeVany, M., Zhang, D., Li, Y., Pagan, J.A. & Siahpush, M. (2019}) 'Diabetes Management Through Remote Patient Monitoring: The Importance of Patient Activation and Engagement with the Technology'. *TELEMEDICINE AND E-HEALTH}* **25}** (10}), 952-959}

Su, F. & Guo, X. (2002) '[Clinical application of the expert type terminal of remote electronic fetal heart rate home monitoring system]'. *Chung-Hua Fu Chan Ko Tsa Chih [Chinese Journal of Obstetrics & Gynecology]* **37** (8), 459-461

Suchdev, P.S., Peña-Rosas, J. & De-Regil, L. (2015) 'Multiple micronutrient powders for home (point-of-use) fortification of foods in pregnant women'. *Cochrane Database of Systematic Reviews* (6)

Suciu, V., Suciu, C., Nicoara, S.D. & Perju-Dumbrava, L. (2020) 'EMPOWERING TELEMEDICINE– ESSENTIAL FOR NEUROLOGIC AND OPHTHALMOLOGIC CONDITIONS IN THE CONTEXT OF A PANDEMIC AND BEYOND.'. *Romanian JouRnal of neuRology* **19** (3)

Sudfeld, C.R., Bliznashka, L., Ashery, G., Yousafzai, A.K. & Masanja, H. (2019) 'Effect of a community health worker delivered health, nutrition and responsive stimulation package and conditional cash transfers on child development and growth in rural Tanzania: protocol for a cluster-randomized trial'. *BMC public health [computer file]* **19** (1), 641

Sugita, K., Suzuki, N., Nogaki, F., Washida, N. & Urano, T. (2021) '[An 89-year-old man who underwent percutaneous peritoneal dialysis catheter placement and had peritoneal dialysis introduced]'. *Nippon Ronen Igakkai Zasshi - Japanese Journal of Geriatrics* **58** (2), 303-308

Sun, J., Guo, Y., Wang, X. & Zeng, Q. (2016) 'mHealth For Aging China: Opportunities and Challenges'. *Aging and disease* **7** (1), 53-67

Sun, S., Folarin, A.A., Ranjan, Y., Rashid, Z., Conde, P., Stewart, C., Cummins, N., Matcham, F., Costa, G.D., Simblett, S., Leocani, L., Lamers, F., Sørensen, P.S., Buron, M., Zabalza, A., Pérez, A.,Isabel Guerrero, Penninx, B.W.J.H., Siddi, S., Haro, J.M. & Myin-Germeys, I. (2020) 'Using Smartphones and Wearable Devices to Monitor Behavioral Changes During COVID-19'. *Journal of Medical Internet Research* **22** (9), N.PAG-N.PAG

Sun, Y., Papin, C., Azorin-Peris, V., Kalawsky, R., Greenwald, S. & Hu, S. (2011}) 'Comparison of scientific CMOS camera and webcam for monitoring cardiac pulse after exercise'. In Tescher, A. (eds.) *APPLICATIONS OF DIGITAL IMAGE PROCESSING XXXIV},* Held 1000 20TH ST, PO BOX 10, BELLINGHAM, WA 98227-0010 USA}: SPIE-INT SOC OPTICAL ENGINEERING}

Sundararaman, L.V., Edwards, R.R., Ross, E.L. & Jamison, R.N. (2017) 'Integration of Mobile Health Technology in the Treatment of Chronic Pain: A Critical Review'. *Regional anesthesia and pain medicine* **42** (4), 488-498

SUPPORT-HF 2 Investigators and Committees. Electronic address: kazem.rahimi@georgeinstitute.ox.ac.uk (2019) 'Home monitoring with IT-supported specialist management versus home monitoring alone in patients with heart failure: Design and baseline results of the SUPPORT-HF 2 randomized trial.'. *American Heart Journal* **208**, 55-64

Suresh Kumar, S., Dashtipour, K., Abbasi, Q.H., Imran, M.A. & Ahmad, W. (2021) 'A review on wearable and contactless sensing for COVID-19 with policy challenges'. *Frontiers in Communications and Networks*

Suter, P., Suter, W.N. & Johnston, D. (2011) 'Theory-Based Telehealth and Patient Empowerment'. *Population Health Management* **14** (2), 87-92

Szankin, M., Kwasniewska, A., Sirlapu, T., Wang, M., Ruminski, J., Nicolas, R. & Bartscherer, M. (2018) 'Long Distance Vital Signs Monitoring with Person Identification for Smart Home Solutions.'. *Annual International Conference Of The IEEE Engineering In Medicine And Biology Society* **2018**, 1558-1561

Tacchino, A., Pedullà, L., Bonzano, L., Vassallo, C., Battaglia, M.A., Mancardi, G., Bove, M. & Brichetto, G. (2015) 'A New App for At-Home Cognitive Training: Description and Pilot Testing on Patients with Multiple Sclerosis'. *JMIR mHealth and uHealth* **3** (3), e85

Tachakra, S., Wang, X., Istepanian, R.S. & Song, Y. (2003) 'Mobile e-health: the unwired evolution of telemedicine'. *Telemedicine Journal and E-health* **9** (3), 247-257

Takemoto, M., Manini, T.M., Rosenberg, D.E., Lazar, A., Zlatar, Z.Z., Das, S.K. & Kerr, J. (2018) 'Diet and activity assessments and interventions using technology in older adults'. *American Journal of Preventive Medicine* **55** (4), e105-e115

Tambo, E., Xia, S., Xin-Yu, F. & Xiao-Nong, Z. (2018) 'Digital Surveillance and Communication Strat-effigies to Infectious Diseases of Poverty Control and Elimination in Africa'. *J Infect Dis Epidemiol* **4**, 056

Tamura, T., Masuda, Y., Sekimoto, M., Higashi, Y. & Fujimoto, T. (2004) 'A mobile-phone based telecare system for the elderly.'. *Conference Proceedings: ...Annual International Conference of the IEEE Engineering in Medicine & Biology Society* **2004**, 3260-3263

Tan, K. & Lai, N.M. (2012) 'Telemedicine for the support of parents of high-risk newborn infants'. *Cochrane Database of Systematic Reviews* (6)

Tang, J., Abraham, C., Stamp, E. & Greaves, C. (2015) 'How can weight-loss app designers' best engage and support users? A qualitative investigation'. *British journal of health psychology* **20** (1), 151-171

Tang, J. & Chen, S. (2014) 'Early event detection using a home monitoring system for patients with cardiac pacemakers.'. *Aging-Clinical & Experimental Research* **26** (2), 131-135

Tarantini, L., Navazio, A., Cioffi, G., Turiano, G., Colivicchi, F. & Gabrielli, D. (2020) '[Being a cardiologist at the time of SARS-COVID-19: is it time to reconsider our way of working?]'. *Giornale italiano di cardiologia* **21** (5), 354-357

Tarraf, R.C., Suter, E., Arain, M., Birney, A., Boakye, O., Boulanger, P. & Sadowski, C.A. (2019) 'Using integrated technology to create quality care for older adults: a feasibility study'. *Informatics for Health and Social Care* **44** (3), 246-261

Taylor, R.M., Haslam, R.L., Burrows, T.L., Duncanson, K.R., Ashton, L.M., Rollo, M.E., Shrewsbury, V.A., Schumacher, T.L. & Collins, C.E. (2019) 'Issues in measuring and interpreting diet and its contribution to obesity'. *Current obesity reports* **8** (2), 53-65

Taylor, G., Dalili, M.N., Semwal, M., Civljak, M., Sheikh, A. & Car, J. (2017) 'Internet-based interventions for smoking cessation'. *Cochrane Database of Systematic Reviews* (9)

Taylor, J.K., Ndiaye, H., Daniels, M., Ahmed, F. & Triage-HF Plus investigators (2021) 'Lockdown, slow down: impact of the COVID-19 pandemic on physical activity-an observational study.'. *Open Heart* **8** (1), 06

Taylor, K.W., Liggins, R., Mendler, P. & Schuh, F. (1976) 'ECG telephone transmission for monitoring pacemakers and cardiac arrhythmias.'. *Medical progress through technology* **4** (3), 133-138

TEC4Home Healthcare Innovation Community (2016) 'Supporting Heart Failure Patient Transitions From Acute to Community Care With Home Telemonitoring Technology: A Protocol for a Provincial Randomized Controlled Trial (TEC4Home).'. *JMIR Research Protocols* **5** (4), e198

Teferra, M.N., Ramos, J.S., Kourbelis, C., Newman, P., Fleury, A., Hobbs, D., Reynolds, K.J. & Clark, R.A. (2019) 'Electronic textile-based electrocardiogram monitoring in cardiac patients: a scoping review.'. *JBI Database Of Systematic Reviews And Implementation Reports* **17** (10), 1958-1998

Tekeste, T., Saleh, H., Mohammad, B., Khandoker, A., Jelinek, H. & Ismail, M. (2018) 'A nanowatt real-time cardiac autonomic neuropathy detector'. *IEEE transactions on biomedical circuits and systems* **12** (4), 739-750

Tersalvi, G., Winterton, D., Cioffi, G.M., Ghidini, S., Roberto, M., Biasco, L., Pedrazzini, G., Dauw, J., Ameri, P. & Vicenzi, M. (2020) 'Telemedicine in Heart Failure During COVID-19: A Step Into the Future'. *Frontiers in Cardiovascular Medicine* **7**, 612818

Thabrew, H., Stasiak, K., Hetrick, S.E., Wong, S., Huss, J.H. & Merry, S.N. (2018) 'E-Health interventions for anxiety and depression in children and adolescents with long-term physical conditions'. *Cochrane Database of Systematic Reviews* (8)

Thieme, H., Morkisch, N., Mehrholz, J., Pohl, M., Behrens, J., Borgetto, B. & Dohle, C. (2018) 'Mirror therapy for improving motor function after stroke'. *Cochrane Database of Systematic Reviews* (7)

Thomas, E.R.E. (2019) *Improving cardiac rehabilitation in Victoria*

Thomas, S.M., Delanni, E., Christophe, B. & Connolly, E.S. (2021) 'Systematic review of novel technology-based interventions for ischemic stroke'. *Neurological Sciences* , 1-13

Thomason, T.R., Hawkins, S.Y., Perkins, K.E., Hamilton, E. & Nelson, B. (2015) 'Home telehealth and hospital readmissions: a retrospective OASIS-C data analysis.'. *Home Healthcare Now* **33** (1), 20-26

Thong, T. (2015}) 'Remote Monitoring of Cardiac Rhythm Management Devices in Vietnam The Role of the Biomedical Engineer'. In VanToi, V. and Phuong, T. (eds.) *5TH INTERNATIONAL CONFERENCE ON BIOMEDICAL ENGINEERING IN VIETNAM},* Held 233 SPRING STREET, NEW YORK, NY 10013, UNITED STATES}: SPRINGER}, 79-82}

Thorogood, M., Connor, M., Tollman, S., Hundt, G.L., Fowkes, G. & Marsh, J. (2007}) 'A cross-sectional study of vascular risk factors in a rural South African population: data from the Southern African Stroke Prevention Initiative (SASPI)'. *BMC PUBLIC HEALTH}* **7}**

Thorup, C.B. (2016) 'Step counter use and self-determined motivation for walking in a cardiac telerehabilitation program: A mixed method study'.

Tieu, J., McPhee, A.J., Crowther, C.A., Middleton, P. & Shepherd, E. (2017) 'Screening for gestational diabetes mellitus based on different risk profiles and settings for improving maternal and infant health'. *Cochrane Database of Systematic Reviews* (8)

Tighe, S.A., Ball, K., Kensing, F., Kayser, L., Rawstorn, J. & Maddison, R. (2020) 'Toward a Digital Platform for the Self-Management of Noncommunicable Disease: Systematic Review of Platform-Like Interventions'. *Journal of medical Internet research* **22** (10), e16774

Tiihonen, P., Hukkanen, T., Tuomilehto, H., Mervaala, E. & Toyras, J. (2009) 'Evaluation of a novel ambulatory device for screening of sleep apnea.'. *Telemedicine Journal & E-Health* **15** (3), 283-289

Till, S.R., Everetts, D. & Haas, D.M. (2015) 'Incentives for increasing prenatal care use by women in order to improve maternal and neonatal outcomes'. *Cochrane Database of Systematic Reviews* (12)

Timmermans, S. & Kaufman, R. (2020) 'Technologies and Health Inequities'. *Annual Review of Sociology* **46**, 583-602

Timmons, B.W., LeBlanc, A.G., Carson, V., Connor Gorber, S., Dillman, C., Janssen, I., Kho, M.E., Spence, J.C., Stearns, J.A. & Tremblay, M.S. (2012) 'Systematic review of physical activity and health in the early years (aged 0–4 years)'. *Applied Physiology, Nutrition, and Metabolism* **37** (4), 773-792

Tobe, S.W., Wentworth, J., Ironstand, L., Hartman, S., Hoppe, J., Whiting, J., Kennedy, J., McAllister, C., Kiss, A., Perkins, N., Vincent, L., Pylypchuk, G. & Lewanczuk, R.Z. (2009}) 'DreamTel; Diabetes risk evaluation and management tele-monitoring study protocol'. *BMC ENDOCRINE DISORDERS}* **9}**

Tobon V, D.P., Falk, T.H. & Maier, M. (2016) 'MS-QI: A Modulation Spectrum-Based ECG Quality Index for Telehealth Applications.'. *IEEE Transactions on Biomedical Engineering* **63** (8), 1613-1622

Tomines, A. (2019) 'Pediatric Telehealth: Approaches by Specialty and Implications for General Pediatric Care'. *Advances in Pediatrics* **66**, 55-85

Torbjørnsen, A., Ribu, L., Rønnevig, M., Grøttland, A. & Helseth, S. (2019) 'Users' acceptability of a mobile application for persons with type 2 diabetes: a qualitative study'. *BMC health services research* **19** (1), 1-14

Tremblay, M.S., Colley, R.C., Saunders, T.J., Healy, G.N. & Owen, N. (2010) 'Physiological and health implications of a sedentary lifestyle'. *Applied physiology, nutrition, and metabolism* **35** (6), 725-740

Triantafyllidis, A., Velardo, C., Chantler, T., Shah, S.A., Paton, C., Khorshidi, R., Tarassenko, L. & Rahimi, K. (2015) 'A personalised mobile-based home monitoring system for heart failure: The SUPPORT-HF Study'. *International journal of medical informatics* **84** (10), 743-753

Triberti, S. & Barello, S. (2016) 'The quest for engaging AmI: Patient engagement and experience design tools to promote effective assisted living'. *Journal of Biomedical Informatics* **63**, 150-156

Tsukinoki R. & Murakami, Y. (2013) 'Non-communicable disease epidemic: epidemiology in action (EuroEpi 2013 and NordicEpi 2013): Aarhus, Denmark from 11 August to 14 August 2013.'. *European journal of epidemiology* **28** (1), 1-270

Tudor-Locke, C., Craig, C.L., Thyfault, J.P. & Spence, J.C. (2013) 'A step-defined sedentary lifestyle index:< 5000 steps/day'. *Applied physiology, nutrition, and metabolism* **38** (2), 100-114

Tunçalp, Ö., Hofmeyr, G.J. & Gülmezoglu, A. (2012) 'Prostaglandins for preventing postpartum haemorrhage'. *Cochrane Database of Systematic Reviews* (8)

Tura, A., Badanai, M., Longo, D. & Quareni, L. (2004) 'A multi-functional, portable device with wireless transmission for home monitoring of children with a learning disability.'. *Journal of Telemedicine & Telecare* **10** (5), 298-302

Turini, G., Condino, S., Viglialoro, R., Carbone, M. & Gesi, M. 'Digital Commons@ Kettering Universit y'.

Turini, G., Condino, S., Viglialoro, R., Carbone, M., Gesi, M. & Ferrari, V. (2019) 'Review of the Augmented Reality Systems for Shoulder Rehabilitation'. *Information* **10** (5)

Turley, R., Saith, R., Bhan, N., Rehfuess, E. & Carter, B. (2013) 'Slum upgrading strategies involving physical environment and infrastructure interventions and their effects on health and socio-economic outcomes'. *Cochrane Database of Systematic Reviews* (1)

Turrisi, T.B., Bittel, K.M., West, A.B., Hojjatinia, S., Hojjatinia, S., Mama, S.K., Lagoa, C.M. & Conroy, D.E. (2021) 'Seasons, weather, and device-measured movement behaviors: a scoping review from 2006 to 2020'. *International Journal of Behavioral Nutrition and Physical Activity* **18** (1), 1-26

Tusting, L.S., Thwing, J., Sinclair, D., Fillinger, U., Gimnig, J., Bonner, K.E., Bottomley, C. & Lindsay, S.W. (2013) 'Mosquito larval source management for controlling malaria'. *Cochrane Database of Systematic Reviews* (8)

Uchiyama, K., Washida, N., Yube, N., Kasai, T., Shinozuka, K., Morimoto, K., Hishikawa, A., Inoue, H., Urai, H., Hagiwara, A., Fujii, K., Wakino, S., Deenitchina, S. & Itoh, H. (2018}) 'The impact of a remote monitoring system of healthcare resource consumption in patients on automated peritoneal dialysis (APD): A simulation study'. *CLINICAL NEPHROLOGY}* **90}** (5}), 334-340}

Ueafuea, K., Boonnag, C., Sudhawiyangkul, T., Leelaarporn, P., Gulistan, A., Chen, W., Mukhopadhyay, S.C., Wilaiprasitporn, T. & Piyayotai, S. (2020) 'Potential Applications of Mobile and Wearable Devices for Psychological Support During the COVID-19 Pandemic: A Review'. *IEEE Sensors Journal*

Uniyal, D. & Raychoudhury, V. (2014) 'Pervasive healthcare-a comprehensive survey of tools and techniques'. *arXiv preprint arXiv:1411.1821*

Ussher, M.H., Faulkner, G., Angus, K., Hartmann-Boyce, J. & Taylor, A.H. (2019) 'Exercise interventions for smoking cessation'. *Cochrane Database of Systematic Reviews* (10)

Valdivia, S., Blanco, R., Uribe-Quevedo, A., Penuela, L., Rojas, D. & Kapralos, B. (2018) 'Development and evaluation of two posture-tracking user interfaces for occupational health care'. *Advances in Mechanical Engineering* **10** (6), 1687814018769489

Van den Broecke, S., Jobard, O., Montalescot, G., Bruyneel, M., Ninane, V., Arnulf, I., Similowski, T. & Attali, V. (2014}) 'Very early screening for sleep-disordered breathing in acute coronary syndrome in patients without acute heart failure'. *SLEEP MEDICINE}* **15}** (12}), 1539-1546}

van der Laan, N. (2019) 'I would use a fitness tracker that fulfils my needs!'.

Van der Velde, E.T., Atsma, D.E., Foeken, H., Witteman, T.A. & Hoekstra, W.H.G.J. (2013) 'Remote monitoring of patients with implanted devices: data exchange and integration.'. *European Journal of Preventive Cardiology* **20** (2 Suppl), 8-12

van Ginneken, N., Tharyan, P., Lewin, S., Rao, G.N., Meera, S.M., Pian, J., Chandrashekar, S. & Patel, V. (2013) 'Non-specialist health worker interventions for the care of mental, neurological and substance-abuse disorders in low- and middle-income countries'. *Cochrane Database of Systematic Reviews* (11)

Van Ommen, B., Wopereis, S., van Empelen, P., van Keulen, H.M., Otten, W., Kasteleyn, M., Molema, J.J., de Hoogh, I.M., Chavannes, N.H. & Numans, M.E. (2018) 'From diabetes care to diabetes cure—the integration of systems biology, eHealth, and behavioral change'. *Frontiers in endocrinology* **8**, 381

Van Rooij, T.W. (2015) 'eHealth and mHealth Pipelines for Clinical Decision Support to Improve Medication Selection and Safety'.

Vanoli, E., D'Elia, E., La Rovere, M.T. & Gronda, E. (2016}) 'Remote heart function monitoring: role of the CardioMEMS HF System'. *JOURNAL OF CARDIOVASCULAR MEDICINE}* **17}** (7}), 518-523}

Vaona, A., Banzi, R., Kwag, K.H., Rigon, G., Cereda, D., Pecoraro, V., Tramacere, I. & Moja, L. (2018) 'E-learning for health professionals'. *Cochrane Database of Systematic Reviews* (1)

Vaona, A., Pappas, Y., Grewal, R.S., Ajaz, M., Majeed, A. & Car, J. (2017) 'Training interventions for improving telephone consultation skills in clinicians'. *Cochrane Database of Systematic Reviews* (1)

Vargas, D.A.B., Castiblanco, K.A.D. & Pico, L.E.A. (2018) 'Current status of technology and telemedicine services in Colombia'. *International Journal of Applied Engineering Research* **13** (20), 14517-14530

Vargiu, E., Fernández, J.M., Gonzales-Gonzales, M., Morales-Garzón, J.M., Prunera-Moreda, K. & Miralles, F. (2019) 'A self-management system for complex chronic patients'. *International Journal of Integrated Care (IJIC)* **19**, 1-2

Varma, N., Cygankiewicz, I., Turakhia, M.P., Heidbuchel, H., Hu, Y., Chen, L.Y., Couderc, J., Cronin, E.M., Estep, J.D. & Grieten, L. (2021) '2021 ISHNE/HRS/EHRA/APHRS expert collaborative statement on mHealth in arrhythmia management: digital medical tools for heart rhythm professionals: from the International Society for Holter and Noninvasive Electrocardiology/Heart Rhythm Society/European Heart Rhythm Association/Asia-Pacific Heart Rhythm Society'. *Circulation: Arrhythmia and Electrophysiology* **14** (2), e009204

Varma, N. (2007) 'Rationale and design of a prospective study of the efficacy of a remote monitoring system used in implantable cardioverter defibrillator follow-up: the Lumos-T Reduces Routine Office Device Follow-Up Study (TRUST) study'. *American Heart Journal* **154** (6), 1029-1034

Varma, N. & Michalski, J. (2014) 'Prolonged remote monitoring without in-person evaluation in advanced heart failure patients: is there a risk?'. *Journal of cardiac failure.* **20** (8), S67

Varma, N., Michalski, J., Epstein, A.E. & Schweikert, R. (2010) 'Automatic remote monitoring of implantable cardioverter-defibrillator lead and generator performance: the Lumos-T Safely RedUceS RouTine Office Device Follow-Up (TRUST) trial'. *Circulation.Arrhythmia and electrophysiology* **3** (5), 428-436

Varma, N. (2013) 'Automatic remote home monitoring of implantable cardioverter defibrillator lead and generator function: a system that tests itself everyday.'. *Europace* **15** (Suppl 1), 26-31

Varma, N. (2009) 'Therapeutic implications of automatic home monitoring of implantable cardiac devices.'. *Current Treatment Options in Cardiovascular Medicine* **11** (5), 366-372

Varma, N., Epstein, A.E., Irimpen, A., Schweikert, R., Love, C. & TRUST Investigators (2010) 'Efficacy and safety of automatic remote monitoring for implantable cardioverter-defibrillator follow-up: the Lumos-T Safely Reduces Routine Office Device Follow-up (TRUST) trial.'. *Circulation* **122** (4), 325-332

Varma, N., Epstein, A.E., Schweikert, R., Michalski, J., Love, C.J. & TRUST Investigators (2016) 'Role of Automatic Wireless Remote Monitoring Immediately Following ICD Implant: The Lumos-T Reduces Routine Office Device Follow-Up Study (TRUST) Trial.'. *Journal of cardiovascular electrophysiology* **27** (3), 321-326

Varma, N. & Johnson, M.A. (2009) 'Prevalence of cancelled shock therapy and relationship to shock delivery in recipients of implantable cardioverter-defibrillators assessed by remote monitoring.'. *Pacing & Clinical Electrophysiology* **32** (Suppl 1), S42-6

Varma, N., Love, C.J., Schweikert, R., Moll, P., Michalski, J. & Epstein, A.E. (2018) 'Automatic remote monitoring utilizing daily transmissions: transmission reliability and implantable cardioverter defibrillator battery longevity in the TRUST trial'. *EP: Europace* **20** (4), 622-628

Varma, N., Michalski, J., Stambler, B., Pavri, B.B. & TRUST Investigators (2014) 'Superiority of automatic remote monitoring compared with in-person evaluation for scheduled ICD follow-up in the TRUST trial - testing execution of the recommendations.'. *European heart journal* **35** (20), 1345-1352

Varma, N., Pavri, B.B., Stambler, B. & Michalski, J. (2013) 'Same-day discovery of implantable cardioverter defibrillator dysfunction in the TRUST remote monitoring trial: influence of contrasting messaging systems'. *EP: Europace* **15** (5), 697-703

Varma, N. & Ricci, R.P. (2013) 'Telemedicine and cardiac implants: what is the benefit?'. *European heart journal* **34** (25), 1885-1895

Varnfield M., Karunanithi M.K., Sarela A., Garcia E., Fairfull A., Oldenburg B.F. & Walters, D.L. (2011) 'Uptake of a technology-assisted home-care cardiac rehabilitation program.'. *Medical Journal of Australia* **194** (4), S15-S19

Vasan, R.S. & Benjamin, E.J. (2016) 'The future of cardiovascular epidemiology'. *Circulation* **133** (25), 2626-2633

Vassli, L.T. (2016) *A Study of ICT Acceptance Among Seniors with Fall Risk Assessment Technologies as a Case*

Veenis J.F., Radhoe S.P., Hooijmans P. & Brugts, J.J. (2021) 'Remote Monitoring in Chronic Heart Failure Patients: Is Non-Invasive Remote Monitoring the Way to Go?.'. *Sensors (Basel, Switzerland)* **21** (3) (pagination), ate of Pubaton: 28 Jan 2021

Veenis, J.F. & Brugts, J.J. (2020) 'Remote monitoring for better management of LVAD patients: the potential benefits of CardioMEMS'. *General Thoracic & Cardiovascular Surgery* **68** (3), 209-218

Veenis, J.F., Radhoe, S.P., Hooijmans, P. & Brugts, J.J. (2021) 'Remote Monitoring in Chronic Heart Failure Patients: Is Non-Invasive Remote Monitoring the Way to Go?'. *Sensors* **21** (3)

Vegesna, A., Tran, M., Angelaccio, M. & Arcona, S. (2017}) 'Remote Patient Monitoring via Non-Invasive Digital Technologies: A Systematic Review'. *TELEMEDICINE AND E-HEALTH}* **23}** (1}), 3-17}

Venter, A., Burns, R., Hefford, M. & Ehrenberg, N. (2012) 'Results of a telehealth-enabled chronic care management service to support people with long-term conditions at home'. *Journal of telemedicine and telecare* **18** (3), 172-175

Verbraecken, J. (2016) 'Telemedicine Applications in Sleep Disordered Breathing: Thinking Out of the Box'. *Sleep medicine clinics* **11** (4), 445-459

Vergara, P., Solimene, F., D'Onofrio, A., Pisano, E.C., Zanotto, G., Pignalberi, C., Iacopino, S., Maglia, G., Della Bella, P., Calvi, V., Curnis, A., Senatore, G., Biffi, M., Capucci, A., Parisi, Q., Quartieri, F., Caravati, F., Giammaria, M., Marini, M., Rapacciuolo, A., Manzo, M., Giacopelli, D., Gargaro,

A. & Ricci, R.P. (2019) 'Are Atrial High-Rate Episodes Associated With Increased Risk of Ventricular Arrhythmias and Mortality?.'. *JACC.Clinical Electrophysiology* **5** (10), 1197-1208

Vieira, E.R., Richard, L. & da Silva, R.A. (2020) *Perspectives on Research and Health Practice in Physical and Occupational Therapy in Geriatrics during and Post COVID-19*

Viglialoro, R.M., Condino, S., Turini, G., Carbone, M., Ferrari, V. & Gesi, M. (2019) 'Review of the augmented reality systems for shoulder rehabilitation'. *Information* **10** (5), 154

Visco, V., Junior Ferruzzi, G., Nicastro, F., Virtuoso, N., Carrizzo, A., Galasso, G., Vecchione, C. & Ciccarelli, M. (2021) 'Artificial Intelligence as a Business Partner in Cardiovascular Precision Medicine: An Emerging Approach for Disease Detection and Treatment Optimization'. *Current medicinal chemistry*

Visser, J., McLachlan, M.H., Maayan, N. & Garner, P. (2018) 'Community-based supplementary feeding for food insecure, vulnerable and malnourished populations – an overview of systematic reviews'. *Cochrane Database of Systematic Reviews* (11)

Vitacca, M., Guerra, A., Assoni, G., Pizzocaro, P., Marchina, L., Scalvini, S. & Balbi, B. (2007) 'Weaning from mechanical ventilation followed at home with the aid of a telemedicine program.'. *Telemedicine Journal & E-Health* **13** (4), 445-449

Vitalari, N.P. (2016) 'Prospects for the future of the us healthcare industry: a speculative analysis'. *American Journal of Medical Research* **3** (2), 7-52

Voeller, H., Bindl, D., Nagels, K., Hofmann, R., Vettorazzi, E., Wegscheider, K., Fleck, E. & Nagel, E. (2017) 'Remote telemonitoring in chronic heart failure does not reduce healthcare cost but improves quality of life: endpoints of the cardiobbeat trial'. *Journal of the American College of Cardiology* **69** (11), 672-

Volterrani, M. & Sposato, B. (2019) 'Remote monitoring and telemedicine.'. *European Heart Journal Supplements* **21** (Suppl M), M54-M56

Von Ah, D., Brown, C.G., Brown, S.J., Bryant, A.L., Davies, M., Dodd, M., Ferrell, B., Hammer, M., Knobf, M.T. & Knoop, T.J. (2019) 'Research agenda of the oncology nursing society: 2019–2022'. In Anonymous (eds.) *Oncology nursing forum,* Held Oncology Nursing Society, 654-669

von Philipsborn, P., Stratil, J.M., Burns, J., Busert, L.K., Pfadenhauer, L.M., Polus, S., Holzapfel, C., Hauner, H. & Rehfuess, E. (2019) 'Environmental interventions to reduce the consumption of sugar-sweetened beverages and their effects on health'. *Cochrane Database of Systematic Reviews* (6)

Vu, A., Rutherford, S. & Phung, D. (2019) 'Heat Health Prevention Measures and Adaptation in Older Populations—A Systematic Review'. *International journal of environmental research and public health* **16** (22), 4370

Wac, K., Hausheer, D., Fiedler, M. & Bonato, P. (2012) 'Future Internet for eHealth'.

Wadden, T.A., Tronieri, J.S. & Butryn, M.L. (2020) 'Lifestyle modification approaches for the treatment of obesity in adults.'. *American Psychologist* **75** (2), 235

Wahidi, N. & Lerner, A.J. (2019) 'Blood pressure control and protection of the aging brain'. *Neurotherapeutics* **16** (3), 569-579

Wakefield, B.J., Bylund, C.L., Holman, J.E., Ray, A., Scherubel, M., Kienzle, M.G. & Rosenthal, G.E. (2008}) 'Nurse and patient communication profiles in a home-based telehealth intervention for heart failure management'. *PATIENT EDUCATION AND COUNSELING}* **71}** (2}), 285-292}

Wakefield, B.J., Holman, J.E., Ray, A., Scherubel, M., Adams, M.R., Hillis, S.L. & Rosenthal, G.E. (2011}) 'Effectiveness of Home Telehealth in Comorbid Diabetes and Hypertension: A Randomized, Controlled Trial'. *TELEMEDICINE AND E-HEALTH}* **17}** (4}), 254-261}

Wakefield, B.J., Holman, J.E., Ray, A., Scherubel, M., Adams, M.R., Hills, S.L. & Rosenthal, G.E. (2012}) 'Outcomes of a Home Telehealth Intervention for Patients with Diabetes and Hypertension'. *TELEMEDICINE AND E-HEALTH}* **18}** (8}), 575-579}

Wakefield, B.J. & Vaughan-Sarrazin, M. (2017}) 'Home Telehealth and Caregiving Appraisal in Chronic Illness'. *TELEMEDICINE AND E-HEALTH}* **23}** (4}), 282-289}

Walkden, J., McCullagh, P.J. & Kernohan, W.G. (2019) 'Patient and carer survey of remote vital sign telemonitoring for self-management of long-term conditions.'. *BMJ Health & Care Informatics* **26** (1)

Walker, P.P., Pompilio, P.P., Zanaboni, P., Bergmo, T.S., Prikk, K., Malinovschi, A., Montserrat, J.M., Middlemass, J., Sonc, S., Munaro, G., Marusic, D., Sepper, R., Rosso, R., Siriwardena, A.N., Janson, C., Farre, R., Calverley, P.M.A. & Dellaca, R.L. (2018}) 'Telemonitoring in Chronic Obstructive Pulmonary Disease (CHROMED) A Randomized Clinical Trial'. *AMERICAN JOURNAL OF RESPIRATORY AND CRITICAL CARE MEDICINE}* **198}** (5}), 620-628}

Walker, R.M., Gillespie, B.M., Thalib, L., Higgins, N.S. & Whitty, J.A. (2017) 'Foam dressings for treating pressure ulcers'. *Cochrane Database of Systematic Reviews* (10)

Wallace, E.L., Rosner, M.H., Alscher, M.D., Schmitt, C.P., Jain, A., Tentori, F., Firanek, C., Rheuban, K.S., Florez-Arango, J., Jha, V., Foo, M., de Blok, K., Marshall, M.R., Sanabria, M., Kudelka, T. & Sloand, J.A. (2017) 'Remote Patient Management for Home Dialysis Patients.'. *KI Reports* **2** (6), 1009-1017

Walters, E.H., Walters, J., Gibson, P.G. & Jones, P. (2003) 'Inhaled short acting beta2-agonist use in chronic asthma: regular versus as needed treatment'. *Cochrane Database of Systematic Reviews* (1)

Walterscheid, I., Biallawons, O. & Berens, P. (2019) 'Contactless Respiration and Heartbeat Monitoring of Multiple People Using a 2-D Imaging Radar.'. *Annual International Conference Of The IEEE Engineering In Medicine And Biology Society* **2019**, 3720-3725

Wang, A. (2015) 'Recent News'. *Journal MTM* **4** (1), 20-24

Wang, J., Barth, J., Göttgens, I., Emchi, K., Pach, D. & Oertelt-Prigione, S. (2020) 'An opportunity for patient-centered care: Results from a secondary analysis of sex-and gender-based data in mobile health trials for chronic medical conditions'. *Maturitas* **138**, 1-7

Wang, J., Wang, Y., Wei, C., Yao, N., Yuan, A., Shan, Y. & Yuan, C. (2014) 'Smartphone interventions for long-term health management of chronic diseases: an integrative review'. *Telemedicine and e-Health* **20** (6), 570-583

Wang, J.B., Cadmus-Bertram, L.A., Natarajan, L., White, M.M., Madanat, H., Nichols, J.F., Ayala, G.X. & Pierce, J.P. (2015) 'Wearable sensor/device (Fitbit One) and SMS text-messaging prompts to increase physical activity in overweight and obese adults: a randomized controlled trial'. *Telemedicine and e-Health* **21** (10), 782-792

Wang, L. & Alexander, C.A. (2020) 'Big data analytics in medical engineering and healthcare: methods, advances and challenges'. *Journal of medical engineering & technology* **44** (6), 267-283

Wang, L. & Alexander, C.A. (2014) 'Telemedicine based on mobile devices and mobile cloud computing'. *International Journal of Cloud Computing and Services Science* **3** (1), 26

Wang, Q. & Wang, Z. (2020) 'Big Data and Atrial Fibrillation: Current Understanding and New Opportunities'. *Journal of cardiovascular translational research* , 1-9

Wang, H., Hua, W., Ding, L., Wang, J., Chen, K. & Zhang, S. (2012) 'Home monitoring system improves the detection of ventricular arrhythmia and inappropriate shock.'. *Chinese medical journal* **125** (19), 3421-3424

Wang, P., Zhang, Y., Ma, Y., Liang, F., An, Q., Xue, H., Yu, X., Lv, H. & Wang, J. (2019) 'Method for Distinguishing Humans and Animals in Vital Signs Monitoring Using IR-UWB Radar.'. *International Journal of Environmental Research & Public Health [Electronic Resource]* **16** (22), 11 13

Wang, V., Smith, V.A., Bosworth, H.B., Oddone, E.Z., Olsen, M.K., McCant, F., Powers, B.J. & Van Houtven, C. (2012) 'Economic evaluation of telephone self-management interventions for blood pressure control'. *American Heart Journal* **163** (6), 980-986

Wang, W., den Brinker, A.C., Stuijk, S. & de Haan, G. (2017}) 'Robust heart rate from fitness videos'. *PHYSIOLOGICAL MEASUREMENT}* **38}** (6}), 1023-1044}

Wang, W., den Brinker, A.C., Stuijk, S. & de Haan, G. (2017) 'Robust heart rate from fitness videos.'. *Physiological Measurement* **38** (6), 1023-1044

Ward, M., Johnson, C., Klein, J., Farber, J.M., Nolin, W. & Peterson, M.J. (2020) 'Orthotics and Assistive Devices'. *Pediatric Rehabilitation: Principles and Practice* , 196

Warner, J.J., Crook, H.L., Whelan, K.M., Bleser, W.K., Roiland, R.A., Hamilton Lopez, M., Saunders, R.S., Wang, T.Y., Hernandez, A.F. & McClellan, M.B. (2020) 'Improving cardiovascular drug and device development and evidence through patient-centered research and clinical trials: a call to action from the value in healthcare initiative's partnering with regulators learning collaborative'. *Circulation: Cardiovascular Quality and Outcomes* **13** (7), e006606

Wasenius, N. (2014) 'Influence of exercise training on daily physical activity and risk factors for type 2 diabetes'.

Watanabe, E., Kasai, A., Fujii, E., Yamashiro, K. & Brugada, P. (2013) 'Reliability of implantable cardioverter defibrillator home monitoring in forecasting the need for regular office visits, and patient perspective. Japanese HOME-ICD study.'. *Circulation Journal* **77** (11), 2704-2711

Watanabe, E., Yamazaki, F., Goto, T., Asai, T., Yamamoto, T., Hirooka, K., Sato, T., Kasai, A., Ueda, M., Yamakawa, T., Ueda, Y., Yamamoto, K., Tokunaga, T., Sugai, Y., Tanaka, K., Hiramatsu, S., Arakawa, T., Schrader, J., Varma, N. & Ando, K. (2020) 'Remote Management of Pacemaker Patients With Biennial In-Clinic Evaluation: Continuous Home Monitoring in the Japanese At-Home Study: A Randomized Clinical Trial.'. *Circulation: Arrhythmia and Electrophysiology* **13** (5), e007734

Webb, V.L. & Wadden, T.A. (2017) 'Intensive lifestyle intervention for obesity: principles, practices, and results'. *Gastroenterology* **152** (7), 1752-1764

Webster, E.K., Kracht, C.L., Newton Jr, R.L., Beyl, R.A. & Staiano, A.E. (2020) 'Intervention to Improve Preschool Children’s Fundamental Motor Skills: Protocol for a Parent-Focused, Mobile App–Based Comparative Effectiveness Trial'. *JMIR Research Protocols* **9** (10), e19943

Webster, D.E., Tummalacherla, M., Higgins, M., Wing, D., Ashley, E., Kelly, V.E., McConnell, M.V., Muse, E.D., Olgin, J.E., Mangravite, L.M., Godino, J., Kellen, M.R. & Omberg, L. (2021) 'Smartphone-Based VO2max Measurement With Heart Snapshot in Clinical and Real-world Settings With a Diverse Population: Validation Study.'. *JMIR MHealth and UHealth* **9** (6), e26006

Weeks, G., George, J., Maclure, K. & Stewart, D. (2016) 'Non-medical prescribing versus medical prescribing for acute and chronic disease management in primary and secondary care'. *Cochrane Database of Systematic Reviews* (11)

Wei, I., Pappas, Y., Car, J., Sheikh, A. & Majeed, A. (2011) 'Computer-assisted versus oral-and-written dietary history taking for diabetes mellitus'. *Cochrane Database of Systematic Reviews* (12)

Weichenthal, S., Kulka, R., Dubeau, A., Martin, C., Wang, D. & Dales, R. (2011) 'Traffic-related air pollution and acute changes in heart rate variability and respiratory function in urban cyclists'. *Environmental health perspectives* **119** (10), 1373-1378

Weiss, A.R., Johnson, N.L., Berger, N.A. & Redline, S. (2010) 'Validity of activity-based devices to estimate sleep'. *Journal of clinical sleep medicine : JCSM : official publication of the American Academy of Sleep Medicine* **6** (4), 336-342

Welch, K.C., Harnett, C. & Lee, Y. (2019) 'A review on measuring affect with practical sensors to monitor driver behavior'. *Safety* **5** (4), 72

Welch, V., Tugwell, P., Petticrew, M., de Montigny, J., Ueffing, E., Kristjansson, B., McGowan, J., Benkhalti Jandu, M., Wells, G.A. & Brand, K. (2010) 'How effects on health equity are assessed in systematic reviews of interventions'. *Cochrane Database of Systematic Reviews* (12)

Welland, A. (2017) 'Electrification of health clinics in rural areas: Challenges and opportunities'.

Wembe, O. (2015) 'Momentum: Assisting heart patients with workout intensity'.

Wenger, N.K., Williams, O.O. & Parashar, S. (2019) 'SMARTWOMAN™: feasibility assessment of a smartphone APP to control cardiovascular risk factors in vulnerable diabetic women'. *Clinical cardiology* **42** (2), 217-221

Whellan, D.J. (2005) 'Heart failure disease management: implementation and outcomes'. *Cardiology in review* **13** (5), 231-239

WhiteWilliams C., Unruh L. & Ward, K. (2015) 'Hospital utilization after a telemonitoring program: a pilot study.'. *Home health care services quarterly* **34** (1), 1-13

White-Williams, C., Unruh, L. & Ward, K. (2015) 'Hospital utilization after a telemonitoring program: a pilot study.'. *Home health care services quarterly* **34** (1), 1-13

Whittaker, R., McRobbie, H., Bullen, C., Rodgers, A., Gu, Y. & Dobson, R. (2019) 'Mobile phone text messaging and app-based interventions for smoking cessation'. *Cochrane Database of Systematic Reviews* (10)

Whitten, P., Bergman, A., Meese, M.A., Bridwell, K. & Jule, K. (2009) 'St. Vincent's Home telehealth for congestive heart failure patients.'. *Telemedicine Journal & E-Health* **15** (2), 148-153

Widlansky, M.E., Gokce, N., Keaney, J.F. & Vita, J.A. (2003) 'The clinical implications of endothelial dysfunction'. *Journal of the American College of Cardiology* **42** (7), 1149-1160

Wieringa, F.P., Broers, N.J.H., Kooman, J.P., Van Der Sande, F.M. & Van Hoof, C. (2017) 'Wearable sensors: can they benefit patients with chronic kidney disease?'. *Expert Review of Medical Devices* **14** (7), 505-519

Wilhelm, S., Weingarden, H., Ladis, I., Braddick, V., Shin, J. & Jacobson, N.C. (2020) 'Cognitive-behavioral therapy in the digital age: presidential address'. *Behavior therapy* **51** (1), 1-14

Williams C. & Wan, T.T. (2016) 'A cost analysis of remote monitoring in a heart failure program.'. *Home health care services quarterly* **35** (3-4), 112-122

Williams, A.D., Bird, M., Hardcastle, S., Kirschbaum, M., Ogden, K.J. & Walters, J. (2018) 'Exercise for reducing falls in people living with and beyond cancer'. *Cochrane Database of Systematic Reviews* (10)

Williams, C. & Wan, T.T.H. (2016) 'A cost analysis of remote monitoring in a heart failure program.'. *Home health care services quarterly* **35** (3-4), 112-122

Williams, C. & Wan, T.T.H. (2016) 'The Influence of Remote Monitoring on Clinical Decision Making'. *Home Health Care Management & Practice* **28** (2), 86-93

Williams, C. & Wan, T.T.H. (2016) 'A remote monitoring program evaluation: a retrospective study'. *Journal of evaluation in clinical practice* **22** (6), 978-988

Williamson, T.M., Moran, C., McLennan, A., Seidel, S., Ma, P.P., Koerner, M. & Campbell, T.S. (2020) 'Promoting adherence to physical activity among individuals with cardiovascular disease using behavioral counseling: A theory and research-based primer for health care professionals'. *Progress in cardiovascular diseases*

Willmott, T.J., Pang, B., Rundle-Thiele, S. & Badejo, A. (2019) 'Weight management in young adults: Systematic review of electronic health intervention components and outcomes'. *Journal of medical Internet research* **21** (2), e10265

Winders, W.T., Garbern, S.C., Bills, C.B., Relan, P., Schultz, M.L., Trehan, I., Kivlehan, S.M., Becker, T.K. & McQuillan, R. (2021) 'The effects of mobile health on emergency care in low-and middle-income countries: A systematic review and narrative synthesis'. *Journal of Global Health* **11**

Winkler, S., Schieber, M., Lucke, S., Heinze, P., Schweizer, T., Wegertseder, D., Scherf, M., Nettlau, H., Henke, S., Braecklein, M., Anker, S.D. & Koehler, F. (2011) 'A new telemonitoring system intended for chronic heart failure patients using mobile telephone technology--feasibility study.'. *International journal of cardiology* **153** (1), 55-58

Winnige, P., Vysoky, R., Dosbaba, F. & Batalik, L. (2021) 'Cardiac rehabilitation and its essential role in the secondary prevention of cardiovascular diseases'. *World journal of clinical cases* **9** (8), 1761

Wiysonge, C.S., Abdullahi, L.H., Ndze, V.N. & Hussey, G.D. (2016) 'Public stewardship of private for-profit healthcare providers in low- and middle-income countries'. *Cochrane Database of Systematic Reviews* (8)

Wiysonge, C.S., Paulsen, E., Lewin, S., Ciapponi, A., Herrera, C.A., Opiyo, N., Pantoja, T., Rada, G. & Oxman, A.D. (2017) 'Financial arrangements for health systems in low-income countries: an overview of systematic reviews'. *Cochrane Database of Systematic Reviews* (9)

Wohlfahrt, P., Stehlik, J., Pan, I.Z. & Ryan, J.J. (2020) 'Empowering People Living with Heart Failure'. *Heart Failure Clinics* **16** (4), 409-420

Wolfe, D.A. (2012) *ACCELEROMETER-BASED PATTERNS OF PHYSICAL ACTIVITY BY WEIGHT STATUS AND GENDER AMONG US ADULTS: NATIONAL HEALTH AND NUTRITION EXAMINATION SURVEY 2003-2006*

Wolfenden, L., Barnes, C., Jones, J., Finch, M., Wyse, R.J., Kingsland, M., Tzelepis, F., Grady, A., Hodder, R.K. & Booth, D. (2020) 'Strategies to improve the implementation of healthy eating, physical activity and obesity prevention policies, practices or programmes within childcare services'. *Cochrane Database of Systematic Reviews* (2)

Woo, K.N. (2012) 'Usability of Medication Adherence Technologies among Older Adults'.

Woodley, S.J., Lawrenson, P., Boyle, R., Cody, J.D., Mørkved, S., Kernohan, A. & Hay-Smith, E. (2020) 'Pelvic floor muscle training for preventing and treating urinary and faecal incontinence in antenatal and postnatal women'. *Cochrane Database of Systematic Reviews* (5)

Woollard, M., Pitt, K., Hayward, A.J. & Taylor, N.C. (2005) 'Limited benefits of ambulance telemetry in delivering early thrombolysis: a randomised controlled trial.'. *Emergency Medicine Journal* **22** (3), 209-215

Wortley, D., An, J.Y. & Heshmati, A. (2017) 'Tackling the Challenge of the Aging Society: Detecting and Preventing Cognitive and Physical Decline through Games and Consumer Technologies'. *Healthcare informatics research* **23** (2), 87-93

Wray, A., Olstad, D.L. & Minaker, L.M. (2018) 'Smart prevention: a new approach to primary and secondary cancer prevention in smart and connected communities'. *Cities* **79**, 53-69

Wright, S.P., Hall Brown, T.S., Collier, S.R. & Sandberg, K. (2017) 'How consumer physical activity monitors could transform human physiology research'. *American Journal of Physiology-Regulatory, Integrative and Comparative Physiology* **312** (3), R358-R367

Wu, D.T., Xin, C., Bindhu, S., Xu, C., Sachdeva, J., Brown, J.L. & Jung, H. (2020) 'Clinician Perspectives and Design Implications in Using Patient-Generated Health Data to Improve Mental Health Practices: Mixed Methods Study'. *JMIR formative research* **4** (8), e18123

Wu, D., Fang, D., Wang, R., Deng, D. & Liao, S. (2020) 'Management of Pregnancy during the COVID-19 Pandemic'. *Global Challenges* **2000052**

Wu, S., Kutlubaev, M.A., Chun, H., Cowey, E., Pollock, A., Macleod, M.R., Dennis, M., Keane, E., Sharpe, M. & Mead, G.E. (2015) 'Interventions for post-stroke fatigue'. *Cochrane Database of Systematic Reviews* (7)

Wu, T., Liu, G.J., Li, P. & Clar, C. (2002) 'Iodised salt for preventing iodine deficiency disorders'. *Cochrane Database of Systematic Reviews* (3)

Xi H., Gan G., Zhang H. & Chen, C. (2015) 'Design of Smart Care Tele-Monitoring System for Mother and Fetus.'. *Zhongguo yi liao qi xie za zhi = Chinese journal of medical instrumentation* **39** (2), 102-104

Xia, Y., Liu, T. & He, N. (2014) 'Point-of-Care Testing Technologies and Applications in Family'. *Journal of Bionanoscience* **8** (3), 151-159

Yan, A.F., Stevens, P., Wang, Y., Weinhardt, L., Holt, C.L., O'Connor, C., Feller, T., Xie, H. & Luelloff, S. (2015) 'mHealth text messaging for physical activity promotion in college students: a formative participatory approach'. *American Journal of Health Behavior* **39** (3), 395-408

Yang, H., Zhang, F., Peng, X., Zhao, D. & Peng, J. (2014) 'Efficacy of medication directed by home-monitoring cardiac resynchronization therapy in chronic heart failure patients.'. *Chinese Medical Sciences Journal* **29** (1), 61-62

Yang, H., Dervin, G., Madden, S., Beaule, P.E., Gagne, S., Crossan, M.L., Fayad, A., Wheeler, K., Afagh, M., Zhang, T. & Taljaard, M. (2018) 'Postoperative Home Monitoring After Joint Replacement: Feasibility Study.'. *JMIR Perioperative Medicine* **1** (2), e10168

Yang, X., Zhang, K. & He, J. (2021) 'Application and Clinical Analysis of Remote Fetal Heart Rate Monitoring Platform in Continuous Fetal Heart Rate Monitoring Images.'. *Journal of Healthcare Engineering* **2021**, 5517692

Yatabe, J., Yatabe, M.S. & Ichihara, A. (2020) 'The current state and future of internet technology-based hypertension management in Japan'. *Hypertension Research* , 1-10

Yatabe, M.S., Yatabe, J., Asayama, K., Staessen, J.A., Mujaj, B., Thijs, L., Ito, K., Sonoo, T., Morimoto, S. & Ichihara, A. (2018) 'The rationale and design of reduction of uncontrolled hypertension by Remote Monitoring and Telemedicine (REMOTE) study.'. *Blood pressure* **27** (2), 99-105

Ye, R., Shi, R., Liu, K., Zhang, X., Wang, S., Liao, H., Li, X., Gou, Q., Rong, X. & Zhang, Z. (2020) 'Internet-based patient- primary care physician-cardiologist integrated management model of hypertension in China: study protocol for a multicentre randomised controlled trial'. *BMJ open* **10** (10)

Yee, R., Verma, A., Beardsall, M., Fraser, J., Philippon, F. & Exner, D.V. (2013) 'Canadian Cardiovascular Society/Canadian Heart Rhythm Society joint position statement on the use of remote monitoring for cardiovascular implantable electronic device follow-up'. *Canadian Journal of Cardiology* **29** (6), 644-651

Yi, L.Y., Samat, N. & Muda, W.M.W. (2017) 'Accelerometer-Measured Physical Activity and its Relationship with Body Mass Index (BMI) and Waist Circumference (WC) Measurements: A Cross-Sectional Study on Malaysian Adults.'. *Malaysian Journal of Nutrition* **23** (3)

Yin Tan, S., Praveena, S.M., Abidin, E.Z., Cheema, M.S. & Tan, S.Y. (2016) 'A review of heavy metals in indoor dust and its human health-risk implications'. *Reviews on environmental health* **31** (4), 447-456

Young, L., Hertzog, M. & Barnason, S. (2017) 'Feasibility of using accelerometer measurements to assess habitual physical activity in rural heart failure patients'. *Geriatrics* **2** (3), 23

Young, T. & Busgeeth, K. (2010) 'Home-based care for reducing morbidity and mortality in people infected with HIV/AIDS'. *Cochrane Database of Systematic Reviews* (1)

Yu, S. & Yunfei, Q. 'Energy-efficient IoT based improved health monitoring system for sports persons'. *Journal of Intelligent & Fuzzy Systems* (Preprint), 1-11

Yu, X. & Xiong, S. (2019) 'A dynamic time warping based algorithm to evaluate kinect-enabled home-based physical rehabilitation exercises for older people'. *Sensors* **19** (13), 2882

Yuan, N., Pevnick, J.M., Botting, P.G., Elad, Y., Miller, S.J., Cheng, S. & Ebinger, J.E. (2021) 'Patient Use and Clinical Practice Patterns of Remote Cardiology Clinic Visits in the Era of COVID-19'. *JAMA network open* **4** (4), e214157-e214157

Zahra, S.R. & Chishti, M.A. (2020) 'A collaborative edge-cloud internet of things based framework for securing the indian healthcare system'. *International Journal of Sensors Wireless Communications and Control* **10** (4), 440-457

Zanotto, G., D'Onofrio, A., Della Bella, P., Solimene, F., Pisano, E.C., Iacopino, S., Dondina, C., Giacopelli, D., Gargaro, A. & Ricci, R.P. (2019) 'Organizational model and reactions to alerts in remote monitoring of cardiac implantable electronic devices: A survey from the Home Monitoring Expert Alliance project.'. *Clinical cardiology* **42** (1), 76-83

Zarakovitis, K., Angelidis, P., Kourtidou-Papadeli, C. & Psymarnou, M. (2004) 'Ambulatory monitoring for chronic cardiac and pulmonary patients.'. *Studies in Health Technology & Informatics* **103**, 362-367

Zartner, P.A., Toussaint-Goetz, N., Photiadis, J., Wiebe, W. & Schneider, M.B. (2012}) 'Telemonitoring with implantable electronic devices in young patients with congenital heart diseases'. *EUROPACE}* **14}** (7}), 1030-1037}

Zhang, J. & Centola, D. (2019) 'Social networks and health: New developments in diffusion, online and offline'. *Annual Review of Sociology* **45**, 91-109

ZHANG, M., SAEED, R., SAEED, S., STANKOVSKI, S. & ZHANG, X. 'Wearable Technology and Applications: A Systematic'.

Zhang, Y., Fang, Y., Xu, Y., Xiong, P., Zhang, J., Yang, J., Ran, L. & Tan, X. (2020) 'Adherence with blood pressure monitoring wearable device among the elderly with hypertension: The case of rural China'. *Brain and behavior* **10** (6), e01599

Zhang, Y., Yang, N., Si, G., Zhang, Y., Dong, Z., Huang, Y. & Tan, X. (2020) 'What matters the adherence with BP 24-hr self-monitoring wearable device among hypertensive patients? A population-based survey'. *Translational behavioral medicine* **10** (4), 1053-1063

Zhao, F., Li, M., Qian, Y. & Tsien, J.Z. (2013) 'Remote measurements of heart and respiration rates for telemedicine.'. *PLoS ONE [Electronic Resource]* **8** (10), e71384

Zhao, F., Li, M. & Tsien, J.Z. (2015) 'Technology platforms for remote monitoring of vital signs in the new era of telemedicine'. *Expert Review of Medical Devices* **12** (4), 411-429

Zhao, W., Zhang, J., Sadowsky, M.G., Meng, R., Ding, Y. & Ji, X. (2018) 'Remote ischaemic conditioning for preventing and treating ischaemic stroke'. *Cochrane Database of Systematic Reviews* (7)

Zhao, X., Fei, D., Doarn, C.R., Harnett, B. & Merrell, R. (2004) 'A telemedicine system for wireless home healthcare based on Bluetooth and the Internet.'. *Telemedicine Journal & E-Health* **10** (Suppl 2), S-110-6

Zhou, H., Al-Ali, F., Wang, C., Hamad, A., Ibrahim, R., Talal, T. & Najafi, B. (2020) 'Harnessing digital health to objectively assess cognitive impairment in people undergoing hemodialysis process: The Impact of cognitive impairment on mobility performance measured by wearables'. *PloS one* **15** (4), e0225358

Zhu, X., Chen, W., Nemoto, T., Kitamura, K. & Wei, D. (2010) 'Long-term monitoring of heart rate, respiration rhythm, and body movement during sleep based upon a network.'. *Telemedicine Journal & E-Health* **16** (2), 244-253

Zhu, X., Chen, W., Tang, Z., Nemoto, T. & Wei, D. (2008) 'Automatic home care system for monitoring HR/RR during sleep.'. *Annual International Conference Of The IEEE Engineering In Medicine And Biology Society* **2008**, 522-525

Zhuo, Q., Yuan, Z., Chen, H. & Wu, T. (2010) 'Traditional Chinese herbal products for stable angina'. *Cochrane Database of Systematic Reviews* (5)

Zobair, K.M. (2019) 'Barriers, Facilitators and Expectations of Telemedicine Healthcare Services Adoption in Rural Public Hospital Settings in Bangladesh'. *Unpublished Thesis).Retrieved from http://hdl.handle.net/10072/388646*

Zobair, K.M., Sanzogni, L. & Sandhu, K. (2020) 'Telemedicine healthcare service adoption barriers in rural Bangladesh'. *Australasian Journal of Information Systems* **24**

Zoorob, R., Buchowski, M.S., Beech, B.M., Canedo, J.R., Chandrasekhar, R., Akohoue, S. & Hull, P.C. (2013) 'Healthy families study: Design of a childhood obesity prevention trial for Hispanic families'. *Contemporary clinical trials* **35** (2), 108-121

Zucchella, C., Sinforiani, E., Tamburin, S., Federico, A., Mantovani, E., Bernini, S., Casale, R. & Bartolo, M. (2018) 'The multidisciplinary approach to Alzheimer's disease and dementia. A narrative review of non-pharmacological treatment'. *Frontiers in neurology* **9**, 1058

Zulfiqar, A.A., Hajjam, A. & Andres, E. (2019) 'Focus on the Different Projects of Telemedicine Centered on the Elderly In France'. *Current Aging Science* **11** (4), 202-215

Zulfiqar, A., Lorenzo-Villalba, N., Zulfiqar, O., Hajjam, M., Courbon, Q., Esteoulle, L., Geny, B., Talha, S., Letourneau, D., Hajjam, J., Erve, S., Hajjam El Hassani, A. & Andres, E. (2020) 'e-Health: A Future Solution for Optimized Management of Elderly Patients. GER-e-TEC TM Project.'. *Medicines* **7** (8)

Zuurbier, S.M. & Al-Shahi Salman, R. (2019) 'Interventions for treating brain arteriovenous malformations in adults'. *Cochrane Database of Systematic Reviews* (9)

Zvolensky, M.J., Garey, L., Rogers, A.H., Schmidt, N.B., Vujanovic, A.A., Storch, E.A., Buckner, J.D., Paulus, D.J., Alfano, C., Smits, J.A.J. & O'Cleirigh, C. (2020) 'Psychological, addictive, and health behavior implications of the COVID-19 pandemic'. *Behaviour research and therapy* **134**, 103715

Grey Literature

https://pure.uhi.ac.uk/en/publications/relationship-between-physical-and-psychosocial-factors-and-attend

https://www.isrctn.com/ISRCTN53784122?q=cardiac%20rehab*&filters=&sort=&offset=1&totalResults=9&page=1&pageSize=10&searchType=basic-search

https://ethos.bl.uk/OrderDetails.do?did=11&uin=uk.bl.ethos.773773

https://www.gov.uk/government/speeches/building-a-digitally-enabled-nhs

https://www.gmc-uk.org/-/media/documents/regulatory-approaches-to-telemedicine_docx-73978543.docx

https://www.nihr.ac.uk/about-us/our-impact/making-a-difference-stories.htm?postid=27607

https://www.nihr.ac.uk/news/successful-home-based-heart-rehabilitation-programme-developed-in-south-west-rolled-out/20290

https://www.nihr.ac.uk/documents/2150-national-evaluation-of-nhs-home-supporting-document/27445

https://www.nihr.ac.uk/blog/assistive-technology-whats-its-proper-role-in-the-future-of-health-and-social-care/10996

https://www.nihr.ac.uk/documents/implantable-and-wearable-medical-devices-for-chronic-obstructive-pulmonary-disease/11943

https://www.nihr.ac.uk/news/artificial-intelligence-research-to-speed-up-cancer-and-heart-care-as-part-of-nhs-ai-lab/25623

https://www.nihr.ac.uk/news/new-wave-of-ai-technologies-in-36-million-funding-boost/27867

https://www.nihr.ac.uk/documents/resource-guide-for-community-engagement-and-involvement-in-global-health-research/27077

https://www.hdruk.ac.uk/case-studies/radar-base-mhealth-platform/

https://www.hdruk.ac.uk/news/computable-knowledge-part-1-of-2-what-is-it-and-why-do-we-need-it-to-build-a-learning-health-system/

https://www.hdruk.ac.uk/news/improving-the-nations-cardio-vascular-health-the-bhf-data-science-centre/

https://www.hdruk.ac.uk/news/csd3-supercomputer-what-does-it-mean-for-hdr-uk/

https://www.hdruk.ac.uk/resources/associations-between-the-home-physical-environment-and-childrens-home-based-physical-activity-and-sitting/

https://www.hdruk.ac.uk/news/implementing-technology-well-a-critical-challenge-for-enabling-better-care-in-a-learning-health-system/

https://www.hdruk.ac.uk/projects/remote-by-default-care-in-the-covid-19-pandemic-addressing-the-challenges-of-a-radical-new-service-model/

https://www.hdruk.ac.uk/news/its-taken-a-pandemic-to-bring-the-uks-health-data-community-together/

https://www.gmc-uk.org/-/media/documents/a-scoping-review-of-evidence-relating-to-communication-failures-that-lead-to-patient-harm_p-80569509.pdf

https://www.gmc-uk.org/ethical-guidance/learning-materials/remote-consultations-flowchart

https://www.cqc.org.uk/guidance-providers/all-services/find-out-if-you-need-consent-use-technology-part-someones-care

https://www.cqc.org.uk/guidance-providers/all-services/innovative-use-technology-can-help-answer-our-five-key-questions

https://www.cqc.org.uk/guidance-providers/registration/transport-services-triage-medical-advice-provided-remotely

https://www.cqc.org.uk/news/stories/protecting-people-who-use-online-healthcare-services

https://www.gov.uk/government/publications/uk-digital-strategy

https://www.gov.uk/government/publications/code-of-conduct-for-data-driven-health-and-care-technology

https://www.gov.uk/guidance/design-your-evaluation-evaluating-digital-health-products

https://www.gov.uk/guidance/health-app-developers-the-assessment-process

https://www.gov.uk/guidance/get-started-evaluating-digital-health-products

https://www.gov.uk/research-for-development-outputs/mortality-reductions-in-patients-receiving-exercise-based-cardiac-rehabilitation-how-much-can-be-attributed-to-cardiovascular-risk-factor-improvements

https://www.gov.uk/government/news/36-million-boost-for-ai-technologies-to-revolutionise-nhs-care

https://www.gov.uk/government/news/digital-innovations-tested-to-support-vulnerable-people-during-covid-19-outbreak

https://www.gov.uk/government/publications/human-augmentation-the-dawn-of-a-new-paradigm

https://www.gov.uk/government/speeches/my-vision-for-a-more-tech-driven-nhs

https://www.gov.uk/guidance/usability-testing-qualitative-studies

https://www.gov.uk/government/publications/competition-wearable-technology-for-injury-prevention

https://ethos.bl.uk/OrderDetails.do?did=41&uin=uk.bl.ethos.765195

https://ethos.bl.uk/OrderDetails.do?did=1&uin=uk.bl.ethos.540360

https://www.isrctn.com/ISRCTN70212111?q=%22Home%20based%22%20and%20exercise%20AND%20(cardiac%20OR%20heart)&filters=&sort=&offset=43&totalResults=51&page=5&pageSize=10&searchType=basic-search

https://www.isrctn.com/ISRCTN51615566?q=%22Home%20based%22%20and%20exercise%20AND%20(cardiac%20OR%20heart)&filters=&sort=&offset=44&totalResults=51&page=5&pageSize=10&searchType=basic-search

https://www.isrctn.com/ISRCTN58458272?q=%22Home%20based%22%20and%20exercise%20AND%20(cardiac%20OR%20heart)&filters=&sort=&offset=50&totalResults=51&page=5&pageSize=10&searchType=basic-search

https://www.isrctn.com/ISRCTN16805986?q=wearable*&filters=&sort=&offset=3&totalResults=65&page=1&pageSize=10&searchType=advanced-search

https://www.isrctn.com/ISRCTN18022985?q=wearable*&filters=&sort=&offset=9&totalResults=65&page=1&pageSize=10&searchType=advanced-search

https://www.isrctn.com/ISRCTN12246987?q=wearable*&filters=&sort=&offset=20&totalResults=65&page=2&pageSize=10&searchType=advanced-search

https://www.isrctn.com/ISRCTN61479659?q=wearable*&filters=&sort=&offset=54&totalResults=65&page=6&pageSize=10&searchType=advanced-search

https://www.isrctn.com/ISRCTN13860094?q=cardiac%20rehab*&filters=&sort=&offset=2&totalResults=9&page=1&pageSize=10&searchType=basic-search

https://www.isrctn.com/ISRCTN57096451?q=cardiac%20rehab*&filters=&sort=&offset=5&totalResults=9&page=1&pageSize=10&searchType=basic-search

https://www.isrctn.com/ISRCTN65746249?q=cardiac%20rehab*&filters=&sort=&offset=6&totalResults=9&page=1&pageSize=10&searchType=basic-search

https://www.isrctn.com/ISRCTN86234930?q=%22Home%20based%22%20and%20exercise%20AND%20(cardiac%20OR%20heart)&filters=&sort=&offset=24&totalResults=51&page=3&pageSize=10&searchType=basic-search

https://www.isrctn.com/ISRCTN25032672?q=%22Home%20based%22%20and%20exercise%20AND%20(cardiac%20OR%20heart)&filters=&sort=&offset=27&totalResults=51&page=3&pageSize=10&searchType=basic-search

https://www.isrctn.com/ISRCTN72884263?q=%22Home%20based%22%20and%20exercise%20AND%20(cardiac%20OR%20heart)&filters=&sort=&offset=51&totalResults=51&page=6&pageSize=10&searchType=basic-search

https://www.isrctn.com/ISRCTN22197467?q=wearable*&filters=&sort=&offset=2&totalResults=65&page=1&pageSize=10&searchType=advanced-search

https://www.isrctn.com/ISRCTN21782066?q=wearable*&filters=&sort=&offset=34&totalResults=65&page=4&pageSize=10&searchType=advanced-search

https://www.dart-europe.org/full.php?id=2163043

https://www.dart-europe.org/full.php?id=86305

https://www.dart-europe.org/full.php?id=267403

https://www.dart-europe.org/full.php?id=1229565

https://www.dart-europe.org/full.php?id=416149

https://www.dart-europe.org/full.php?id=16543

https://www.dart-europe.org/full.php?id=2295319

https://www.dart-europe.org/full.php?id=1202033

https://www.dart-europe.org/full.php?id=38349

https://www.dart-europe.org/full.php?id=2088407

https://www.dart-europe.org/full.php?id=565659

http://www.opengrey.eu/item/display/10068/990417

http://www.opengrey.eu/item/display/10068/592956

http://www.opengrey.eu/handle/10068/986685

http://www.opengrey.eu/handle/10068/551272

http://www.opengrey.eu/item/display/10068/601028

http://www.opengrey.eu/item/display/10068/952085

http://www.opengrey.eu/item/display/10068/1012774

http://www.opengrey.eu/item/display/10068/993180

http://www.opengrey.eu/item/display/10068/986829

http://www.opengrey.eu/item/display/10068/967503

http://www.opengrey.eu/item/display/10068/938695

http://www.opengrey.eu/item/display/10068/913782

http://www.opengrey.eu/item/display/10068/905084

http://www.opengrey.eu/item/display/10068/576303

http://www.opengrey.eu/item/display/10068/1007881

http://www.opengrey.eu/item/display/10068/995349

http://www.opengrey.eu/item/display/10068/964401

http://www.opengrey.eu/item/display/10068/939593

https://pure.uhi.ac.uk/en/publications/home-based-cardiac-rehabilitation-a-review

https://pure.uhi.ac.uk/en/publications/home-versus-hospital-based-cardiac-rehabilitation-a-systematic-re

https://pure.uhi.ac.uk/en/projects/assessing-the-usability-and-adherence-to-wearable-technology-and-

https://pure.uhi.ac.uk/en/publications/barriers-and-facilitators-to-participating-in-cardiac-rehabilitat

https://pure.uhi.ac.uk/en/publications/measurement-of-heart-rate-using-the-polar-oh1-and-fitbit-charge-3

https://pure.uhi.ac.uk/en/publications/caalyx-a-new-generation-of-location-based-services-in-healthcare

https://pure.uhi.ac.uk/en/publications/how-smartphones-are-changing-the-face-of-mobile-and-participatory

https://pure.uhi.ac.uk/en/publications/cardiac-rehabilitation-a-euro-postcode-lottery

https://pure.uhi.ac.uk/en/publications/the-influence-of-non-modifiable-illness-perceptions-on-attendance

https://pure.uhi.ac.uk/en/clippings/patients-urged-to-attend-cardiac-rehab

https://pure.uhi.ac.uk/en/clippings/health-literacy-for-cardiac-rehabilitation-an-examination-of-asso

https://pure.uhi.ac.uk/en/publications/healthy-hearts-a-community-based-primary-prevention-programme-to-

https://pure.uhi.ac.uk/en/publications/digital-technology-interventions-for-risk-factor-modification-in-

https://pure.uhi.ac.uk/en/publications/health-literacy-for-cardiac-rehabilitation-an-examination-of-asso

https://ethos.bl.uk/OrderDetails.do?did=23&uin=uk.bl.ethos.779871

https://ethos.bl.uk/OrderDetails.do?did=1&uin=uk.bl.ethos.411820

https://ethos.bl.uk/OrderDetails.do?did=4&uin=uk.bl.ethos.579596

https://ethos.bl.uk/OrderDetails.do?did=7&uin=uk.bl.ethos.722474

https://ethos.bl.uk/OrderDetails.do?did=15&uin=uk.bl.ethos.649403

https://ethos.bl.uk/OrderDetails.do?did=69&uin=uk.bl.ethos.580530
https://ethos.bl.uk/OrderDetails.do?did=2&uin=uk.bl.ethos.619172

https://ethos.bl.uk/OrderDetails.do?did=1&uin=uk.bl.ethos.567100

https://ethos.bl.uk/OrderDetails.do?did=3&uin=uk.bl.ethos.667848
